# Desymmetrization of Prochiral *N*-Pyrazolyl Maleimides via Organocatalyzed Asymmetric Michael Addition with Pyrazolones: Construction of Tri-*N*-Heterocyclic Scaffolds Bearing Both Central and Axial Chirality

**DOI:** 10.3390/molecules28114279

**Published:** 2023-05-23

**Authors:** Jianqi Geng, Xingfu Wei, Biru He, Yuting Hao, Jingping Qu, Baomin Wang

**Affiliations:** State Key Laboratory of Fine Chemicals, Department of Pharmaceutical Sciences, School of Chemical Engineering, Dalian University of Technology, 2 Linggong Road, Dalian 116024, China; 17863920817@163.com (J.G.); xingfuwei0230@163.com (X.W.); biruhe@mail.dlut.edu.cn (B.H.); 17361567236@163.com (Y.H.); qujp@dlut.edu.cn (J.Q.)

**Keywords:** desymmetrization, tri-*N*-heterocycle, C–N chiral axis, asymmetric Michael addition

## Abstract

The desymmetrization of *N*-pyrazolyl maleimides was realized through an asymmetric Michael addition by using pyrazolones under mild conditions, leading to the formation of a tri-*N*-heterocyclic pyrazole–succinimide–pyrazolone assembly in high yields with excellent enantioselectivities (up to 99% yield, up to 99% ee). The use of a quinine-derived thiourea catalyst was essential for achieving stereocontrol of the vicinal quaternary–tertiary stereocenters together with the C–N chiral axis. Salient features of this protocol included a broad substrate scope, atom economy, mild conditions and simple operation. Moreover, a gram-scale experiment and derivatization of the product further illustrated the practicability and potential application value of this methodology.

## 1. Introduction

**Scheme 1:**
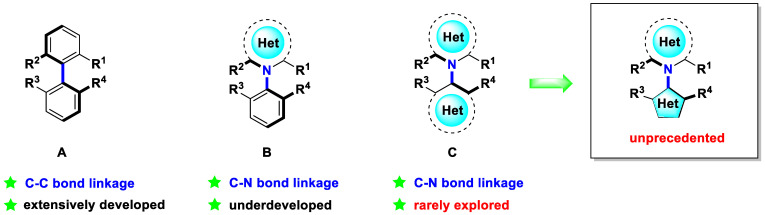
Representative axially chiral skeletons bearing a C–C or C–N axis. (**A**): C–C axially chiral skeletons; (**B**): C–N axially chiral skeletons; (**C**): C–N bi-heterocyclic axially chiral skeletons. Het: heterocyclic ring.

As a prominent stereochemical feature, axial chirality is frequently seen in natural products [1,2], drugs [3,4,5], biologically active molecules [6,7,8] and has also found wide applications in asymmetric catalysis as chiral ligands [9,10,11] or organocatalysts [12]. Among numerous axially chiral structures, biaryl compounds having a rotationally hindered C–C axis have been well explored in the past decades (Scheme 1A) [9,13,14,15,16,17,18,19,20]. In comparison, the asymmetric construction of axially biaryl skeletons bearing a C–N axis has been relatively underdeveloped due to their higher degree of rotational freedom and lower conformational stability (Scheme 1B) [21,22,23,24,25,26]. However, until now, axially chiral bi-heteroaryl scaffolds based on a C–N bond linkage has rarely been explored. Moreover, the study of axially chiral five-membered bi-*N*-heterocycle skeletons has not previously been reported, which is probably because of the smaller bond angle leading to poorer stability when compared to six-membered aryl or heteroaryl scaffolds. Therefore, developing an efficient approach to the synthesis of five-membered bi-heterocyclic scaffolds bearing axial chirality is meaningful and desirable (Scheme 1C).

**Scheme 2:**
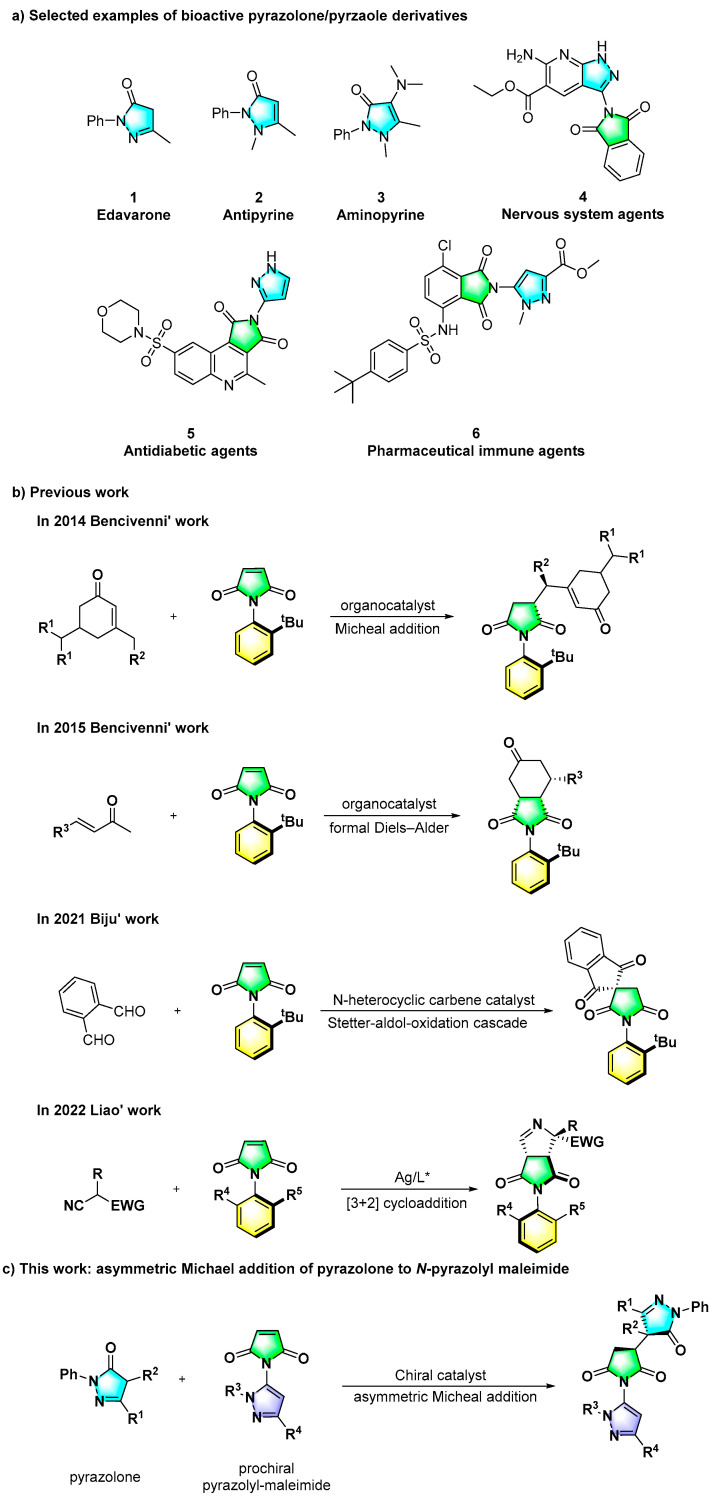
Previous strategies to achieve axially chiral scaffolds via desymmetrization and our strategy [37,38,39,40].

Pyrazoles and pyrazolones are among the important five-membered *N*-heterocycles that can be found in numerous bioactive molecules and drugs, possessing unique biological and pharmacological activities (Scheme 2a) [27,28,29]. For example, edaravone (**1**) is a neuroprotective agent [30], and aminopyrine (**2**) and antipyrine (**3**) are used to treat migraine headaches [31]. In addition, some pyrazole–lactim derivatives are also considered as important structural motifs of bioactive molecules and have been widely explored in many applications, such as a nervous system drug molecule (**4**) [32], antidiabetic agent (**5**) [33] and immunologically active compound (**6**) [34]. Considering the significance of axially chiral scaffolds and the distinctive biological activities of pyrazolone and pyrazole skeletons mentioned above, we envisaged the development of a novel synthetic strategy to construct axially chiral C–N pyrazole–lactim scaffolds containing a pyrazolone motif.

Since the first report on the C–N axially chiral framework of *N*-phenylpyrrole by Adams in 1931 [35], the construction of novel axially chiral *N*-aryl heterocyclic molecules was reported successively, giving a series of *N*-aryl lactam, pyrrole, indole or imide heterocyclic skeletons [36]. Overall, among the reported synthetic strategies, the desymmetrization reaction, starting from simple and easily available prochiral substrates, was regarded as a valuable and efficient approach, which further constructed multiple chiral centers at the reaction site and the prochiral center at the same time. In this regard, Bencivenni’s group reported the first construction of enantiomerically enriched atropisomeric succinimides via an organocatalytic asymmetric vinylogous Michael addition reaction of *N*-arylmaleimides in 2014 (Scheme 2b) [37]. Subsequently, Bencivenni and co-workers disclosed a novel desymmetrization strategy to construct axially chiral succinimides bearing a C–N axis and contiguous stereocenters by a formal Diels–Alder desymmetrization reaction (Scheme 2b) [38]. In 2021, Biju’s group reported an atroposelective synthesis of C–N axially chiral *N*-aryl succinimides based on the *N*-heterocyclic carbene-catalyzed Stetter-aldol-oxidation cascade process (Scheme 2b) [39]. More recently, following a related strategy, Liao’s group successfully achieved the desymmetrization reaction of prochiral *N*-aryl maleimide by silver-catalyzed asymmetric [3 + 2] cycloaddition (Scheme 2b) [40]. Inspired by the above methods and based on our continuous interest in pyrazole and pyrazolone skeletons, we herein report an enantioselective desymmetrization of a new prochiral *N*-pyrazolyl maleimide through an asymmetric Michael addition reaction with pyrazolones to construct a tri-*N*-heterocyclic pyrazole–succinimide–pyrazolone assembly bearing vicinal quaternary–tertiary stereocenters together with a C–N chiral axis (Scheme 2c).

## 2. Results and Discussion

### 2.1. Optimization of the Reaction Conditions

Initially, pyrazolone **1a** and prochiral *N*-pyrazolyl maleimide **2a** were chosen as the model substrates using DABCO as the catalyst to investigate the feasibility of the reaction. To our delight, the desired axially chiral product **3aa** was smoothly obtained in a 99% yield with 1:1 dr in DCM at 25 °C after 0.5 h (Table 1, entry 1). Encouraged by this result, an asymmetric version of this reaction was carried out by using the series of chiral catalysts **C1**–**C10** derived from cinchona alkaloids to realize atroposelective construction of the optically active product **3aa** (Table 1, entries 2–11). When using the quinine catalyst **C1**, the product **3aa** was obtained in a 91% yield but only with 40% ee (entry 2). We then tested the cinchona alkaloid catalysts **C2** and **C3**, which produced product **3aa** in a 98% yield and 14% ee (entry 3) and 49% ee (entry 4), respectively. Next, we explored the efficacy of various cinchona alkaloid derivatives functionalized with a squaramide or *N*-Boc-protected substituent (entries 5–7). To our delight, the quinine-derived squaramide catalyst **C4** could increase not only the enantioselectivity to 81% ee but also the reactivity of the reaction, furnishing **3aa** in a 99% yield (entry 5). Unfortunately, when the quinine-derived squaramide catalyst **C5** was employed, the product **3aa** was formed in high yield, but no enantioselectivity was obtained in this reaction (entry 6). Moreover, with the quinine-derived *N*-Boc-protected catalyst **C6**, no improvement was observed in terms of the enantioselectivity and the yield of **3aa** dropped to 85% (entry 7). When using sulfonamide **C7** and quaternary ammonium salt **C8**, the target product **3aa** was generated only in moderate yields with poor enantioselectivities (entries 8–9). Subsequently, we used quinine-derived thiourea catalysts **C9** and **C10** to perform this reaction (entries 10–11), and to our surprise, the catalyst **C10** could increase the enantioselectivity of product **3aa** to 91% ee (entry 11). In order to further enhance the enantioselectivity of the reaction, the solvent (entries 12–16) effect was then examined and the results revealed that toluene was optimal with regard to both the yield and enantioselectivity, and 99% yield and 94% ee were observed (entry 12). Finally, the concentration and temperature were investigated (entries 17–19), and the best condition was confirmed with 10 mol% of **C10** in 2 mL of toluene at 25 °C, affording the product **3aa** in a 99% yield with 1:1 dr and 99% ee (entry 17).

### 2.2. Substrate Scope of Pyrazolones **1**

**Scheme 3:**
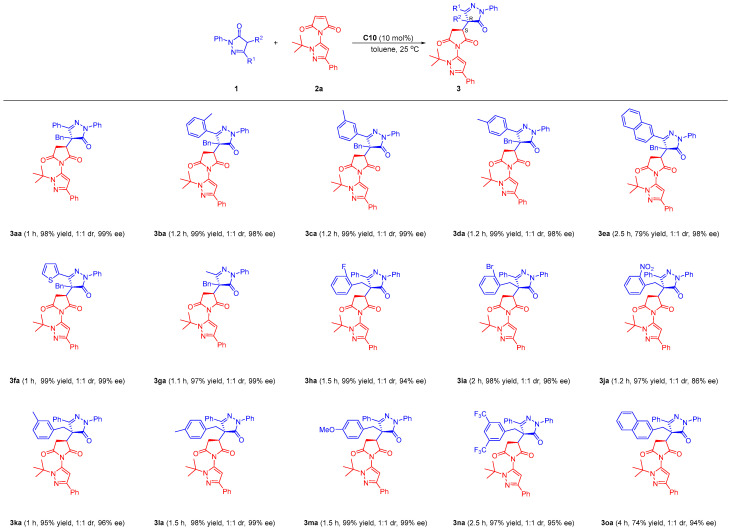
Substrate scope for reactions of pyrazolones **1** with pyrazolyl–maleimide **2a**. The reaction was carried out on a 0.2 mmol scale with **C10** (10 mol%) in toluene (4.0 mL) at 25 °C; the ratio of **1**/**2a** was 1.2/1.0. Isolated yields are given. The dr was determined by ^1^H NMR of crude product. The ee for both diastereomers was determined by chiral HPLC.

With the optimized reaction conditions in hand, we next explored the scope of pyrazolone **1** and the results were shown in Scheme 3. The results showed that these reactants were well tolerated, and most reactions could be accomplished within 4 h to afford the axially chiral product **3** in good yield (up to 99% yield) with excellent enantioselectivity (up to 99% ee). First, pyrazolone substrates bearing different aryl groups (R^1^) at the C-3 position of the pyrazolone unit were examined. When a methyl group was introduced into the *ortho*, *meta* and *para* positions of the phenyl substituents at the pyrazolone unit, the product **3ba**–**3da** could be obtained in a 99% yield with 1:1 dr and high enantioselectivities (98–99% ee). However, when R^1^ was a naphthalene substituent, the yield of the target compound **3ea** was only 79% due to the influence of steric hindrance. In addition, when the substituted phenyl group was replaced by a thiophene substituent, product **3fa** was formed in 99% yield with 1:1 dr and 99% ee. To our delight, methyl proved to be a suitable substituent leading to the target product **3ga** in a 97% yield with 1:1 dr and excellent enantioselectivity (99% ee). To further expand the reaction scope of the pyrazolone unit, additional substituent groups (R^2^) were also explored. A series of substituents on the *ortho*, *meta* and *para* positions of the benzene ring were well tolerated, such as those bearing halides (**3ha**, **3ia**), NO_2_ (**3ja**), methyl (**3ka**, **3la**) and methoxyl (**3ma**) in high yields (95–99%) with 1:1 dr and excellent enantioselectivities (86–99% ee). When the benzene ring was replaced by 3,5-bis(trifluoromethyl)phenyl, the yield and enantioselectivity were maintained, leading to the formation of product **3na** in a 97% yield with 1:1 dr and 95% ee. In addition, the naphthalene-containing substrate **1o** was also tested to afford the corresponding **3oa** in 94% ee, but the yield was reduced to 74%, probably because of the steric hindrance of the bulky substituent.

### 2.3. Substrate Scope of N-Pyrazolyl Maleimides **2**

**Scheme 4:**
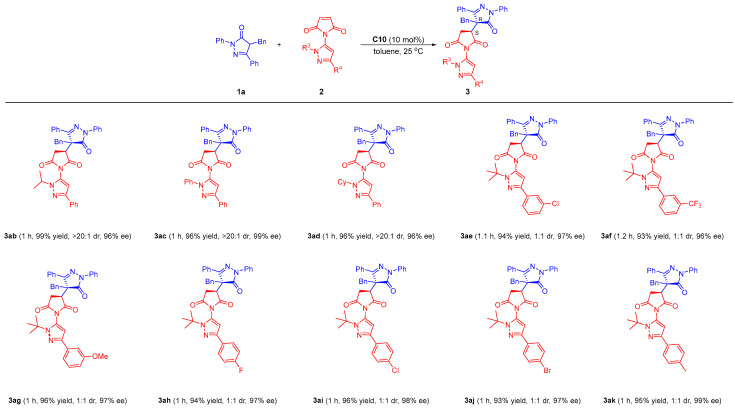
Substrate scope for the reactions of pyrazolone **1a** with N-pyrazolyl maleimides **2**. The reaction was carried out on a 0.2 mmol scale with **C10** (10 mol%) in toluene (4.0 mL) at 25 °C; the ratio of **1a**/**2** was 1.2/1.0. Isolated yields are given. The dr was determined by ^1^H NMR of crude product. The ee was determined by chiral HPLC.

Subsequently, the scope of *N*-pyrazolyl maleimide **2** reacting with pyrazolone **1a** was examined under the optimized reaction conditions. It is noteworthy that good yields and excellent enantioselectivitives were achieved for most cases (Scheme 4). Surprisingly, when the *tert*-butyl group was replaced by isopropyl (**2b**), phenyl (**2c**) and cyclohexyl (**2e**), the corresponding products **3ab**–**3ae** were smoothly afforded in high yields (96–99%) with excellent stereoselectivities (>20:1 dr, >96% ee for all cases). Moreover, benzene rings bearing either electron-withdrawing or electron-donating groups at the pyrazole–maleimide C-3 positions were also examined, and the results disclosed that the fluoro, chloro, bromo, methyl, trifluoromethyl and methoxyl substituent groups were all amenable, producing the corresponding products (**3af**–**3ak**) in high yields (93–96%) with 1:1 dr and excellent enantioselectivities (96–99% ee).

### 2.4. Gram-Scale Reaction and Transformation of Products

**Scheme 5:**
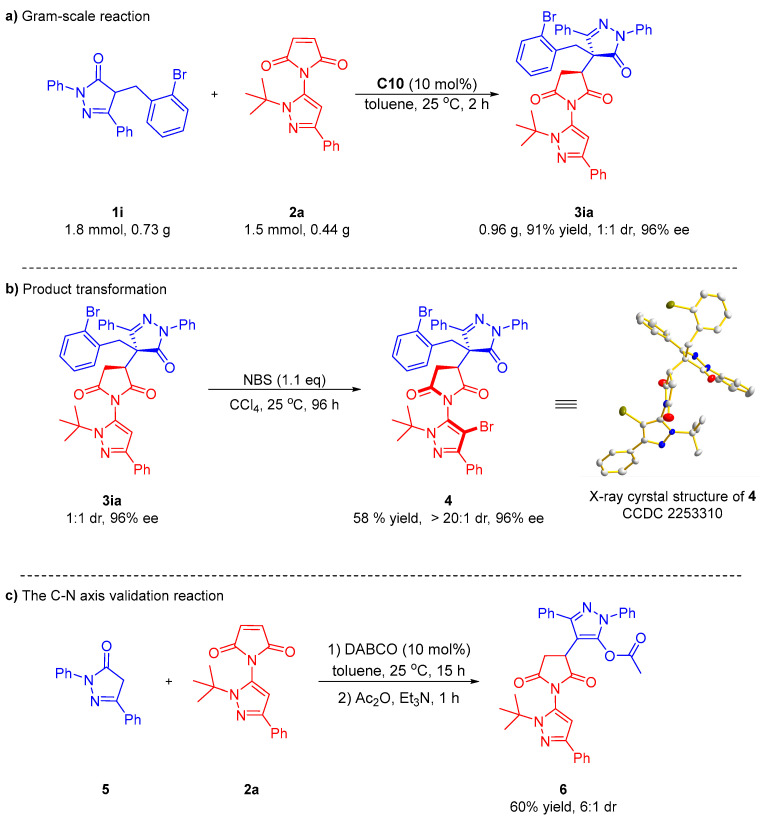
Gram-scale reaction and transformation of products.

To demonstrate the scalability of this protocol, we conducted a gram-scale reaction of pyrazolone **1i** with pyrazolyl–maleimide **2a** under the standard reaction conditions, and the product **3ia** was successfully obtained in a 91% yield with 1:1 dr and 96% ee (Scheme 5a). Subsequently, the selective bromination of compound **3ia** in the presence of NBS proceeded smoothly, producing the product **4** in a 58% yield, >20:1 dr and 96% ee. The excellent diastereoselective results revealed that the bromination at the C-4 position of the pyrazole ring played an important role in controlling the stereoselectivity of the reaction. The *N*-1 and C-4 positions of the pyrazole ring were substituted with tert-butyl and bromine, respectively, and their large steric hindrance effect successfully achieved good stereoselective control of the C–N axis (Scheme 5b). In order to further prove the existence of the C–N axis in the target products, a Michael reaction of 4-nonsubstituted pyrazolone **5** and pyrazolyl–maleimide **2a** was performed under similar reaction conditions, followed by esterification using acetic anhydride to produce the compound **6** in a 60% yield with 6:1 dr. This diastereomeric ratio indicated that the product **6** contained two chiral elements, namely center chirality and axial chirality (Scheme 5c).

### 2.5. Plausible Transition State for the Enantioselective Desymmetrization

**Scheme 6:**
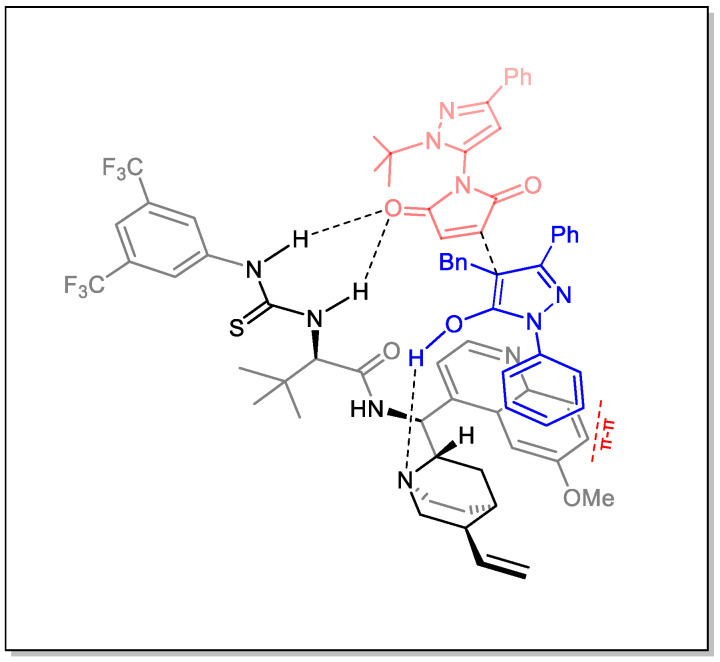
Plausible transition state for the enantioselective desymmetrization.

On the basis of the reaction results and previous similar reports [37,38,41,42], a plausible reaction transition state for the desymmetrization was proposed in Scheme 6. The transition state was made up of a ternary complex in which catalyst **C10** promoted the formation of a reactive enolate and at the same time anchored the maleimide by means of hydrogen bonds with the thiourea functional group. In addition, there may have existed a π–π interaction between the phenyl of pyrazolone and the naphthalene ring of catalyst **C10** that played an important role in the control of the enantioselectivity of the target product. Subsequently, succinimides with two adjacent stereocenters were generated via an asymmetric Michael reaction, which further obtained the C–N axially chiral pyrazolyl–succinimide **3**.

## 3. Materials and Methods

### 3.1. General Information

Unless otherwise noted, the materials were purchased from commercial suppliers and used without further purification. Column chromatography was performed on silica gel (200~300 mesh). Enantiomeric excesses (ee) were determined by HPLC (Agilent, Palo Alto, CA, USA) using the corresponding commercial chiral columns as stated at 25 °C with a UV detector at 254 nm. Optical rotations (JiaHang Instruments, Shanghai, China) were reported as follows: ${\left[\alpha \right]}_{D}^{T}$ (*c* g/100 mL, solvent). All ^1^H NMR and ^19^F NMR spectra were recorded on a Bruker Avance II 400 MHz (Bruker, Karlsruhe, Germany) and Bruker Avance III 600 MHz (Bruker, Karlsruhe, Germany), respectively; (Supplementary Materials) ^13^C NMR spectra were recorded on a Bruker Avance II 101 MHz or Bruker Avance III 151 MHz with chemical shifts reported as ppm (in CDCl_3_, TMS as an internal standard). Data for ^1^H NMR were recorded as follows: chemical shift (δ, ppm), multiplicity (s = singlet, d = doublet, t = triplet, m = multiplet, br = broad singlet, dd = double doublet, coupling constants in Hz and integration). HRMS (ESI) was obtained with an HRMS/MS instrument (LTQ Orbitrap XL TM, Agilent, Palo Alto, CA, USA). The absolute configuration of **4** was assigned by the X-ray analysis.

### 3.2. Procedure for the Synthesis of Compounds **2**

The compound 5-Aminopyrazole was prepared according to the literature [43]. The maleic anhydride (7.5 mmol) and 5-aminopyrazole (5 mmol) were dissolved in 10 mL CHCl_3_, stirred for 10 h and the solid (maleimide acid) precipitated from the reaction mixture was filtered. Maleimide acid was dissolved in 20 mL acetic anhydride and 200 mg sodium acetate was added. The mixture was heated at 85 °C and stirred for 4 h. The reaction was cooled and quenched with water, then the mixture was filtered, quenched with water and extracted with ethyl acetate. The organic phase was separated, washed with water and dried over Na_2_SO_4_. The product was purified by silica gel column chromatography with a mixture of petroleum ether and ethyl acetate (10:1) as eluent. The target compound **2** (0.96 g, 65 %) was obtained as a solid.

*1-(1-(tert-butyl)-3-phenyl-1H-pyrazol-5-yl)-1H-pyrrole-2,5-dione* (**2a**)

This compound was prepared according to the procedure within 15 h as a white solid (960 mg, 65% yield, Petroleum ether/EtOAc = 10/1 as the eluent), mp 154.1–154.9 °C. ^1^H NMR (600 MHz, Chloroform-d) δ: 7.80–7.75 (m, 2H), 7.38 (dd, *J* = 7.5 Hz, 2H), 7.31–7.27 (m, 1H), 6.92 (d, *J* = 3.4 Hz, 2H), 6.47 (s, 1H), 1.59 (s, 9H). ^13^C NMR (101 MHz, CDCl_3_) δ: 169.10, 148.85, 135.29, 133.38, 128.54, 127.99, 127.71, 125.45, 104.50, 61.24, 29.90. HRMS (*m*/*z*): Calcd for C_17_H_18_N_3_O_2_, [M + H]^+^, 296.1394, found: 296.1392.

*1-(1-isopropyl-3-phenyl-1H-pyrazol-5-yl)-1H-pyrrole-2,5-dione* (**2b**)

This compound was prepared according to the procedure within 15 h as a white solid (300 mg, 60% yield, Petroleum ether/EtOAc = 10/1 as the eluent), mp 115.1–115.9 °C. ^1^H NMR (400 MHz, Chloroform-d) δ: 7.84–7.76 (m, 2H), 7.38 (dd, *J* = 7.5 Hz, 2H), 7.33–7.26 (m, 1H), 6.90 (d, *J* = 1.8 Hz, 2H), 6.48 (s, 1H), 4.14 (h, *J* = 6.6 Hz, 1H), 1.50 (d, *J* = 6.6 Hz, 6H). ^13^C NMR (101 MHz, CDCl_3_) δ: 168.48, 150.58, 135.04, 133.47, 128.57, 127.78, 125.56, 101.61, 50.35, 22.63. HRMS (*m*/*z*): Calcd for C_16_H_16_N_3_O_2_, [M + H]^+^, 282.1237, found: 282.1240.

*1-(1,3-diphenyl-1H-pyrazol-5-yl)-1H-pyrrole-2,5-dione* (**2c**)

This was prepared according to the procedure within 15 h as a white solid (200 mg, 40% yield, Petroleum ether/EtOAc = 10/1 as the eluent), mp 108.1–108.9 °C. ^1^H NMR (400 MHz, Chloroform-d) δ: 7.90–7.83 (m, 2H), 7.46–7.34 (m, 8H), 6.81 (s, 2H), 6.76 (s, 1H). ^13^C NMR (101 MHz, CDCl_3_) δ: 168.11, 152.02, 138.22, 134.94, 132.60, 129.54, 129.38, 128.69, 128.65, 128.36, 125.76, 124.51, 104.10. HRMS (*m*/*z*): Calcd for C_19_H_14_N_3_O_2_, [M + H]^+^, 316.1081, found: 316.1080.

*1-(1-cyclohexyl-3-phenyl-1H-pyrazol-5-yl)-1H-pyrrole-2,5-dione* (**2d**)

This was prepared according to the procedure within 15 h as a white solid (400 mg, 50% yield, Petroleum ether/EtOAc = 10/1 as the eluent), mp 105.1–105.9 °C. ^1^H NMR (400 MHz, Chloroform-d) δ: 7.79 (d, *J* = 7.6 Hz, 2H), 7.38 (t, *J* = 7.5 Hz, 2H), 7.30 (d, *J* = 7.3 Hz, 1H), 6.93 (s, 2H), 6.47 (s, 1H), 3.67 (tt, *J* = 10.9, 4.5 Hz, 1H), 2.16–1.79 (m, 7H), 1.74–1.62 (m, 1H), 1.31 (d, *J* = 11.7 Hz, 3H). ^13^C NMR (101 MHz, CDCl_3_) δ: 168.51, 150.45, 135.05, 133.48, 128.54, 127.97, 127.72, 125.57, 101.51, 58.04, 32.91, 25.65, 25.12. HRMS (*m*/*z*): Calcd for C_19_H_20_N_3_O_2_, [M + H]^+^, 322.1550, found: 322.1551.

*1-(1-(tert-butyl)-3-(3-chlorophenyl)-1H-pyrazol-5-yl)-1H-pyrrole-2,5-dione* (**2e**)

This was prepared according to the procedure within 15 h as an orange solid (600 mg, 52% yield, Petroleum ether/EtOAc = 10/1 as the eluent), mp 140.1–140.9 °C. ^1^H NMR (400 MHz, Chloroform-d) δ: 7.79 (s, 1H), 7.63 (dt, *J* = 7.5, 1.5 Hz, 1H), 7.33–7.25 (m, 2H), 6.94 (d, *J* = 0.9 Hz, 2H), 6.46 (s, 1H), 1.58 (s, 9H). ^13^C NMR (101 MHz, CDCl_3_) δ: 168.99, 147.50, 135.32, 135.17, 134.51, 129.81, 128.25, 127.66, 125.46, 123.55, 104.70, 61.52, 29.87. HRMS (*m*/*z*): Calcd for C_17_H_17_ClN_3_O_2_, [M + H]^+^, 330.1004, found: 330.0999.

*1-(1-(tert-butyl)-3-(3-(trifluoromethyl)phenyl)-1H-pyrazol-5-yl)-1H-pyrrole-2,5-dione* (**2f**)

This was prepared according to the procedure within 15 h as a white solid (400 mg, 53% yield, Petroleum ether/EtOAc = 10/1 as the eluent), mp 146.1–146.9 °C. ^1^H NMR (400 MHz, Chloroform-d) δ: 8.03 (d, *J* = 2.2 Hz, 1H), 7.97–7.93 (m, 1H), 7.56–7.47 (m, 2H), 6.95 (s, 2H), 6.52 (s, 1H), 1.60 (s, 9H). ^13^C NMR (101 MHz, CDCl_3_) δ: 168.98, 147.46, 135.34, 134.15, 131.08, 130.76, 128.99, 128.57, 128.41, 125.60, 124.25 (d, *J* = 4.04 Hz), 122.89, 122.21 (d, *J* = 4.04 Hz), 104.73, 61.62, 29.87. ^19^F NMR (565 MHz, CDCl_3_) δ: −62.66. HRMS (*m*/*z*): Calcd for C_18_H_17_F_3_N_3_O_2_, [M + H]^+^, 364.1267, found: 364.1267.

*1-(1-(tert-butyl)-3-(3-methoxyphenyl)-1H-pyrazol-5-yl)-1H-pyrrole-2,5-dione* (**2g**)

This was prepared according to the procedure within 15 h as a white solid (300 mg, 50% yield, Petroleum ether/EtOAc = 10/1 as the eluent), mp 103.1–103.9 °C. ^1^H NMR (400 MHz, Chloroform-d) δ: 7.35 (s, 2H), 7.31 (s, 1H), 6.91 (s, 2H), 6.84 (s, 1H), 6.45 (s, 1H), 3.85 (s, 3H), 1.58 (s, 9H). ^13^C NMR (101 MHz, CDCl_3_) δ: 169.10, 159.84, 135.29, 134.77, 129.58, 127.99, 118.11, 113.44, 110.83, 104.69, 61.28, 55.65, 29.89. HRMS (*m*/*z*): Calcd for C_18_H_20_N_3_O_3_, [M + H]^+^, 326.1499, found: 326.1498.

*1-(1-(tert-butyl)-3-(4-fluorophenyl)-1H-pyrazol-5-yl)-1H-pyrrole-2,5-dione* (**2h**)

This was prepared according to the procedure within 15 h as a white solid (500 mg, 55% yield, Petroleum ether/EtOAc = 10/1 as the eluent), mp 166.1–166.9 °C. ^1^H NMR (400 MHz, Chloroform-d) δ: 7.77–7.72 (m, 2H), 7.09–7.04 (m, 2H), 6.94 (s, 2H), 6.42 (s, 1H), 1.58 (s, 9H). ^13^C NMR (101 MHz, CDCl_3_) δ: 169.06, 162.6 (d, *J* = 246.4 Hz), 148.01, 135.31, 129.63, 129.60, 128.10, 127.15, 127.07, 115.53, 115.32, 104.27, 61.29, 29.88. ^19^F NMR (376 MHz, CDCl_3_) δ: −114.71. HRMS (*m*/*z*): Calcd for C_17_H_17_FN_3_O_2_, [M + H]^+^, 314.1299, found: 314.1298.

*1-(1-(tert-butyl)-3-(4-chlorophenyl)-1H-pyrazol-5-yl)-1H-pyrrole-2,5-dione* (**2i**)

This was prepared according to the procedure within 15 h as an orange solid (500 mg, 55% yield, Petroleum ether/EtOAc = 10/1 as the eluent), mp 155.1–155.9 °C. ^1^H NMR (400 MHz, Chloroform-d) δ: 7.72 (s, 2H), 7.35 (s, 2H), 6.93 (s, 2H), 6.44 (s, 1H), 1.58 (s, 9H). ^13^C NMR (101 MHz, CDCl_3_) δ: 169.04, 147.76, 135.32, 133.39, 131.91, 128.71, 128.20, 126.70, 104.48, 61.43, 29.87. HRMS (*m*/*z*): Calcd for C_17_H_17_ClN_3_O_2_, [M + H]^+^, 330.1004, found: 330.1003.

*1-(3-(4-bromophenyl)-1-(tert-butyl)-1H-pyrazol-5-yl)-1H-pyrrole-2,5-dione* (**2j**)

This was prepared according to the procedure within 15 h as a white solid (400 mg, 58% yield, Petroleum ether/EtOAc = 10/1 as the eluent), mp 164.1–164.9 °C. ^1^H NMR (400 MHz, Chloroform-d) δ: 7.67 (d, *J* = 8.6 Hz, 2H), 7.52 (d, *J* = 8.6 Hz, 2H), 6.97 (s, 2H), 6.47 (s, 1H), 1.60 (s, 9H). ^13^C NMR (101 MHz, CDCl_3_) δ: 169.02, 147.77, 135.32, 132.35, 131.64, 128.20, 127.00, 121.59, 104.47, 61.45, 29.87. HRMS (*m*/*z*): Calcd for C_17_H_17_BrN_3_O_2_, [M + H]^+^, 374.0499, found: 374.0496.

*1-(1-(tert-butyl)-3-(p-tolyl)-1H-pyrazol-5-yl)-1H-pyrrole-2,5-dione* (**2k**)

This was prepared according to the procedure within 15 h as a white solid (400 mg, 48% yield, Petroleum ether/EtOAc = 10/1 as the eluent), mp 176.1–176.9 °C. ^1^H NMR (400 MHz, Chloroform-d) δ: 7.65 (s, 2H), 7.17 (s, 2H), 6.91 (s, 2H), 6.42 (s, 1H), 2.36 (s, 3H), 1.55 (s, 9H). ^13^C NMR (101 MHz, CDCl_3_) δ: 169.14, 148.95, 137.38, 135.28, 130.63, 129.22, 127.85, 125.36, 104.27, 61.12, 29.90. HRMS (*m*/*z*): Calcd for C_18_H_20_N_3_O_2_, [M + H]^+^, 310.1550, found: 310.1551.

### 3.3. Procedure for the Synthesis of Compounds **3**

In a reaction tube, pyrazol-5-ones **1** (0.24 mmol), pyrazole-maleimide **2** (0.20 mmol) and catalyst **C10** (0.02 mmol) were added into toluene (4 mL). The reaction solution was stirred at 25 °C. After the reaction was complete (monitored by TLC), the crude product was purified by column chromatography (ethyl acetate/petroleum ether = 1/10 to 1/3) on silica gel to produce the product **3**.

*(S)-3-((R)-4-benzyl-5-oxo-1,3-diphenyl-4,5-dihydro-1H-pyrazol-4-yl)-1-(1-(tert-butyl)-3-phenyl-1H-pyrazol-5-yl)pyrrolidine-2,5-dione* (**3aa**)

This was prepared according to the procedure within 1 h as a white solid (121.8 mg, 98% yield, dr = 1:1). mp 127.1–127.9 °C; ${\left[\alpha \right]}_{D}^{17}$ = −33.206 (*c* 0.52, CH_2_Cl_2_); ^1^H NMR (400 MHz, Chloroform-d) δ: 8.00 (dq, *J* = 6.7, 2.6, 1.6 Hz, 4H), 7.69–7.48 (m, 15H), 7.40–7.28 (m, 9H), 7.23–7.16 (m, 2H), 7.12–7.01 (m, 10H), 6.29 (s, 1H), 5.50 (s, 1H), 4.22 (dd, *J* = 17.6, 7.4 Hz, 2H), 3.99 (dd, *J* = 9.3, 7.4 Hz, 1H), 3.76 (dd, *J* = 9.4, 5.5 Hz, 1H), 3.61 (dd, *J* = 19.5, 13.5 Hz, 2H), 3.50 (d, *J* = 13.2 Hz, 1H), 3.18 (dd, *J* = 17.8, 9.4 Hz, 1H), 3.01 (dd, *J* = 18.6, 9.7 Hz, 1H), 2.78 (s, 1H), 1.53 (s, 9H), 1.38 (s, 9H). ^13^C NMR (151 MHz, CDCl_3_) δ: 174.89, 173.87, 173.72, 173.43, 173.07, 158.07, 157.37, 148.93, 148.66, 137.00, 136.82, 133.31, 133.24, 132.50, 131.10, 131.02, 130.99, 130.71, 129.42, 129.36, 129.35, 129.17, 128.99, 128.79, 128.71, 128.49, 128.47, 128.44, 128.39, 127.95, 127.79, 127.69, 127.61, 127.00, 126.29, 126.05, 125.40, 125.34, 120.04, 119.89, 103.68, 61.50, 57.13, 44.93, 43.58, 41.24, 40.02, 31.06, 30.04, 29.81, 29.69. HRMS (ESI) *m*/*z* Calcd for C_39_H_36_N_5_O_3_, [M + H]^+^, 622.2813, found: 622.2806. Enantiomeric excess was determined to be 99% (determined by HPLC using chiral IB-H column, hexane/2-propanol = 7/3, λ = 254 nm, 25 °C, 0.8 mL/min, t_major_ = 21.9 min, t_minor_ = 16.6 min).

*(S)-3-((R)-4-benzyl-5-oxo-1-phenyl-3-(o-tolyl)-4,5-dihydro-1H-pyrazol-4-yl)-1-(1-(tert-butyl)-3-phenyl-1H-pyrazol-5-yl)pyrrolidine-2,5-dione* (**3ba**)

This was prepared according to the procedure within 1.2 h as a white solid (125.8 mg, 99% yield, dr = 1:1), mp 108.1–108.9 °C; ${\left[\alpha \right]}_{D}^{17}$ = −35.030 (*c* 0.33, CH_2_Cl_2_). ^1^H NMR (400 MHz, Chloroform-d) δ: 7.83 (d, *J* = 11.5 Hz, 2H), 7.77 (q, *J* = 3.5 Hz, 2H), 7.69–7.61 (m, 6H), 7.58 (d, *J* = 8.0 Hz, 2H), 7.46–7.41 (m, 2H), 7.41–7.26 (m, 12H), 7.22–7.15 (m, 2H), 7.08 (d, *J* = 6.9 Hz, 8H), 7.03 (dd, *J* = 7.8, 1.8 Hz, 2H), 6.29 (s, 1H), 5.51 (s, 1H), 4.31–4.13 (m, 2H), 3.98 (dd, *J* = 9.3, 7.4 Hz, 1H), 3.75 (dd, *J* = 9.4, 5.4 Hz, 1H), 3.60 (dd, *J* = 26.7, 13.5 Hz, 2H), 3.48 (d, *J* = 13.2 Hz, 1H), 3.18 (dd, *J* = 17.8, 9.4 Hz, 1H), 2.99 (dd, *J* = 18.6, 9.7 Hz, 1H), 2.66 (d, *J* = 58.4 Hz, 1H), 2.43 (s, 6H), 1.38 (s, 9H). ^13^C NMR (101 MHz, CDCl_3_) δ: 173.89, 173.75, 173.46, 173.08, 158.23, 157.50, 148.90, 148.64, 139.37, 139.09, 137.00, 136.83, 133.39, 133.33, 133.27, 132.55, 131.90, 131.63, 130.98, 130.89, 129.39, 129.26, 128.97, 128.88, 128.81, 128.70, 128.50, 128.46, 128.41, 128.37, 128.17, 127.93, 127.75, 127.68, 127.60, 126.27, 126.03, 125.39, 125.26, 124.66, 123.88, 120.09, 119.94, 103.70, 103.63, 61.50, 57.14, 44.95, 43.55, 41.28, 40.04, 31.10, 30.07, 29.81, 29.70, 21.68, 21.65. HRMS (ESI) *m*/*z* Calcd for C_40_H_38_N_5_O_3_, [M + H]^+^, 636.2969, found: 636.2972. Enantiomeric excess was determined to be 98% (determined by HPLC using chiral OD-H column, hexane/2-propanol = 7/3, λ = 254 nm, 25 °C, 0.8 mL/min, t_major_ = 25.1 min, t_minor_ = 12.1 min).

*(S)-3-((R)-4-benzyl-5-oxo-1-phenyl-3-(m-tolyl)-4,5-dihydro-1H-pyrazol-4-yl)-1-(1-(tert-butyl)-3-phenyl-1H-pyrazol-5-yl)pyrrolidine-2,5-dione* (**3ca**)

This was prepared according to the procedure within 1.2 h as a white solid (125.8 mg, 99% yield, dr = 1:1), mp 109.1–109.9 °C; ${\left[\alpha \right]}_{D}^{17}$ = −33.491 (*c* 0.42, CH_2_Cl_2_). ^1^H NMR (400 MHz, Chloroform-d) δ: 7.85 (s, 1H), 7.82 (s, 1H), 7.78 (d, *J* = 7.2 Hz, 2H), 7.68 (d, *J* = 1.5 Hz, 1H), 7.67–7.61 (m, 5H), 7.60–7.55 (m, 2H), 7.43 (dd, *J* = 12.2, 6.9 Hz, 3H), 7.40–7.27 (m, 11H), 7.23–7.16 (m, 2H), 7.09 (d, *J* = 5.6 Hz, 8H), 7.04 (dd, *J* = 7.7, 1.9 Hz, 2H), 6.29 (d, *J* = 1.0 Hz, 1H), 5.50 (s, 1H), 4.31–4.15 (m, 2H), 3.99 (dd, *J* = 9.3, 7.5 Hz, 1H), 3.75 (dd, *J* = 9.5, 5.4 Hz, 1H), 3.65 (d, *J* = 13.2 Hz, 1H), 3.57 (d, *J* = 13.8 Hz, 1H), 3.49 (d, *J* = 13.2 Hz, 1H), 3.19 (dd, *J* = 17.8, 9.4 Hz, 1H), 3.00 (dd, *J* = 18.6, 9.7 Hz, 1H), 2.66 (d, *J* = 49.9 Hz, 1H), 2.44 (s, 6H), 1.54 (s, 9H), 1.38 (d, *J* = 1.0 Hz, 9H). ^13^C NMR (101 MHz, CDCl_3_) δ: 174.94, 173.90, 173.76, 173.46, 173.11, 173.08, 158.23, 157.51, 148.90, 148.65, 139.37, 139.09, 137.00, 136.82, 133.39, 133.33, 133.26, 132.55, 131.90, 131.63, 130.98, 130.89, 129.39, 129.26, 128.97, 128.88, 128.81, 128.70, 128.50, 128.47, 128.41, 128.37, 128.17, 127.93, 127.75, 127.69, 127.61, 126.28, 126.04, 125.40, 125.27, 124.66, 123.88, 120.09, 119.94, 103.70, 103.63, 61.50, 57.14, 44.95, 43.55, 41.28, 40.04, 31.10, 30.07, 29.81, 29.70, 21.65. HRMS (ESI) *m*/*z* Calcd for C_40_H_38_N_5_O_3_, [M + H]^+^, 636.2969, found: 636.2974. Enantiomeric excess was determined to be 99% (determined by HPLC using chiral OD-H column, hexane/2-propanol = 7/3, λ = 254 nm, 25 °C, 0.8 mL/min, t_major_ = 22.5 min, t_minor_ = 12.1 min).

*(S)-3-((R)-4-benzyl-5-oxo-1-phenyl-3-(p-tolyl)-4,5-dihydro-1H-pyrazol-4-yl)-1-(1-(tert-butyl)-3-phenyl-1H-pyrazol-5-yl)pyrrolidine-2,5-dione* (**3da**)

This was prepared according to the procedure within 1.2 h as a white solid (125.8 mg, 99% yield, dr = 1:1), mp 127.1–127.9 °C; ${\left[\alpha \right]}_{D}^{17}$ = −61.572 (*c* 0.23, CH_2_Cl_2_). ^1^H NMR (400 MHz, Chloroform-d) δ: 7.89 (dd, *J* = 8.0 Hz, 4H), 7.68–7.56 (m, 9H), 7.39–7.28 (m, 14H), 7.19 (dd, *J* = 16.7, 8.2 Hz, 2H), 7.09 (d, *J* = 6.7 Hz, 8H), 7.06–7.01 (m, 2H), 6.28 (s, 1H), 5.48 (s, 1H), 4.20 (d, *J* = 26.5 Hz, 2H), 3.97 (dd, *J* = 9.3, 7.4 Hz, 1H), 3.74 (dd, *J* = 9.7, 5.2 Hz, 1H), 3.61 (dd, *J* = 27.2, 13.5 Hz, 2H), 3.49 (d, *J* = 13.2 Hz, 1H), 3.17 (dd, *J* = 17.8, 9.4 Hz, 1H), 2.99 (dd, *J* = 18.6, 9.7 Hz, 1H), 2.63 (d, *J* = 27.3 Hz, 1H), 2.50 (s, 3H), 2.44 (s, 3H), 1.38 (s, 9H). ^13^C NMR (101 MHz, CDCl_3_) δ: 174.98, 173.84, 173.76, 173.38, 173.08, 173.01, 158.07, 157.37, 148.92, 148.63, 141.58, 141.11, 137.03, 136.86, 133.46, 133.33, 133.26, 132.59, 130.09, 129.89, 129.37, 128.95, 128.80, 128.67, 128.48, 128.45, 128.40, 128.35, 128.32, 128.20, 127.89, 127.71, 127.58, 126.90, 126.21, 125.96, 125.38, 125.36, 120.04, 119.87, 103.68, 103.66, 61.51, 61.48, 57.15, 44.97, 43.50, 41.21, 39.88, 31.12, 30.07, 29.80, 29.67, 21.62, 21.55. HRMS (ESI) *m*/*z* Calcd for C_40_H_38_N_5_O_3_, [M + H]^+^, 636.2969, found: 636.2979. Enantiomeric excess was determined to be 98% (determined by HPLC using chiral OD-H column, hexane/2-propanol = 7/3, λ = 254 nm, 25 °C, 0.8 mL/min, t_major_ = 25.3 min, t_minor_ = 12.1 min).

*(S)-3-((R)-4-benzyl-3-(naphthalen-2-yl)-5-oxo-1-phenyl-4,5-dihydro-1H-pyrazol-4-yl)-1-(1-(tert-butyl)-3-phenyl-1H-pyrazol-5-yl)pyrrolidine-2,5-dione* (**3ea**)

This was prepared according to the procedure within 2.5 h as a white solid (106.1 mg, 79% yield, dr = 1:1), mp 110.1–110.9 °C; ${\left[\alpha \right]}_{D}^{17}$ = −46.491 (*c* 0.79, CH_2_Cl_2_). ^1^H NMR (400 MHz, Chloroform-d) δ: 8.42 (d, *J* = 2.3 Hz, 1H), 8.39 (s, 1H), 8.20–8.12 (m, 3H), 8.00–7.92 (m, 7H), 7.91–7.85 (m, 1H), 7.72–7.61 (m, 9H), 7.61–7.51 (m, 4H), 7.37 (dt, *J* = 15.5, 7.6 Hz, 5H), 7.32–7.17 (m, 15H), 7.15–7.01 (m, 13H), 6.26 (d, *J* = 2.0 Hz, 1H), 4.95 (s, 1H), 4.47 (s, 1H), 4.20 (dd, *J* = 10.1, 5.1 Hz, 1H), 4.11 (t, *J* = 8.2 Hz, 1H), 3.81 (q, *J* = 6.2, 5.6 Hz, 2H), 3.66 (dd, *J* = 13.9, 1.6 Hz, 1H), 3.56 (d, *J* = 13.2 Hz, 1H), 3.22 (dd, *J* = 17.6, 9.1 Hz, 1H), 3.00 (dd, *J* = 18.7, 9.8 Hz, 1H), 2.47 (s, 1H), 1.52 (d, *J* = 1.2 Hz, 9H), 1.35 (d, *J* = 2.1 Hz, 9H). ^13^C NMR (151 MHz, CDCl3) δ: 175.41, 173.92, 173.74, 173.51, 173.14, 173.03, 157.91, 157.12, 148.68, 137.00, 136.82, 134.37, 134.28, 133.50, 133.29, 132.95, 132.92, 132.85, 132.56, 129.43, 129.39, 129.29, 129.23, 129.04, 128.77, 128.71, 128.46, 128.43, 128.40, 128.23, 128.15, 128.04, 128.00, 127.97, 127.76, 127.67, 127.60, 127.48, 127.05, 126.56, 126.40, 126.16, 125.38, 125.28, 124.13, 124.04, 120.13, 119.97, 103.66, 103.58, 61.49, 57.31, 45.19, 43.54, 41.45, 40.11, 31.21, 30.12, 29.82, 29.65. HRMS (ESI) *m*/*z* Calcd for C_43_H_38_N_5_O_3_, [M + H]^+^, 672.2969, found: 672.2976. Enantiomeric excess was determined to be 98% (determined by HPLC using chiral OD-H-AD-H column, hexane/2-propanol = 7/3, λ = 254 nm, 25 °C, 0.6 mL/min, t_major_ = 61.0 min, t_minor_ = 34.0 min).

*(S)-3-((R)-4-benzyl-5-oxo-1-phenyl-3-(thiophen-2-yl)-4,5-dihydro-1H-pyrazol-4-yl)-1-(1-(tert-butyl)-3-phenyl-1H-pyrazol-5-yl)pyrrolidine-2,5-dione* (**3fa**)

This was prepared according to the procedure within 1.1 h as a white solid (124.2 mg, 99% yield, dr = 1:1), mp 120.1–120.9 °C; ${\left[\alpha \right]}_{D}^{17}$ = −35.474 (*c* 0.65, CH_2_Cl_2_). ^1^H NMR (400 MHz, Chloroform-d) δ: 7.64 (ddd, *J* = 19.8, 10.9, 7.0 Hz, 9H), 7.53 (dd, *J* = 10.3, 6.5 Hz, 3H), 7.39–7.28 (m, 9H), 7.25–7.17 (m, 4H), 7.16–7.00 (m, 11H), 6.33 (s, 1H), 5.80 (s, 1H), 4.09 (d, *J* = 28.1 Hz, 2H), 4.00–3.94 (m, 1H), 3.77 (dd, *J* = 9.4, 5.6 Hz, 1H), 3.59–3.51 (m, 2H), 3.47 (d, *J* = 13.1 Hz, 1H), 3.19 (dd, *J* = 17.8, 9.3 Hz, 1H), 3.03 (dd, *J* = 18.5, 9.6 Hz, 1H), 2.79 (s, 1H), 1.57 (s, 9H), 1.38 (s, 9H). ^13^C NMR (101 MHz, CDCl_3_) δ: 173.69, 173.56, 172.98, 172.69, 172.39, 154.24, 149.01, 148.68, 136.79, 136.61, 134.42, 134.31, 133.30, 133.21, 132.36, 129.63, 129.46, 129.40, 128.96, 128.92, 128.70, 128.50, 128.46, 128.44, 128.24, 127.97, 127.91, 127.78, 127.72, 127.61, 127.39, 126.33, 126.10, 125.39, 125.35, 120.08, 119.96, 103.67, 61.59, 61.51, 45.35, 41.07, 39.72, 31.05, 30.11, 29.83, 29.65. HRMS (ESI) *m*/*z* Calcd for C_37_H_34_N_5_O_3_S, [M + H]^+^, 628.2377, found: 628.2387. Enantiomeric excess was determined to be 99% (determined by HPLC using chiral OD-H column, hexane/2-propanol = 8/2, λ = 254 nm, 25 °C, 0.8 mL/min, t_major_ = 32.4 min, t_minor_ = 21.3 min).

*(S)-3-((R)-4-benzyl-3-methyl-5-oxo-1-phenyl-4,5-dihydro-1H-pyrazol-4-yl)-1-(1-(tert-butyl)-3-phenyl-1H-pyrazol-5-yl)pyrrolidine-2,5-dione* (**3ga**)

This was prepared according to the procedure within 1.1 h as a white solid (108.5mg, 97% yield, dr = 1:1), mp 124.1–124.9 °C; ${\left[\alpha \right]}_{D}^{17}$ = 80.357 (*c* 0.45, CH_2_Cl_2_). ^1^H NMR (400 MHz, Chloroform-d) δ: 7.75–7.70 (m, 2H), 7.66–7.61 (m, 2H), 7.61–7.56 (m, 2H), 7.53–7.48 (m, 2H), 7.38–7.26 (m, 10H), 7.21–7.08 (m, 12H), 6.38 (s, 1H), 6.18 (s, 1H), 4.17 (dd, *J* = 18.1, 6.8 Hz, 1H), 3.56–3.35 (m, 4H), 3.28 (t, *J* = 14.2 Hz, 2H), 3.13–3.03 (m, 2H), 3.02–2.96 (m, 1H), 2.28 (s, 3H), 2.26 (s, 3H), 1.58 (s, 7H), 1.38 (s, 9H). ^13^C NMR (101 MHz, CDCl_3_) δ: 174.51, 174.00, 173.76, 173.29, 172.63, 172.49, 160.48, 159.29, 149.12, 148.67, 137.04, 136.92, 133.30, 133.14, 133.09, 132.60, 129.20, 129.13, 128.93, 128.91, 128.85, 128.74, 128.70, 128.66, 128.61, 128.53, 128.47, 128.02, 127.92, 127.75, 127.69, 127.25, 125.91, 125.70, 125.43, 125.41, 125.12, 119.63, 119.07, 103.61, 103.51, 61.59, 61.52, 59.86, 57.34, 53.71, 44.04, 43.24, 40.46, 39.44, 33.77, 30.78, 29.87, 29.68, 29.61, 15.32, 14.71. HRMS (ESI) *m*/*z* Calcd for C_34_H_34_N_5_O_3_, [M + H]^+^, 560.2656, found: 560.2659. Enantiomeric excess was determined to be 99% (determined by HPLC using chiral IA-H-OD-H column, hexane/2-propanol = 8/2, λ = 254 nm, 25 °C, 0.6 mL/min, t_major_ = 113.3 min, t_minor_ = 71.1 min).

*(S)-1-(1-(tert-butyl)-3-phenyl-1H-pyrazol-5-yl)-3-((R)-4-(2-fluorobenzyl)-5-oxo-1,3-diphenyl-4,5-dihydro-1H-pyrazol-4-yl)pyrrolidine-2,5-dione* (**3ha**)

This was prepared according to the procedure within 1.5 h as a white solid (126.6 mg, 99% yield, dr = 1:1), mp 113.1–113.9 °C; ${\left[\alpha \right]}_{D}^{17}$ = −42.005 (*c* 0.89, CH_2_Cl_2_). ^1^H NMR (400 MHz, Chloroform-d) δ: 7.99–7.93 (m, 4H), 7.76–7.71 (m, 2H), 7.67 (ddd, *J* = 8.1, 3.3, 1.2 Hz, 4H), 7.64–7.55 (m, 4H), 7.55–7.44 (m, 5H), 7.42–7.28 (m, 9H), 7.23–7.03 (m, 6H), 6.92–6.81 (m, 4H), 6.28 (s, 1H), 5.54 (s, 1H), 4.36–4.14 (m, 2H), 4.02 (dd, *J* = 9.3, 7.4 Hz, 1H), 3.81–3.71 (m, 2H), 3.66 (d, *J* = 14.2 Hz, 1H), 3.52 (d, *J* = 13.7 Hz, 1H), 3.18 (dd, *J* = 17.8, 9.4 Hz, 1H), 2.99 (dd, *J* = 18.5, 9.7 Hz, 1H), 2.82 (s, 1H), 1.52 (s, 9H), 1.38 (s, 9H). ^13^C NMR (151 MHz, CDCl_3_) δ: 174.85, 173.83, 173.72, 173.42, 173.01, 172.89, 161.74 (d, *J* = 8.08 Hz), 160.14, 160.06, 158.39, 157.77, 148.94, 148.63, 137.06, 136.88, 133.29, 133.23, 131.29, 131.26, 131.20, 131.18, 131.02, 130.71, 130.64, 129.88, 129.83, 129.73, 129.67, 129.54, 129.28, 129.22, 129.08, 129.05, 128.93, 128.79, 128.50, 128.47, 128.40, 127.74, 127.69, 127.62, 127.09, 126.89, 126.27, 126.04, 125.39, 125.35, 124.15, 124.12, 124.08, 120.70, 120.60, 120.03, 119.93, 119.84, 119.78, 119.70, 115.69, 115.67, 115.53, 103.71, 61.50, 56.25, 49.45, 45.07, 44.90, 43.50, 33.77, 32.63, 31.04, 30.09, 29.81, 29.75, 29.69, 29.67, 17.67. ^19^F NMR (376 MHz, CDCl_3_) δ: −113.91, −114.43. HRMS (ESI) *m*/*z* Calcd for C_39_H_35_FN_5_O_3_, [M + H]^+^, 640.2718, found: 640.2726. Enantiomeric excess was determined to be 94% (determined by HPLC using chiral OD-H column, hexane/2-propanol = 9/1, λ = 254 nm, 25 °C, 0.8 mL/min, t_major_ = 86.7 min, t_minor_ = 34.5 min).

*(S)-3-((R)-4-(2-bromobenzyl)-5-oxo-1,3-diphenyl-4,5-dihydro-1H-pyrazol-4-yl)-1-(1-(tert-butyl)-3-phenyl-1H-pyrazol-5-yl)pyrrolidine-2,5-dione* (**3ia**)

This was prepared according to the procedure within 2 h as a white solid (137.1 mg, 98% yield, dr = 1:1), mp 125.1–125.9 °C; ${\left[\alpha \right]}_{D}^{17}$ = −7.206 (*c* 0.68, CH_2_Cl_2_). ^1^H NMR (400 MHz, Chloroform-d) δ: 7.99–7.95 (m, 2H), 7.93–7.89 (m, 2H), 7.87–7.82 (m, 2H), 7.78–7.73 (m, 2H), 7.69–7.65 (m, 2H), 7.63–7.58 (m, 3H), 7.55–7.41 (m, 10H), 7.40–7.35 (m, 3H), 7.34–7.30 (m, 2H), 7.29–7.26 (m, 2H), 7.23–7.19 (m, 2H), 7.07 (ddtd, *J* = 28.0, 14.7, 7.3, 1.8 Hz, 6H), 6.29 (d, *J* = 2.9 Hz, 1H), 5.37 (s, 1H), 4.26 (d, *J* = 7.9 Hz, 1H), 4.21–4.03 (m, 3H), 3.89 (d, *J* = 14.4 Hz, 1H), 3.77 (q, *J* = 7.1 Hz, 2H), 3.17 (dd, *J* = 17.5, 9.0 Hz, 1H), 2.97 (dd, *J* = 18.6, 9.6 Hz, 1H), 2.72 (s, 1H), 1.53 (s, 9H), 1.33 (s, 9H). ^13^C NMR (151 MHz, CDCl_3_) δ: 173.73, 173.70, 172.98, 172.93, 172.27, 158.50, 157.85, 148.93, 148.65, 137.16, 137.05, 133.55, 133.37, 133.29, 133.23, 132.93, 131.10, 130.80, 130.74, 130.71, 130.33, 129.95, 129.49, 129.37, 129.25, 129.13, 129.07, 128.92, 128.84, 128.76, 128.69, 128.46, 128.30, 127.76, 127.67, 127.60, 127.56, 127.24, 126.23, 126.04, 125.77, 125.40, 125.33, 119.77, 119.54, 103.65, 103.63, 61.49, 61.47, 56.05, 45.22, 39.18, 37.65, 30.88, 30.09, 29.82, 29.65. HRMS (ESI) *m*/*z* Calcd for C_39_H_35_BrN_5_O_3_, [M + H]^+^, 700.1918, found: 700.1926. Enantiomeric excess was determined to be 96% (determined by HPLC using chiral OD-H column, hexane/2-propanol = 9/1, λ = 254 nm, 25 °C, 0.8 mL/min, t_major_ = 51.7 min, t_minor_ = 37.6 min).

*(S)-1-(1-(tert-butyl)-3-phenyl-1H-pyrazol-5-yl)-3-((R)-4-(2-nitrobenzyl)-5-oxo-1,3-diphenyl-4,5-dihydro-1H-pyrazol-4-yl)pyrrolidine-2,5-dione* (**3ja**)

This was prepared according to the procedure within 1.2 h as a white solid (129.3 mg, 97% yield, dr = 1:1), mp 120.1–120.9 °C; ${\left[\alpha \right]}_{D}^{17}$ = −42.404 (*c* 0.44, CH_2_Cl_2_). ^1^H NMR (400 MHz, Chloroform-d) δ: 7.97–7.89 (m, 5H), 7.78–7.72 (m, 3H), 7.68–7.64 (m, 4H), 7.63–7.59 (m, 3H), 7.55 (t, *J* = 7.6 Hz, 3H), 7.50 (dd, *J* = 5.2, 2.0 Hz, 4H), 7.47–7.41 (m, 2H), 7.40 (s, 1H), 7.39–7.33 (m, 6H), 7.32 (s, 1H), 7.31–7.26 (m, 4H), 7.22 (dd, *J* = 5.6, 3.2 Hz, 3H), 6.27 (s, 1H), 5.37 (s, 1H), 4.43 (q, *J* = 14.2 Hz, 2H), 4.18 (d, *J* = 14.1 Hz, 1H), 4.09 (q, *J* = 4.4, 3.8 Hz, 2H), 4.06 (s, 1H), 3.73 (s, 1H), 3.24–3.12 (m, 1H), 2.93 (dd, *J* = 18.6, 9.6 Hz, 1H), 2.61 (s, 1H), 1.52 (s, 9H), 1.31 (s, 9H). ^13^C NMR (101 MHz, CDCl_3_) δ: 174.87, 173.65, 173.42, 172.82, 172.12, 157.74, 157.28, 149.97, 148.93, 148.64, 136.88, 136.81, 133.27, 133.20, 133.03, 132.65, 132.32, 132.01, 131.27, 131.08, 130.42, 130.20, 129.54, 129.27, 129.09, 128.99, 128.91, 128.81, 128.72, 128.48, 128.44, 128.26, 127.78, 127.69, 127.60, 127.43, 126.69, 126.32, 126.13, 125.46, 125.37, 125.32, 119.61, 119.55, 103.61, 61.52, 61.47, 56.51, 45.05, 35.22, 34.06, 30.89, 30.03, 29.80, 29.62. HRMS (ESI) *m*/*z* Calcd for C_39_H_35_N_6_O_5_, [M + H]^+^, 667.2663, found: 667.2673. Enantiomeric excess was determined to be 86% (determined by HPLC using chiral IG-H column, hexane/2-propanol = 7/3, λ = 254 nm, 25 °C, 0.8 mL/min, t_major_ = 55.8 min, t_minor_ = 22.1 min).

*(S)-1-(1-(tert-butyl)-3-phenyl-1H-pyrazol-5-yl)-3-((R)-4-(3-methylbenzyl)-5-oxo-1,3-diphenyl-4,5-dihydro-1H-pyrazol-4-yl)pyrrolidine-2,5-dione* (**3ka**)

This was prepared according to the procedure within 1 h as a white solid (120.7 mg, 95% yield, dr = 1:1), mp 109.1–109.9 °C; ${\left[\alpha \right]}_{D}^{17}$ = −40.141 (*c* 0.14, CH_2_Cl_2_). ^1^H NMR (400 MHz, Chloroform-d) δ: 8.04–7.97 (m, 4H), 7.71 (d, *J* = 7.8 Hz, 4H), 7.66–7.52 (m, 10H), 7.38 (ddt, *J* = 16.5, 12.1, 7.9 Hz, 9H), 7.26–7.20 (m, 2H), 7.03–6.80 (m, 9H), 6.33 (s, 1H), 5.55 (s, 1H), 4.32–4.12 (m, 2H), 4.02 (dd, *J* = 9.4, 7.3 Hz, 1H), 3.85–3.75 (m, 1H), 3.60 (dd, *J* = 13.5, 4.0 Hz, 2H), 3.51 (d, *J* = 13.2 Hz, 1H), 3.22 (dd, *J* = 17.8, 9.4 Hz, 1H), 3.05 (dd, *J* = 18.4, 9.6 Hz, 1H), 2.10 (d, *J* = 8.6 Hz, 6H), 1.56 (s, 9H), 1.42 (s, 9H). ^13^C NMR (101 MHz, CDCl_3_) δ: 173.90, 173.75, 173.62, 173.24, 158.24, 148.92, 148.64, 138.00, 137.01, 136.87, 133.17, 132.35, 131.15, 130.94, 130.65, 130.10, 129.32, 129.08, 128.95, 128.78, 128.69, 128.62, 128.46, 128.25, 128.20, 127.66, 127.59, 127.02, 126.27, 126.24, 126.02, 120.05, 119.87, 103.67, 61.48, 57.11, 44.90, 41.26, 31.07, 30.04, 29.80, 29.67, 21.16, 21.10. HRMS (ESI) *m*/*z* Calcd for C_40_H_38_N_5_O_3_, [M + H]^+^, 636.2969, found: 636.2976. Enantiomeric excess was determined to be 96% (determined by HPLC using chiral IB-H column, hexane/2-propanol = 4/1, λ = 254 nm, 25 °C, 0.6 mL/min, t_major_ = 51.1 min, t_minor_ = 41.1 min).

*(S)-1-(1-(tert-butyl)-3-phenyl-1H-pyrazol-5-yl)-3-((R)-4-(4-methylbenzyl)-5-oxo-1,3-diphenyl-4,5-dihydro-1H-pyrazol-4-yl)pyrrolidine-2,5-dione* (**3la**)

This was prepared according to the procedure within 1.5 h as a white solid (124.5 mg, 98% yield, dr = 1:1), mp 112.1–112.9 °C; [${\left[\alpha \right]}_{D}^{17}$ = −30.363 (*c* 0.85, CH_2_Cl_2_). ^1^H NMR (400 MHz, Chloroform-d) δ: 8.04–7.95 (m, 4H), 7.67 (ddd, *J* = 8.1, 5.5, 1.2 Hz, 4H), 7.65–7.58 (m, 5H), 7.58– 7.47 (m, 5H), 7.41–7.25 (m, 10H), 7.24–7.16 (m, 2H), 6.97 (d, *J* = 7.8 Hz, 2H), 6.93–6.84 (m, 6H), 6.29 (s, 1H), 5.50 (s, 1H), 4.20 (dd, *J* = 18.6, 6.7 Hz, 2H), 3.96 (dd, *J* = 9.3, 7.3 Hz, 1H), 3.74 (dd, *J* = 9.3, 5.5 Hz, 1H), 3.57 (dd, *J* = 13.6, 10.3 Hz, 2H), 3.46 (d, *J* = 13.3 Hz, 1H), 3.16 (dd, *J* = 17.9, 9.4 Hz, 1H), 2.99 (dd, *J* = 18.5, 9.7 Hz, 1H), 2.83 (s, 1H), 2.16 (d, *J* = 2.5 Hz, 6H), 1.37 (s, 9H). ^13^C NMR (101 MHz, CDCl_3_) δ: 174.84, 173.87, 173.72, 173.55, 173.19, 173.09, 158.20, 157.51, 148.93, 148.65, 137.58, 137.39, 137.09, 136.93, 133.34, 133.27, 131.15, 131.04, 130.94, 130.65, 130.19, 129.40, 129.35, 129.19, 129.12, 129.07, 128.96, 128.82, 128.67, 128.46, 127.66, 127.60, 127.02, 126.23, 125.99, 125.39, 125.34, 120.07, 119.91, 103.67, 61.48, 57.20, 44.95, 43.63, 40.90, 39.63, 31.04, 30.02, 29.80, 29.67, 20.99. HRMS (ESI) *m*/*z* Calcd for C_40_H_38_N_5_O_3_, [M + H]^+^, 636.2969, found: 636.2976. Enantiomeric excess was determined to be 99% (determined by HPLC using chiral ID-H-OD-H column, hexane/2-propanol = 7/3, λ = 254 nm, 25 °C, 0.8 mL/min, t_major_ = 48.6 min, t_minor_ = 31.9 min).

*(S)-1-(1-(tert-butyl)-3-phenyl-1H-pyrazol-5-yl)-3-((R)-4-(4-methoxybenzyl)-5-oxo-1,3-diphenyl-4,5-dihydro-1H-pyrazol-4-yl)pyrrolidine-2,5-dione* (**3ma**)

This was prepared according to the procedure within 1.5 h as a white solid (129.0 mg, 99% yield, dr = 1:1), mp 112.1–112.9 °C; ${\left[\alpha \right]}_{D}^{17}$ = −29.344 (*c* 0.78, CH_2_Cl_2_). ^1^H NMR (400 MHz, Chloroform-d) δ: 8.04–7.96 (m, 4H), 7.71–7.65 (m, 4H), 7.64–7.58 (m, 5H), 7.58–7.48 (m, 5H), 7.41–7.27 (m, 9H), 7.24–7.17 (m, 2H), 7.04–6.98 (m, 2H), 6.96–6.91 (m, 2H), 6.64–6.57 (m, 4H), 6.29 (s, 1H), 5.51 (s, 1H), 4.20 (dd, *J* = 18.1, 7.4 Hz, 2H), 3.96 (dd, *J* = 9.3, 7.3 Hz, 1H), 3.74 (dd, *J* = 9.0, 5.8 Hz, 1H), 3.63 (d, *J* = 2.0 Hz, 6H), 3.56 (dd, *J* = 18.2, 13.7 Hz, 2H), 3.44 (d, *J* = 13.3 Hz, 1H), 3.16 (dd, *J* = 17.8, 9.4 Hz, 1H), 3.00 (dd, *J* = 18.5, 9.6 Hz, 1H), 2.83 (s, 1H), 1.52 (s, 9H), 1.38 (s, 9H). ^13^C NMR (101 MHz, CDCl_3_) δ: 174.85, 173.91, 173.75, 173.57, 173.22, 173.11, 159.15, 159.02, 158.20, 157.51, 148.92, 148.65, 137.08, 136.91, 133.33, 133.25, 131.15, 131.04, 130.97, 130.67, 130.45, 129.39, 129.15, 128.98, 128.83, 128.70, 128.46, 127.67, 127.60, 127.56, 126.99, 126.24, 126.00, 125.39, 125.34, 125.23, 124.45, 120.02, 119.88, 113.81, 113.76, 103.67, 61.49, 57.30, 55.11, 44.88, 43.59, 40.52, 39.26, 31.04, 30.00, 29.80, 29.68. HRMS (ESI) *m*/*z* Calcd for C_40_H_38_N_5_O_4_, [M + H]^+^, 652.2918, found: 652.2922. Enantiomeric excess was determined to be 99% (determined by HPLC using chiral OD-H column, hexane/2-propanol = 9/1, λ = 254 nm, 25 °C, 0.8 mL/min, t_major_ = 74.5 min, t_minor_ = 51.3 min).

*(S)-3-((R)-4-(3,5-bis(trifluoromethyl)benzyl)-5-oxo-1,3-diphenyl-4,5-dihydro-1H-pyrazol-4-yl)-1-(1-(tert-butyl)-3-phenyl-1H-pyrazol-5-yl)pyrrolidine-2,5-dione* (**3na**)

This was prepared according to the procedure within 2.5 h as a white solid (146.9 mg, 97% yield, dr = 1:1), mp 112.1–112.9 °C; ${\left[\alpha \right]}_{D}^{17}$ = 1.1617 (*c* 0.80, CH_2_Cl_2_). ^1^H NMR (400 MHz, Chloroform-d) δ: 7.94 (d, *J* = 7.4 Hz, 4H), 7.72–7.47 (m, 20H), 7.41–7.30 (m, 10H), 7.23 (d, *J* = 8.7 Hz, 2H), 6.32 (s, 1H), 5.58 (s, 1H), 4.23 (s, 2H), 4.11 (t, *J* = 8.2 Hz, 1H), 3.81 (t, *J* = 7.3 Hz, 1H), 3.70 (dd, *J* = 22.0, 13.6 Hz, 2H), 3.60 (d, *J* = 13.1 Hz, 1H), 3.21 (dd, *J* = 17.6, 9.1 Hz, 1H), 3.03 (dd, *J* = 18.6, 9.7 Hz, 1H), 2.78 (s, 1H), 1.56 (s, 9H), 1.35 (s, 9H). ^13^C NMR (151 MHz, CDCl_3_) δ: 173.53, 173.32, 172.71, 172.68, 172.32, 157.34, 156.70, 148.97, 148.70, 136.49, 136.38, 135.93, 135.13, 133.21, 133.09, 131.96, 131.79, 131.74, 131.57, 131.52, 131.35, 131.29, 130.36 (d, J=14.14 Hz), 129.75, 129.62, 129.42, 129.10, 128.84, 128.63, 128.53, 128.50, 128.29, 127.78, 127.70, 127.13, 126.63, 126.60, 126.44, 125.51, 125.37, 125.30, 123.74, 123.69, 121.93, 121.89, 121.83, 119.66, 119.48, 103.64, 103.59, 61.58, 61.53, 56.82, 44.86, 43.37, 40.35, 39.14, 30.90, 29.81, 29.77, 29.62. ^19^F NMR (376 MHz, CDCl_3_) δ: −63.14, −63.17. HRMS (ESI) *m*/*z* Calcd for C_41_H_34_F_6_N_5_O_3_, [M + H]^+^, 758.2560, found: 758.2570. Enantiomeric excess was determined to be 95% (determined by HPLC using chiral OD-H column, hexane/2-propanol = 9/1, λ = 254 nm, 25 °C, 0.8 mL/min, t_major_ = 41.5 min, t_minor_ = 24.1 min).

*(S)-1-(1-(tert-butyl)-3-phenyl-1H-pyrazol-5-yl)-3-((R)-4-(naphthalen-2-ylmethyl)-5-oxo-1,3-diphenyl-4,5-dihydro-1H-pyrazol-4-yl)pyrrolidine-2,5-dione* (**3oa**)

This was prepared according to the procedure within 4 h as a white solid (99.4 mg, 74% yield, dr = 1:1), mp 116.1–116.9 °C; ${\left[\alpha \right]}_{D}^{17}$ = −66.632 (*c* 0.96, CH_2_Cl_2_). ^1^H NMR (600 MHz, Chloroform-d) δ: 8.19 (d, *J* = 8.3 Hz, 1H), 8.08–8.03 (m, 1H), 7.96–7.93 (m, 2H), 7.92–7.89 (m, 2H), 7.76–7.71 (m, 2H), 7.69–7.56 (m, 8H), 7.52–7.38 (m, 13H), 7.37–7.26 (m, 11H), 7.24–7.14 (m, 9H), 7.13–7.09 (m, 1H), 6.32 (s, 1H), 5.49 (s, 1H), 4.60 (s, 1H), 4.40 (s, 1H), 4.29 (d, *J* = 15.0 Hz, 1H), 4.17 (dd, *J* = 15.4, 6.8 Hz, 2H), 4.00–3.86 (m, 2H), 3.29 (dd, *J* = 17.9, 9.4 Hz, 1H), 3.04 (dd, *J* = 18.9, 9.9 Hz, 1H), 2.88 (s, 1H), 1.56 (d, *J* = 2.0 Hz, 9H), 1.34 (s, 9H). ^13^C NMR (101 MHz, Chloroform-d) δ: 173.91, 173.71, 173.46, 173.02, 158.36, 148.97, 148.62, 136.74, 136.68, 133.82, 133.75, 133.33, 133.25, 131.82, 131.68, 131.31, 131.06, 130.97, 130.60, 129.91, 129.35, 129.06, 128.98, 128.88, 128.79, 128.73, 128.69, 128.54, 128.51, 128.45, 127.90, 127.66, 127.58, 127.04, 126.74, 126.17, 126.04, 125.97, 125.75, 125.70, 125.39, 125.34, 124.92, 124.76, 123.67, 123.50, 120.06, 119.71, 103.67, 103.63, 61.51, 61.47, 56.60, 45.40, 43.90, 36.34, 34.65, 31.09, 30.26, 29.84, 29.65. HRMS (ESI) *m*/*z* Calcd for C_43_H_38_N_5_O_3_, [M + H]^+^, 672.2969, found: 672.2981. Enantiomeric excess was determined to be 94% (determined by HPLC using chiral IB-H column, hexane/2-propanol = 4/1, λ = 254 nm, 25 °C, 0.8 mL/min, t_major_ = 56.4 min, t_minor_ = 47.9 min).

*(S)-3-((R)-4-benzyl-5-oxo-1,3-diphenyl-4,5-dihydro-1H-pyrazol-4-yl)-1-(1-isopropyl-3-phenyl-1H-pyrazol-5-yl)pyrrolidine-2,5-dione* (**3ab**)

This was prepared according to the procedure within 1 h as a white solid (120.2 mg, 99% yield, dr > 20:1), mp 105.1–105.9 °C; ${\left[\alpha \right]}_{D}^{17}$ = 9.067 (*c* 1.15, CH_2_Cl2). ^1^H NMR (400 MHz, Chloroform-d) δ: 8.04–7.95 (m, 2H), 7.76–7.62 (m, 4H), 7.53 (dt, *J* = 5.3, 2.7 Hz, 3H), 7.34 (q, *J* = 7.4 Hz, 4H), 7.27 (d, *J* = 1.4 Hz, 1H), 7.20 (dd, *J* = 7.4 Hz, 1H), 7.15–7.01 (m, 5H), 6.17 (s, 1H), 3.94 (dd, *J* = 9.4, 5.7 Hz, 1H), 3.82 (s, 2H), 3.56 (d, *J* = 13.4 Hz, 1H), 3.16 (dd, *J* = 18.3, 9.7 Hz, 1H), 1.58 (s, 1H), 1.35 (d, *J* = 6.6 Hz, 3H), 1.10 (s, 3H). ^13^C NMR (101 MHz, CDCl_3_) δ: 173.72, 173.51, 172.82, 150.49, 136.88, 133.39, 132.76, 130.98, 130.85, 129.31, 129.21, 128.83, 128.49, 128.40, 128.12, 127.91, 127.68, 127.17, 126.14, 125.51, 119.77, 100.97, 50.25, 44.09, 40.68, 30.51, 22.49, 22.11. HRMS (ESI) *m*/*z* Calcd for C_38_H_34_N_5_O_3_, [M + H]^+^, 608.2656, found: 608.2666. Enantiomeric excess was determined to be 96% (determined by HPLC using chiral OD-H column, hexane/2-propanol = 7/3, λ = 254 nm, 25 °C, 0.8 mL/min, t_major_ = 25.3 min, t_minor_ = 13.6 min).

*(S)-3-((R)-4-benzyl-5-oxo-1,3-diphenyl-4,5-dihydro-1H-pyrazol-4-yl)-1-(1,3-diphenyl-1H-pyrazol-5-yl)pyrrolidine-2,5-dione* (**3ac**)

This was prepared according to the procedure within 1 h as a white solid (123.1 mg, 96% yield, dr > 20:1), mp 108.1–108.9 °C; ${\left[\alpha \right]}_{D}^{17}$ = 11.915 (*c* 1.13, CH_2_Cl_2_). ^1^H NMR (400 MHz, Chloroform-d) δ: 7.98–7.91 (m, 2H), 7.78–7.72 (m, 2H), 7.59–7.48 (m, 5H), 7.43–7.26 (m, 9H), 7.22 (d, *J* = 11.9 Hz, 2H), 7.12–6.99 (m, 5H), 6.28 (s, 1H), 4.02–3.09 (m, 4H), 2.95 (dd, *J* = 18.1, 9.4 Hz, 1H). ^13^C NMR (101 MHz, CDCl_3_) δ: 173.51, 173.01, 172.25, 157.43, 151.85, 137.82, 136.86, 132.51, 131.02, 130.82, 129.78, 129.42, 129.32, 129.25, 128.82, 128.64, 128.35, 128.32, 127.84, 127.19, 126.19, 125.68, 124.19, 120.15, 103.42, 44.16, 40.63, 30.50. HRMS (ESI) *m*/*z* Calcd for C_41_H_32_N_5_O_3_, [M + H]^+^, 642.2500, found: 642.2507. Enantiomeric excess was determined to be 99% (determined by HPLC using chiral OD-H column, hexane/2-propanol = 7/3, λ = 254 nm, 25 °C, 0.8 mL/min, t_major_ = 33.7 min, t_minor_ = 22.2 min).

*(S)-3-((R)-4-benzyl-5-oxo-1,3-diphenyl-4,5-dihydro-1H-pyrazol-4-yl)-1-(1-cyclohexyl-3-phenyl-1H-pyrazol-5-yl)pyrrolidine-2,5-dione* (**3ad**)

This was prepared according to the procedure within 1 h as a white solid (124.3 mg, 96% yield, dr > 20:1), mp 112.1–112.9 °C; ${\left[\alpha \right]}_{D}^{17}$ = 10.511 (*c* 1.05, CH_2_Cl_2_). ^1^H NMR (400 MHz, Chloroform-d) δ: 7.99 (dd, *J* = 6.6, 3.0 Hz, 2H), 7.71–7.65 (m, 2H), 7.63–7.57 (m, 2H), 7.56–7.49 (m, 3H), 7.33 (t, *J* = 7.4 Hz, 4H), 7.27 (d, *J* = 1.4 Hz, 1H), 7.18 (dd, *J* = 7.4 Hz, 1H), 7.09 (q, *J* = 5.0, 3.3 Hz, 5H), 6.16 (s, 1H), 3.96 (t, *J* = 7.9 Hz, 1H), 3.78 (s, 1H), 3.55 (d, *J* = 13.3 Hz, 1H), 3.47 (dt, *J* = 11.3, 6.7 Hz, 1H), 3.17 (dd, *J* = 18.2, 9.6 Hz, 1H), 1.87 (q, *J* = 6.6 Hz, 4H), 1.56–0.76 (m, 6H). ^13^C NMR (151 MHz, CDCl_3_) δ: 173.86, 172.95, 157.77, 150.33, 136.82, 133.41, 130.97, 130.82, 129.36, 129.25, 128.81, 128.51, 128.39, 128.25, 127.95, 127.67, 127.10, 126.24, 125.52, 120.25, 100.94, 57.88, 44.11, 40.89, 32.82, 32.72, 30.52, 25.39, 25.07. HRMS (ESI) *m*/*z* Calcd for C_41_H_38_N_5_O_3_, [M + H]^+^, 648.2969, found: 648.2978. Enantiomeric excess was determined to be 96% (determined by HPLC using chiral OD-H column, hexane/2-propanol = 7/3, λ = 254 nm, 25 °C, 0.8 mL/min, t_major_ = 20.6 min, t_minor_ = 11.3 min).

*(S)-3-((R)-4-benzyl-5-oxo-1,3-diphenyl-4,5-dihydro-1H-pyrazol-4-yl)-1-(1-(tert-butyl)-3-(3-chlorophenyl)-1H-pyrazol-5-yl)pyrrolidine-2,5-dione* (**3ae**)

This was prepared according to the procedure within 1.1 h as a white solid (123.2 mg, 94% yield, dr = 1:1), mp 120.1–120.9 °C; ${\left[\alpha \right]}_{D}^{17}$ = −17.123 (*c* 0.22, CH_2_Cl_2_). ^1^H NMR (400 MHz, Chloroform-d) δ: 8.03–7.97 (m, 4H), 7.71–7.45 (m, 15H), 7.42–7.27 (m, 5H), 7.24–7.16 (m, 4H), 7.13–7.05 (m, 8H), 7.04–7.00 (m, 2H), 6.28 (s, 1H), 5.45 (s, 1H), 4.23 (d, *J* = 26.3 Hz, 2H), 4.00 (dd, *J* = 9.4, 7.4 Hz, 1H), 3.80–3.74 (m, 1H), 3.61 (dd, *J* = 18.0, 13.5 Hz, 2H), 3.50 (d, *J* = 13.2 Hz, 1H), 3.19 (dd, *J* = 17.8, 9.4 Hz, 1H), 3.02 (dd, *J* = 18.4, 9.7 Hz, 1H), 2.83 (s, 1H), 1.52 (s, 9H), 1.38 (s, 9H). ^13^C NMR (101 MHz, CDCl_3_) δ: 174.73, 173.82, 173.63, 173.42, 173.01, 158.05, 157.35, 147.57, 147.29, 136.97, 136.79, 135.09, 134.99, 134.44, 133.23, 132.45, 131.07, 131.03, 131.00, 130.73, 129.75, 129.42, 129.34, 129.17, 129.04, 128.99, 128.72, 128.45, 128.39, 127.96, 127.81, 127.62, 127.58, 126.98, 126.39, 126.06, 125.39, 123.47, 123.36, 120.01, 119.87, 103.89, 103.86, 61.78, 61.75, 57.11, 44.94, 43.72, 41.24, 40.01, 31.03, 30.04, 29.78, 29.65. HRMS (ESI) *m*/*z* Calcd for C_39_H_35_ClN_5_O_3_, [M + H]^+^, 656.2423, found: 656.2434. Enantiomeric excess was determined to be 97% (determined by HPLC using chiral OD-H column, hexane/2-propanol = 8/2, λ = 254 nm, 25 °C, 0.8 mL/min, t_major_ = 36.5 min, t_minor_ = 19.4 min).

*(S)-3-((R)-4-benzyl-5-oxo-1,3-diphenyl-4,5-dihydro-1H-pyrazol-4-yl)-1-(1-(tert-butyl)-3-(3-(trifluoromethyl)phenyl)-1H-pyrazol-5-yl)pyrrolidine-2,5-dione* (**3af**)

This was prepared according to the procedure within 1 h as a white solid (128.2 mg, 93% yield, dr = 1:1), mp 118.1–118.9 °C; ${\left[\alpha \right]}_{D}^{17}$ = −24.115 (*c* 0.68, CH_2_Cl_2_). ^1^H NMR (400 MHz, Chloroform-d) δ: 8.03–7.97 (m, 4H), 7.92 (s, 1H), 7.86–7.79 (m, 3H), 7.66 (d, *J* = 8.1 Hz, 2H), 7.62 (d, *J* = 7.0 Hz, 1H), 7.58 (dd, *J* = 8.3, 3.0 Hz, 4H), 7.51 (dtd, *J* = 11.6, 8.9, 7.8, 3.9 Hz, 6H), 7.44 (d, *J* = 8.1 Hz, 1H), 7.36 (dt, *J* = 16.5, 7.8 Hz, 4H), 7.24–7.17 (m, 2H), 7.13–7.06 (m, 8H), 7.03 (d, *J* = 7.0 Hz, 2H), 6.35 (s, 1H), 5.46 (s, 1H), 4.31–4.15 (m, 2H), 4.00 (dd, *J* = 9.4, 7.4 Hz, 1H), 3.78 (dd, *J* = 9.3, 5.4 Hz, 1H), 3.62 (dd, *J* = 18.9, 13.5 Hz, 2H), 3.51 (d, *J* = 13.2 Hz, 1H), 3.20 (dd, *J* = 17.9, 9.4 Hz, 1H), 3.03 (dd, *J* = 18.5, 9.7 Hz, 1H), 2.83 (s, 1H), 1.54 (s, 10H), 1.39 (s, 9H). ^13^C NMR (101 MHz, CDCl_3_) δ: 174.72, 173.81, 173.62, 173.43, 173.01, 158.05, 157.32, 147.51, 147.25, 136.97, 136.78, 134.05, 133.97, 133.24, 132.43, 131.06, 131.00, 130.95, 130.74, 130.67, 129.44, 129.33, 129.20, 129.17, 128.98, 128.93, 128.87, 128.72, 128.46, 128.39, 127.97, 127.81, 127.64, 126.97, 126.37, 126.07, 124.19, 122.14, 120.02, 119.85, 103.92, 103.89, 61.88, 61.85, 57.10, 44.96, 43.68, 41.25, 40.00, 31.07, 30.05, 29.78, 29.65. ^19^F NMR (376 MHz, CDCl_3_) δ: −62.62, −62.70. HRMS (ESI) *m*/*z* Calcd for C_40_H_35_F_3_N_5_O_3_, [M + H]^+^, 690.2687, found: 690.2694. Enantiomeric excess was determined to be 96% (determined by HPLC using chiral OD-H column, hexane/2-propanol = 8/2, λ = 254 nm, 25 °C, 0.8 mL/min, t_major_ = 34.9 min, t_minor_ = 16.9 min).

*(S)-3-((R)-4-benzyl-5-oxo-1,3-diphenyl-4,5-dihydro-1H-pyrazol-4-yl)-1-(1-(tert-butyl)-3-(3-methoxyphenyl)-1H-pyrazol-5-yl)pyrrolidine-2,5-dione* (**3ag**)

This was prepared according to the procedure within 1 h as a white solid (125.0 mg, 96% yield, dr = 1:1), mp 114.1–114.9 °C; ${\left[\alpha \right]}_{D}^{17}$ = −37.751 (*c* 0.25, CH_2_Cl_2_). ^1^H NMR (400 MHz, Chloroform-d) δ: 7.98 (d, *J* = 7.6 Hz, 4H), 7.67–7.46 (m, 10H), 7.34 (dt, *J* = 15.4, 7.7 Hz, 4H), 7.26–7.15 (m, 8H), 7.07 (d, *J* = 9.5 Hz, 8H), 7.00 (d, *J* = 7.5 Hz, 2H), 6.85–6.75 (m, 2H), 6.29 (s, 1H), 5.50 (s, 1H), 4.21 (q, *J* = 7.8, 7.3 Hz, 2H), 3.97 (t, *J* = 8.3 Hz, 1H), 3.80 (d, *J* = 21.3 Hz, 7H), 3.58 (dd, *J* = 13.6, 6.5 Hz, 2H), 3.47 (d, *J* = 13.2 Hz, 1H), 3.15 (dd, *J* = 17.8, 9.4 Hz, 1H), 3.00 (dd, *J* = 18.5, 9.6 Hz, 1H), 1.52 (s, 9H), 1.35 (s, 9H). ^13^C NMR (101 MHz, CDCl_3_) δ: 174.88, 173.92, 173.79, 173.47, 173.10, 159.83, 159.78, 158.11, 157.40, 148.78, 148.50, 137.01, 136.82, 134.73, 134.63, 133.31, 132.53, 131.09, 131.04, 131.00, 130.73, 129.54, 129.44, 129.37, 129.19, 129.00, 128.85, 128.73, 128.45, 128.38, 127.94, 127.81, 127.60, 126.99, 126.34, 126.07, 120.04, 119.91, 118.07, 118.01, 113.42, 110.77, 103.90, 103.88, 61.55, 57.12, 55.29, 55.25, 44.94, 43.60, 41.23, 40.04, 31.05, 30.03, 29.82, 29.70. HRMS (ESI) *m*/*z* Calcd for C_40_H_38_N_5_O_4_, [M + H]^+^, 652.2918, found: 562.2926. Enantiomeric excess was determined to be 97% (determined by HPLC using chiral OD-H column, hexane/2-propanol = 8/2, λ = 254 nm, 25 °C, 0.8 mL/min, t_major_ = 43.0 min, t_minor_ = 23.3 min).

*(S)-3-((R)-4-benzyl-5-oxo-1,3-diphenyl-4,5-dihydro-1H-pyrazol-4-yl)-1-(1-(tert-butyl)-3-(4-fluorophenyl)-1H-pyrazol-5-yl)pyrrolidine-2,5-dione* (**3ah**)

This was prepared according to the procedure within 1 h as a white solid (120.2 mg, 94% yield, dr = 1:1), mp 104.1–104.9 °C; ${\left[\alpha \right]}_{D}^{17}$ = −24.525 (*c* 0.58, CH_2_Cl_2_). ^1^H NMR (400 MHz, Chloroform-d) δ: 7.99 (ddt, *J* = 6.7, 4.2, 1.9 Hz, 4H), 7.67–7.48 (m, 14H), 7.41–7.30 (m, 4H), 7.23– 7.16 (m, 2H), 7.13–6.96 (m, 14H), 6.24 (s, 1H), 5.47 (s, 1H), 4.22 (dd, *J* = 17.9, 7.2 Hz, 2H), 3.99 (dd, *J* = 9.3, 7.4 Hz, 1H), 3.81–3.72 (m, 1H), 3.61 (dd, *J* = 18.5, 13.5 Hz, 2H), 3.49 (d, *J* = 13.2 Hz, 1H), 3.18 (dd, *J* = 17.8, 9.4 Hz, 1H), 3.02 (dd, *J* = 18.4, 9.6 Hz, 1H), 1.52 (s, 9H), 1.37 (s, 9H). ^13^C NMR (151 MHz, CDCl_3_) δ: 173.86, 173.71, 173.42, 173.09, 173.04, 163.36, 163.32, 161.72, 161.69, 158.05, 157.35, 148.07, 147.79, 136.97, 136.82, 133.22, 132.45, 131.08, 131.01, 130.95, 130.72, 129.55, 129.48, 129.39, 129.34, 129.17, 128.98, 128.89, 128.71, 128.57, 128.44, 128.39, 127.96, 127.81, 127.56, 127.07, 127.01, 126.99, 126.96, 126.28, 126.06, 120.02, 119.88, 115.37 (d, *J* = 15.15Hz), 103.47, 103.43, 61.55, 57.11, 44.93, 41.24, 40.04, 31.03, 30.04, 29.79, 29.66. ^19^F NMR (376 MHz, CDCl3) δ: −114.70, −114.83. HRMS (ESI) *m*/*z* Calcd for C_39_H_35_FN_5_O_3_, [M + H]^+^, 640.2718, found: 640.2731. Enantiomeric excess was determined to be 97% (determined by HPLC using chiral OD-H column, hexane/2-propanol = 8/2, λ = 254 nm, 25 °C, 0.8 mL/min, t_major_ = 34.2 min, t_minor_ = 18.0 min).

*(S)-3-((R)-4-benzyl-5-oxo-1,3-diphenyl-4,5-dihydro-1H-pyrazol-4-yl)-1-(1-(tert-butyl)-3-(4-chlorophenyl)-1H-pyrazol-5-yl)pyrrolidine-2,5-dione* (**3ai**)

This was prepared according to the procedure within 1 h as a white solid (125.8 mg, 96% yield, dr = 1:1), mp 101.1–101.9 °C; ${\left[\alpha \right]}_{D}^{17}$ = −29.557 (*c* 0.77, CH_2_Cl_2_). ^1^H NMR (400 MHz, Chloroform-d) δ: 8.07–8.00 (m, 4H), 7.71–7.66 (m, 2H), 7.65–7.52 (m, 12H), 7.43–7.29 (m, 8H), 7.27–7.18 (m, 2H), 7.11 (q, *J* = 6.8, 5.1 Hz, 8H), 7.09–7.02 (m, 2H), 6.30 (s, 1H), 5.51 (s, 1H), 4.26 (d, *J* = 25.9 Hz, 2H), 4.02 (dd, *J* = 9.3, 7.4 Hz, 1H), 3.83–3.75 (m, 1H), 3.65 (dd, *J* = 21.6, 13.5 Hz, 2H), 3.53 (d, *J* = 13.2 Hz, 1H), 3.22 (dd, *J* = 17.8, 9.4 Hz, 1H), 3.05 (dd, *J* = 18.4, 9.6 Hz, 1H), 2.87 (s, 1H), 1.55 (s, 9H), 1.40 (s, 9H). ^13^C NMR (101 MHz, CDCl_3_) δ: 174.77, 173.84, 173.66, 173.41, 173.06, 173.03, 158.04, 157.34, 147.84, 147.55, 136.97, 136.82, 133.36, 133.30, 133.21, 132.44, 131.83, 131.76, 131.08, 131.02, 130.94, 130.72, 129.38, 129.33, 129.17, 128.98, 128.71, 128.64, 128.44, 128.39, 127.96, 127.81, 127.55, 126.98, 126.62, 126.57, 126.28, 126.06, 103.66, 103.63, 61.68, 57.10, 44.93, 43.70, 41.24, 40.04, 31.02, 30.04, 29.78, 29.65. HRMS (ESI) *m*/*z* Calcd for C_39_H_35_ClN_5_O_3_, [M + H]^+^, 656.2423, found: 656.2433. Enantiomeric excess was determined to be 98% (determined by HPLC using chiral OD-H column, hexane/2-propanol = 8/2, λ = 254 nm, 25 °C, 0.8 mL/min, t_major_ = 34.4 min, t_minor_ = 18.5 min).

*(S)-3-((R)-4-benzyl-5-oxo-1,3-diphenyl-4,5-dihydro-1H-pyrazol-4-yl)-1-(3-(4-bromophenyl)-1-(tert-butyl)-1H-pyrazol-5-yl)pyrrolidine-2,5-dione* (**3aj**)

This was prepared according to the procedure within 1 h as a white solid (130.0 mg, 93% yield, dr = 1:1), mp 126.1–126.9 °C; ${\left[\alpha \right]}_{D}^{17}$ = −27.602 (*c* 0.22, CH_2_Cl_2_). ^1^H NMR (400 MHz, Chloroform-d) δ: 7.99 (ddt, *J* = 6.9, 5.4, 2.5 Hz, 4H), 7.68–7.62 (m, 2H), 7.61–7.49 (m, 10H), 7.48–7.30 (m, 10H), 7.24–7.16 (m, 2H), 7.14–6.99 (m, 10H), 6.27 (s, 1H), 5.49 (s, 1H), 4.23 (dd, *J* = 18.1, 7.4 Hz, 2H), 3.99 (dd, *J* = 9.4, 7.4 Hz, 1H), 3.82–3.75 (m, 1H), 3.61 (dd, *J* = 20.1, 13.5 Hz, 2H), 3.50 (d, *J* = 13.2 Hz, 1H), 3.19 (dd, *J* = 17.9, 9.4 Hz, 1H), 3.02 (dd, *J* = 18.3, 9.6 Hz, 1H), 2.84 (s, 1H), 1.52 (s, 9H), 1.37 (s, 9H). ^13^C NMR (101 MHz, CDCl_3_) δ: 173.83, 173.65, 173.41, 173.05, 173.02, 158.03, 157.33, 147.85, 147.55, 136.96, 136.81, 133.20, 132.43, 132.27, 132.20, 131.58, 131.07, 131.02, 130.94, 130.72, 129.38, 129.33, 129.17, 128.98, 128.71, 128.68, 128.44, 128.39, 127.96, 127.81, 127.54, 126.97, 126.93, 126.89, 126.28, 126.06, 121.55, 121.49, 120.02, 119.87, 103.66, 103.63, 61.70, 57.10, 44.93, 43.69, 41.24, 40.04, 31.02, 30.04, 29.78, 29.65. HRMS (ESI) *m*/*z* Calcd for C_39_H_35_BrN_5_O_3_, [M + H]^+^, 700.1918, found: 700.1924. Enantiomeric excess was determined to be 97% (determined by HPLC using chiral OD-H column, hexane/2-propanol = 8/2, λ = 254 nm, 25 °C, 0.8 mL/min, t_major_ = 36.0 min, t_minor_ = 19.1 min).

*(S)-3-((R)-4-benzyl-5-oxo-1,3-diphenyl-4,5-dihydro-1H-pyrazol-4-yl)-1-(1-(tert-butyl)-3-(p-tolyl)-1H-pyrazol-5-yl)pyrrolidine-2,5-dione* (**3ak**)

This was prepared according to the procedure within 1 h as a white solid (120.7 mg, 95% yield, dr = 1:1), mp 125.1–125.9 °C; ${\left[\alpha \right]}_{D}^{17}$ = −39.223 (*c* 0.28, CH_2_Cl_2_). ^1^H NMR (400 MHz, Chloroform-d) δ: 8.01–7.92 (m, 4H), 7.67–7.62 (m, 2H), 7.62–7.45 (m, 12H), 7.33 (dt, *J* = 16.2, 7.9 Hz, 4H), 7.23–7.17 (m, 2H), 7.17–7.09 (m, 5H), 7.06 (d, *J* = 11.2 Hz, 7H), 6.99 (dd, *J* = 7.9, 1.8 Hz, 2H), 6.26 (s, 1H), 5.49 (s, 1H), 4.24–4.12 (m, 2H), 3.94 (dd, *J* = 9.3, 7.3 Hz, 1H), 3.75 (dd, *J* = 8.9, 5.8 Hz, 1H), 3.57 (d, *J* = 14.2 Hz, 2H), 3.45 (d, *J* = 13.2 Hz, 1H), 3.12 (dd, *J* = 17.8, 9.4 Hz, 1H), 2.98 (dd, *J* = 18.4, 9.6 Hz, 1H), 2.33 (s, 3H), 2.29 (s, 3H), 1.51 (s, 9H), 1.37 (s, 9H). ^13^C NMR (101 MHz, CDCl_3_) δ: 174.89, 173.95, 173.82, 173.48, 173.17, 173.12, 158.13, 157.45, 149.05, 148.77, 137.39, 137.32, 137.05, 136.88, 133.35, 132.58, 131.14, 131.03, 130.71, 130.61, 130.53, 129.42, 129.39, 129.21, 129.18, 129.01, 128.78, 128.73, 128.45, 128.42, 128.38, 127.93, 127.81, 127.59, 127.02, 126.31, 126.06, 125.34, 125.29, 120.06, 119.92, 103.47, 61.41, 57.15, 44.95, 43.67, 41.23, 40.06, 31.04, 30.03, 29.83, 29.73, 21.34, 21.30. HRMS (ESI) *m*/*z* Calcd for C_40_H_38_N_5_O_3_, [M + H]^+^, 636.2969, found: 636.2976. Enantiomeric excess was determined to be 99% (determined by HPLC using chiral OD-H column, hexane/2-propanol = 8/2, λ = 254 nm, 25 °C, 0.8 mL/min, t_major_ = 31.7 min, t_minor_ = 16.6 min).

### 3.4. Procedure for the Synthesis of Compounds **4**

*(S)-1-(4-bromo-1-(tert-butyl)-3-phenyl-1H-pyrazol-5-yl)-3-((R)-4-(2-bromobenzyl)-5-oxo-1,3-diphenyl-4,5-dihydro-1H-pyrazol-4-yl)pyrrolidine-2,5-dione* (**4**)

This was prepared according to the procedure within 96 h as a white solid (106.1mg, 58% yield, dr > 20:1), mp 126.1–126.9 °C; ${\left[\alpha \right]}_{D}^{17}$ = −22.000 (*c* 0.50, CH_2_Cl_2_). ^1^H NMR (400 MHz, Chloroform-d) δ: 7.95 (dd, *J* = 6.8, 2.9 Hz, 2H), 7.79 (dd, *J* = 11.0, 7.5 Hz, 4H), 7.49 (dd, *J* = 6.4, 3.6 Hz, 4H), 7.38 (q, *J* = 7.6 Hz, 4H), 7.31 (dd, *J* = 7.3 Hz, 1H), 7.21 (s, 1H), 7.14–6.99 (m, 3H), 4.19–4.02 (m, 2H), 3.92 (d, *J* = 14.4 Hz, 1H), 3.82 (d, *J* = 14.6 Hz, 1H), 3.24 (dd, *J* = 17.2, 8.7 Hz, 1H), 1.30 (s, 9H). ^13^C NMR (101 MHz, CDCl_3_) δ: 172.93, 172.85, 172.68, 158.49, 145.99, 137.17, 133.37, 132.98, 132.06, 130.84, 130.68, 130.20, 129.46, 129.10, 128.85, 128.38, 128.29, 128.17, 127.58, 127.30, 126.03, 125.46, 119.70, 93.34, 62.45, 56.44, 45.18, 39.06, 30.28, 29.33. HRMS (ESI) *m*/*z* Calcd for C_39_H_34_Br_2_N_5_O_3_, [M + H]^+^, 778.1023, found: 778.1022. Enantiomeric excess was determined to be 96% (determined by HPLC using chiral IB-H column, hexane/2-propanol = 9/1, λ = 254 nm, 25 °C, 0.8 mL/min, t_major_ = 30.3 min, t_minor_ = 26.8 min).

### 3.5. Procedure for the Synthesis of Compounds **6**

*4-(1-(1-(tert-butyl)-3-phenyl-1H-pyrazol-5-yl)-2,5-dioxopyrrolidin-3-yl)-1,3-diphenyl-1H-pyrazol-5-yl acetate* (**6**)

This was prepared according to the procedure within 18 h as a white solid (68.8 mg, 60% yield, dr = 6:1), mp 126.1–126.9 °C. ^1^H NMR (600 MHz, Chloroform-d) δ: 7.77 (d, *J* = 7.6 Hz, 2H), 7.69 (d, *J* = 7.3 Hz, 2H), 7.59 (d, *J* = 7.9 Hz, 2H), 7.50 (q, *J* = 8.1 Hz, 5H), 7.39 (dd, *J* = 7.5 Hz, 4H), 6.33 (s, 1H), 4.21 (dd, *J* = 10.2, 5.2 Hz, 1H), 3.25 (dd, *J* = 18.8, 10.1 Hz, 1H), 3.04 (dd, *J* = 18.7, 5.3 Hz, 1H), 2.26 (s, 3H), 1.60 (s, 9H). ^13^C NMR (101 MHz, CDCl_3_) δ: 175.53, 174.64, 167.28, 151.32, 149.07, 141.74, 137.56, 133.39, 132.65, 129.39, 128.96, 128.94, 128.79, 128.55, 128.22, 127.74, 125.47, 123.50, 104.06, 103.44, 61.51, 36.50, 29.92, 20.56. HRMS (ESI) *m*/*z* Calcd for C_34_H_32_N_5_O_4_, [M + H]^+^, 574.2449, found: 574.2455.

## 4. Conclusions

In conclusion, we developed a novel organocatalyzed pathway to realize the desymmetrization of *N*-pyrazolyl maleimides via a Michael addition reaction, achieving various pyrazolyl–succinimides in high enantioselectivities. It is noteworthy that the novel asymmetric synthesis strategy worked well with a broad substrate scope and excellent atom economy. In addition, the bifunctional quinine-derived thiourea catalyst was fundamental for the desymmetrization, which produced the chiral axis and the adjacent stereocenters simultaneously. Moreover, further exploration of novel synthesis strategies to construct C–N five-membered bi-heterocyclic skeletons are underway in our laboratory.

## Figures and Tables

**Table 1:** Optimization of reaction conditions.

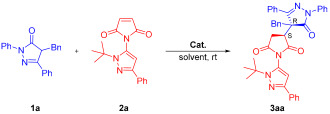							
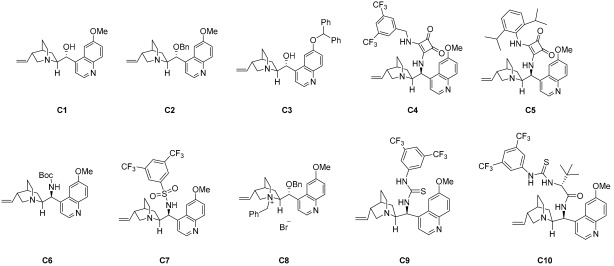							
Entry *^a^*	Cat.	Solvent	t [h]	T [°C]	Yield (%) *^b^*	Dr *^c^*	Ee [%] *^d^*
1	DBACO	DCM	0.5	25	99	1:1	-
2	**C1**	DCM	0.3	25	91	1:1	40
3	**C2**	DCM	9.5	25	98	1:1	14
4	**C3**	DCM	4	25	98	1:1	49
5	**C4**	DCM	0.5	25	99	1:1	81
6	**C5**	DCM	4	25	99	1:1	0
7	**C6**	DCM	2.5	25	85	1:1	80
8	**C7**	DCM	6	25	77	1:1	13
9	**C8**	DCM	24	25	48	1:1	21
10	**C9**	DCM	2.5	25	99	1:1	80
11	**C10**	DCM	3	25	99	1:1	91
12	**C10**	toluene	0.5	25	99	1:1	94
13	**C10**	1,4-dioxane	18	25	99	1:1	91
14	**C10**	DCE	4.5	25	99	1:1	89
15	**C10**	CCl_4_	18	25	99	1:1	92
16	**C10**	CHCl_3_	18	25	99	1:1	91
17 *^e^*	**C10**	toluene	1	25	99	1:1	99
18	**C10**	toluene	2	0	98	1:1	99
19	**C10**	toluene	3.5	−10	96	1:1	99

*^a^* The reaction was conducted with **1a** (0.12 mmol), **2a** (0.1 mmol) and **Cat.** (10 mol%) in solvent (1.0 mL). ^*b*^ Isolated yield. ^*c*^ Determined by ^1^H NMR of the crude mixture. ^*d*^ Detected by chiral HPLC analysis for both diastereomers. *^e^* toluene (2.0 mL) was used.

## Data Availability

Not applicable.

## Supplementary Materials

The following supporting information can be downloaded at: https://www.mdpi.com/article/10.3390/molecules28114279/s1, materials and methods [44,45,46], catalyst synthesis methods [47,48,49,50,51,52], experimental procedures, characterization data, ^1^H, ^13^C and ^19^F NMR spectra, HRMS spectrometry data and HPLC chromatogram.

## Sample Availability

Samples of the compounds are available from the authors.
