# Supplementary material for: Desymmetrization of Prochiral N-Pyrazolyl Maleimides via Organocatalyzed Asymmetric Michael Addition with Pyrazolones: Construction of Tri-N-Heterocyclic Scaffolds Bearing Both Central and Axial Chirality
Source: Molecules. 2023 May 23;28(11):4279. doi: 10.3390/molecules28114279 (PMC10254726; doi:10.3390/molecules28114279)
Supplement: Supplementary file 1 [file molecules-28-04279-s001.zip › Supporting Information-molecules-2399445-final-updated.pdf]

Desymmetrisation of Prochiral *N*-Pyrazolyl Maleimides via Organocatalyzed Asymmetric Michael Addition with Pyrazolones: Construction of Tri-*N*-heterocyclic Scaffolds Bearing both Central and Axial Chirality

Jianqi Geng, Xingfu Wei, Biru He, Yuting Hao, Jingping Qu and Baomin Wang*

State Key Laboratory of Fine Chemicals, Department of Pharmaceutical Sciences, School of Chemical Engineering, Dalian University of Technology, Dalian 116024, P. R. China

Contents:

1. General information 1
2. General procedures for preparation of pyrazol-5-ones **1** 1
3. General procedures for preparation of pyrazol-maleimide **2** 1
4. Experimental procedures and characterization of products **3** and **4** 4
5. X-ray crystal structure of **4** 37
6. Copies of ^{1}H NMR and ^{13}C NMR spectra 39

1. General information

Unless otherwise noted, materials were purchased from commercial suppliers and used without further purification. Column chromatography was performed on silica gel (200~300 mesh). Enantiomeric excesses (ee) were determined by HPLC (Agilent, Palo Alto, CA, USA) using corresponding commercial chiral columns as stated at 25 °C with UV detector at 254 nm. Optical rotations (JiaHang Instruments, Shanghai, China) were reported as follows: $[\alpha]_D^T$ (*c* g/100 mL, solvent). All ^{1}H NMR and ^{19}F NMR spectra were recorded on a Bruker Avance II 400 MHz (Bruker, Karlsruhe, Germany) and Bruker Avance III 600 MHz (Bruker, Karlsruhe, Germany), respectively, ^{13}C NMR spectra were recorded on a Bruker Avance II 101 MHz or Bruker Avance III 151 MHz with chemical shifts reported as ppm (in $CDCl_3$, TMS as an internal standard). Data for ^{1}H NMR are recorded as follows: chemical shift (δ, ppm), multiplicity (s = singlet, d = doublet, t = triplet, m = multiplet, br = broad singlet, dd = double doublet, coupling constants in Hz, integration). HRMS (ESI) was obtained with a HRMS/MS instrument (LTQ Orbitrap XL TM, Agilent, Palo Alto, CA, USA). The absolute configuration of **4** was assigned by the X-ray analysis.

2. General procedures for preparation of pyrazol-5-ones 1

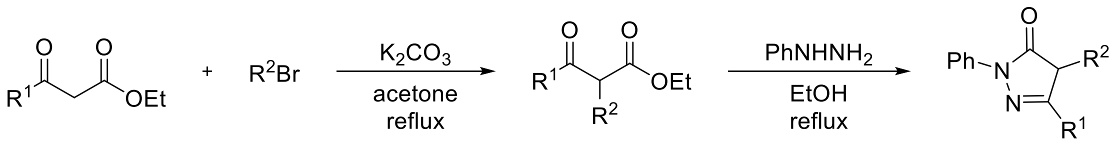

S1 **S2** **1**

A mixture of *β*-keto ester **S1** (10 mmol) and anhydrous K_2CO_3 (13 mmol) in dry acetone was stirred under argon atmosphere for five minutes. Then, alkyl bromide or corresponding benzyl bromide (13 mmol) was added carefully. Then reaction was refluxed overnight. After filtration, the solvent was evaporated. The crude mixture purified by flash chromatography on silica gel with mixture of hexane/ethyl acetate (20:1) affording corresponding pure compound **S2**,

A mixture of **S2** (1.0 eq) and phenylhydrazine (1.0 eq) was refluxed in EtOH until full conversion. The solvent was removed and a residue was crystallized from Et_2O. Solid material was filtered affording corresponding pyrazol-5-ones **1**. NMR data fit with data published in the literature.

3. General procedures for preparation of pyrazol-maleimide 2

Typical procedure for the synthesis of substrates 2a–2k

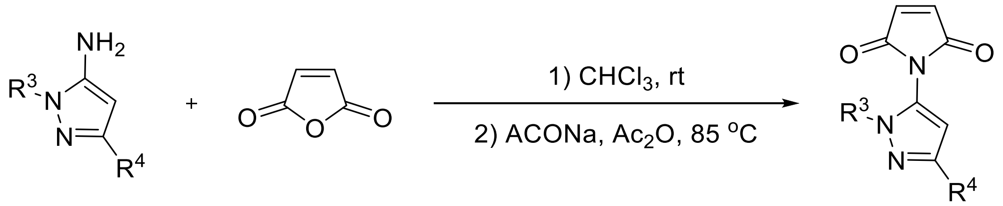

S3 **2**

5-Aminopyrazole **S3** were prepared according to the literature. 1) The maleic anhydride (7.5 mmol) and 5-aminopyrazole **S3** (5 mmol) were dissolved in 10 mL $CHCl_3$, stirred for 10 hours, and the solid (maleimide acid) precipitated from the reaction mixture was filtered. 2) Maleimide acid was dissolved in 20 mL acetic anhydride and 200 mg sodium acetate was added. Heat the mixture at 85 °C and stir for 4 hours. The reaction is cooled and quenched with water, mixture was filtered, quenched with water and extracted with ethyl acetate. The organic phase was separated, washed

with water and dried over Na_2SO_4. The product was purified by silica gel column chromatography with a mixture of petroleum ether and ethyl acetate (10:1) as eluent. The target compound **2** (0.96 g, 65 %) was obtained as a solid.

Characterization of substrates 2a–2k

1-(1-(tert-butyl)-3-phenyl-1H-pyrazol-5-yl)-1H-pyrrole-2,5-dione (2a)

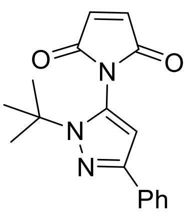

White solid (960 mg, 65% yield, Petroleum ether/EtOAc = 10/1 as the eluent), mp 154.1 - 154.9 °C. ^{1}H NMR (600 MHz, Chloroform-d) δ 7.80 – 7.75 (m, 2H), 7.38 (dd, J = 7.5 Hz, 2H), 7.31 – 7.27 (m, 1H), 6.92 (d, J = 3.4 Hz, 2H), 6.47 (s, 1H), 1.59 (s, 9H). ^{13}C NMR (101 MHz, $CDCl_3$) δ 169.10, 148.85, 135.29, 133.38, 128.54, 127.99, 127.71, 125.45, 104.50, 61.24, 29.90. HRMS (m/z): Calcd for $C_{17}H_{18}N_3O_2$, $[M+H]^+$, 296.1394, found: 296.1392.

1-(1-isopropyl-3-phenyl-1H-pyrazol-5-yl)-1H-pyrrole-2,5-dione (2b)

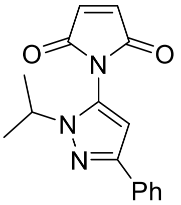

White solid (300 mg, 60% yield, Petroleum ether/EtOAc = 10/1 as the eluent), mp 115.1 - 115.9 °C. ^{1}H NMR (400 MHz, Chloroform-d) δ 7.84 – 7.76 (m, 2H), 7.38 (dd, J = 7.5 Hz, 2H), 7.33 – 7.26 (m, 1H), 6.90 (d, J = 1.8 Hz, 2H), 6.48 (s, 1H), 4.14 (h, J = 6.6 Hz, 1H), 1.50 (d, J = 6.6 Hz, 6H). ^{13}C NMR (101 MHz, $CDCl_3$) δ 168.48, 150.58, 135.04, 133.47, 128.57, 127.78, 125.56, 101.61, 50.35, 22.63. HRMS (m/z): Calcd for $C_{16}H_{16}N_3O_2$, $[M+H]^+$, 282.1237, found: 282.1240.

1-(1,3-diphenyl-1H-pyrazol-5-yl)-1H-pyrrole-2,5-dione (2c)

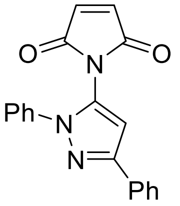

White solid (200 mg, 40% yield, Petroleum ether/EtOAc = 10/1 as the eluent), mp 108.1 - 108.9 °C. ^{1}H NMR (400 MHz, Chloroform-d) δ 7.90 – 7.83 (m, 2H), 7.46 – 7.34 (m, 8H), 6.81 (s, 2H), 6.76 (s, 1H). ^{13}C NMR (101 MHz, $CDCl_3$) δ 168.11, 152.02, 138.22, 134.94, 132.60, 129.54, 129.38, 128.69, 128.65, 128.36, 125.76, 124.51, 104.10. HRMS (m/z): Calcd for $C_{19}H_{14}N_3O_2$, $[M+H]^+$, 316.1081, found: 316.1080.

1-(1-cyclohexyl-3-phenyl-1H-pyrazol-5-yl)-1H-pyrrole-2,5-dione (2d)

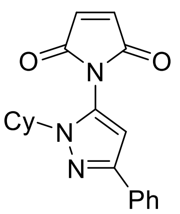

White solid (400 mg, 50% yield, Petroleum ether/EtOAc = 10/1 as the eluent), mp 105.1 - 105.9 °C. ^{1}H NMR (400 MHz, Chloroform-d) δ 7.79 (d, J = 7.6 Hz, 2H), 7.38 (t, J = 7.5 Hz, 2H), 7.30 (d, J = 7.3 Hz, 1H), 6.93 (s, 2H), 6.47 (s, 1H), 3.67 (tt, J = 10.9, 4.5 Hz, 1H), 2.16 – 1.79 (m, 7H), 1.74 – 1.62 (m, 1H), 1.31 (d, J = 11.7 Hz, 3H). ^{13}C NMR (101 MHz, $CDCl_3$) δ 168.51, 150.45, 135.05, 133.48, 128.54, 127.97, 127.72, 125.57, 101.51, 58.04, 32.91, 25.65, 25.12. HRMS (m/z): Calcd for $C_{19}H_{20}N_3O_2$, $[M+H]^+$, 322.1550, found: 322.1551.

1-(1-(tert-butyl)-3-(3-chlorophenyl)-1H-pyrazol-5-yl)-1H-pyrrole-2,5-dione (2e)

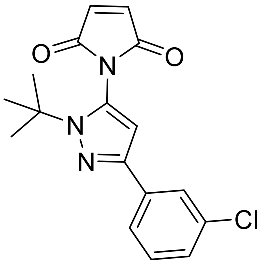

Orange solid (600 mg, 52% yield, Petroleum ether/EtOAc = 10/1 as the eluent), mp 140.1 - 140.9 °C. ^{1}H NMR (400 MHz, Chloroform-d) δ 7.79 (s, 1H), 7.63 (dt, J = 7.5, 1.5 Hz, 1H), 7.33 – 7.25 (m, 2H), 6.94 (d, J = 0.9 Hz, 2H), 6.46 (s, 1H), 1.58 (s, 9H). ^{13}C NMR (101 MHz, $CDCl_3$) δ 168.99, 147.50, 135.32, 135.17, 134.51, 129.81, 128.25, 127.66, 125.46, 123.55, 104.70, 61.52, 29.87. HRMS (m/z): Calcd for $C_{17}H_{17}ClN_3O_2$, $[M+H]^+$, 330.1004, found: 330.0999.

1-(1-(tert-butyl)-3-(3-(trifluoromethyl)phenyl)-1H-pyrazol-5-yl)-1H-pyrrole-2,5-dione (2f)

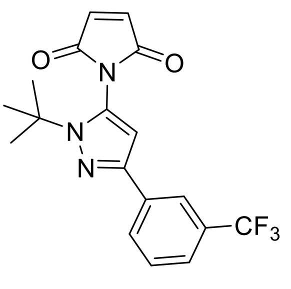

White solid (400 mg, 53% yield, Petroleum ether/EtOAc = 10/1 as the eluent), mp 146.1 - 146.9 °C. ^{1}H NMR (400 MHz, Chloroform-d) δ 8.03 (d, J = 2.2 Hz, 1H), 7.97 – 7.93 (m, 1H), 7.56 – 7.47 (m, 2H), 6.95 (s, 2H), 6.52 (s, 1H), 1.60 (s, 9H). ^{13}C NMR (101 MHz, CDCl3) δ 168.98, 147.46, 135.34, 134.15, 131.08, 130.76, 128.99, 128.57, 128.41, 125.60, 124.25 (d, J= 4.04 Hz), 122.89, 122.21 (d, J= 4.04 Hz), 104.73, 61.62, 29.87. ^{19}F NMR (565 MHz, $CDCl_3$) δ -62.66. HRMS (m/z): Calcd for $C_{18}H_{17}F_3N_3O_2$, $[M+H]^+$, 364.1267, found: 364.1267.

1-(1-(tert-butyl)-3-(3-methoxyphenyl)-1H-pyrazol-5-yl)-1H-pyrrole-2,5-dione (2g)

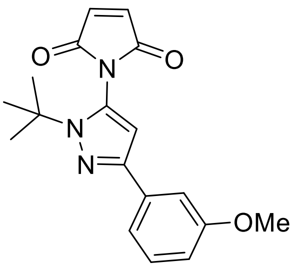

White solid (300 mg, 50% yield, Petroleum ether/EtOAc = 10/1 as the eluent), mp 103.1 - 103.9 °C. ^{1}H NMR (400 MHz, Chloroform-d) δ 7.35 (s, 2H), 7.31 (s, 1H), 6.91 (s, 2H), 6.84 (s, 1H), 6.45 (s, 1H), 3.85 (s, 3H), 1.58 (s, 9H). ^{13}C NMR (101 MHz, $CDCl_3$) δ 169.10, 159.84, 135.29, 134.77, 129.58, 127.99, 118.11, 113.44, 110.83, 104.69, 61.28, 55.65, 29.89. HRMS (m/z): Calcd for $C_{18}H_{20}N_3O_3$, $[M+H]^+$, 326.1499, found: 326.1498.

1-(1-(tert-butyl)-3-(4-fluorophenyl)-1H-pyrazol-5-yl)-1H-pyrrole-2,5-dione (2h)

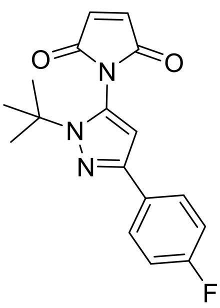

White solid (500 mg, 55% yield, Petroleum ether/EtOAc = 10/1 as the eluent), mp 166.1 - 166.9 °C. ^{1}H NMR (400 MHz, Chloroform-d) δ 7.77 – 7.72 (m, 2H), 7.09 – 7.04 (m, 2H), 6.94 (s, 2H), 6.42 (s, 1H), 1.58 (s, 9H). ^{13}C NMR (101 MHz, $CDCl_3$) δ 169.06, 162.6 (d, J=246.4 Hz), 148.01, 135.31, 129.63, 129.60, 128.10, 127.15, 127.07, 115.53, 115.32, 104.27, 61.29, 29.88. ^{19}F NMR (376 MHz, $CDCl_3$) δ -114.71. HRMS (m/z): Calcd for $C_{17}H_{17}FN_3O_2$, $[M+H]^+$, 314.1299, found: 314.1298.

1-(1-(tert-butyl)-3-(4-chlorophenyl)-1H-pyrazol-5-yl)-1H-pyrrole-2,5-dione (2i)

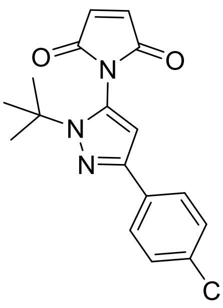

Orange solid (500 mg, 55% yield, Petroleum ether/EtOAc = 10/1 as the eluent), mp 155.1 - 155.9 °C. ^{1}H NMR (400 MHz, Chloroform-d) δ 7.72 (s, 2H), 7.35 (s, 2H), 6.93 (s, 2H), 6.44 (s, 1H), 1.58 (s, 9H). ^{13}C NMR (101 MHz, $CDCl_3$) δ 169.04, 147.76, 135.32, 133.39, 131.91, 128.71, 128.20, 126.70, 104.48, 61.43, 29.87. HRMS (m/z): Calcd for $C_{17}H_{17}ClN_3O_2$, $[M+H]^+$, 330.1004, found: 330.1003.

1-(3-(4-bromophenyl)-1-(tert-butyl)-1H-pyrazol-5-yl)-1H-pyrrole-2,5-dione (2j)

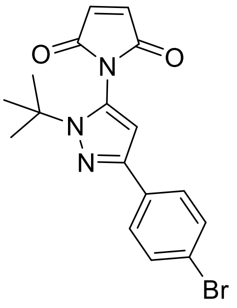

White solid (400 mg, 58% yield, Petroleum ether/EtOAc = 10/1 as the eluent), mp 164.1 - 164.9 °C. ^{1}H NMR (400 MHz, Chloroform-d) δ 7.67 (d, J = 8.6 Hz, 2H), 7.52 (d, J = 8.6 Hz, 2H), 6.97 (s, 2H), 6.47 (s, 1H), 1.60 (s, 9H). ^{13}C NMR (101 MHz, $CDCl_3$) δ 169.02, 147.77, 135.32, 132.35, 131.64, 128.20, 127.00, 121.59, 104.47, 61.45, 29.87. HRMS (m/z): Calcd for $C_{17}H_{17}BrN_3O_2$, $[M+H]^+$, 374.0499, found: 374.0496.

1-(1-(tert-butyl)-3-(p-tolyl)-1H-pyrazol-5-yl)-1H-pyrrole-2,5-dione (2k)

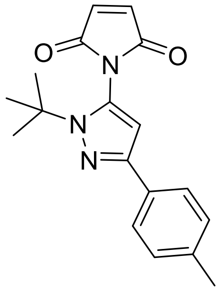

White solid (400 mg, 48% yield, Petroleum ether/EtOAc = 10/1 as the eluent), mp 176.1 - 176.9 °C. ^{1}H NMR (400 MHz, Chloroform-d) δ 7.65 (s, 2H), 7.17 (s, 2H), 6.91 (s, 2H), 6.42 (s, 1H), 2.36 (s, 3H), 1.55 (s, 9H). ^{13}C NMR (101 MHz, $CDCl_3$) δ 169.14, 148.95, 137.38, 135.28, 130.63, 129.22, 127.85, 125.36, 104.27, 61.12, 29.90. HRMS (m/z): Calcd for $C_{18}H_{20}N_3O_2$, $[M+H]^+$, 310.1550, found: 310.1551.

Catalyst **C1-C10** were synthesized according to the literature procedure. The racemic products were synthesized using quinine/quinidine = 1:1 as the catalyst.

4. Experimental procedures and characterization of products 3 and 4

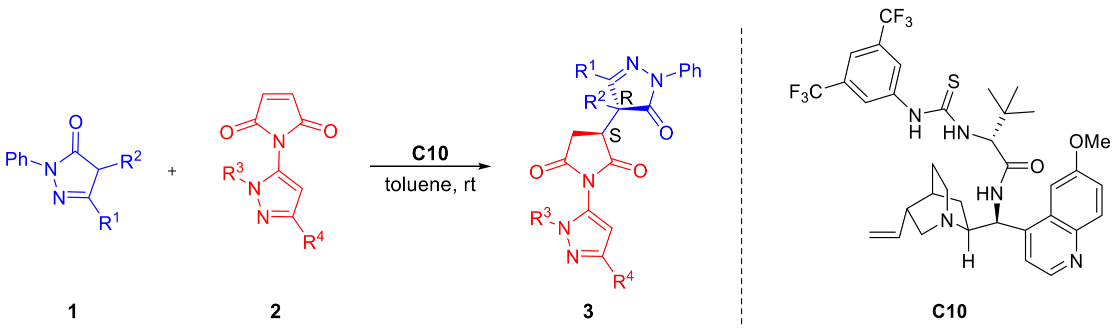

In a reaction tube, pyrazol-5-ones **1** (0.24 mmol), pyrazol-maleimide **2** (0.20 mmol), catalyst **C10** (0.02 mmol) were added into toluene (4 mL). The reaction solution was stirred at 25 °C. After the reaction was complete (monitored by TLC), the crude product was purified by column chromatography (ethyl acetate/petroleum ether = 1/10 to 1/3) on silica gel to give the product **3**.

(S)-3-((R)-4-benzyl-5-oxo-1,3-diphenyl-4,5-dihydro-1H-pyrazol-4-yl)-1-(1-(tert-butyl)-3-phenyl-1H-pyrazol-5-yl)pyrrolidine-2,5-dione (3aa)

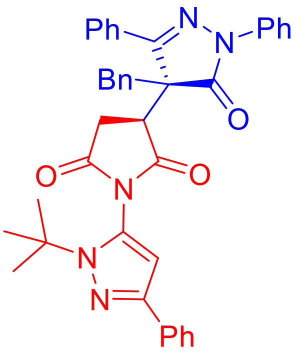

3aa

Prepared according to the procedure within 1 h as white solid (121.8 mg, 98% yield, dr = 1:1). mp 127.1 – 127.9 °C; $[\alpha]_D^{17}$ = -33.206 (*c* 0.52, CH_2Cl_2); ^{1}H NMR (400 MHz, Chloroform-d) δ 8.00 (dq, J = 6.7, 2.6, 1.6 Hz, 4H), 7.69 – 7.48 (m, 15H), 7.40 – 7.28 (m, 9H), 7.23 – 7.16 (m, 2H), 7.12 – 7.01 (m, 10H), 6.29 (s, 1H), 5.50 (s, 1H), 4.22 (dd, J = 17.6, 7.4 Hz, 2H), 3.99 (dd, J = 9.3, 7.4 Hz, 1H), 3.76 (dd, J = 9.4, 5.5 Hz, 1H), 3.61 (dd, J = 19.5, 13.5 Hz, 2H), 3.50 (d, J = 13.2 Hz, 1H), 3.18 (dd, J = 17.8, 9.4 Hz, 1H), 3.01 (dd, J = 18.6, 9.7 Hz, 1H), 2.78 (s, 1H), 1.53 (s, 9H), 1.38 (s, 9H). ^{13}C NMR (151 MHz, $CDCl_3$) δ 174.89, 173.87, 173.72, 173.43, 173.07, 158.07, 157.37, 148.93, 148.66, 137.00, 136.82, 133.31, 133.24, 132.50, 131.10, 131.02, 130.99, 130.71, 129.42, 129.36, 129.35, 129.17, 128.99, 128.79, 128.71, 128.49, 128.47, 128.44, 128.39, 127.95, 127.79, 127.69, 127.61, 127.00, 126.29, 126.05, 125.40, 125.34, 120.04, 119.89, 103.68, 61.50, 57.13, 44.93, 43.58, 41.24, 40.02, 31.06, 30.04, 29.81, 29.69. HRMS (ESI) m/z Calcd. for $C_{39}H_{36}N_5O_3$, $[M+H]^+$, 622.2813, Found: 622.2806. Enantiomeric excess was determined to be 99% (determined by HPLC using chiral IB-H column, hexane/2-propanol = 7/3, λ = 254 nm, 25 °C, 0.8 mL/min, t_{major} = 21.9 min, t_{minor} = 16.6 min).

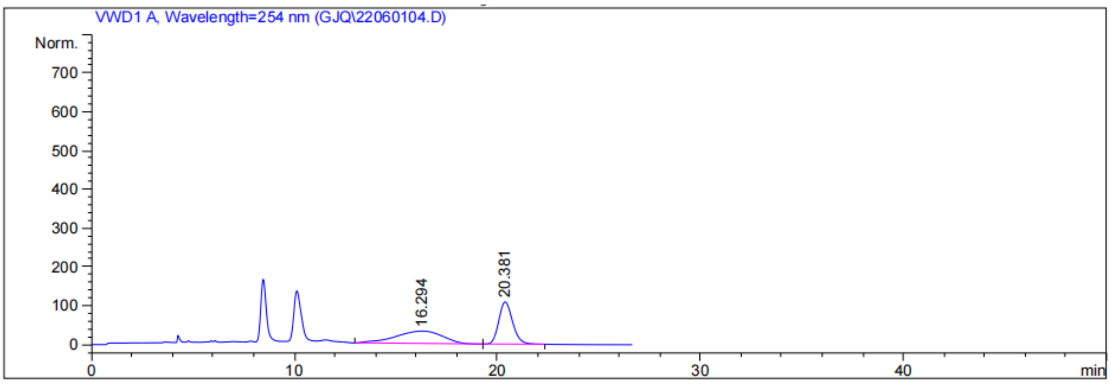

Signal 1: VWD1 A, Wavelength=254 nm

Peak #	RetTime [min]	Type	Width [min]	Area mAU *s	Height [mAU]	Area %
1	16.294	BP	2.2224	5175.55811	32.16374	50.0676
2	20.381	VB	0.7344	5161.57666	108.73219	49.9324

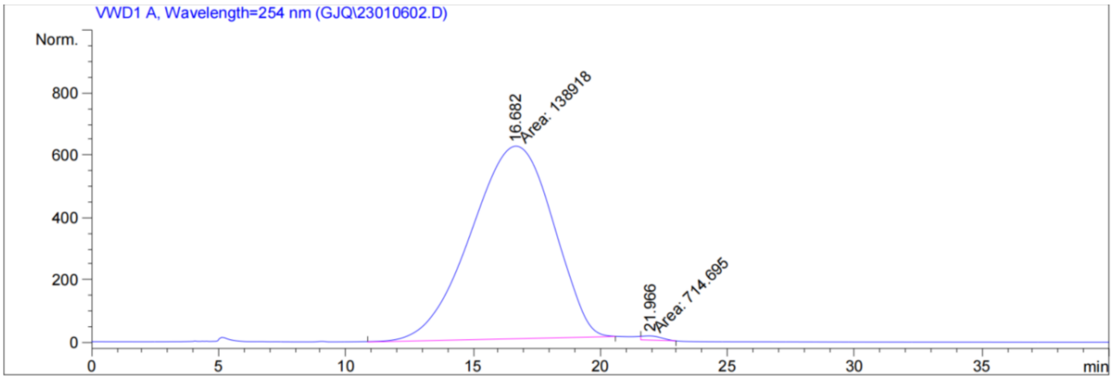

Signal 1: VWD1 A, Wavelength=254 nm

Peak #	RetTime [min]	Type	Width [min]	Area mAU *s	Height [mAU]	Area %
1	16.682	MM	3.7494	1.38918e5	617.51477	99.4882
2	21.966	MM	0.8841	714.69501	13.47279	0.5118

(S)-3-((R)-4-benzyl-5-oxo-1-phenyl-3-(o-tolyl)-4,5-dihydro-1H-pyrazol-4-yl)-1-(1-(tert-butyl)-3-phenyl-1H-pyrazol-5-yl)pyrrolidine-2,5-dione (3ba)

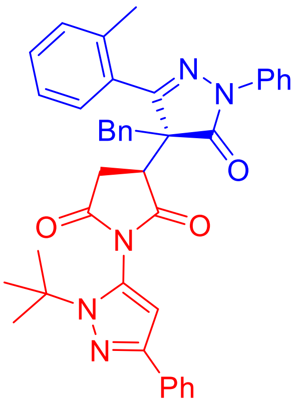

3ba

Prepared according to the procedure within 1.2 h as white solid (125.8 mg, 99% yield, dr = 1:1). mp 108.1 – 108.9 °C; $[\alpha]_D^{17}$ = -35.030 (*c* 0.33, CH_2Cl_2); ^{1}H NMR (400 MHz, Chloroform-d) δ 7.83 (d, J = 11.5 Hz, 2H), 7.77 (q, J = 3.5 Hz, 2H), 7.69 – 7.61 (m, 6H), 7.58 (d, J = 8.0 Hz, 2H), 7.46 – 7.41 (m, 2H), 7.41 – 7.26 (m, 12H), 7.22 – 7.15 (m, 2H), 7.08 (d, J = 6.9 Hz, 8H), 7.03 (dd, J = 7.8, 1.8 Hz, 2H), 6.29 (s, 1H), 5.51 (s, 1H), 4.31 – 4.13 (m, 2H), 3.98 (dd, J = 9.3, 7.4 Hz, 1H), 3.75 (dd, J = 9.4, 5.4 Hz, 1H), 3.60 (dd, J = 26.7, 13.5 Hz, 2H), 3.48 (d, J = 13.2 Hz, 1H), 3.18 (dd, J = 17.8, 9.4 Hz, 1H), 2.99 (dd, J = 18.6, 9.7 Hz, 1H), 2.66 (d, J = 58.4 Hz, 1H), 2.43 (s, 6H), 1.38 (s, 9H). ^{13}C NMR (101 MHz, $CDCl_3$) δ 173.89, 173.75, 173.46, 173.08, 158.23, 157.50, 148.90, 148.64, 139.37, 139.09, 137.00, 136.83, 133.39, 133.33, 133.27, 132.55, 131.90, 131.63, 130.98, 130.89, 129.39, 129.26, 128.97, 128.88, 128.81, 128.70, 128.50, 128.46, 128.41, 128.37, 128.17, 127.93, 127.75, 127.68, 127.60, 126.27, 126.03, 125.39, 125.26, 124.66, 123.88, 120.09, 119.94, 103.70, 103.63, 61.50, 57.14, 44.95, 43.55, 41.28, 40.04, 31.10, 30.07, 29.81, 29.70, 21.68, 21.65. HRMS (ESI) m/z Calcd. for $C_{40}H_{38}N_5O_3$, $[M+H]^+$, 636.2969, Found: 636.2972. Enantiomeric excess was determined to be 98% (determined by HPLC using chiral OD-H column, hexane/2-propanol = 7/3, λ = 254 nm, 25 °C, 0.8 mL/min, t_{major} = 25.1 min, t_{minor} = 12.1 min).

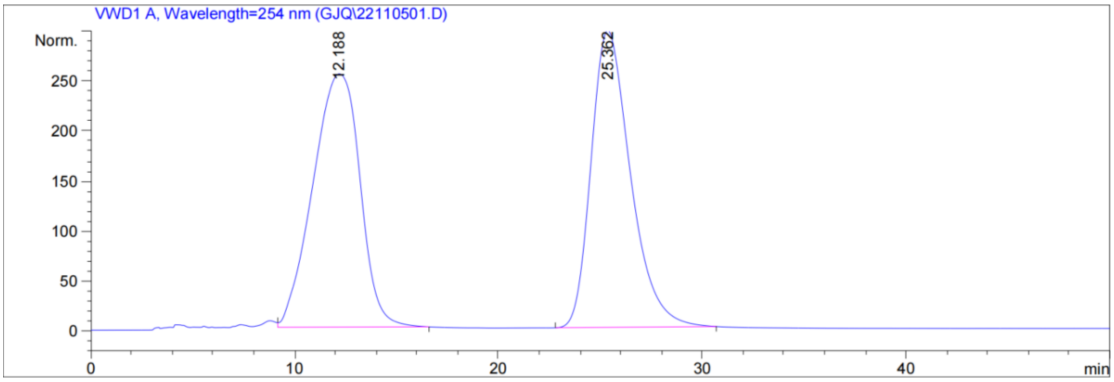

Peak #	RetTime [min]	Type	Width [min]	Area mAU *s	Height [mAU]	Area %
1	12.188	VB	2.4728	4.08427e4	253.82530	50.5509
2	25.362	BB	2.0536	3.99524e4	297.39368	49.4491

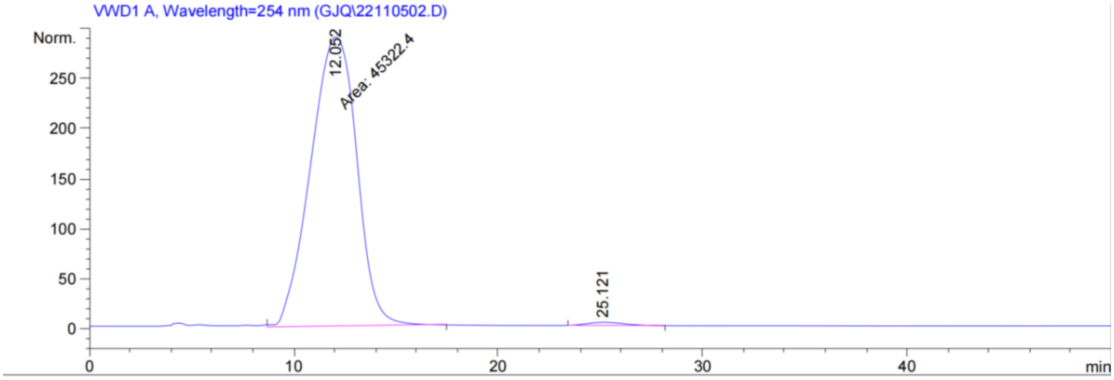

Peak #	RetTime [min]	Type	Width [min]	Area mAU *s	Height [mAU]	Area %
1	12.052	MM	2.6128	4.53224e4	289.10687	99.1233
2	25.121	BB	1.4906	400.85406	3.16214	0.8767

(S)-3-((R)-4-benzyl-5-oxo-1-phenyl-3-(m-tolyl)-4,5-dihydro-1H-pyrazol-4-yl)-1-(1-(tert-butyl)-3-phenyl-1H-pyrazol-5-yl)pyrrolidine-2,5-dione (3ca)

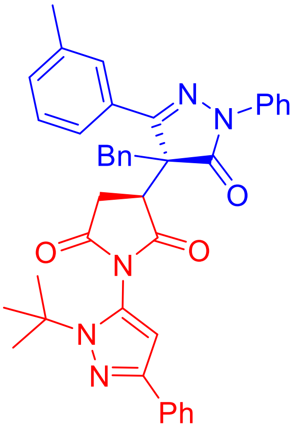

3ca

Prepared according to the procedure within 1.2 h as white solid (125.8 mg, 99% yield, dr = 1:1). mp 109.1 – 109.9 °C; $[\alpha]_D^{17}$ = -33.491 (*c* 0.42, CH_2Cl_2); ^{1}H NMR (400 MHz, Chloroform-d) δ 7.85 (s, 1H), 7.82 (s, 1H), 7.78 (d, J = 7.2 Hz, 2H), 7.68 (d, J = 1.5 Hz, 1H), 7.67 – 7.61 (m, 5H), 7.60 – 7.55 (m, 2H), 7.43 (dd, J = 12.2, 6.9 Hz, 3H), 7.40 – 7.27 (m, 11H), 7.23 – 7.16 (m, 2H), 7.09 (d, J = 5.6 Hz, 8H), 7.04 (dd, J = 7.7, 1.9 Hz, 2H), 6.29 (d, J = 1.0 Hz, 1H), 5.50 (s, 1H), 4.31 – 4.15 (m, 2H), 3.99 (dd, J = 9.3, 7.5 Hz, 1H), 3.75 (dd, J = 9.5, 5.4 Hz, 1H), 3.65 (d, J = 13.2 Hz, 1H), 3.57 (d, J = 13.8 Hz, 1H), 3.49 (d, J = 13.2 Hz, 1H), 3.19 (dd, J = 17.8, 9.4 Hz, 1H), 3.00 (dd, J = 18.6, 9.7 Hz, 1H), 2.66 (d, J = 49.9 Hz, 1H), 2.44 (s, 6H), 1.54 (s, 9H), 1.38 (d, J = 1.0 Hz, 9H). ^{13}C NMR (101 MHz, $CDCl_3$) δ 174.94, 173.90, 173.76, 173.46, 173.11, 173.08, 158.23, 157.51, 148.90, 148.65, 139.37, 139.09, 137.00, 136.82, 133.39, 133.33, 133.26, 132.55, 131.90, 131.63, 130.98, 130.89, 129.39, 129.26, 128.97, 128.88, 128.81, 128.70, 128.50, 128.47, 128.41, 128.37, 128.17, 127.93, 127.75, 127.69, 127.61, 126.28, 126.04, 125.40, 125.27, 124.66, 123.88, 120.09, 119.94, 103.70, 103.63, 61.50, 57.14, 44.95, 43.55, 41.28, 40.04, 31.10, 30.07, 29.81, 29.70, 21.65. HRMS (ESI) m/z Calcd. for $C_{40}H_{38}N_5O_3$, $[M+H]^+$, 636.2969, Found: 636.2974. Enantiomeric excess was determined to be 99% (determined by HPLC using chiral OD-H column, hexane/2-propanol = 7/3, λ = 254 nm, 25 °C, 0.8 mL/min, t_{major} = 22.5 min, t_{minor} = 12.1 min).

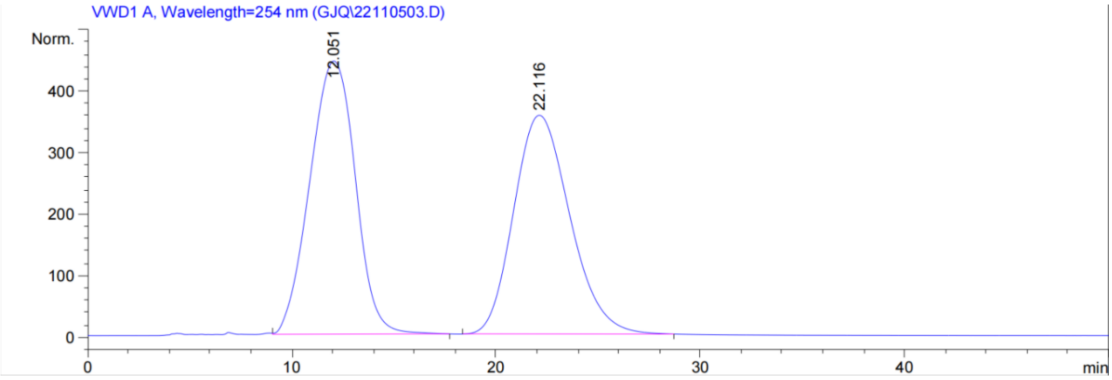

Peak #	RetTime [min]	Type	Width [min]	Area mAU *s	Height [mAU]	Area %
1	12.051	VB	2.4691	6.89116e4	444.30835	50.2770
2	22.116	BB	2.9303	6.81522e4	355.85284	49.7230

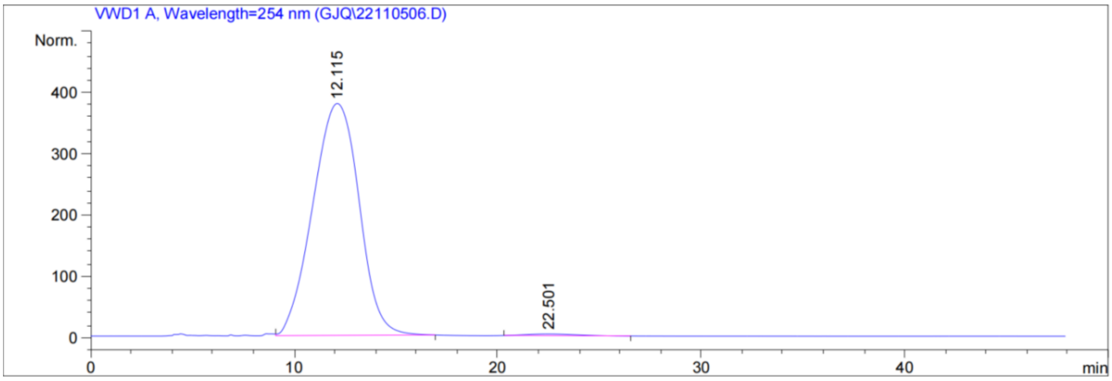

Peak #	RetTime [min]	Type	Width [min]	Area mAU *s	Height [mAU]	Area %
1	12.115	VB	2.4983	5.99550e4	378.61520	99.2216
2	22.501	BB	2.0237	470.34357	2.72523	0.7784

(S)-3-((R)-4-benzyl-5-oxo-1-phenyl-3-(p-tolyl)-4,5-dihydro-1H-pyrazol-4-yl)-1-(1-(tert-butyl)-3-phenyl-1H-pyrazol-5-yl)pyrrolidine-2,5-dione (3da)

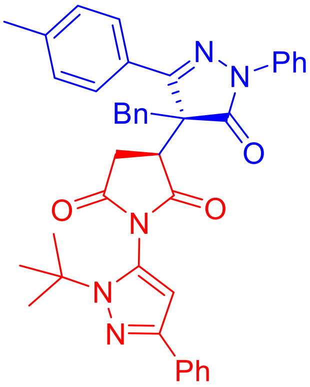

3da

Prepared according to the procedure within 1.2 h as white solid (125.8 mg, 99% yield, dr = 1:1). mp 127.1 – 127.9 °C; $[\alpha]_D^{17}$ = -61.572 (*c* 0.23, CH_2Cl_2); ^1H NMR (400 MHz, Chloroform-d) δ 7.89 (dd, J = 8.0 Hz, 4H), 7.68 – 7.56 (m, 9H), 7.39 – 7.28 (m, 14H), 7.19 (dd, J = 16.7, 8.2 Hz, 2H), 7.09 (d, J = 6.7 Hz, 8H), 7.06 – 7.01 (m, 2H), 6.28 (s, 1H), 5.48 (s, 1H), 4.20 (d, J = 26.5 Hz, 2H), 3.97 (dd, J = 9.3, 7.4 Hz, 1H), 3.74 (dd, J = 9.7, 5.2 Hz, 1H), 3.61 (dd, J = 27.2, 13.5 Hz, 2H), 3.49 (d, J = 13.2 Hz, 1H), 3.17 (dd, J = 17.8, 9.4 Hz, 1H), 2.99 (dd, J = 18.6, 9.7 Hz, 1H), 2.63 (d, J = 27.3 Hz, 1H), 2.50 (s, 3H), 2.44 (s, 3H), 1.38 (s, 9H). ^{13}C NMR (101 MHz, $CDCl_3$) δ 174.98, 173.84, 173.76, 173.38, 173.08, 173.01, 158.07, 157.37, 148.92, 148.63, 141.58, 141.11, 137.03, 136.86, 133.46, 133.33, 133.26, 132.59, 130.09, 129.89, 129.37, 128.95, 128.80, 128.67, 128.48, 128.45, 128.40, 128.35, 128.32, 128.20, 127.89, 127.71, 127.58, 126.90, 126.21, 125.96, 125.38, 125.36, 120.04, 119.87, 103.68, 103.66, 61.51, 61.48, 57.15, 44.97, 43.50, 41.21, 39.88, 31.12, 30.07, 29.80, 29.67, 21.62, 21.55. HRMS (ESI) m/z Calcd. for $C_{40}H_{38}N_5O_3$, $[M+H]^+$, 636.2969, Found: 636.2979. Enantiomeric excess was determined to be 98% (determined by HPLC using chiral OD-H column, hexane/2-propanol = 7/3, λ = 254 nm, 25 °C, 0.8 mL/min, t_{major} = 25.3 min, t_{minor} = 12.1 min).

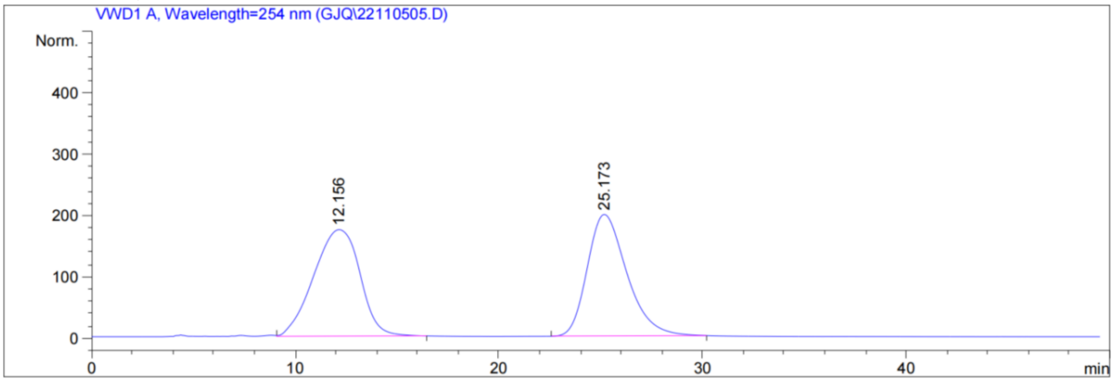

Peak #	RetTime [min]	Type	Width [min]	Area mAU *s	Height [mAU]	Area %
1	12.156	VB	2.4071	2.74575e4	173.61469	50.5762
2	25.173	BB	2.0362	2.68319e4	198.25240	49.4238

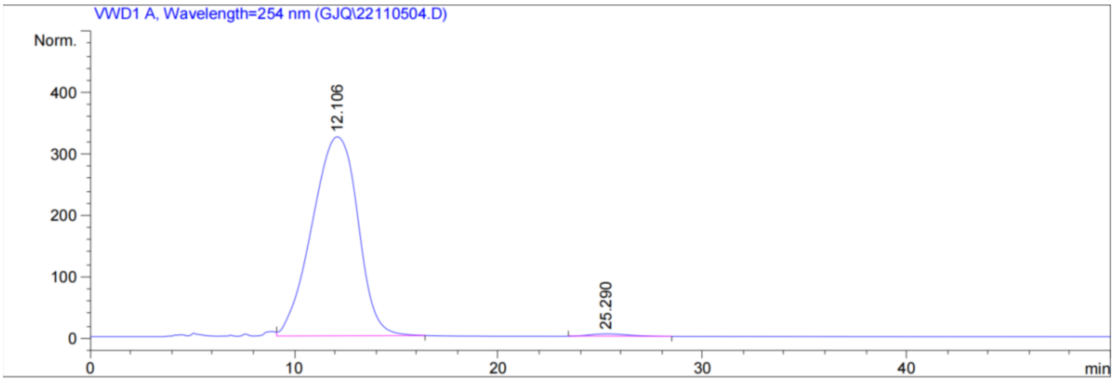

Peak #	RetTime [min]	Type	Width [min]	Area mAU *s	Height [mAU]	Area %
1	12.106	VB	2.4667	5.06706e4	324.25516	99.0246
2	25.290	BB	1.5296	499.11697	3.85080	0.9754

(S)-3-((R)-4-benzyl-3-(naphthalen-2-yl)-5-oxo-1-phenyl-4,5-dihydro-1H-pyrazol-4-yl)-1-(1-(tert-butyl)-3-phenyl-1H-pyrazol-5-yl)pyrrolidine-2,5-dione (3ea)

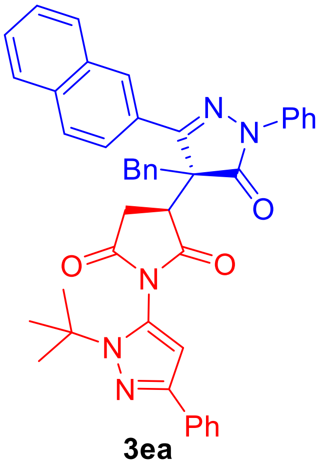

3ea

Prepared according to the procedure within 2.5 h as white solid (106.1mg, 79% yield, dr = 1:1). mp 110.1 – 110.9 °C; $[\alpha]_D^{17}$ = -46.491 (c 0.79, CH_2Cl_2); ^{1}H NMR (400 MHz, Chloroform-d) δ 8.42 (d, J = 2.3 Hz, 1H), 8.39 (s, 1H), 8.20 – 8.12 (m, 3H), 8.00 – 7.92 (m, 7H), 7.91 – 7.85 (m, 1H), 7.72 – 7.61 (m, 9H), 7.61 – 7.51 (m, 4H), 7.37 (dt, J = 15.5, 7.6 Hz, 5H), 7.32 – 7.17 (m, 15H), 7.15 – 7.01 (m, 13H), 6.26 (d, J = 2.0 Hz, 1H), 4.95 (s, 1H), 4.47 (s, 1H), 4.20 (dd, J = 10.1, 5.1 Hz, 1H), 4.11 (t, J = 8.2 Hz, 1H), 3.81 (q, J = 6.2, 5.6 Hz, 2H), 3.66 (dd, J = 13.9, 1.6 Hz, 1H), 3.56 (d, J = 13.2 Hz, 1H), 3.22 (dd, J = 17.6, 9.1 Hz, 1H), 3.00 (dd, J = 18.7, 9.8 Hz, 1H), 2.47 (s, 1H), 1.52 (d, J = 1.2 Hz, 9H), 1.35 (d, J = 2.1 Hz, 9H). ^{13}C NMR (151 MHz, CDCl3) δ 175.41, 173.92, 173.74, 173.51, 173.14, 173.03, 157.91, 157.12, 148.68, 137.00, 136.82, 134.37, 134.28, 133.50, 133.29, 132.95, 132.92, 132.85, 132.56, 129.43, 129.39, 129.29, 129.23, 129.04, 128.77, 128.71, 128.46, 128.43, 128.40, 128.23, 128.15, 128.04, 128.00, 127.97, 127.76, 127.67, 127.60, 127.48, 127.05, 126.56, 126.40, 126.16, 125.38, 125.28, 124.13, 124.04, 120.13, 119.97, 103.66, 103.58, 61.49, 57.31, 45.19, 43.54, 41.45, 40.11, 31.21, 30.12, 29.82, 29.65. HRMS (ESI) m/z Calcd. for $C_{43}H_{38}N_5O_3$, $[M+H]^+$, 672.2969, Found: 672.2976. Enantiomeric excess was determined to be 98% (determined by HPLC using chiral OD-H-AD-H column, hexane/2-propanol = 7/3, λ = 254 nm, 25 °C, 0.6 mL/min, t_{major} = 61.0 min, t_{minor} = 34.0 min).

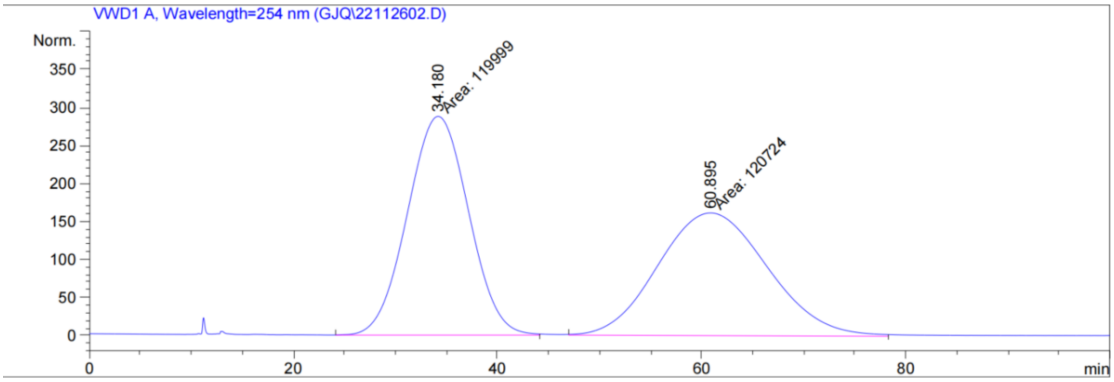

Peak #	RetTime [min]	Type	Width [min]	Area mAU *s	Height [mAU]	Area %
1	34.180	MM	6.9493	1.19999e5	287.79471	49.8494
2	60.895	MM	12.4691	1.20724e5	161.36330	50.1506

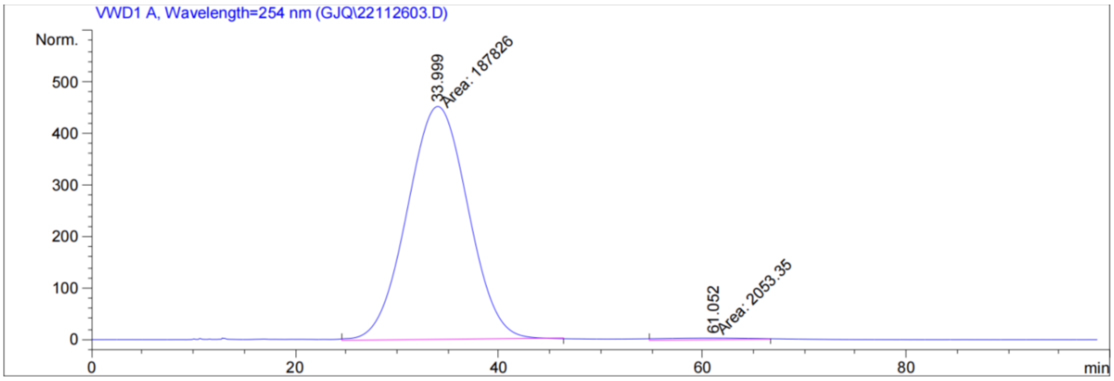

Peak #	RetTime [min]	Type	Width [min]	Area mAU *s	Height [mAU]	Area %
1	33.999	MM	4.9622	1.87826e5	452.31607	98.9186
2	61.052	MM	10.5063	2053.35425	3.25733	1.0814

(S)-3-((R)-4-benzyl-5-oxo-1-phenyl-3-(thiophen-2-yl)-4,5-dihydro-1H-pyrazol-4-yl)-1-(1-(tert-butyl)-3-phenyl-1H-pyrazol-5-yl)pyrrolidine-2,5-dione (3fa)

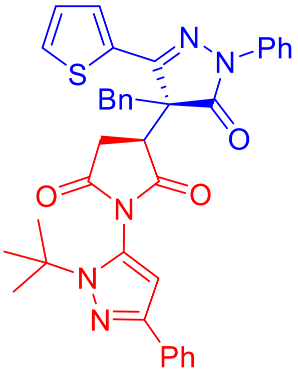

3fa

Prepared according to the procedure within 1.1 h as white solid (124.2 mg, 99% yield, dr = 1:1). mp 120.1 – 120.9 °C; $[\alpha]_D^{17}$ = -35.474 (*c* 0.65, CH_2Cl_2); ^{1}H NMR (400 MHz, Chloroform-d) δ 7.64 (ddd, J = 19.8, 10.9, 7.0 Hz, 9H), 7.53 (dd, J = 10.3, 6.5 Hz, 3H), 7.39 – 7.28 (m, 9H), 7.25 – 7.17 (m, 4H), 7.16 – 7.00 (m, 11H), 6.33 (s, 1H), 5.80 (s, 1H), 4.09 (d, J = 28.1 Hz, 2H), 4.00 – 3.94 (m, 1H), 3.77 (dd, J = 9.4, 5.6 Hz, 1H), 3.59 – 3.51 (m, 2H), 3.47 (d, J = 13.1 Hz, 1H), 3.19 (dd, J = 17.8, 9.3 Hz, 1H), 3.03 (dd, J = 18.5, 9.6 Hz, 1H), 2.79 (s, 1H), 1.57 (s, 9H), 1.38 (s, 9H). ^{13}C NMR (101 MHz, $CDCl_3$) δ 173.69, 173.56, 172.98, 172.69, 172.39, 154.24, 149.01, 148.68, 136.79, 136.61, 134.42, 134.31, 133.30, 133.21, 132.36, 129.63, 129.46, 129.40, 128.96, 128.92, 128.70, 128.50, 128.46, 128.44, 128.24, 127.97, 127.91, 127.78, 127.72, 127.61, 127.39, 126.33, 126.10, 125.39, 125.35, 120.08, 119.96, 103.67, 61.59, 61.51, 45.35, 41.07, 39.72, 31.05, 30.11, 29.83, 29.65. HRMS (ESI) m/z Calcd. for $C_{37}H_{34}N_5O_3S$, $[M+H]^+$, 628.2377, Found:

628.2387. Enantiomeric excess was determined to be 99% (determined by HPLC using chiral OD-H column, hexane/2-propanol = 8/2, λ = 254 nm, 25 °C, 0.8 mL/min, t_{major} = 32.4 min, t_{minor} = 21.3 min).

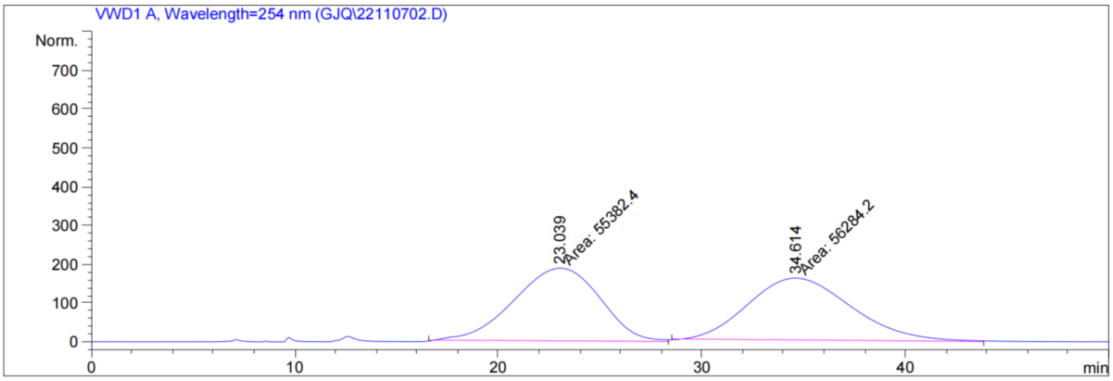

Peak #	RetTime [min]	Type	Width [min]	Area mAU *s	Height [mAU]	Area %
1	23.039	MM	4.9139	5.53824e4	187.84431	49.5962
2	34.614	MM	5.8841	5.62842e4	159.42593	50.4038

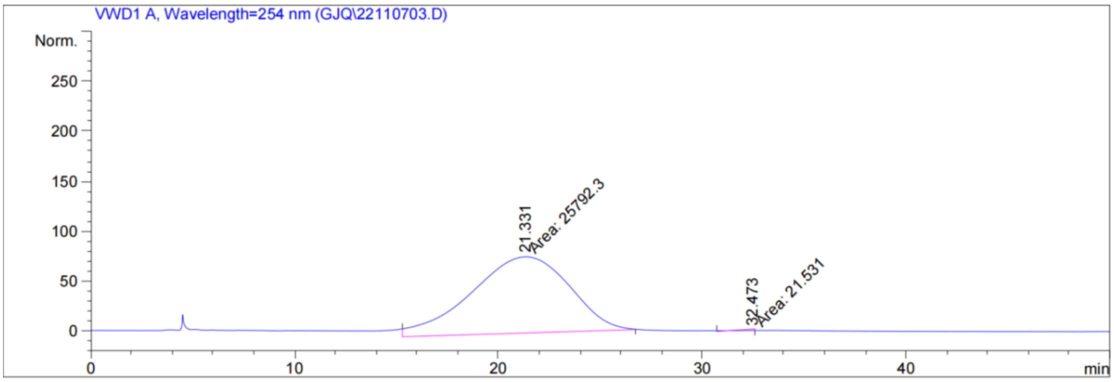

Peak #	RetTime [min]	Type	Width [min]	Area mAU *s	Height [mAU]	Area %
1	21.331	MM	5.6201	2.57923e4	76.48786	99.9166
2	32.473	MM	0.0000	21.53103	1.33860	0.0834

(S)-3-((R)-4-benzyl-3-methyl-5-oxo-1-phenyl-4,5-dihydro-1H-pyrazol-4-yl)-1-(1-(tert-butyl)-3-phenyl-1H-pyrazol-5-yl)pyrrolidine-2,5-dione (3ga)

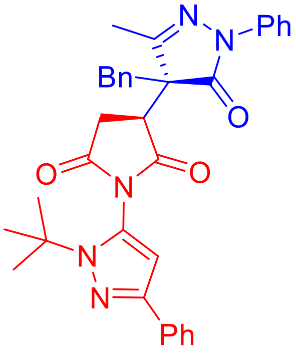

3ga

Prepared according to the procedure within 1.1 h as white solid (108.5mg, 97% yield, dr = 1:1). mp 124.1 – 124.9 °C; $[\alpha]_D^{17}$ = 80.357 (*c* 0.45, CH_2Cl_2); ^{1}H NMR (400 MHz, Chloroform-d) δ 7.75 – 7.70 (m, 2H), 7.66 – 7.61 (m, 2H), 7.61 – 7.56 (m, 2H), 7.53 – 7.48 (m, 2H), 7.38 – 7.26 (m, 10H), 7.21 – 7.08 (m, 12H), 6.38 (s, 1H), 6.18 (s, 1H), 4.17 (dd, J = 18.1, 6.8 Hz, 1H), 3.56 – 3.35 (m, 4H), 3.28 (t, J = 14.2 Hz, 2H), 3.13 – 3.03 (m, 2H), 3.02 – 2.96 (m, 1H), 2.28 (s, 3H), 2.26 (s, 3H), 1.58 (s, 7H), 1.38 (s, 9H). ^{13}C NMR (101 MHz, $CDCl_3$) δ 174.51, 174.00, 173.76, 173.29, 172.63, 172.49, 160.48, 159.29, 149.12, 148.67, 137.04, 136.92, 133.30, 133.14, 133.09, 132.60,

129.20, 129.13, 128.93, 128.91, 128.85, 128.74, 128.70, 128.66, 128.61, 128.53, 128.47, 128.02, 127.92, 127.75, 127.69, 127.25, 125.91, 125.70, 125.43, 125.41, 125.12, 119.63, 119.07, 103.61, 103.51, 61.59, 61.52, 59.86, 57.34, 53.71, 44.04, 43.24, 40.46, 39.44, 33.77, 30.78, 29.87, 29.68, 29.61, 15.32, 14.71. HRMS (ESI) m/z Calcd. for $C_{34}H_{34}N_5O_3$, $[M+H]^+$, 560.2656, Found: 560.2659. Enantiomeric excess was determined to be 99% (determined by HPLC using chiral IA-H-OD-H column, hexane/2-propanol = 8/2, λ = 254 nm, 25 °C, 0.6 mL/min, t_{major} = 113.3 min, t_{minor} = 71.1 min).

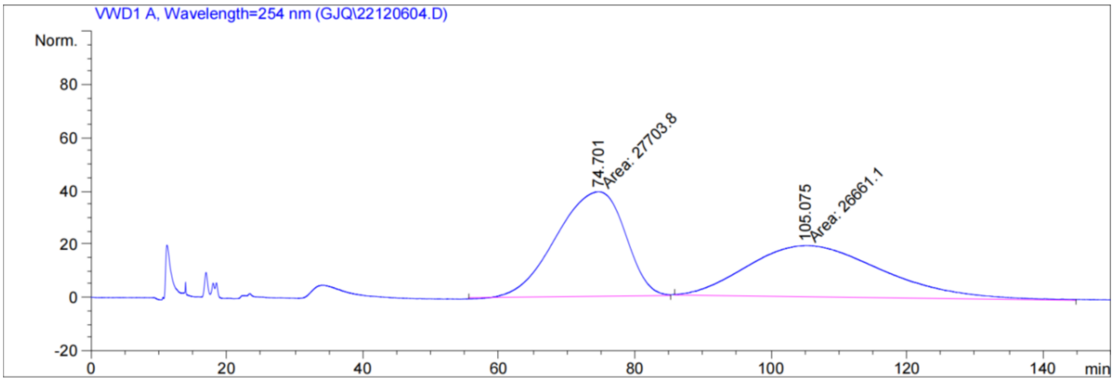

Peak #	RetTime [min]	Type	Width [min]	Area mAU *s	Height [mAU]	Area %
1	74.701	MM	11.7340	2.77038e4	39.34961	50.9590
2	105.075	MM	23.0530	2.66611e4	19.27520	49.0410

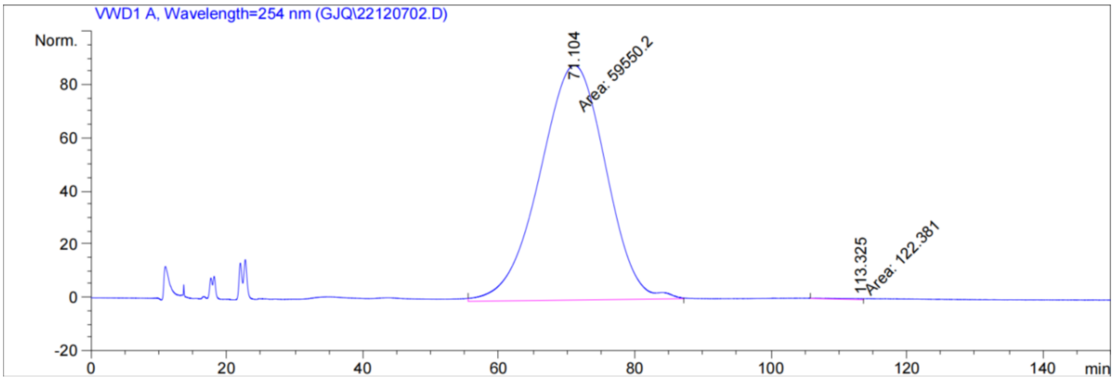

Peak #	RetTime [min]	Type	Width [min]	Area mAU *s	Height [mAU]	Area %
1	71.104	MM	11.2414	5.95502e4	88.28976	99.7949
2	113.325	MM	5.2524	122.38092	3.88330e-1	0.2051

(S)-1-(1-(tert-butyl)-3-phenyl-1H-pyrazol-5-yl)-3-((R)-4-(2-fluorobenzyl)-5-oxo-1,3-diphenyl-4,5-dihydro-1H-pyrazol-4-yl)pyrrolidine-2,5-dione (3ha)

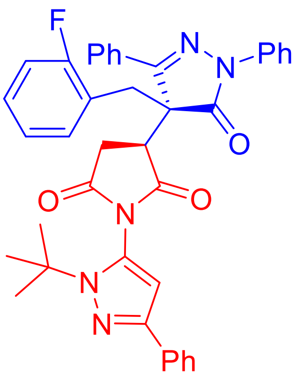

3ha

Prepared according to the procedure within 1.5 h as white solid (126.6 mg, 99% yield, dr = 1:1). mp 113.1 – 113.9 °C; $[\alpha]_D^{17}$ = -42.005 (c 0.89, CH_2Cl_2); ^{1}H NMR (400 MHz, Chloroform-d) δ 7.99 – 7.93 (m, 4H), 7.76 – 7.71 (m, 2H), 7.67 (ddd, J = 8.1, 3.3, 1.2 Hz, 4H), 7.64 – 7.55 (m, 4H), 7.55 – 7.44 (m, 5H), 7.42 – 7.28 (m, 9H), 7.23 – 7.03 (m, 6H), 6.92 – 6.81 (m, 4H), 6.28 (s, 1H), 5.54 (s, 1H), 4.36 – 4.14 (m, 2H), 4.02 (dd, J = 9.3, 7.4 Hz, 1H), 3.81 – 3.71 (m, 2H), 3.66 (d, J = 14.2 Hz, 1H), 3.52 (d, J = 13.7 Hz, 1H), 3.18 (dd, J = 17.8, 9.4 Hz, 1H), 2.99 (dd, J = 18.5, 9.7 Hz, 1H), 2.82 (s, 1H), 1.52 (s, 9H), 1.38 (s, 9H). ^{13}C NMR (151 MHz, $CDCl_3$) δ 174.85, 173.83, 173.72, 173.42, 173.01, 172.89, 161.74 (d, J= 8.08 Hz), 160.14, 160.06, 158.39, 157.77, 148.94, 148.63, 137.06, 136.88, 133.29, 133.23, 131.29, 131.26, 131.20, 131.18, 131.02, 130.71, 130.64, 129.88, 129.83, 129.73, 129.67, 129.54, 129.28, 129.22, 129.08, 129.05, 128.93, 128.79, 128.50, 128.47, 128.40, 127.74, 127.69, 127.62, 127.09, 126.89, 126.27, 126.04, 125.39, 125.35, 124.15, 124.12, 124.08, 120.70, 120.60, 120.03, 119.93, 119.84, 119.78, 119.70, 115.69, 115.67, 115.53, 103.71, 61.50, 56.25, 49.45, 45.07, 44.90, 43.50, 33.77, 32.63, 31.04, 30.09, 29.81, 29.75, 29.69, 29.67, 17.67. ^{19}F NMR (376 MHz, $CDCl_3$) δ -113.91, -114.43. HRMS (ESI) m/z Calcd. for $C_{39}H_{35}FN_5O_3$, $[M+H]^+$, 640.2718, Found: 640.2726. Enantiomeric excess was determined to be 94% (determined by HPLC using chiral OD-H column, hexane/2-propanol = 9/1, λ = 254 nm, 25 °C, 0.8 mL/min, t_{major} = 86.7 min, t_{minor} = 34.5 min).

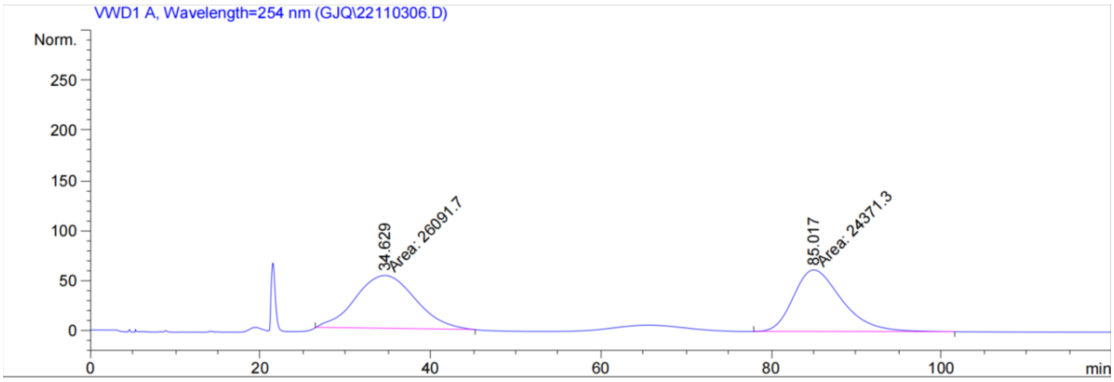

Peak #	RetTime [min]	Type	Width [min]	Area mAU *s	Height [mAU]	Area %
1	34.629	MM T	8.2096	2.60917e4	52.96978	51.7046
2	85.017	MM T	6.5931	2.43713e4	61.60832	48.2954

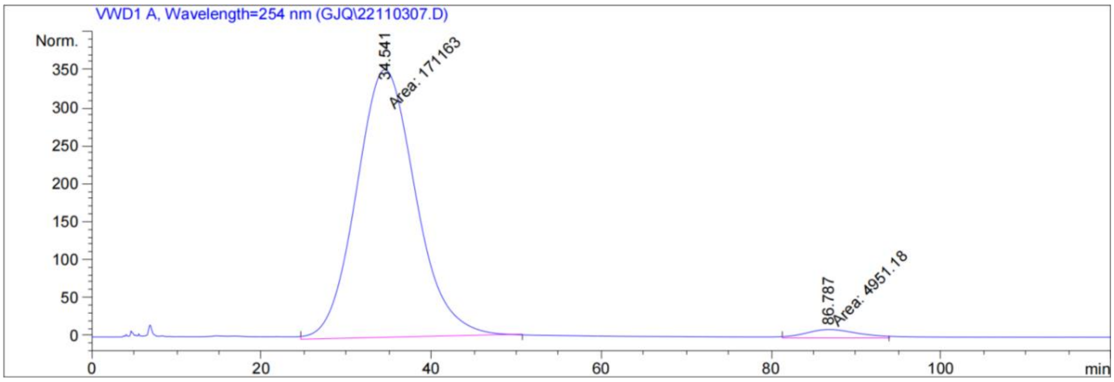

Peak #	RetTime [min]	Type	Width [min]	Area mAU *s	Height [mAU]	Area %
1	34.541	MM	8.0945	1.71163e5	352.42606	97.1887
2	86.787	MM T	7.3152	4951.17969	11.28065	2.8113

(S)-3-((R)-4-(2-bromobenzyl)-5-oxo-1,3-diphenyl-4,5-dihydro-1H-pyrazol-4-yl)-1-(1-(tert-butyl)-3-phenyl-1H-pyrazol-5-yl)pyrrolidine-2,5-dione (3ia)

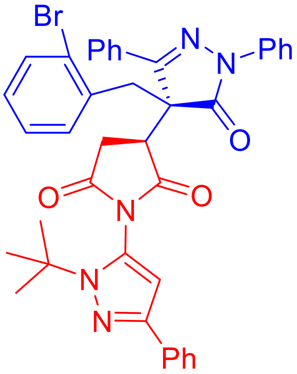

3ia

Prepared according to the procedure within 2 h as white solid (137.1 mg, 98% yield, dr = 1:1). mp 125.1 – 125.9 °C; $[\alpha]_D^{17}$ = -7.206 (*c* 0.68, CH_2Cl_2); ^{1}H NMR (400 MHz, Chloroform-d) δ 7.99 – 7.95 (m, 2H), 7.93 – 7.89 (m, 2H), 7.87 – 7.82 (m, 2H), 7.78 – 7.73 (m, 2H), 7.69 – 7.65 (m, 2H), 7.63 – 7.58 (m, 3H), 7.55 – 7.41 (m, 10H), 7.40 – 7.35 (m, 3H), 7.34 – 7.30 (m, 2H), 7.29 – 7.26 (m, 2H), 7.23 – 7.19 (m, 2H), 7.07 (ddtd, J = 28.0, 14.7, 7.3, 1.8 Hz, 6H), 6.29 (d, J = 2.9 Hz, 1H), 5.37 (s, 1H), 4.26 (d, J = 7.9 Hz, 1H), 4.21 – 4.03 (m, 3H), 3.89 (d, J = 14.4 Hz, 1H), 3.77 (q, J = 7.1 Hz, 2H), 3.17 (dd, J = 17.5, 9.0 Hz, 1H), 2.97 (dd, J = 18.6, 9.6 Hz, 1H), 2.72 (s, 1H), 1.53 (s, 9H), 1.33 (s, 9H). ^{13}C NMR (151 MHz, $CDCl_3$) δ 173.73, 173.70, 172.98, 172.93, 172.27, 158.50, 157.85, 148.93, 148.65, 137.16, 137.05, 133.55, 133.37, 133.29, 133.23, 132.93, 131.10, 130.80, 130.74, 130.71, 130.33, 129.95, 129.49, 129.37, 129.25, 129.13, 129.07, 128.92, 128.84, 128.76, 128.69, 128.46, 128.30, 127.76, 127.67, 127.60, 127.56, 127.24, 126.23, 126.04, 125.77, 125.40, 125.33, 119.77, 119.54, 103.65, 103.63, 61.49, 61.47, 56.05, 45.22, 39.18, 37.65, 30.88, 30.09, 29.82, 29.65. HRMS (ESI) m/z Calcd. for $C_{39}H_{35}BrN_5O_3$, $[M+H]^+$, 700.1918, Found: 700.1926. Enantiomeric excess was determined to be 96% (determined by HPLC using chiral OD-H column, hexane/2-propanol = 9/1, λ = 254 nm, 25 °C, 0.8 mL/min, t_{major} = 51.7 min, t_{minor} = 37.6 min).

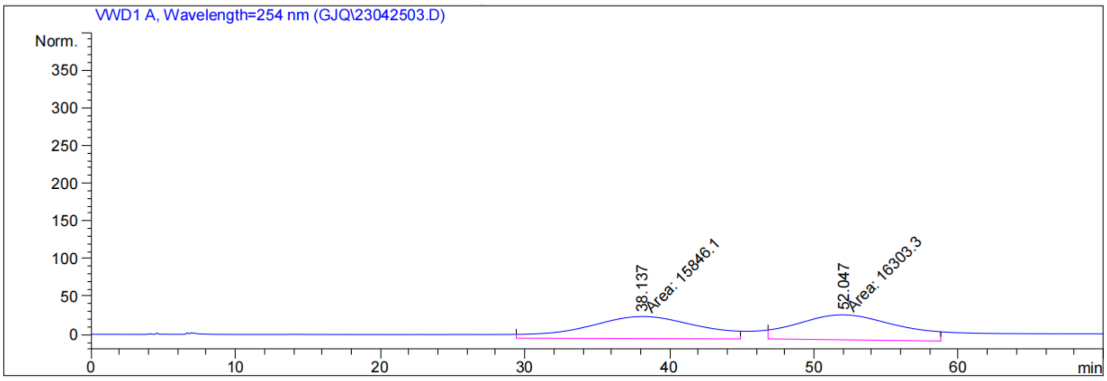

Peak #	RetTime [min]	Type	Width [min]	Area mAU *s	Height [mAU]	Area %
1	38.137	MM	9.0624	1.58461e4	29.14249	49.2889
2	52.047	MM	8.2131	1.63033e4	33.08375	50.7111

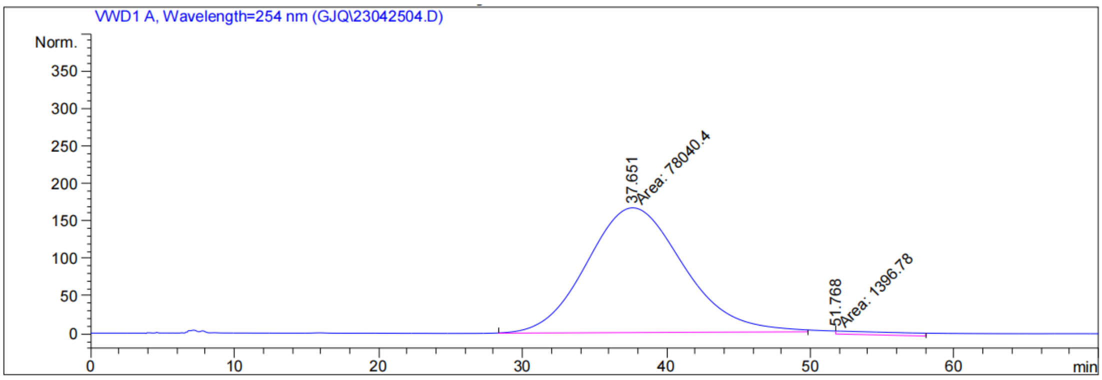

Peak #	RetTime [min]	Type	Width [min]	Area mAU *s	Height [mAU]	Area %
1	37.651	MM T	7.8287	7.80404e4	166.14117	98.2417
2	51.768	MM	5.9877	1396.77734	3.88789	1.7583

(S)-1-(1-(tert-butyl)-3-phenyl-1H-pyrazol-5-yl)-3-((R)-4-(2-nitrobenzyl)-5-oxo-1,3-diphenyl-4,5-dihydro-1H-pyrazol-4-yl)pyrrolidine-2,5-dione (3ja)

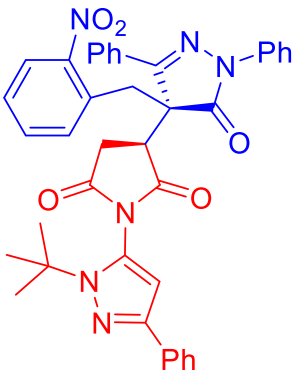

3ja

Prepared according to the procedure within 1.2 h as white solid (129.3 mg, 97% yield, dr = 1:1). mp 120.1 – 120.9 °C; $[\alpha]_D^{17}$ = -42.404 (*c* 0.44, CH_2Cl_2); ^{1}H NMR (400 MHz, Chloroform-d) δ 7.97 – 7.89 (m, 5H), 7.78 – 7.72 (m, 3H), 7.68 – 7.64 (m, 4H), 7.63 – 7.59 (m, 3H), 7.55 (t, J = 7.6 Hz, 3H), 7.50 (dd, J = 5.2, 2.0 Hz, 4H), 7.47 – 7.41 (m, 2H), 7.40 (s, 1H), 7.39 – 7.33 (m, 6H), 7.32 (s, 1H), 7.31 – 7.26 (m, 4H), 7.22 (dd, J = 5.6, 3.2 Hz, 3H), 6.27 (s, 1H), 5.37 (s, 1H), 4.43 (q, J = 14.2 Hz, 2H), 4.18 (d, J = 14.1 Hz, 1H), 4.09 (q, J = 4.4, 3.8 Hz, 2H), 4.06 (s, 1H), 3.73 (s, 1H), 3.24 – 3.12 (m, 1H), 2.93 (dd, J = 18.6, 9.6 Hz, 1H), 2.61 (s, 1H), 1.52 (s, 9H), 1.31 (s, 9H). ^{13}C NMR (101 MHz, $CDCl_3$) δ 174.87, 173.65, 173.42, 172.82, 172.12, 157.74, 157.28, 149.97, 148.93, 148.64, 136.88, 136.81, 133.27, 133.20, 133.03, 132.65, 132.32, 132.01, 131.27, 131.08, 130.42, 130.20, 129.54, 129.27, 129.09, 128.99, 128.91, 128.81, 128.72, 128.48, 128.44, 128.26, 127.78, 127.69, 127.60, 127.43, 126.69, 126.32, 126.13, 125.46, 125.37, 125.32, 119.61, 119.55, 103.61, 61.52, 61.47, 56.51, 45.05, 35.22, 34.06, 30.89, 30.03, 29.80, 29.62. HRMS (ESI) m/z Calcd. for $C_{39}H_{35}N_6O_5$, $[M+H]^+$, 667.2663, Found: 667.2673. Enantiomeric excess was determined to be 86% (determined by HPLC using chiral IG-H column, hexane/2-propanol = 7/3, λ = 254 nm, 25 °C, 0.8 mL/min, t_{major} = 55.8 min, t_{minor} = 22.1 min).

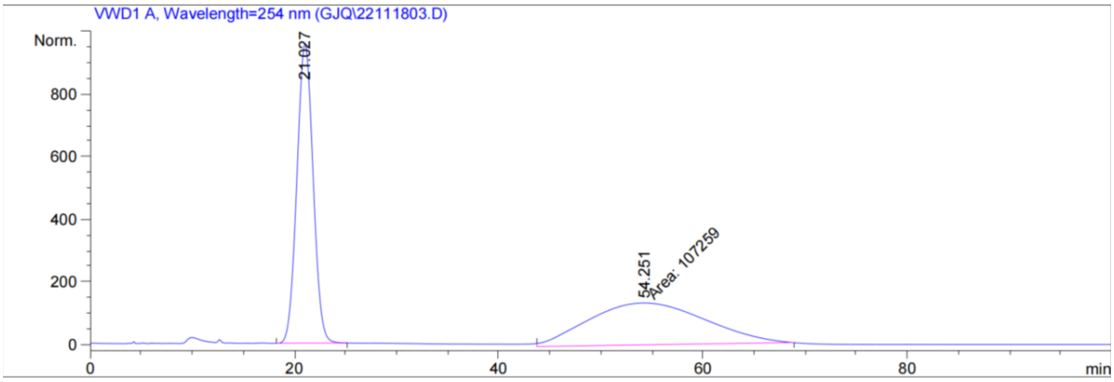

Peak #	RetTime [min]	Type	Width [min]	Area mAU *s	Height [mAU]	Area %
1	21.027	BB	1.7308	1.05528e5	953.61609	49.5933
2	54.251	MM	9.3342	1.07259e5	134.28444	50.4067

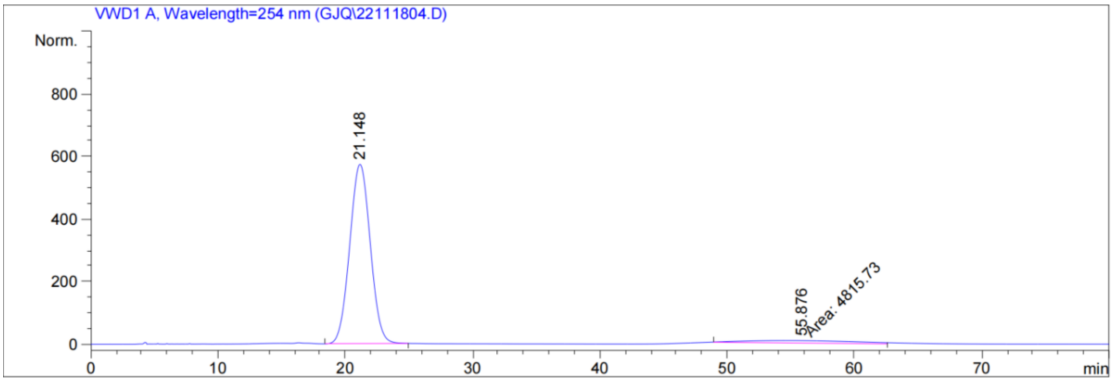

Peak #	RetTime [min]	Type	Width [min]	Area mAU *s	Height [mAU]	Area %
1	21.148	PB	1.7549	6.43660e4	572.88092	93.0390
2	55.876	MM	10.1646	4815.73242	7.89623	6.9610

(S)-1-(1-(tert-butyl)-3-phenyl-1H-pyrazol-5-yl)-3-((R)-4-(3-methylbenzyl)-5-oxo-1,3-diphenyl-4,5-dihydro-1H-pyrazol-4-yl)pyrrolidine-2,5-dione (3ka)

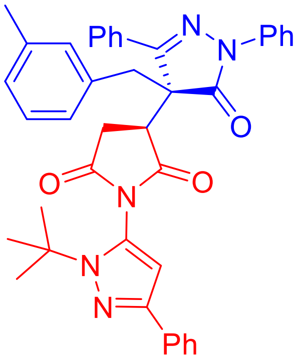

3ka

Prepared according to the procedure within 1 h as white solid (120.7 mg, 95% yield, dr = 1:1). mp 109.1 – 109.9 °C; $[\alpha]_D^{17}$ = -40.141 (*c* 0.14, CH_2Cl_2); ^{1}H NMR (400 MHz, Chloroform-d) δ 8.04 – 7.97 (m, 4H), 7.71 (d, J = 7.8 Hz, 4H), 7.66 – 7.52 (m, 10H), 7.38 (ddt, J = 16.5, 12.1, 7.9 Hz, 9H), 7.26 – 7.20 (m, 2H), 7.03 – 6.80 (m, 9H), 6.33 (s, 1H), 5.55 (s, 1H), 4.32 – 4.12 (m, 2H), 4.02 (dd, J = 9.4, 7.3 Hz, 1H), 3.85 – 3.75 (m, 1H), 3.60 (dd, J = 13.5, 4.0 Hz, 2H), 3.51 (d, J = 13.2 Hz, 1H), 3.22 (dd, J = 17.8, 9.4 Hz, 1H), 3.05 (dd, J = 18.4, 9.6 Hz, 1H), 2.10 (d, J = 8.6 Hz, 6H), 1.56 (s, 9H), 1.42 (s, 9H). ^{13}C NMR (101 MHz, $CDCl_3$) δ 173.90, 173.75, 173.62, 173.24, 158.24, 148.92, 148.64, 138.00, 137.01, 136.87, 133.17, 132.35, 131.15, 130.94, 130.65, 130.10, 129.32, 129.08, 128.95, 128.78, 128.69, 128.62, 128.46, 128.25, 128.20, 127.66, 127.59, 127.02, 126.27, 126.24, 126.02, 120.05, 119.87, 103.67, 61.48, 57.11, 44.90, 41.26, 31.07, 30.04, 29.80, 29.67, 21.16, 21.10. HRMS (ESI) m/z Calcd. for $C_{40}H_{38}N_5O_3$, $[M+H]^+$, 636.2969, Found: 636.2976. Enantiomeric excess was determined to be 96% (determined by HPLC using chiral IB-H column, hexane/2-propanol = 4/1, λ = 254 nm, 25 °C, 0.6 mL/min, t_{major} = 51.1 min, t_{minor} = 41.1 min).

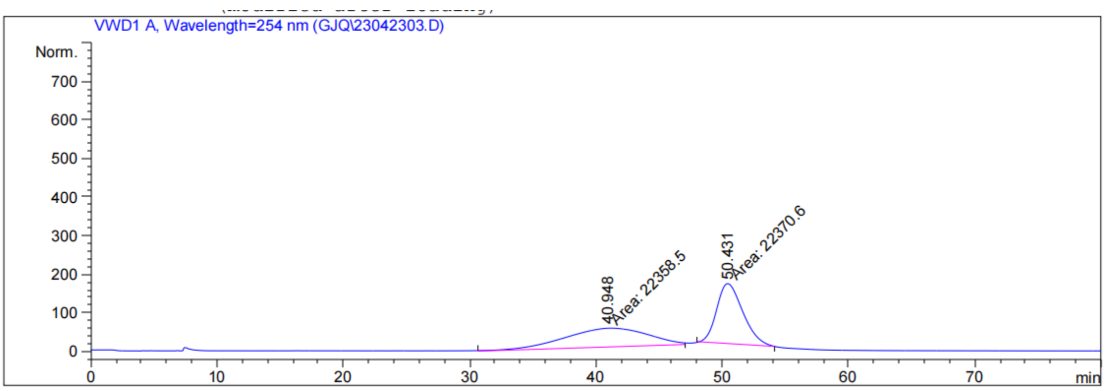

Peak #	RetTime [min]	Type	Width [min]	Area mAU *s	Height [mAU]	Area %
1	40.948	MM T	8.4498	2.23585e4	48.63107	49.9864
2	50.431	MM T	2.4018	2.23706e4	155.23814	50.0136

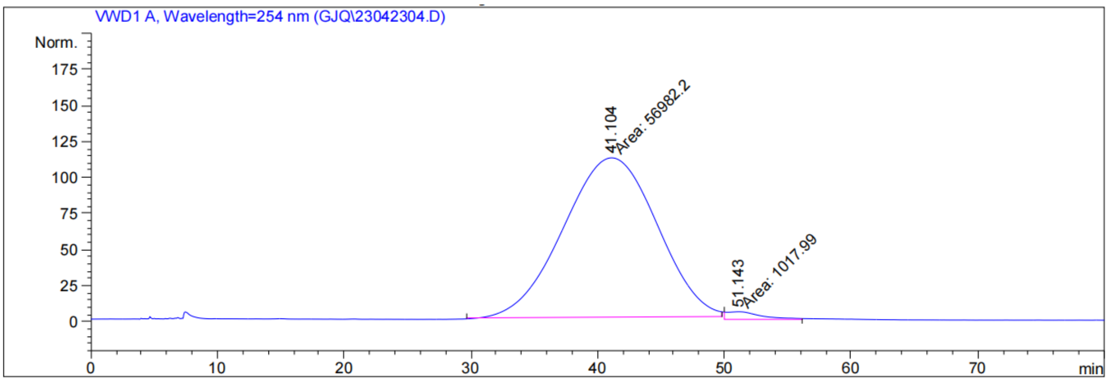

Peak #	RetTime [min]	Type	Width [min]	Area mAU *s	Height [mAU]	Area %
1	41.104	MM	8.5845	5.69822e4	110.63047	98.2448
2	51.143	MM	3.1904	1017.99371	5.31807	1.7552

(S)-1-(1-(tert-butyl)-3-phenyl-1H-pyrazol-5-yl)-3-((R)-4-(4-methylbenzyl)-5-oxo-1,3-diphenyl-4,5-dihydro-1H-pyrazol-4-yl)pyrrolidine-2,5-dione (3la)

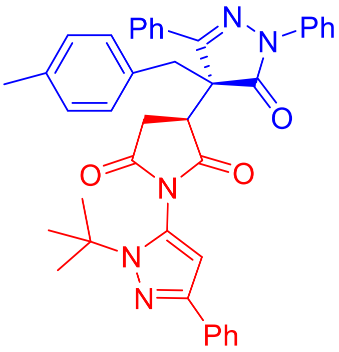

3la

Prepared according to the procedure within 1.5 h as white solid (124.5 mg, 98% yield, dr = 1:1). mp 112.1 – 112.9 ºC; $[\alpha]_D^{17}$ = -30.363 (*c* 0.85, CH_2Cl_2); ^{1}H NMR (400 MHz, Chloroform-d) δ 8.04 – 7.95 (m, 4H), 7.67 (ddd, J = 8.1, 5.5, 1.2 Hz, 4H), 7.65 – 7.58 (m, 5H), 7.58 – 7.47 (m, 5H), 7.41 – 7.25 (m, 10H), 7.24 – 7.16 (m, 2H), 6.97 (d, J = 7.8 Hz, 2H), 6.93 – 6.84 (m, 6H), 6.29 (s, 1H), 5.50 (s, 1H), 4.20 (dd, J = 18.6, 6.7 Hz, 2H), 3.96 (dd, J = 9.3, 7.3 Hz, 1H), 3.74 (dd, J = 9.3, 5.5 Hz, 1H), 3.57 (dd, J = 13.6, 10.3 Hz, 2H), 3.46 (d, J = 13.3 Hz, 1H), 3.16 (dd, J = 17.9, 9.4 Hz, 1H), 2.99 (dd, J = 18.5, 9.7 Hz, 1H), 2.83 (s, 1H), 2.16 (d, J = 2.5 Hz, 6H), 1.37 (s, 9H). ^{13}C NMR (101 MHz, $CDCl_3$) δ 174.84, 173.87, 173.72, 173.55, 173.19, 173.09, 158.20, 157.51, 148.93, 148.65, 137.58, 137.39, 137.09,

136.93, 133.34, 133.27, 131.15, 131.04, 130.94, 130.65, 130.19, 129.40, 129.35, 129.19, 129.12, 129.07, 128.96, 128.82, 128.67, 128.46, 127.66, 127.60, 127.02, 126.23, 125.99, 125.39, 125.34, 120.07, 119.91, 103.67, 61.48, 57.20, 44.95, 43.63, 40.90, 39.63, 31.04, 30.02, 29.80, 29.67, 20.99. HRMS (ESI) m/z Calcd. for $C_{40}H_{38}N_5O_3$, $[M+H]^+$, 636.2969, found: 636.2976. Enantiomeric excess was determined to be 99% (determined by HPLC using chiral ID-H-OD-H column, hexane/2-propanol = 7/3, λ = 254 nm, 25 °C, 0.8 mL/min, t_{major} = 48.6 min, t_{minor} = 31.9 min).

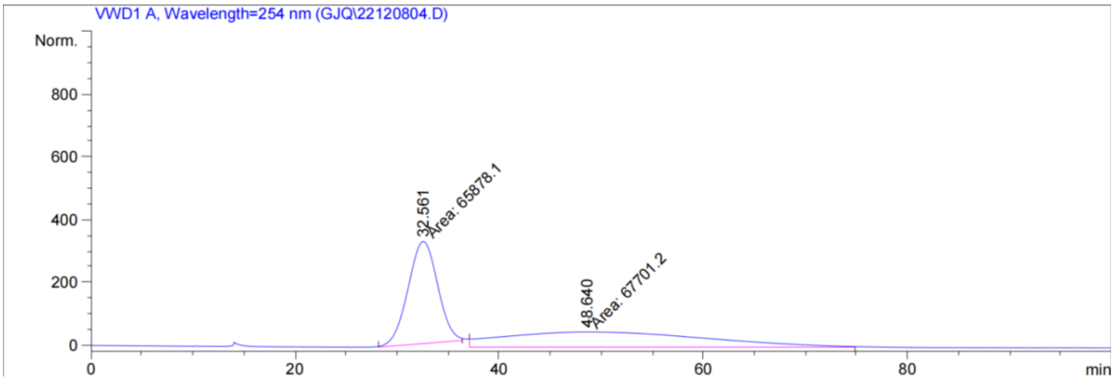

Peak #	RetTime [min]	Type	Width [min]	Area mAU *s	Height [mAU]	Area %
1	32.561	MM	3.3724	6.58781e4	325.57639	49.3176
2	48.640	MM T	23.1456	6.77012e4	48.75010	50.6824

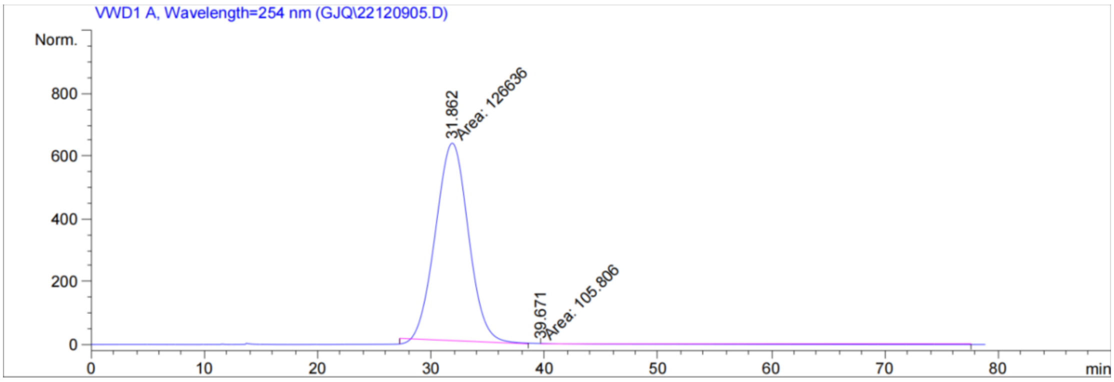

Peak #	RetTime [min]	Type	Width [min]	Area mAU *s	Height [mAU]	Area %
1	31.862	MM	3.3545	1.26636e5	629.17596	99.9165
2	39.671	MM	0.8980	105.80643	1.38719	0.0835

(S)-1-(1-(tert-butyl)-3-phenyl-1H-pyrazol-5-yl)-3-((R)-4-(4-methoxybenzyl)-5-oxo-1,3-diphenyl-4,5-dihydro-1H-pyrazol-4-yl)pyrrolidine-2,5-dione (3ma)

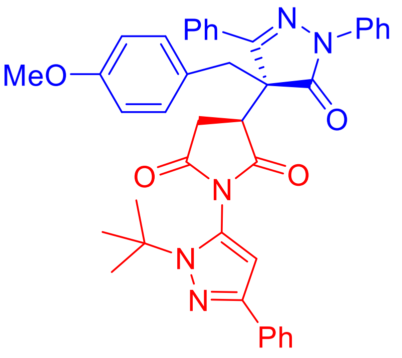

3ma

Prepared according to the procedure within 1.5 h as white solid (129.0 mg, 99% yield, dr = 1:1). mp 112.1 – 112.9 °C; $[\alpha]_D^{17}$ = -29.344 (*c* 0.78, CH_2Cl_2); ^1H NMR (400 MHz, Chloroform-d) δ 8.04 – 7.96 (m, 4H), 7.71 – 7.65 (m, 4H), 7.64 – 7.58 (m, 5H), 7.58 – 7.48 (m, 5H), 7.41 – 7.27 (m, 9H), 7.24 – 7.17 (m, 2H), 7.04 – 6.98 (m, 2H), 6.96 – 6.91 (m, 2H), 6.64 – 6.57 (m, 4H), 6.29 (s, 1H), 5.51 (s, 1H), 4.20 (dd, J = 18.1, 7.4 Hz, 2H), 3.96 (dd, J = 9.3, 7.3 Hz, 1H), 3.74 (dd, J = 9.0, 5.8 Hz, 1H), 3.63 (d, J = 2.0 Hz, 6H), 3.56 (dd, J = 18.2, 13.7 Hz, 2H), 3.44 (d, J = 13.3 Hz, 1H), 3.16 (dd, J = 17.8, 9.4 Hz, 1H), 3.00 (dd, J = 18.5, 9.6 Hz, 1H), 2.83 (s, 1H), 1.52 (s, 9H), 1.38 (s, 9H). ^{13}C NMR (101 MHz, $CDCl_3$) δ 174.85, 173.91, 173.75, 173.57, 173.22, 173.11, 159.15, 159.02, 158.20, 157.51, 148.92, 148.65, 137.08, 136.91, 133.33, 133.25, 131.15, 131.04, 130.97, 130.67, 130.45, 129.39, 129.15, 128.98, 128.83, 128.70, 128.46, 127.67, 127.60, 127.56, 126.99, 126.24, 126.00, 125.39, 125.34, 125.23, 124.45, 120.02, 119.88, 113.81, 113.76, 103.67, 61.49, 57.30, 55.11, 44.88, 43.59, 40.52, 39.26, 31.04, 30.00, 29.80, 29.68. HRMS (ESI) m/z Calcd. for $C_{40}H_{38}N_5O_4$, $[M+H]^+$, 652.2918, found: 652.2922. Enantiomeric excess was determined to be 99% (determined by HPLC using chiral OD-H column, hexane/2-propanol = 9/1, λ = 254 nm, 25 °C, 0.8 mL/min, t_{major} = 74.5 min, t_{minor} = 51.3 min).

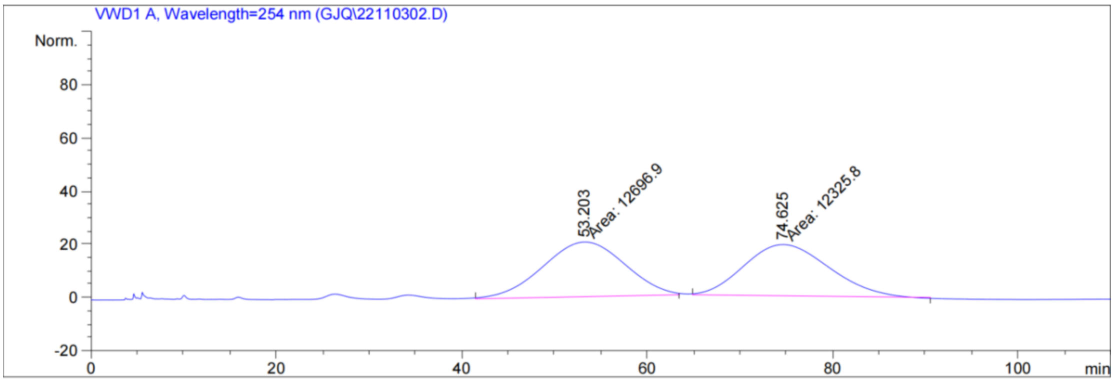

Peak #	RetTime [min]	Type	Width [min]	Area mAU *s	Height [mAU]	Area %
1	53.203	MM	10.2805	1.26969e4	20.58406	50.7415
2	74.625	MM	10.6463	1.23258e4	19.29592	49.2585

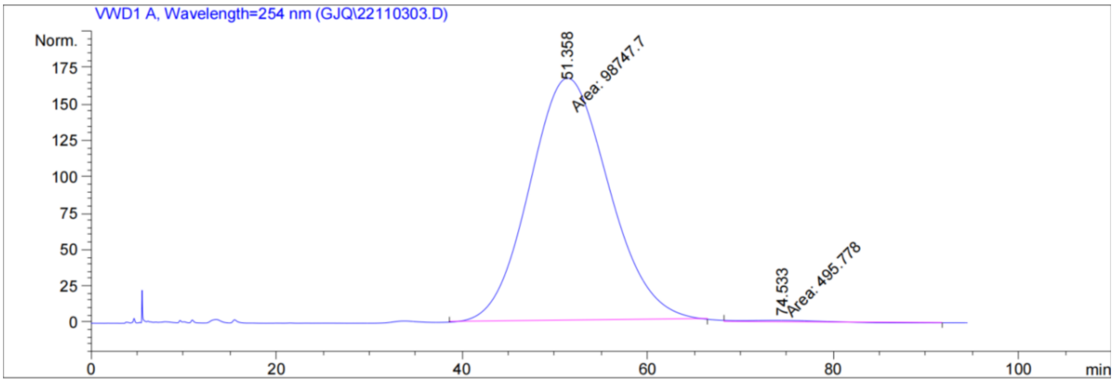

Peak #	RetTime [min]	Type	Width [min]	Area mAU *s	Height [mAU]	Area %
1	51.358	MM	9.8925	9.87477e4	166.36861	99.5004
2	74.533	MM	9.0046	495.77789	9.17636e-1	0.4996

(S)-3-((R)-4-(3,5-bis(trifluoromethyl)benzyl)-5-oxo-1,3-diphenyl-4,5-dihydro-1H-pyrazol-4-yl)-1-(1-(tert-butyl)-3-phenyl-1H-pyrazol-5-yl)pyrrolidine-2,5-dione (3na)

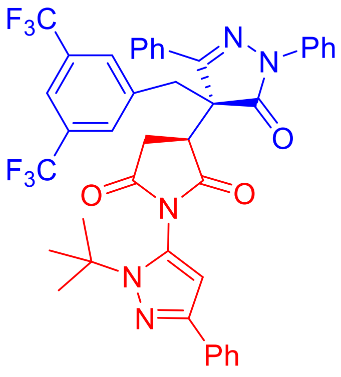

3na

Prepared according to the procedure within 2.5 h as white solid (146.9 mg, 97% yield, dr = 1:1). mp 112.1 – 112.9 ºC; $[\alpha]_D^{17}$ = 1.1617 (*c* 0.80, CH_2Cl_2); ^{1}H NMR (400 MHz, Chloroform-d) δ 7.94 (d, J = 7.4 Hz, 4H), 7.72 – 7.47 (m, 20H), 7.41 – 7.30 (m, 10H), 7.23 (d, J = 8.7 Hz, 2H), 6.32 (s, 1H), 5.58 (s, 1H), 4.23 (s, 2H), 4.11 (t, J = 8.2 Hz, 1H), 3.81 (t, J = 7.3 Hz, 1H), 3.70 (dd, J = 22.0, 13.6 Hz, 2H), 3.60 (d, J = 13.1 Hz, 1H), 3.21 (dd, J = 17.6, 9.1 Hz, 1H), 3.03 (dd, J = 18.6, 9.7 Hz, 1H), 2.78 (s, 1H), 1.56 (s, 9H), 1.35 (s, 9H). ^{13}C NMR (151 MHz, $CDCl_3$) δ 173.53, 173.32, 172.71, 172.68, 172.32, 157.34, 156.70, 148.97, 148.70, 136.49, 136.38, 135.93, 135.13, 133.21, 133.09, 131.96, 131.79, 131.74, 131.57, 131.52, 131.35, 131.29, 130.36 (d, J=14.14 Hz), 129.75, 129.62, 129.42, 129.10, 128.84, 128.63, 128.53, 128.50, 128.29, 127.78, 127.70, 127.13, 126.63, 126.60, 126.44, 125.51, 125.37, 125.30, 123.74, 123.69, 121.93, 121.89, 121.83, 119.66, 119.48, 103.64, 103.59, 61.58, 61.53, 56.82, 44.86, 43.37, 40.35, 39.14, 30.90, 29.81, 29.77, 29.62. ^{19}F NMR (376 MHz, $CDCl_3$) δ -63.14, -63.17. HRMS (ESI) m/z Calcd. for $C_{41}H_{34}F_6N_5O_3$, $[M+H]^+$, 758.2560, Found: 758.2570. Enantiomeric excess was determined to be 95% (determined by HPLC using chiral OD-H column, hexane/2-propanol = 9/1, λ = 254 nm, 25 ºC, 0.8 mL/min, t_{major} = 41.5 min, t_{minor} = 24.1 min).

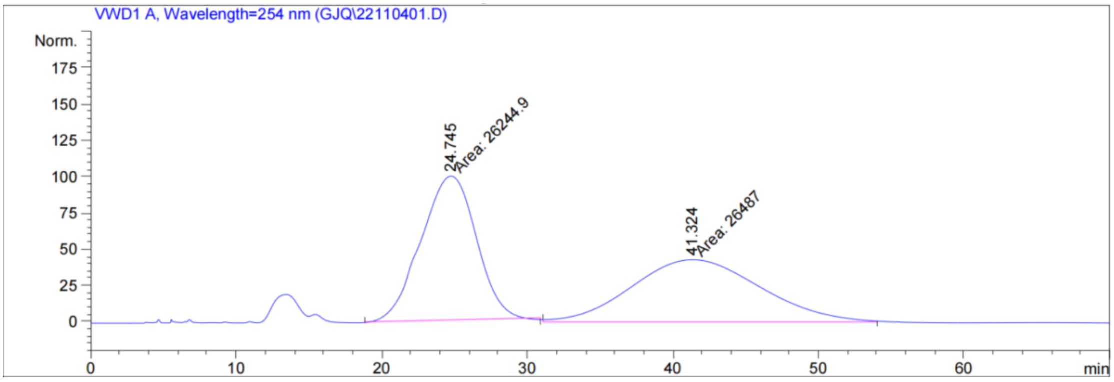

Peak #	RetTime [min]	Type	Width [min]	Area mAU *s	Height [mAU]	Area %
1	24.745	MM	4.4064	2.62449e4	99.26736	49.7704
2	41.324	MM	10.2737	2.64870e4	42.96878	50.2296

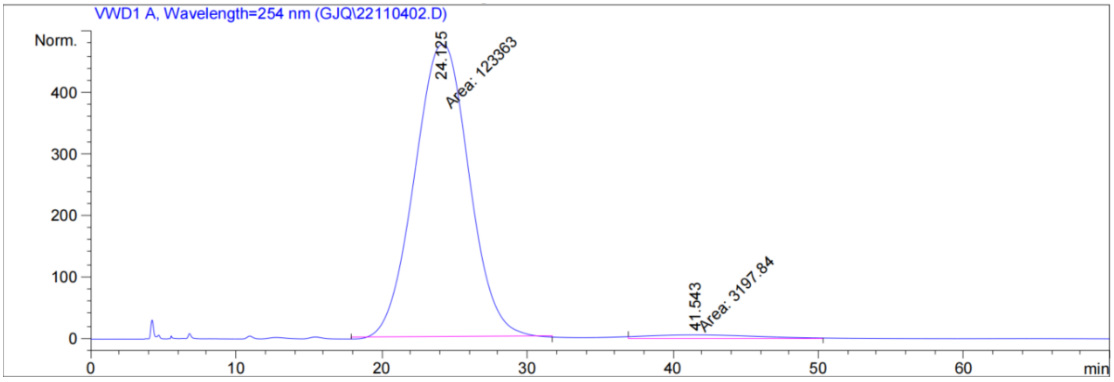

Peak #	RetTime [min]	Type	Width [min]	Area mAU *s	Height [mAU]	Area %
1	24.125	MM	4.3269	1.23363e5	475.18039	97.4733
2	41.543	MM	8.9395	3197.83618	5.96202	2.5267

(S)-1-(1-(tert-butyl)-3-phenyl-1H-pyrazol-5-yl)-3-((R)-4-(naphthalen-2-ylmethyl)-5-oxo-1,3-diphenyl-4,5-dihydro-1H-pyrazol-4-yl)pyrrolidine-2,5-dione (3oa)

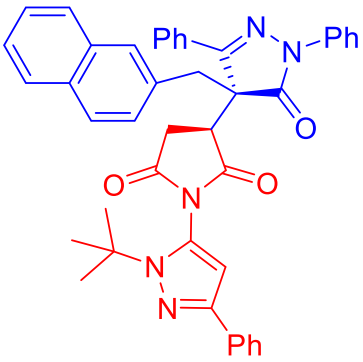

3oa

Prepared according to the procedure within 4 h as white solid (99.4 mg, 74% yield, dr = 1:1). mp 116.1 – 116.9 °C; $[\alpha]_D^{17}$ = -66.632 (c 0.96, CH_2Cl_2); ^{1}H NMR (600 MHz, Chloroform-d) δ 8.19 (d, J = 8.3 Hz, 1H), 8.08 – 8.03 (m, 1H), 7.96 – 7.93 (m, 2H), 7.92 – 7.89 (m, 2H), 7.76 – 7.71 (m, 2H), 7.69 – 7.56 (m, 8H), 7.52 – 7.38 (m, 13H), 7.37 – 7.26 (m, 11H), 7.24 – 7.14 (m, 9H), 7.13 – 7.09 (m, 1H), 6.32 (s, 1H), 5.49 (s, 1H), 4.60 (s, 1H), 4.40 (s, 1H), 4.29 (d, J = 15.0 Hz, 1H), 4.17 (dd, J = 15.4, 6.8 Hz, 2H), 4.00 – 3.86 (m, 2H), 3.29 (dd, J = 17.9, 9.4 Hz, 1H), 3.04 (dd, J = 18.9, 9.9 Hz, 1H), 2.88 (s, 1H), 1.56 (d, J = 2.0 Hz, 9H), 1.34 (s, 9H). ^{13}C NMR (101 MHz, Chloroform-d) δ 173.91, 173.71, 173.46, 173.02, 158.36, 148.97, 148.62, 136.74, 136.68, 133.82, 133.75, 133.33, 133.25, 131.82, 131.68, 131.31, 131.06, 130.97, 130.60, 129.91, 129.35, 129.06, 128.98, 128.88, 128.79, 128.73, 128.69, 128.54, 128.51, 128.45, 127.90, 127.66, 127.58, 127.04, 126.74, 126.17, 126.04, 125.97, 125.75, 125.70, 125.39, 125.34, 124.92, 124.76, 123.67, 123.50, 120.06, 119.71, 103.67, 103.63, 61.51, 61.47, 56.60, 45.40, 43.90, 36.34, 34.65, 31.09, 30.26, 29.84, 29.65. HRMS (ESI) m/z Calcd. for $C_{43}H_{38}N_5O_3$, $[M+H]^+$, 672.2969, Found: 672.2981. Enantiomeric excess was determined to be 94% (determined by HPLC using chiral IB-H column, hexane/2-propanol = 4/1, λ = 254 nm, 25 °C, 0.8 mL/min, t_{major} = 56.4 min, t_{minor} = 47.9 min).

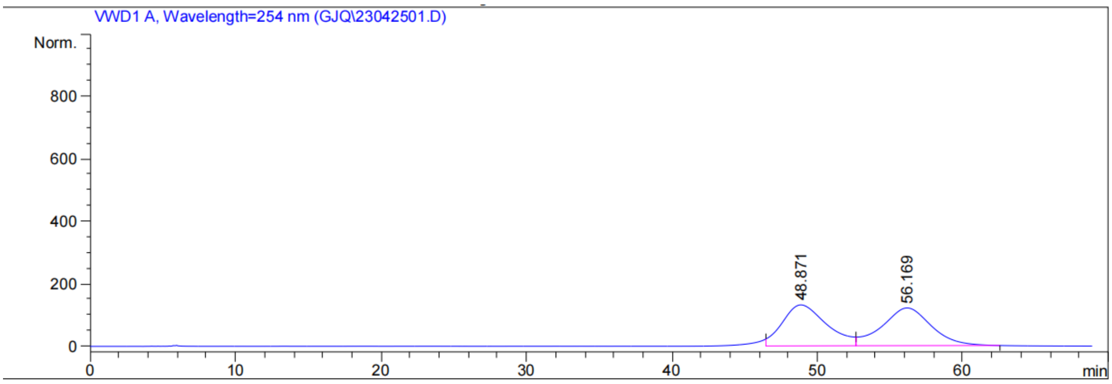

Peak #	RetTime [min]	Type	Width [min]	Area mAU *s	Height [mAU]	Area %
1	48.871	BV	2.9766	2.77069e4	131.02893	49.4175
2	56.169	VB	3.2680	2.83601e4	120.77757	50.5825

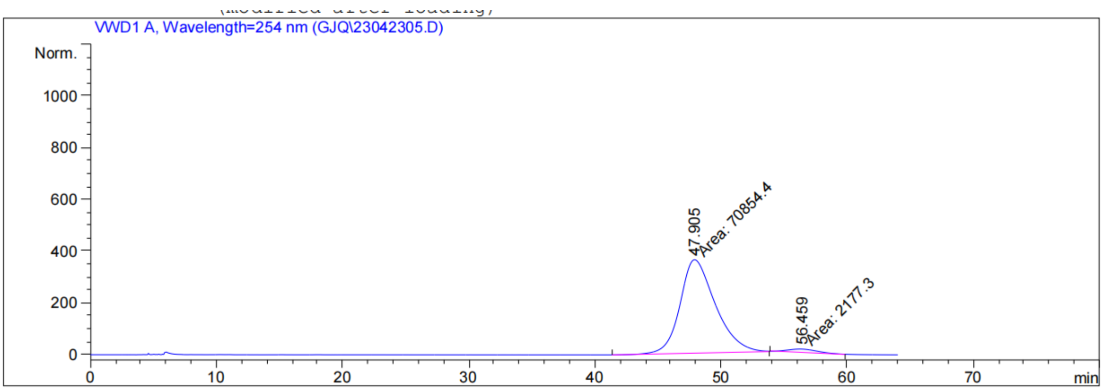

Peak #	RetTime [min]	Type	Width [min]	Area mAU *s	Height [mAU]	Area %
1	47.905	MM	3.2651	7.08544e4	361.67343	97.0187
2	56.459	MM T	2.7281	2177.30493	13.30177	2.9813

(S)-3-((R)-4-benzyl-5-oxo-1,3-diphenyl-4,5-dihydro-1H-pyrazol-4-yl)-1-(1-isopropyl-3-phenyl-1H-pyrazol-5-yl)pyrrolidine-2,5-dione (3ab)

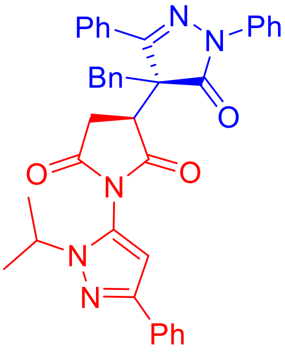

3ab

Prepared according to the procedure within 1 h as white solid (120.2 mg, 99% yield, dr > 20:1). mp 105.1 – 105.9 ºC; $[\alpha]_D^{17}$ = 9.067 (*c* 1.15, CH_2Cl2); ^{1}H NMR (400 MHz, Chloroform-d) δ 8.04 – 7.95 (m, 2H), 7.76 – 7.62 (m, 4H), 7.53 (dt, J = 5.3, 2.7 Hz, 3H), 7.34 (q, J = 7.4 Hz, 4H), 7.27 (d, J = 1.4 Hz, 1H), 7.20 (dd, J = 7.4 Hz, 1H), 7.15 – 7.01 (m, 5H), 6.17 (s, 1H), 3.94 (dd, J = 9.4, 5.7 Hz, 1H), 3.82 (s, 2H), 3.56 (d, J = 13.4 Hz, 1H), 3.16 (dd, J = 18.3, 9.7 Hz, 1H), 1.58 (s, 1H), 1.35 (d, J = 6.6 Hz, 3H), 1.10 (s, 3H). ^{13}C NMR (101 MHz, $CDCl_3$) δ 173.72, 173.51, 172.82, 150.49, 136.88, 133.39, 132.76, 130.98, 130.85, 129.31, 129.21, 128.83, 128.49, 128.40, 128.12, 127.91, 127.68, 127.17, 126.14, 125.51, 119.77, 100.97, 50.25, 44.09, 40.68, 30.51, 22.49, 22.11. HRMS (ESI) m/z Calcd. for $C_{38}H_{34}N_5O_3$, $[M+H]^+$, 608.2656, Found: 608.2666. Enantiomeric excess was determined to be 96% (determined by HPLC using chiral OD-H column, hexane/2-propanol = 7/3, λ = 254 nm, 25 ºC, 0.8 mL/min, t_{major} = 25.3 min, t_{minor} = 13.6 min).

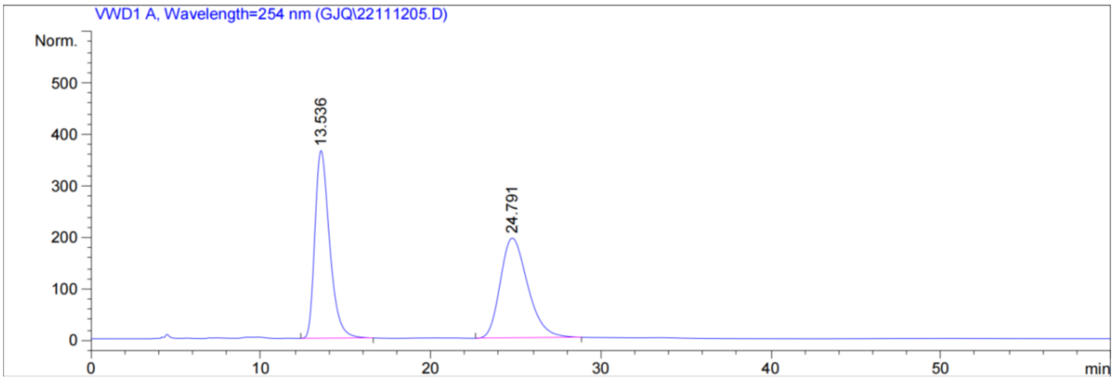

Peak #	RetTime [min]	Type	Width [min]	Area mAU *s	Height [mAU]	Area %
1	13.536	BB	0.9235	2.19903e4	364.61627	50.2205
2	24.791	BB	1.7339	2.17972e4	194.08760	49.7795

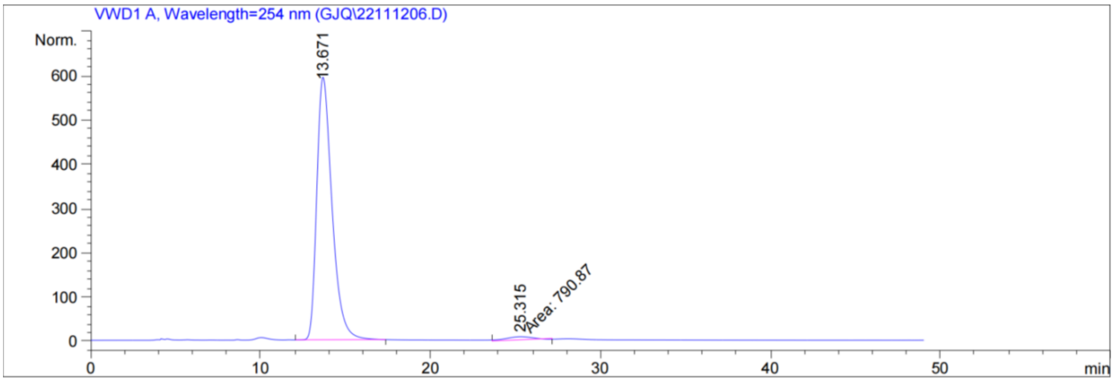

Peak #	RetTime [min]	Type	Width [min]	Area mAU *s	Height [mAU]	Area %
1	13.671	VB	0.9557	3.69886e4	592.29419	97.9066
2	25.315	MM	1.3057	790.86957	7.24189	2.0934

(S)-3-((R)-4-benzyl-5-oxo-1,3-diphenyl-4,5-dihydro-1H-pyrazol-4-yl)-1-(1,3-diphenyl-1H-pyrazol-5-yl)pyrrolidine-2,5-dione (3ac)

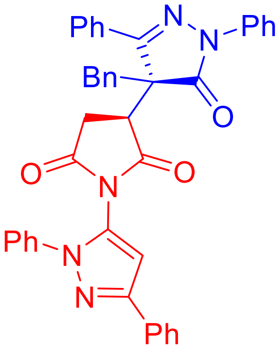

3ac

Prepared according to the procedure within 1 h as white solid (123.1 mg, 96% yield, dr > 20:1). mp 108.1 – 108.9 ºC; $[\alpha]_D^{17}$ = 11.915 (*c* 1.13, CH_2Cl_2); ^{1}H NMR (400 MHz, Chloroform-d) δ 7.98 – 7.91 (m, 2H), 7.78 – 7.72 (m, 2H), 7.59 – 7.48 (m, 5H), 7.43 – 7.26 (m, 9H), 7.22 (d, J = 11.9 Hz, 2H), 7.12 – 6.99 (m, 5H), 6.28 (s, 1H), 4.02 – 3.09 (m, 4H), 2.95 (dd, J = 18.1, 9.4 Hz, 1H). ^{13}C NMR (101 MHz, $CDCl_3$) δ 173.51, 173.01, 172.25, 157.43, 151.85, 137.82, 136.86, 132.51, 131.02, 130.82, 129.78, 129.42, 129.32, 129.25, 128.82, 128.64, 128.35, 128.32, 127.84, 127.19, 126.19, 125.68, 124.19, 120.15, 103.42, 44.16, 40.63, 30.50. HRMS (ESI) m/z Calcd. for $C_{41}H_{32}N_5O_3$, $[M+H]^+$, 642.2500, Found: 642.2507. Enantiomeric excess was determined to be 99% (determined by HPLC using chiral OD-H column, hexane/2-propanol = 7/3, λ = 254 nm, 25 ℃, 0.8 mL/min, t_{major} = 33.7 min, t_{minor} = 22.2 min).

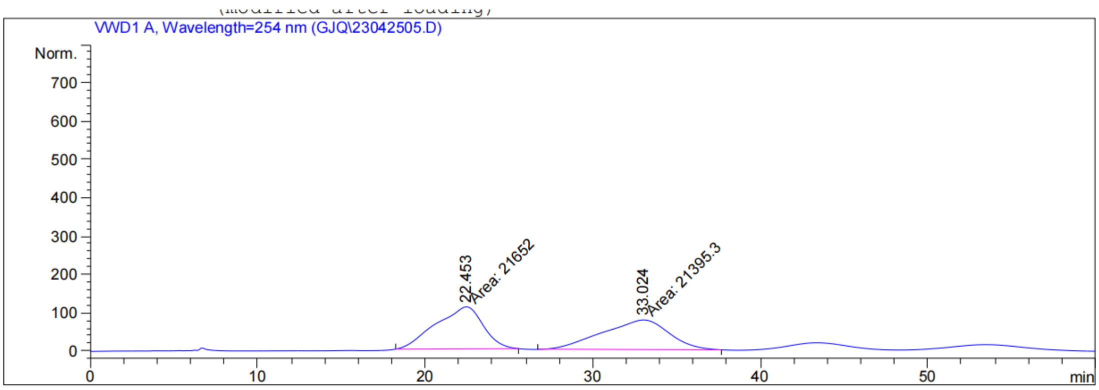

Peak #	RetTime [min]	Type	Width [min]	Area mAU *s	Height [mAU]	Area %
1	22.453	MM T	3.2914	2.16520e4	109.63880	50.2982
2	33.024	MM T	4.6189	2.13953e4	77.20210	49.7018

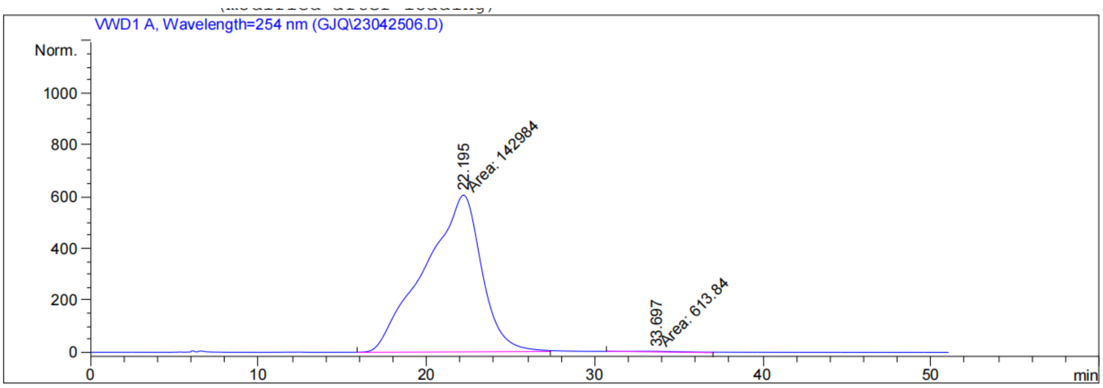

Peak #	RetTime [min]	Type	Width [min]	Area mAU *s	Height [mAU]	Area %
1	22.195	MM T	3.9314	1.42984e5	606.15686	99.5725
2	33.697	MM T	4.1505	613.84021	2.46493	0.4275

(S)-3-((R)-4-benzyl-5-oxo-1,3-diphenyl-4,5-dihydro-1H-pyrazol-4-yl)-1-(1-cyclohexyl-3-phenyl-1H-pyrazol-5-yl)pyrrolidine-2,5-dione (3ad)

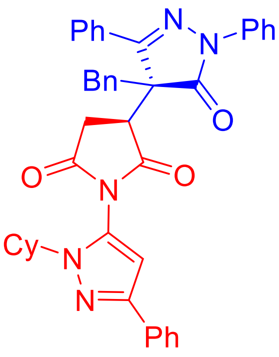

3ad

Prepared according to the procedure within 1 h as white solid (124.3 mg, 96% yield, dr > 20:1). mp 112.1 – 112.9 °C; $[\alpha]_D^{17}$ = 10.511 (*c* 1.05, CH_2Cl_2); ^{1}H NMR (400 MHz, Chloroform-d) δ 7.99 (dd, J = 6.6, 3.0 Hz, 2H), 7.71 – 7.65 (m, 2H), 7.63 – 7.57 (m, 2H), 7.56 – 7.49 (m, 3H), 7.33 (t, J = 7.4 Hz, 4H), 7.27 (d, J = 1.4 Hz, 1H), 7.18 (dd, J = 7.4 Hz, 1H), 7.09 (q, J = 5.0, 3.3 Hz, 5H), 6.16 (s, 1H), 3.96 (t, J = 7.9 Hz, 1H), 3.78 (s, 1H), 3.55 (d, J = 13.3 Hz, 1H), 3.47 (dt, J = 11.3, 6.7 Hz, 1H), 3.17 (dd, J = 18.2, 9.6 Hz, 1H), 1.87 (q, J = 6.6 Hz, 4H), 1.56 – 0.76 (m, 6H). ^{13}C NMR (151 MHz, $CDCl_3$) δ 173.86, 172.95, 157.77, 150.33, 136.82, 133.41, 130.97, 130.82, 129.36, 129.25, 128.81, 128.51, 128.39, 128.25, 127.95, 127.67, 127.10, 126.24, 125.52, 120.25, 100.94, 57.88, 44.11, 40.89, 32.82, 32.72, 30.52, 25.39, 25.07. HRMS (ESI) m/z Calcd. for $C_{41}H_{38}N_5O_3$, $[M+H]^+$,

648.2969, Found: 648.2978. Enantiomeric excess was determined to be 96% (determined by HPLC using chiral OD-H column, hexane/2-propanol = 7/3, λ = 254 nm, 25 °C, 0.8 mL/min, t_{major} = 20.6 min, t_{minor} = 11.3 min).

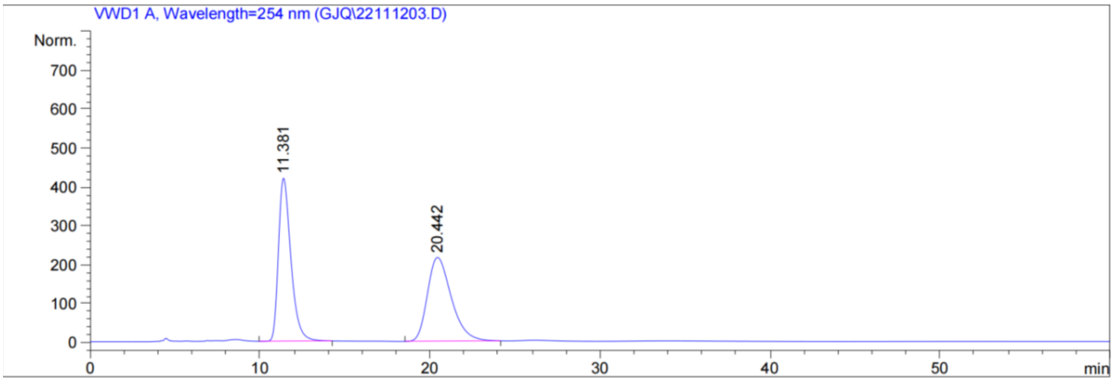

Peak #	RetTime [min]	Type	Width [min]	Area mAU *s	Height [mAU]	Area %
1	11.381	VB	0.7808	2.13340e4	418.68210	50.3124
2	20.442	BB	1.4919	2.10690e4	215.16153	49.6876

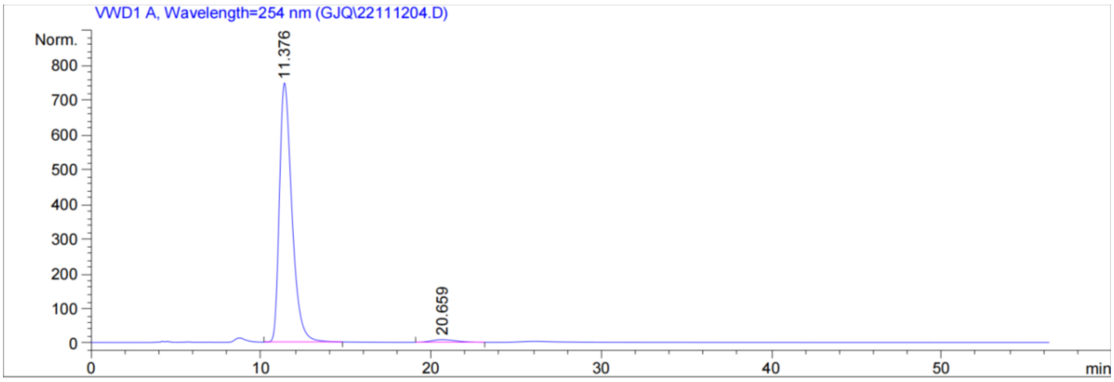

Peak #	RetTime [min]	Type	Width [min]	Area mAU *s	Height [mAU]	Area %
1	11.376	BB	0.7803	3.80303e4	745.22333	98.1156
2	20.659	BB	1.2031	730.40985	7.52933	1.8844

(S)-3-((R)-4-benzyl-5-oxo-1,3-diphenyl-4,5-dihydro-1H-pyrazol-4-yl)-1-(1-(tert-butyl)-3-(3-chlorophenyl)-1H-pyrazol-5-yl)pyrrolidine-2,5-dione (3ae)

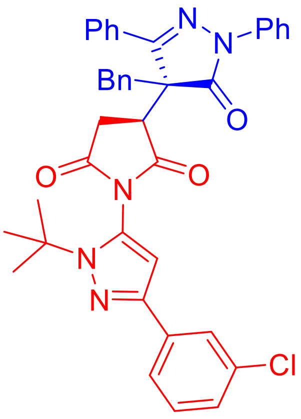

3ae

Prepared according to the procedure within 1.1 h as white solid (123.2 mg, 94% yield, dr = 1:1). mp 120.1 – 120.9 °C; $[\alpha]_D^{17}$ = -17.123 (*c* 0.22, CH_2Cl_2); ^1H NMR (400 MHz, Chloroform-d) δ 8.03 – 7.97 (m, 4H), 7.71 – 7.45 (m, 15H), 7.42 – 7.27 (m, 5H), 7.24 – 7.16 (m, 4H), 7.13 – 7.05 (m, 8H), 7.04 – 7.00 (m, 2H), 6.28 (s, 1H), 5.45 (s, 1H), 4.23 (d, J = 26.3 Hz, 2H), 4.00 (dd, J = 9.4, 7.4 Hz, 1H), 3.80 – 3.74 (m, 1H), 3.61 (dd, J = 18.0, 13.5 Hz, 2H), 3.50 (d, J = 13.2 Hz, 1H), 3.19 (dd, J = 17.8, 9.4 Hz, 1H), 3.02 (dd, J = 18.4, 9.7 Hz, 1H), 2.83 (s, 1H), 1.52 (s, 9H), 1.38 (s, 9H). ^{13}C NMR (101 MHz, $CDCl_3$) δ 174.73, 173.82, 173.63, 173.42, 173.01, 158.05, 157.35, 147.57, 147.29, 136.97, 136.79, 135.09, 134.99, 134.44, 133.23, 132.45, 131.07, 131.03, 131.00, 130.73, 129.75, 129.42, 129.34, 129.17, 129.04, 128.99, 128.72, 128.45, 128.39, 127.96, 127.81, 127.62, 127.58, 126.98, 126.39, 126.06, 125.39, 123.47, 123.36, 120.01, 119.87, 103.89, 103.86, 61.78, 61.75, 57.11, 44.94, 43.72, 41.24, 40.01, 31.03, 30.04, 29.78, 29.65. HRMS (ESI) m/z Calcd. for $C_{39}H_{35}ClN_5O_3$, $[M+H]^+$, 656.2423, Found: 656.2434. Enantiomeric excess was determined to be 97% (determined by HPLC using chiral OD-H column, hexane/2-propanol = 8/2, λ = 254 nm, 25 °C, 0.8 mL/min, t_{major} = 36.5 min, t_{minor} = 19.4 min).

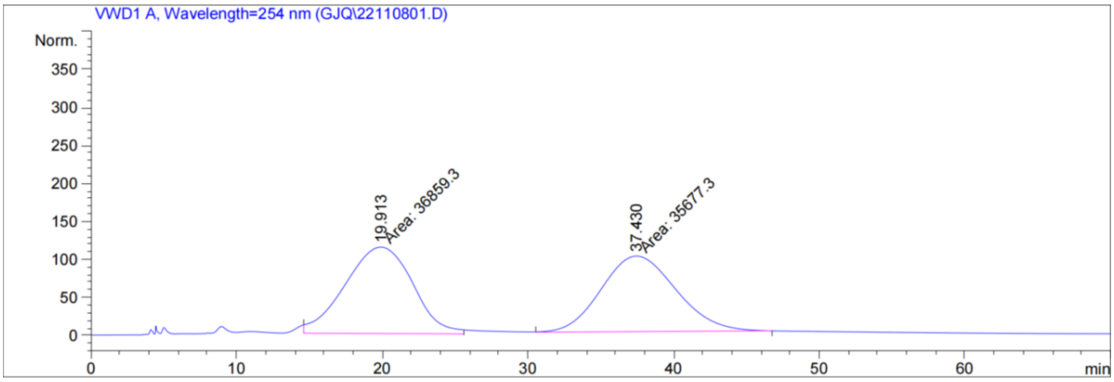

Peak #	RetTime [min]	Type	Width [min]	Area mAU *s	Height [mAU]	Area %
1	19.913	MM T	5.3959	3.68593e4	113.84943	50.8148
2	37.430	MM T	5.9743	3.56773e4	99.53004	49.1852

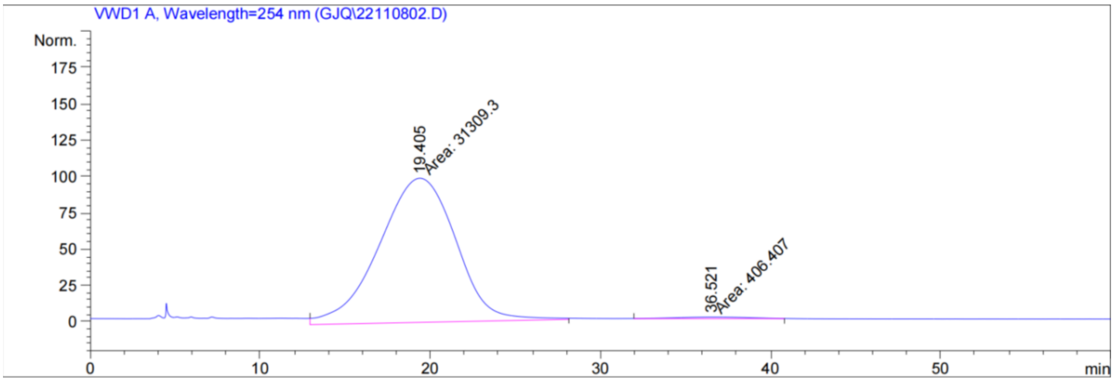

Peak #	RetTime [min]	Type	Width [min]	Area mAU *s	Height [mAU]	Area %
1	19.405	MM	3.7002	3.13093e4	99.38270	98.7186
2	36.521	MM T	5.5009	406.40714	1.23134	1.2814

(S)-3-((R)-4-benzyl-5-oxo-1,3-diphenyl-4,5-dihydro-1H-pyrazol-4-yl)-1-(1-(tert-butyl)-3-(3-(trifluoromethyl)phenyl)-1H-pyrazol-5-yl)pyrrolidine-2,5-dione (3af)

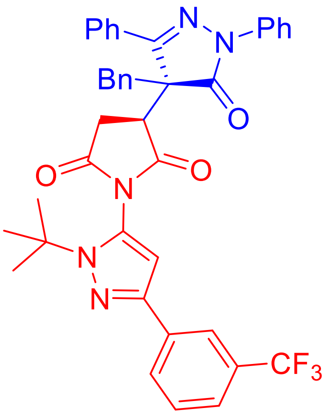

3af

Prepared according to the procedure within 1 h as white solid (128.2 mg, 93% yield, dr = 1:1). mp 118.1 – 118.9 °C; $[\alpha]_D^{17}$ = -24.115 (*c* 0.68, CH2Cl2); ^{1}H NMR (400 MHz, Chloroform-d) δ 8.03 – 7.97 (m, 4H), 7.92 (s, 1H), 7.86 – 7.79 (m, 3H), 7.66 (d, J = 8.1 Hz, 2H), 7.62 (d, J = 7.0 Hz, 1H), 7.58 (dd, J = 8.3, 3.0 Hz, 4H), 7.51 (dtd, J = 11.6, 8.9, 7.8, 3.9 Hz, 6H), 7.44 (d, J = 8.1 Hz, 1H), 7.36 (dt, J = 16.5, 7.8 Hz, 4H), 7.24 – 7.17 (m, 2H), 7.13 – 7.06 (m, 8H), 7.03 (d, J = 7.0 Hz, 2H), 6.35 (s, 1H), 5.46 (s, 1H), 4.31 – 4.15 (m, 2H), 4.00 (dd, J = 9.4, 7.4 Hz, 1H), 3.78 (dd, J = 9.3, 5.4 Hz, 1H), 3.62 (dd, J = 18.9, 13.5 Hz, 2H), 3.51 (d, J = 13.2 Hz, 1H), 3.20 (dd, J = 17.9, 9.4 Hz, 1H), 3.03 (dd, J = 18.5, 9.7 Hz, 1H), 2.83 (s, 1H), 1.54 (s, 10H), 1.39 (s, 9H). ^{13}C NMR (101 MHz, $CDCl_3$) δ 174.72, 173.81, 173.62, 173.43, 173.01, 158.05, 157.32, 147.51, 147.25, 136.97, 136.78, 134.05, 133.97, 133.24, 132.43, 131.06, 131.00, 130.95, 130.74, 130.67, 129.44, 129.33, 129.20, 129.17, 128.98, 128.93, 128.87, 128.72, 128.46, 128.39, 127.97, 127.81, 127.64, 126.97, 126.37, 126.07, 124.19, 122.14, 120.02, 119.85, 103.92, 103.89, 61.88, 61.85, 57.10, 44.96, 43.68, 41.25, 40.00, 31.07, 30.05, 29.78, 29.65. ^{19}F NMR (376 MHz, $CDCl_3$) δ -62.62, -62.70. HRMS (ESI) m/z Calcd. for $C_{40}H_{35}F_3N_5O_3$, $[M+H]^+$, 690.2687, Found: 690.2694. Enantiomeric excess was determined to be 96% (determined by HPLC using chiral OD-H column, hexane/2-propanol = 8/2, λ = 254 nm, 25 °C, 0.8 mL/min, t_{major} = 34.9 min, t_{minor} = 16.9 min).

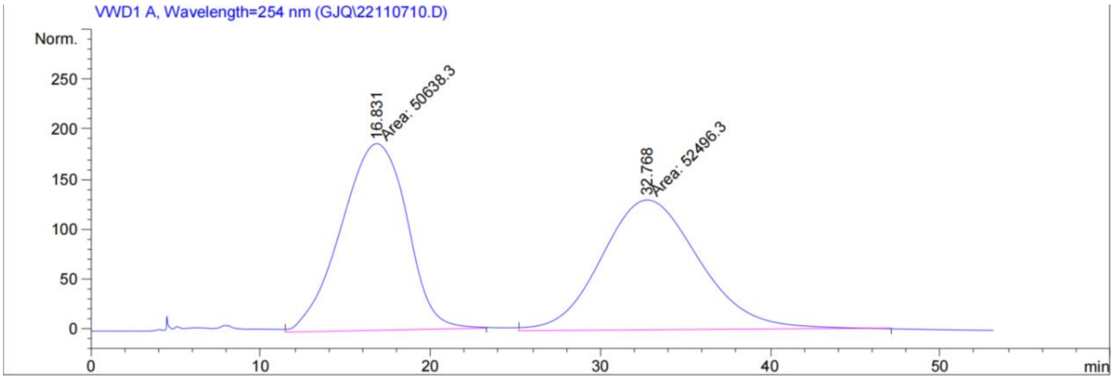

Peak #	RetTime [min]	Type	Width [min]	Area mAU *s	Height [mAU]	Area %
1	16.831	MM	4.5051	5.06383e4	187.33504	49.0993
2	32.768	MM	6.7110	5.24963e4	130.37428	50.9007

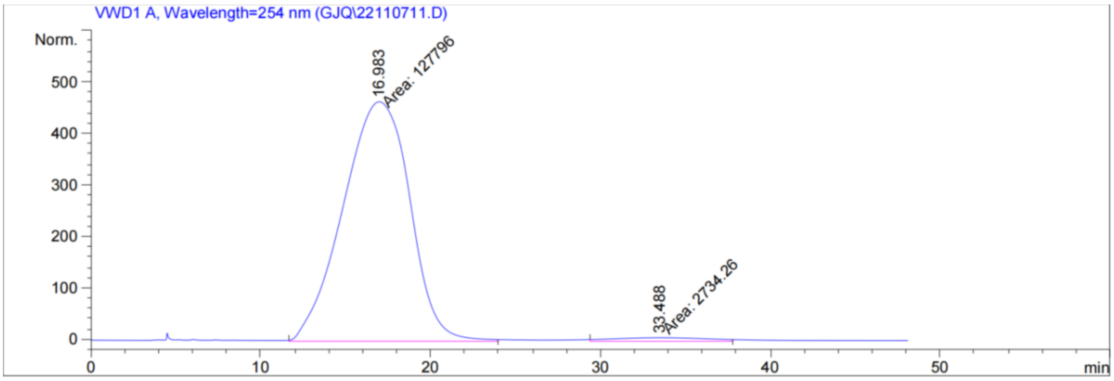

Peak #	RetTime [min]	Type	Width [min]	Area mAU *s	Height [mAU]	Area %
1	16.983	MM	4.5753	1.27796e5	465.53143	97.9053
2	33.488	MM	6.5821	2734.26123	6.92344	2.0947

(S)-3-((R)-4-benzyl-5-oxo-1,3-diphenyl-4,5-dihydro-1H-pyrazol-4-yl)-1-(1-(tert-butyl)-3-(3-methoxyphenyl)-1H-pyrazol-5-yl)pyrrolidine-2,5-dione (3ag)

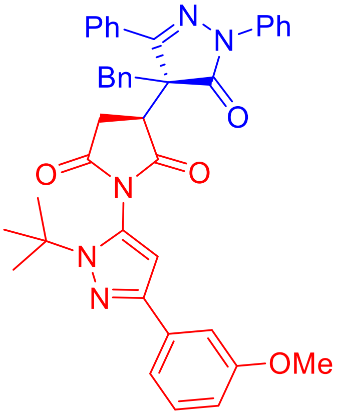

3ag

Prepared according to the procedure within 1 h as white solid (125.0 mg, 96% yield, dr = 1:1). mp 114.1 – 114.9 °C; $[\alpha]_D^{17}$ = -37.751 (c 0.25, CH_2Cl_2); ^{1}H NMR (400 MHz, Chloroform-d) δ 7.98 (d, J = 7.6 Hz, 4H), 7.67 – 7.46 (m, 10H), 7.34 (dt, J = 15.4, 7.7 Hz, 4H), 7.26 – 7.15 (m, 8H), 7.07 (d, J = 9.5 Hz, 8H), 7.00 (d, J = 7.5 Hz, 2H), 6.85 – 6.75 (m, 2H), 6.29 (s, 1H), 5.50 (s, 1H), 4.21 (q, J = 7.8, 7.3 Hz, 2H), 3.97 (t, J = 8.3 Hz, 1H), 3.80 (d, J = 21.3 Hz, 7H), 3.58 (dd, J = 13.6, 6.5 Hz, 2H), 3.47 (d, J = 13.2 Hz, 1H), 3.15 (dd, J = 17.8, 9.4 Hz, 1H), 3.00 (dd, J = 18.5, 9.6 Hz, 1H), 1.52 (s, 9H), 1.35 (s, 9H). ^{13}C NMR (101 MHz, $CDCl_3$) δ 174.88, 173.92, 173.79, 173.47, 173.10, 159.83, 159.78, 158.11, 157.40, 148.78, 148.50, 137.01, 136.82, 134.73, 134.63, 133.31, 132.53, 131.09, 131.04, 131.00, 130.73, 129.54, 129.44, 129.37, 129.19, 129.00, 128.85, 128.73, 128.45, 128.38, 127.94, 127.81, 127.60, 126.99, 126.34, 126.07, 120.04, 119.91, 118.07, 118.01, 113.42, 110.77, 103.90, 103.88, 61.55, 57.12, 55.29, 55.25, 44.94, 43.60, 41.23, 40.04, 31.05, 30.03, 29.82, 29.70. HRMS (ESI) m/z Calcd. for $C_{40}H_{38}N_5O_4$ $[M+H]^+$ 652.2918, Found: 652.2926. Enantiomeric excess was determined to be 97% (determined by HPLC using chiral, $[M+H]^+$, column, hexane/2-propanol = 8/2, λ = 254 nm, 25 °C, 0.8 mL/min, t_{major} = 43.0 min, t_{minor} = 23.3 min).

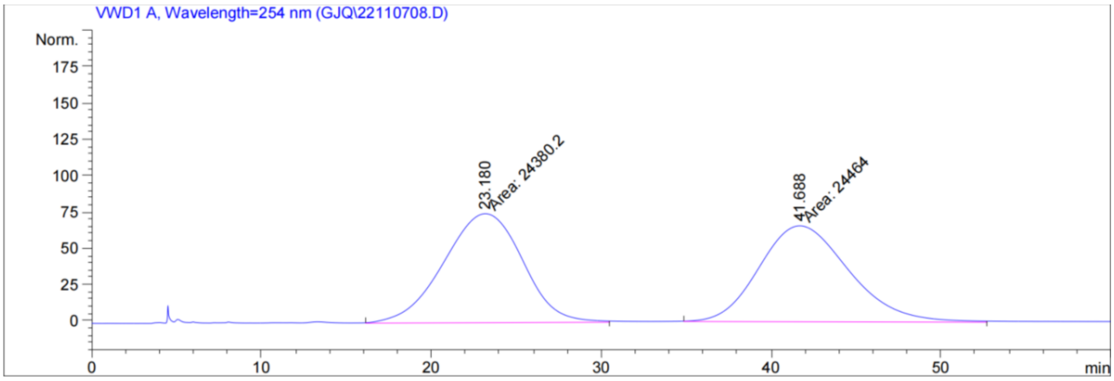

Peak #	RetTime [min]	Type	Width [min]	Area mAU *s	Height [mAU]	Area %
1	23.180	MM	5.4062	2.43802e4	75.16162	49.9141
2	41.688	MM	6.1597	2.44640e4	66.19364	50.0859

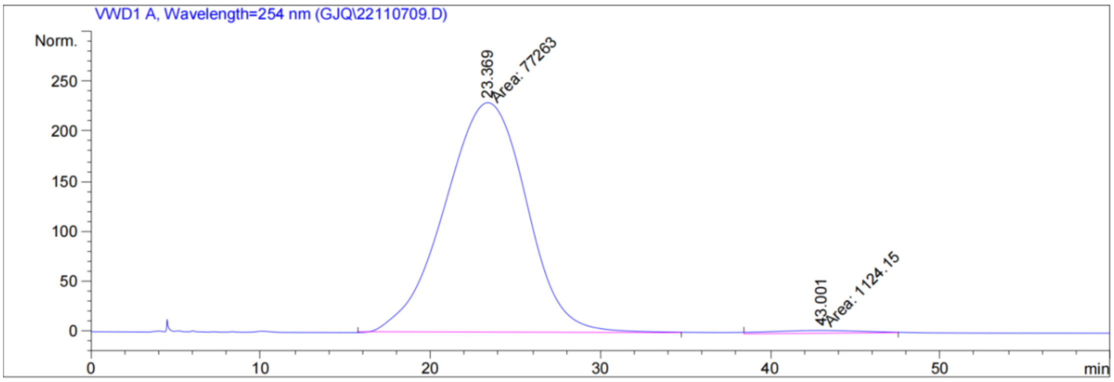

Peak #	RetTime [min]	Type	Width [min]	Area mAU *s	Height [mAU]	Area %
1	23.369	MM	5.5949	7.72630e4	230.15930	98.5659
2	43.001	MM	6.4655	1124.15320	2.89782	1.4341

(S)-3-((R)-4-benzyl-5-oxo-1,3-diphenyl-4,5-dihydro-1H-pyrazol-4-yl)-1-(1-(tert-butyl)-3-(4-fluorophenyl)-1H-pyrazol-5-yl)pyrrolidine-2,5-dione (3ah)

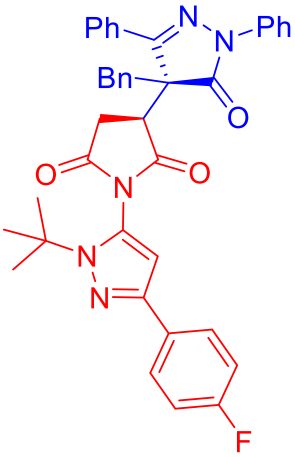

3ah

Prepared according to the procedure within 1 h as white solid (120.2 mg, 94% yield, dr = 1:1). mp 104.1 – 104.9 ºC; $[\alpha]_D^{17}$ = -24.525 (*c* 0.58, CH_2Cl_2); ^{1}H NMR (400 MHz, Chloroform-d) δ 7.99 (ddt, J = 6.7, 4.2, 1.9 Hz, 4H), 7.67 – 7.48 (m, 14H), 7.41 – 7.30 (m, 4H), 7.23 – 7.16 (m, 2H), 7.13 – 6.96 (m, 14H), 6.24 (s, 1H), 5.47 (s, 1H), 4.22 (dd, J = 17.9, 7.2 Hz, 2H), 3.99 (dd, J = 9.3, 7.4 Hz, 1H), 3.81 – 3.72 (m, 1H), 3.61 (dd, J = 18.5, 13.5 Hz, 2H), 3.49 (d, J = 13.2 Hz, 1H), 3.18 (dd, J = 17.8, 9.4 Hz, 1H), 3.02 (dd, J = 18.4, 9.6 Hz, 1H), 1.52 (s, 9H), 1.37 (s, 9H). ^{13}C NMR (151 MHz, $CDCl_3$) δ 173.86, 173.71, 173.42, 173.09, 173.04, 163.36, 163.32, 161.72, 161.69, 158.05, 157.35, 148.07, 147.79, 136.97, 136.82, 133.22, 132.45, 131.08, 131.01, 130.95, 130.72, 129.55, 129.48, 129.39, 129.34, 129.17, 128.98, 128.89, 128.71, 128.57, 128.44, 128.39, 127.96, 127.81, 127.56, 127.07, 127.01, 126.99, 126.96, 126.28, 126.06, 120.02, 119.88, 115.37 (d, J= 15.15Hz), 103.47, 103.43, 61.55, 57.11, 44.93, 41.24, 40.04, 31.03, 30.04, 29.79, 29.66. ^{19}F NMR (376 MHz, $CDCl_3$) δ -114.70, -114.83. HRMS (ESI) m/z Calcd. for $C_{39}H_{35}FN_5O_3$, $[M+H]^+$, 640.2718, Found: 640.2731. Enantiomeric excess was determined to be 97% (determined by HPLC using chiral OD-H column, hexane/2-propanol = 8/2, λ = 254 nm, 25 ºC, 0.8 mL/min, t_{major} = 34.2 min, t_{minor} = 18.0 min).

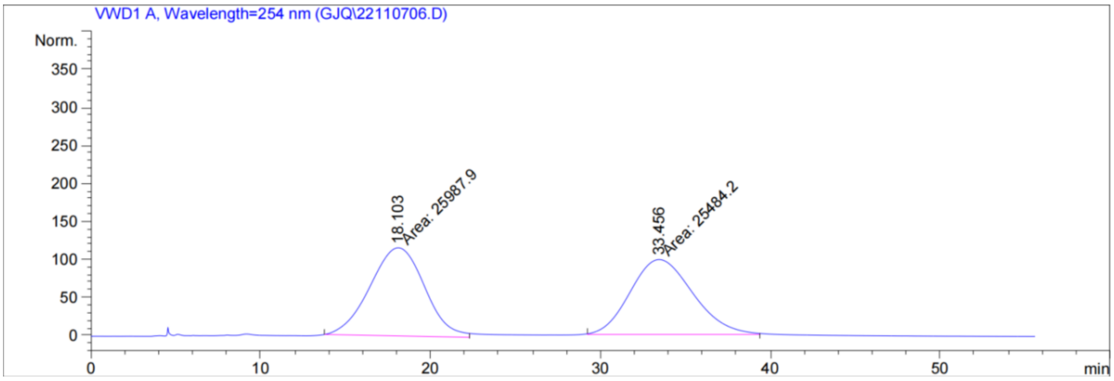

Peak #	RetTime [min]	Type	Width [min]	Area mAU *s	Height [mAU]	Area %
1	18.103	MM	3.7317	2.59879e4	116.06711	50.4893
2	33.456	MM	4.2994	2.54842e4	98.78864	49.5107

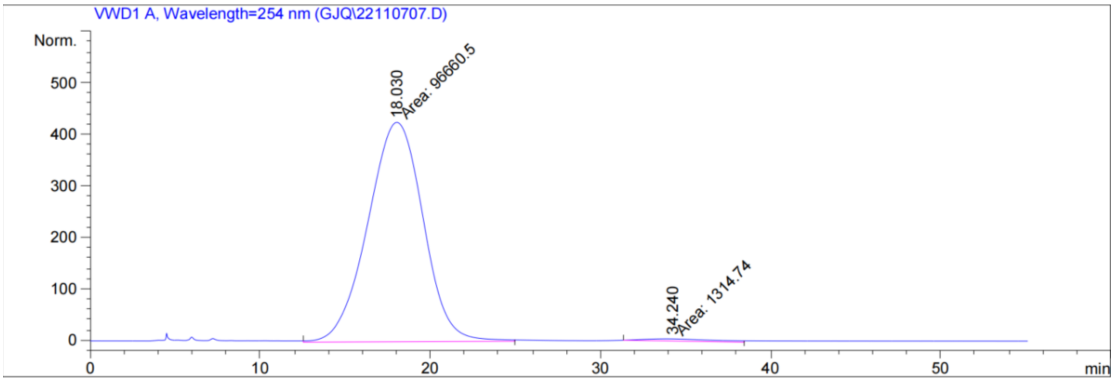

Peak #	RetTime [min]	Type	Width [min]	Area mAU *s	Height [mAU]	Area %
1	18.030	MM	3.7800	9.66605e4	426.18832	98.6581
2	34.240	MM	5.0129	1314.73633	4.37118	1.3419

(S)-3-((R)-4-benzyl-5-oxo-1,3-diphenyl-4,5-dihydro-1H-pyrazol-4-yl)-1-(1-(tert-butyl)-3-(4-chlorophenyl)-1H-pyrazol-5-yl)pyrrolidine-2,5-dione (3ai)

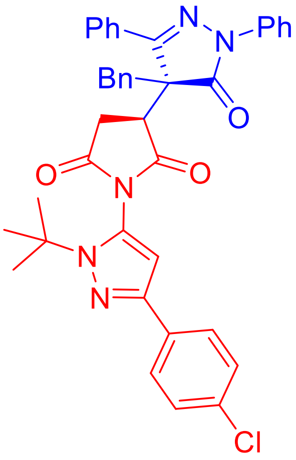

3ai

Prepared according to the procedure within 1 h as white solid (125.8 mg, 96% yield, dr = 1:1). mp 101.1 – 101.9 °C; $[\alpha]_D^{17}$ = -29.557 (*c* 0.77, CH_2Cl_2); ^{1}H NMR (400 MHz, Chloroform-d) δ 8.07 – 8.00 (m, 4H), 7.71 – 7.66 (m, 2H), 7.65 – 7.52 (m, 12H), 7.43 – 7.29 (m, 8H), 7.27 – 7.18 (m, 2H), 7.11 (q, J = 6.8, 5.1 Hz, 8H), 7.09 – 7.02 (m, 2H), 6.30 (s, 1H), 5.51 (s, 1H), 4.26 (d, J = 25.9 Hz, 2H), 4.02 (dd, J = 9.3, 7.4 Hz, 1H), 3.83 – 3.75 (m, 1H), 3.65 (dd, J = 21.6, 13.5 Hz, 2H), 3.53 (d, J = 13.2 Hz, 1H), 3.22 (dd, J = 17.8, 9.4 Hz, 1H), 3.05 (dd, J = 18.4, 9.6 Hz, 1H), 2.87 (s, 1H), 1.55 (s, 9H), 1.40 (s, 9H). ^{13}C NMR (101 MHz, $CDCl_3$) δ 174.77, 173.84, 173.66, 173.41, 173.06, 173.03, 158.04, 157.34, 147.84, 147.55, 136.97, 136.82, 133.36, 133.30, 133.21, 132.44, 131.83, 131.76, 131.08, 131.02, 130.94, 130.72, 129.38, 129.33, 129.17, 128.98, 128.71, 128.64, 128.44, 128.39, 127.96, 127.81, 127.55, 126.98, 126.62, 126.57, 126.28, 126.06, 103.66, 103.63, 61.68, 57.10, 44.93,

43.70, 41.24, 40.04, 31.02, 30.04, 29.78, 29.65. HRMS (ESI) m/z Calcd. for $C_{39}H_{35}ClN_5O_3$, $[M+H]^+$, 656.2423, Found: 656.2433. Enantiomeric excess was determined to be 98% (determined by HPLC using chiral OD-H column, hexane/2-propanol = 8/2, λ = 254 nm, 25 °C, 0.8 mL/min, t_{major} = 34.4 min, t_{minor} = 18.5 min).

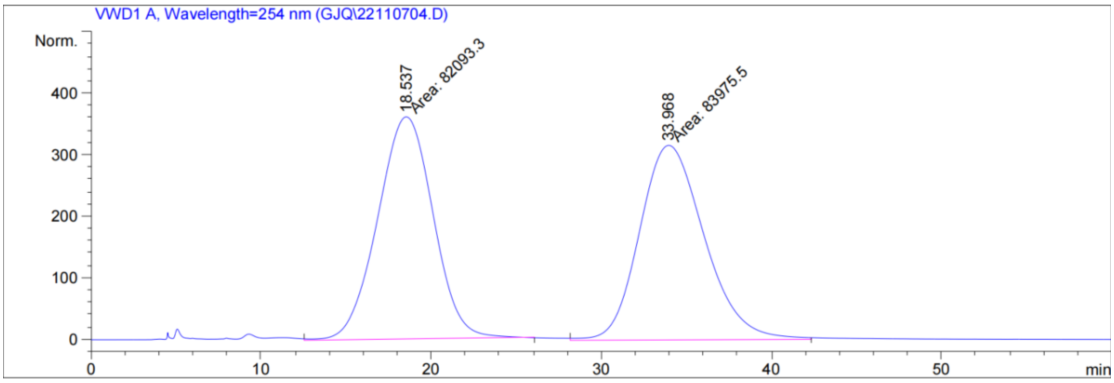

Peak #	RetTime [min]	Type	Width [min]	Area mAU *s	Height [mAU]	Area %
1	18.537	MM	3.7862	8.20933e4	361.36658	49.4333
2	33.968	MM	4.4218	8.39755e4	316.52121	50.5667

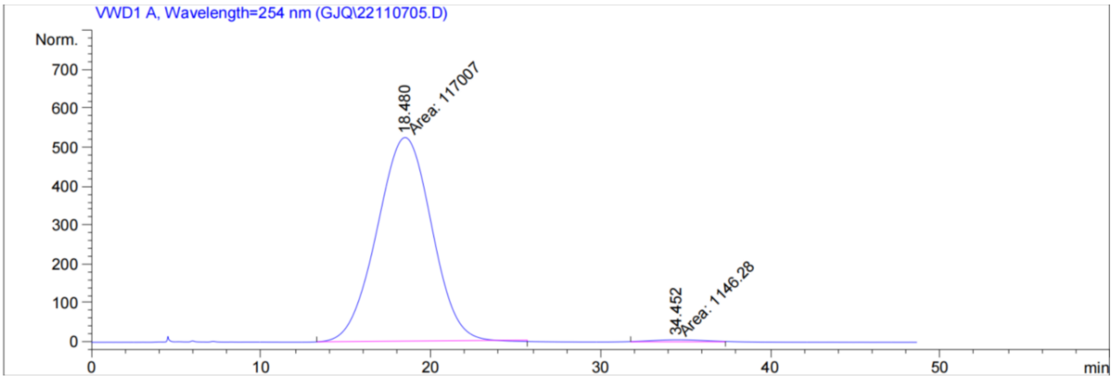

Peak #	RetTime [min]	Type	Width [min]	Area mAU *s	Height [mAU]	Area %
1	18.480	MM	3.7272	1.17007e5	523.21790	99.0298
2	34.452	MM	3.6800	1146.27747	5.19148	0.9702

(S)-3-((R)-4-benzyl-5-oxo-1,3-diphenyl-4,5-dihydro-1H-pyrazol-4-yl)-1-(3-(4-bromophenyl)-1-(tert-butyl)-1H-pyrazol-5-yl)pyrrolidine-2,5-dione (3aj)

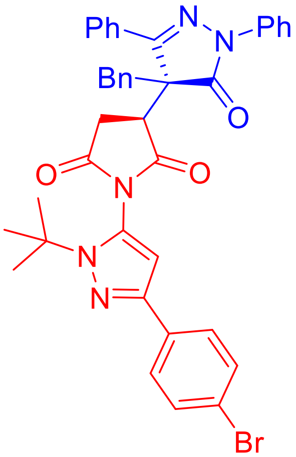

3aj

Prepared according to the procedure within 1 h as white solid (130.0 mg, 93% yield, dr = 1:1). mp 126.1 – 126.9 ºC; $[\alpha]_D^{17}$ = -27.602 (c 0.22, CH_2Cl_2); ^{1}H NMR (400 MHz, Chloroform-d) δ 7.99 (ddt, J = 6.9, 5.4, 2.5 Hz, 4H), 7.68 – 7.62 (m, 2H), 7.61 – 7.49 (m, 10H), 7.48 – 7.30 (m, 10H), 7.24 – 7.16 (m, 2H), 7.14 – 6.99 (m, 10H), 6.27 (s, 1H), 5.49 (s, 1H), 4.23 (dd, J = 18.1, 7.4 Hz, 2H), 3.99 (dd, J = 9.4, 7.4 Hz, 1H), 3.82 – 3.75 (m, 1H), 3.61 (dd, J = 20.1, 13.5 Hz, 2H), 3.50 (d, J = 13.2 Hz, 1H), 3.19 (dd, J = 17.9, 9.4 Hz, 1H), 3.02 (dd, J = 18.3, 9.6 Hz, 1H), 2.84 (s, 1H), 1.52 (s, 9H), 1.37 (s, 9H). ^{13}C NMR (101 MHz, $CDCl_3$) δ 173.83, 173.65, 173.41, 173.05, 173.02, 158.03, 157.33, 147.85, 147.55, 136.96, 136.81, 133.20, 132.43, 132.27, 132.20, 131.58, 131.07, 131.02, 130.94, 130.72, 129.38, 129.33, 129.17, 128.98, 128.71, 128.68, 128.44, 128.39, 127.96, 127.81, 127.54, 126.97, 126.93, 126.89, 126.28, 126.06, 121.55, 121.49, 120.02, 119.87, 103.66, 103.63, 61.70, 57.10, 44.93, 43.69, 41.24, 40.04, 31.02, 30.04, 29.78, 29.65. HRMS (ESI) m/z Calcd. for $C_{39}H_{35}BrN_5O_3$, $[M+H]^+$, 700.1918, Found: 700.1924. Enantiomeric excess was determined to be 97% (determined by HPLC using chiral OD-H column, hexane/2-propanol = 8/2, λ = 254 nm, 25 ºC, 0.8 mL/min, t_{major} = 36.0 min, t_{minor} = 19.1 min).

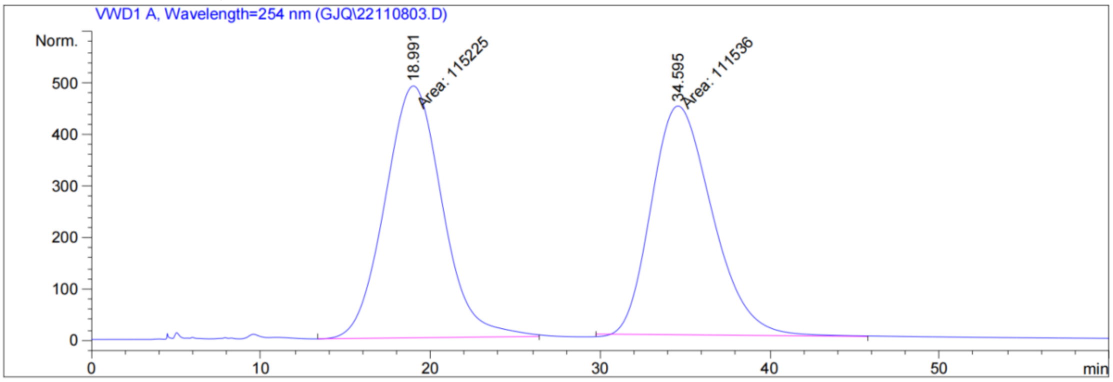

Peak #	RetTime [min]	Type	Width [min]	Area mAU *s	Height [mAU]	Area %
1	18.991	MM	3.9216	1.15225e5	489.70486	50.8132
2	34.595	MM	4.1777	1.11536e5	444.97040	49.1868

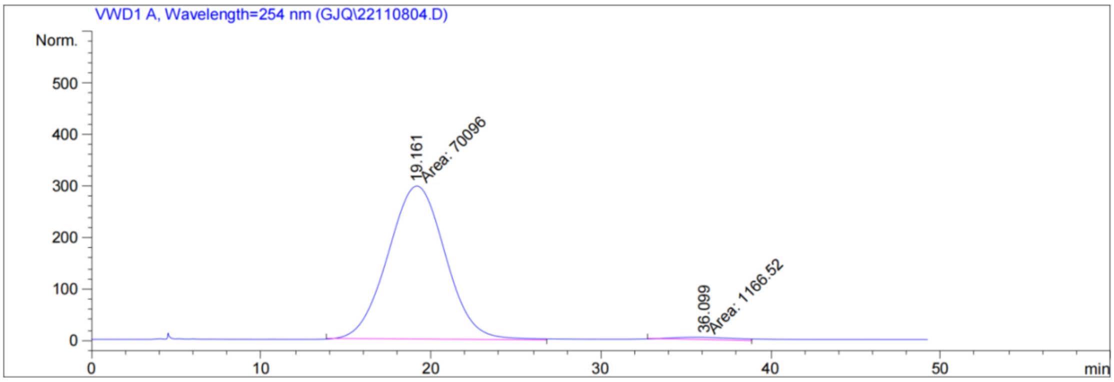

Peak #	RetTime [min]	Type	Width [min]	Area mAU *s	Height [mAU]	Area %
1	19.161	MM	3.9293	7.00960e4	297.32370	98.3631
2	36.099	MM	4.2089	1166.51892	4.61926	1.6369

(S)-3-((R)-4-benzyl-5-oxo-1,3-diphenyl-4,5-dihydro-1H-pyrazol-4-yl)-1-(1-(tert-butyl)-3-(p-tolyl)-1H-pyrazol-5-yl)pyrrolidine-2,5-dione (3ak)

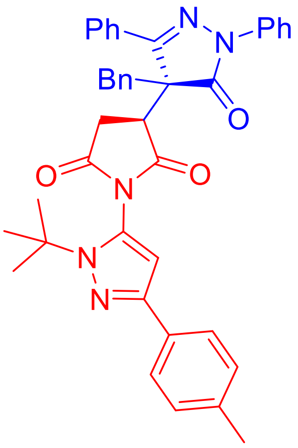

3ak

Prepared according to the procedure within 1 h as white solid (120.7 mg, 95% yield, dr = 1:1). mp 125.1 – 125.9 ºC; $[\alpha]_D^{17}$ = -39.223 (*c* 0.28, CH_2Cl_2); ^1H NMR (400 MHz, Chloroform-d) δ 8.01 – 7.92 (m, 4H), 7.67 – 7.62 (m, 2H), 7.62 – 7.45 (m, 12H), 7.33 (dt, J = 16.2, 7.9 Hz, 4H), 7.23 – 7.17 (m, 2H), 7.17 – 7.09 (m, 5H), 7.06 (d, J = 11.2 Hz, 7H), 6.99 (dd, J = 7.9, 1.8 Hz, 2H), 6.26 (s, 1H), 5.49 (s, 1H), 4.24 – 4.12 (m, 2H), 3.94 (dd, J = 9.3, 7.3 Hz, 1H), 3.75 (dd, J = 8.9, 5.8 Hz, 1H), 3.57 (d, J = 14.2 Hz, 2H), 3.45 (d, J = 13.2 Hz, 1H), 3.12 (dd, J = 17.8, 9.4 Hz, 1H), 2.98 (dd, J = 18.4, 9.6 Hz, 1H), 2.33 (s, 3H), 2.29 (s, 3H), 1.51 (s, 9H), 1.37 (s, 9H). ^{13}C NMR (101 MHz, $CDCl_3$) δ 174.89, 173.95, 173.82, 173.48, 173.17, 173.12, 158.13, 157.45, 149.05, 148.77, 137.39, 137.32, 137.05, 136.88, 133.35, 132.58, 131.14, 131.03, 130.71, 130.61, 130.53, 129.42, 129.39, 129.21, 129.18, 129.01, 128.78, 128.73, 128.45, 128.42, 128.38, 127.93, 127.81, 127.59, 127.02, 126.31, 126.06, 125.34, 125.29, 120.06, 119.92, 103.47, 61.41, 57.15, 44.95, 43.67, 41.23, 40.06, 31.04, 30.03, 29.83, 29.73, 21.34, 21.30. HRMS (ESI) m/z Calcd. for $C_{40}H_{38}N_5O_3$, $[M+H]^+$, 636.2969, Found: 636.2976. Enantiomeric excess was determined to be 99% (determined by HPLC using chiral OD-H column, hexane/2-propanol = 8/2, λ = 254 nm, 25 ºC, 0.8 mL/min, t_{major} = 31.7 min, t_{minor} = 16.6 min).

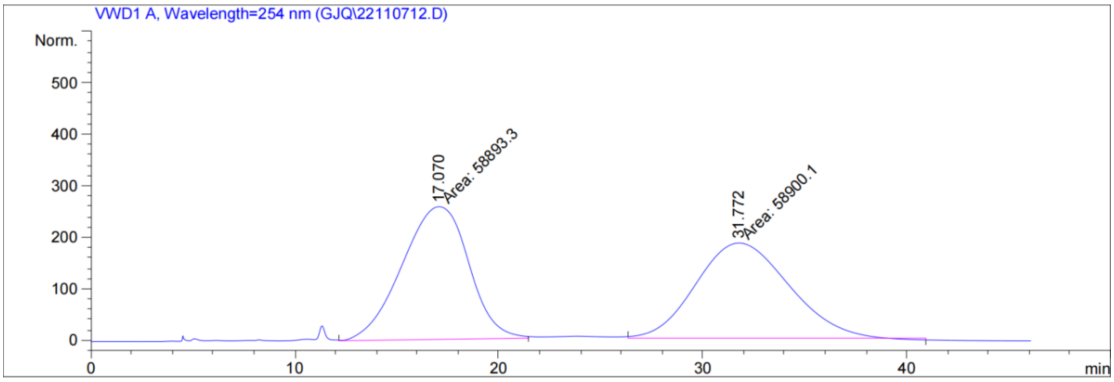

Peak #	RetTime [min]	Type	Width [min]	Area mAU *s	Height [mAU]	Area %
1	17.070	MM	3.8044	5.88933e4	258.00790	49.9971
2	31.772	MM	5.3084	5.89001e4	184.92781	50.0029

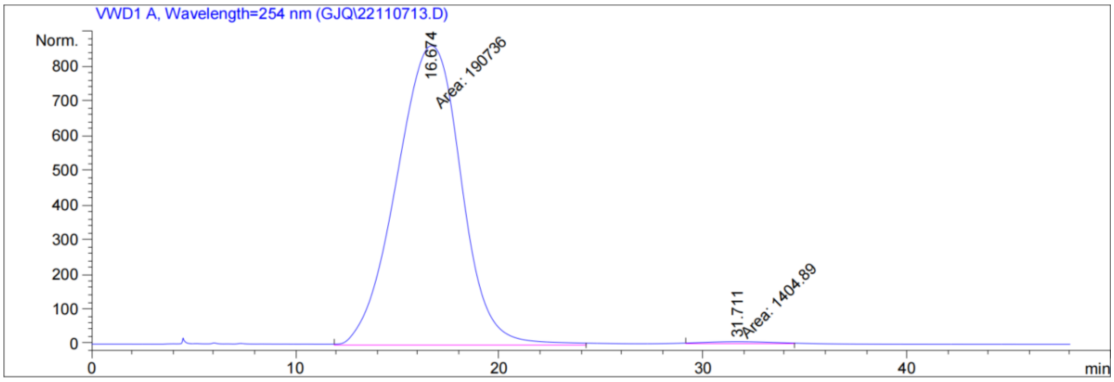

Peak #	RetTime [min]	Type	Width [min]	Area mAU *s	Height [mAU]	Area %
1	16.674	MM	3.6831	1.90736e5	863.11517	99.2688
2	31.711	MM	3.9622	1404.89136	5.90956	0.7312

(S)-1-(4-bromo-1-(tert-butyl)-3-phenyl-1H-pyrazol-5-yl)-3-((R)-4-(2-bromobenzyl)-5-oxo-1,3-diphenyl-4,5-dihydro-1H-pyrazol-4-yl)pyrrolidine-2,5-dione (4)

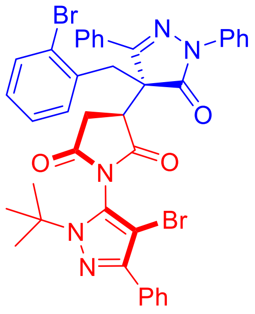

4

Prepared according to the procedure within 96 h as white solid (106.1mg, 58% yield, dr > 20:1). mp 126.1–126.9 °C; $[\alpha]_D^{17}$ = -22.000 (*c* 0.50, CH_2Cl_2); ^1H NMR (400 MHz, Chloroform-d) δ 7.95 (dd, J = 6.8, 2.9 Hz, 2H), 7.79 (dd, J = 11.0, 7.5 Hz, 4H), 7.49 (dd, J = 6.4, 3.6 Hz, 4H), 7.38 (q, J = 7.6 Hz, 4H), 7.31 (dd, J = 7.3 Hz, 1H), 7.21 (s, 1H), 7.14 – 6.99 (m, 3H), 4.19 – 4.02 (m, 2H), 3.92 (d, J = 14.4 Hz, 1H), 3.82 (d, J = 14.6 Hz, 1H), 3.24 (dd, J = 17.2, 8.7 Hz, 1H), 1.30 (s, 9H). ^{13}C NMR (101 MHz, $CDCl_3$) δ 172.93, 172.85, 172.68, 158.49, 145.99, 137.17, 133.37, 132.98, 132.06, 130.84, 130.68, 130.20, 129.46, 129.10, 128.85, 128.38, 128.29, 128.17, 127.58, 127.30, 126.03, 125.46, 119.70, 93.34, 62.45, 56.44, 45.18, 39.06, 30.28, 29.33. HRMS (ESI) m/z Calcd. for $C_{39}H_{34}Br_2N_5O_3$, $[M+H]^+$, 778.1023, Found: 778.1022. Enantiomeric excess was determined to be 96% (determined by HPLC using chiral IB-H column, hexane/2-propanol = 9/1, λ = 254 nm, 25 °C, 0.8 mL/min, t_{major} = 30.3 min, t_{minor} = 26.8 min).

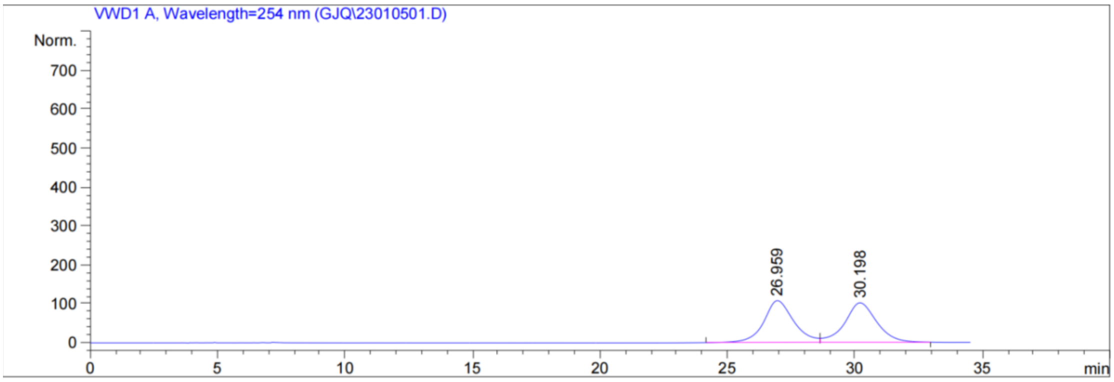

Peak #	RetTime [min]	Type	Width [min]	Area mAU *s	Height [mAU]	Area %
1	26.959	BV	1.2117	8816.33691	107.48934	49.8292
2	30.198	VB	1.2962	8876.78613	101.57886	50.1708

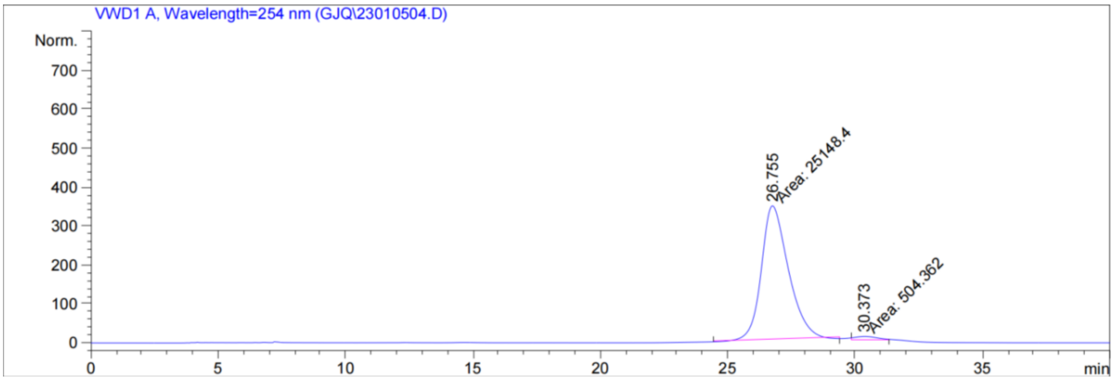

Peak #	RetTime [min]	Type	Width [min]	Area mAU *s	Height [mAU]	Area %
1	26.755	MM	1.2241	2.51484e4	342.39932	98.0339
2	30.373	MM	0.9887	504.36154	8.50242	1.9661

4-(1-(1-(tert-butyl)-3-phenyl-1H-pyrazol-5-yl)-2,5-dioxopyrrolidin-3-yl)-1,3-diphenyl-1H-pyrazol-5-yl acetate (6)

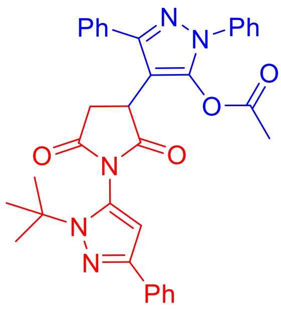

6

Prepared according to the procedure within 18 h as white solid (68.8 mg, 60% yield, dr = 6:1). mp 126.1–126.9 °C; ^{1}H NMR (600 MHz, Chloroform-d) δ 7.77 (d, J = 7.6 Hz, 2H), 7.69 (d, J = 7.3 Hz, 2H), 7.59 (d, J = 7.9 Hz, 2H), 7.50 (q, J = 8.1 Hz, 5H), 7.39 (dd, J = 7.5 Hz, 4H), 6.33 (s, 1H), 4.21 (dd, J = 10.2, 5.2 Hz, 1H), 3.25 (dd, J = 18.8, 10.1 Hz, 1H), 3.04 (dd, J = 18.7, 5.3 Hz, 1H), 2.26 (s, 3H), 1.60 (s, 9H). ^{13}C NMR (101 MHz, $CDCl_3$) δ 175.53, 174.64, 167.28, 151.32, 149.07, 141.74, 137.56, 133.39, 132.65, 129.39, 128.96, 128.94, 128.79, 128.55, 128.22, 127.74, 125.47, 123.50, 104.06, 103.44, 61.51, 36.50, 29.92, 20.56. HRMS (ESI) m/z Calcd. for $C_{34}H_{32}N_5O_4$, $[M+H]^+$, 574.2449, Found: 574.2455.

Gram-scale reaction

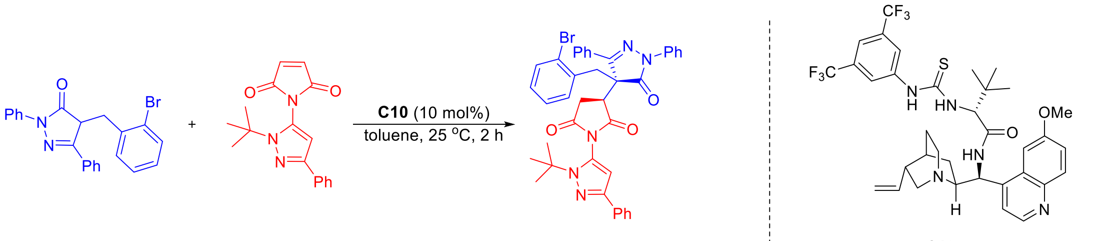

1i 1.8 mmol, 0.73 g | **2a** 1.5 mmol, 0.44 g | **3ia** 0.96 g, 91% yield, 1:1 dr, 96% ee | **C10**

In a reaction tube, 4-(2-bromobenzyl)-2,5-diphenyl-2,4-dihydro-3H-pyrazol-3-one **1i** (1.8 mmol), **2a** (1.5 mmol) were added into toluene (30 mL). The reaction solution was stirred at 25 °C. After the reaction was complete (monitored by TLC), the crude product was purified by column chromatography (ethyl acetate/petroleum ether = 1/10 to 1/3) on silica gel to give the product **3ia** with 91% yield with 1:1 dr and 96% ee.

Procedure for the Synthesis of 4

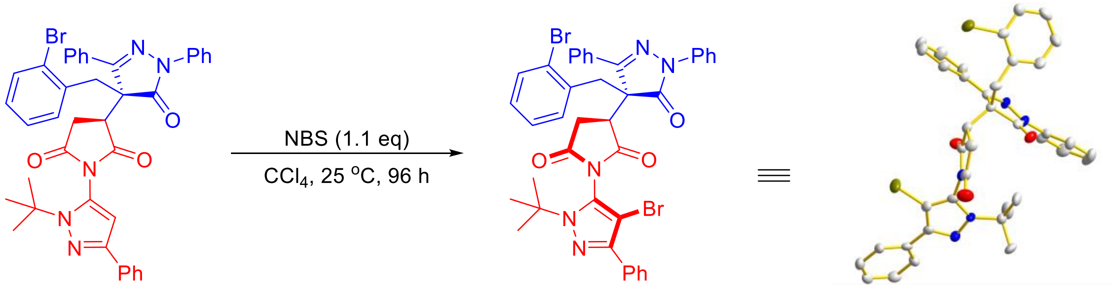

3ia 1:1 dr, 96% ee | **4** 58 % yield, > 20:1 dr, 96% ee | X-ray cyrstal structure of **4** CCDC 2253310

A solution of **3ia** (70 mg, 0.1 mmol) in CCl_4 was stirred at 0 °C, and NBS (20 mg, 0.11 mmol) was added. The reaction was stirred at 25 °C until it was complete (monitored by TLC), then the crude product was purified by column chromatography (ethyl acetate/petroleum ether = 1/10) on silica gel to give the product **4** as a white solid.

Procedure for the Synthesis of 6

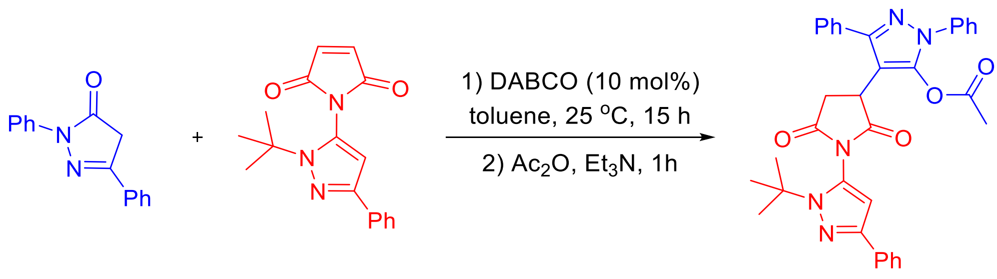

5 **2a** **6**

60% yield, 6:1 dr

In a reaction tube, 2,5-diphenyl-2,4-dihydro-3H-pyrazol-3-one **5** (0.24 mmol), 2a (0.2 mmol) were added into toluene (4 mL). The reaction solution was stirred for 15 hours at 25 °C. Subsequently, the Ac_2O (0.3 mmol) and Et_3N (0.04 mmol) were added into the reaction solution. After the reaction was complete (monitored by TLC), the crude product was purified by column chromatography (ethyl acetate/petroleum ether = 1/8 to 1/3) on silica gel to give the product **6** with 60% yield and 6:1 dr.

5. X-ray crystal structure of 4

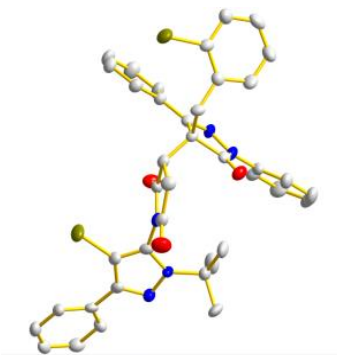

X-ray cyrstal structure of **4**
CCDC 2253310

Table 1 Crystal data and structure refinement for 1.

Identification code	1
Empirical formula	$C_{39}H_{33}Br_2N_5O_3$
Formula weight	779.52
Temperature/K	202.00
Crystal system	orthorhombic
Space group	$P2_12_12_1$
a/Å	9.6462(12)
b/Å	17.850(2)
c/Å	20.055(3)
α/°	90
β/°	90
γ/°	90
Volume/Å^3	3453.1(8)

Z	4
$\rho_{calc}g/cm^3$	1.499
μ/mm^{-1}	2.394
F(000)	1584.0
Crystal size/mm^3	0.3 × 0.25 × 0.22
Radiation	MoKα (λ = 0.71073)
2Θ range for data collection/°	4.564 to 50
Index ranges	$-11 \leq h \leq 11$, $-21 \leq k \leq 21$, $-23 \leq l \leq 23$
Reflections collected	41191
Independent reflections	6057 [R_{int} = 0.0730, R_{sigma} = 0.0544]
Data/restraints/parameters	6057/0/445
Goodness-of-fit on F^2	1.036
Final R indexes [I>=2σ (I)]	R_1 = 0.0458, wR_2 = 0.1130
Final R indexes [all data]	R_1 = 0.0594, wR_2 = 0.1182
Largest diff. peak/hole / e $Å^{-3}$	0.69/-0.48
Flack parameter	0.033(5)

6. Copies of ^{1}H NMR and ^{13}C NMR spectra

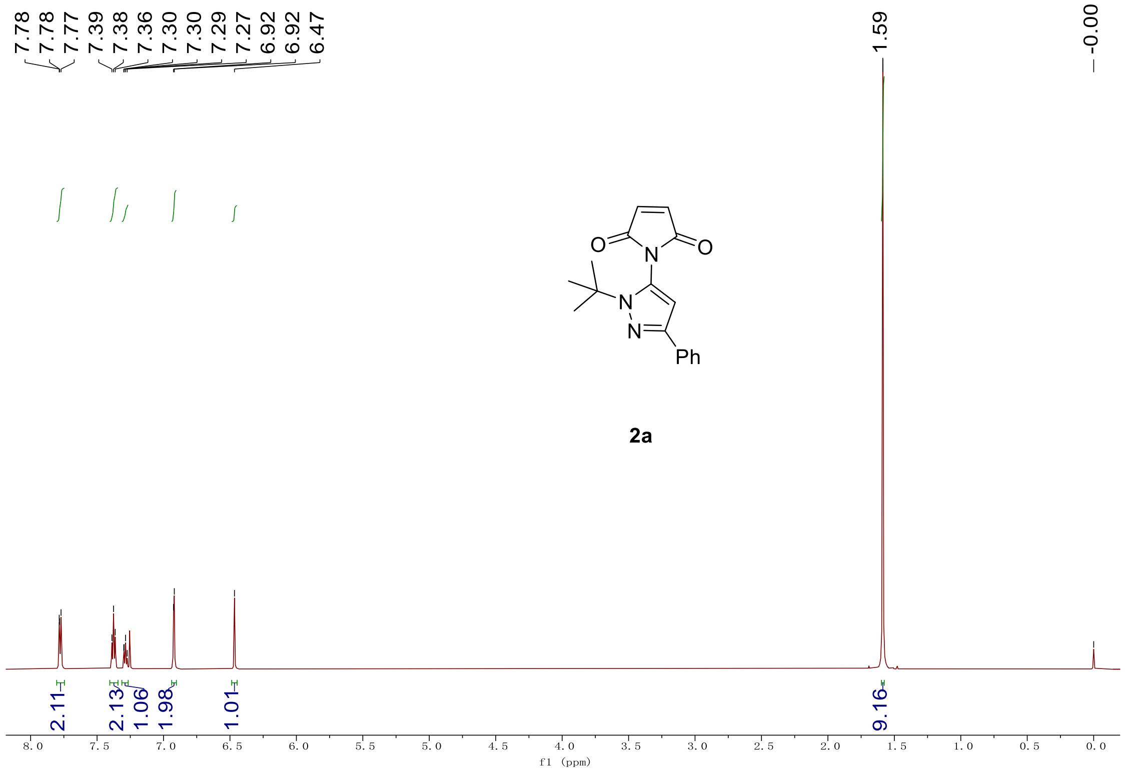

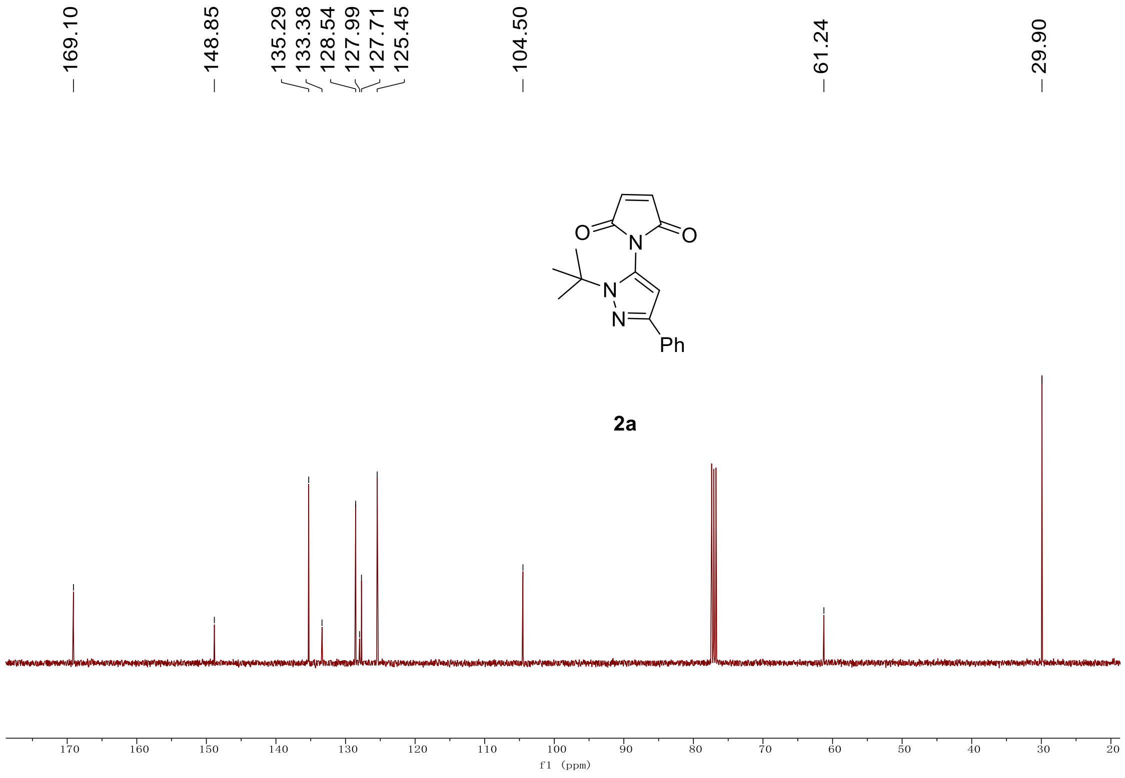

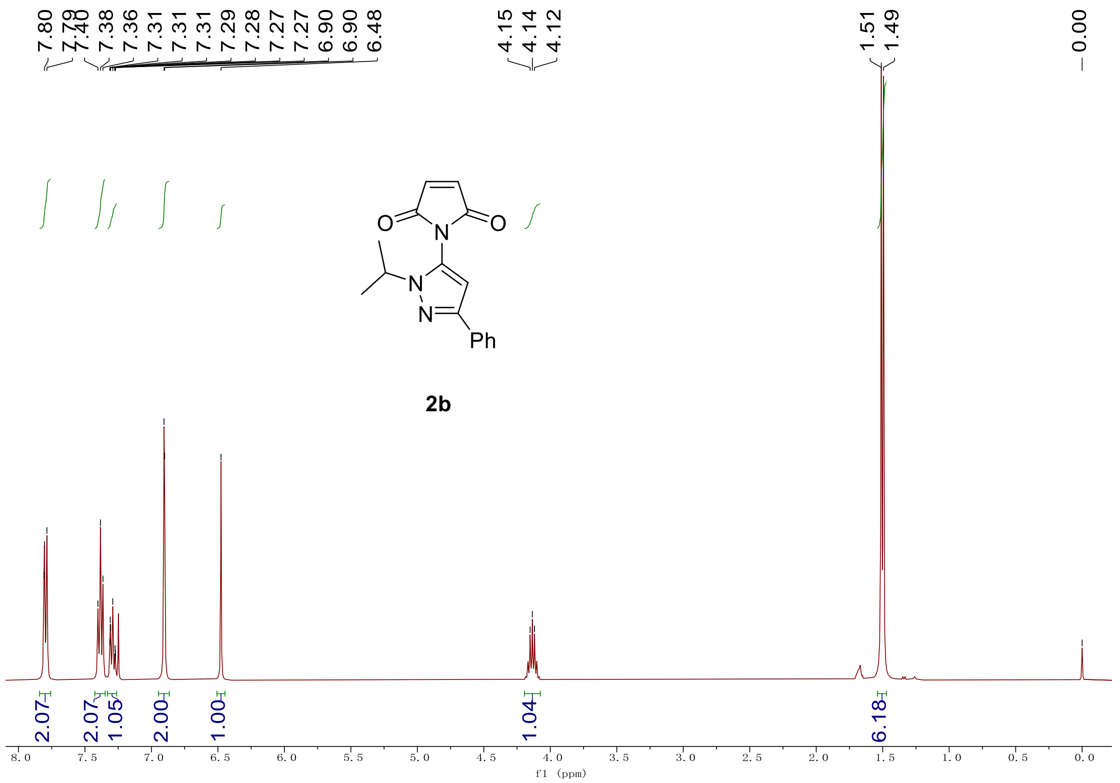
7.80
7.79
7.40
7.38
7.36
7.31
7.31
7.31
7.29
7.28
7.27
7.27
6.90
6.90
6.48
4.15
4.14
4.12
1.51
1.49
0.00
Ph
2b
2.07
2.07
1.05
2.00
1.00
1.04
6.18
f1 (ppm)

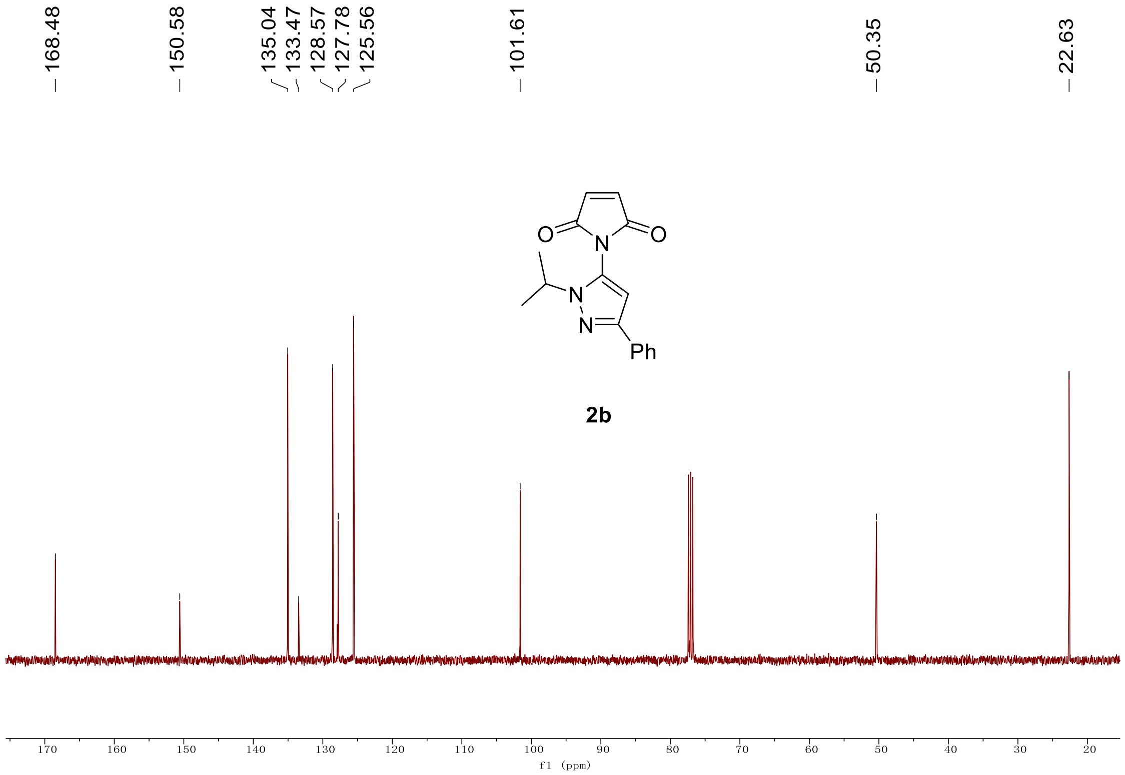
168.48
150.58
135.04
133.47
128.57
127.78
125.56
101.61
50.35
22.63
Ph
2b
f1 (ppm)

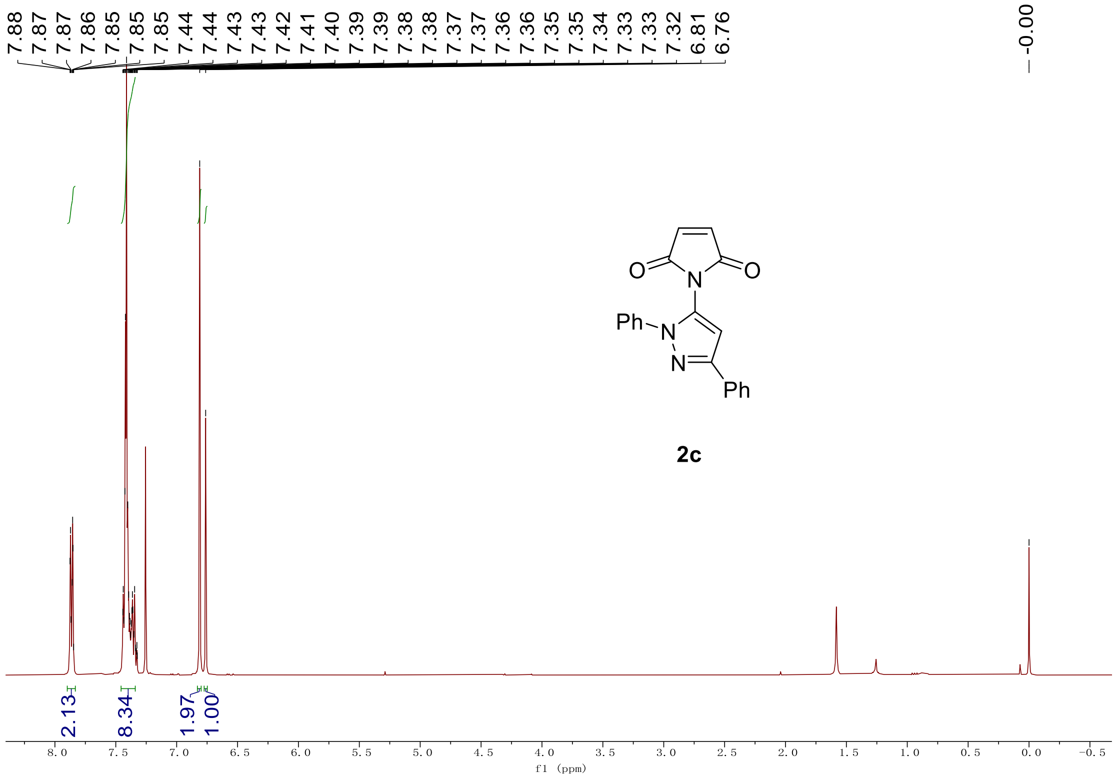
7.88
7.87
7.87
7.86
7.85
7.85
7.85
7.44
7.44
7.43
7.43
7.42
7.41
7.40
7.39
7.39
7.38
7.38
7.37
7.37
7.36
7.36
7.35
7.35
7.34
7.33
7.33
7.32
6.81
6.76
-0.00
Ph
N
N
Ph
O
N
O
2c
2.13
8.34
1.97
1.00
f1 (ppm)

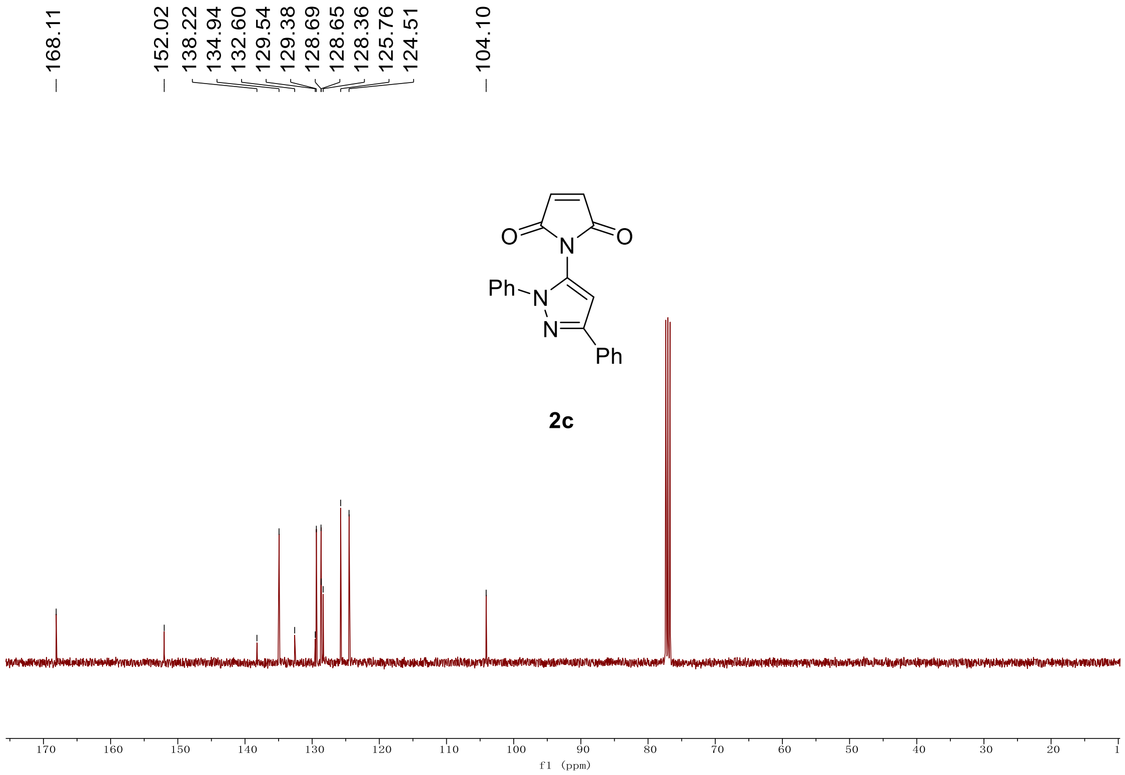
168.11
152.02
138.22
134.94
132.60
129.54
129.38
128.69
128.65
128.36
125.76
124.51
104.10
Ph
N
N
Ph
O
N
O
2c
f1 (ppm)

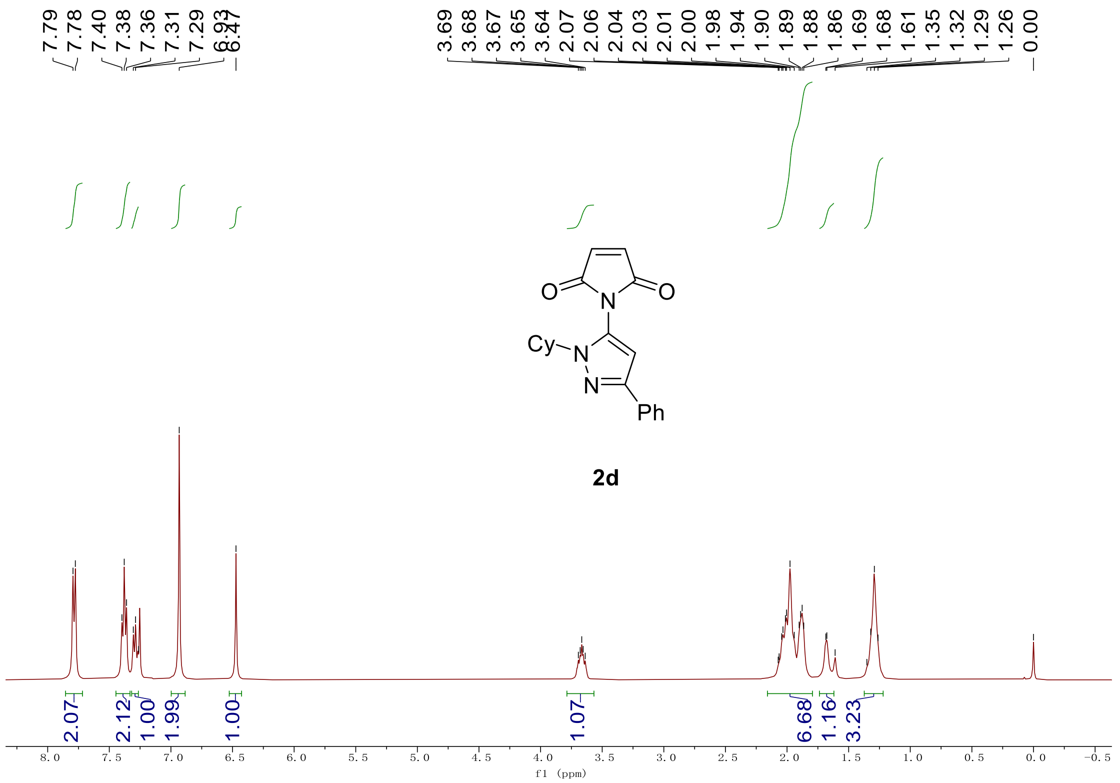

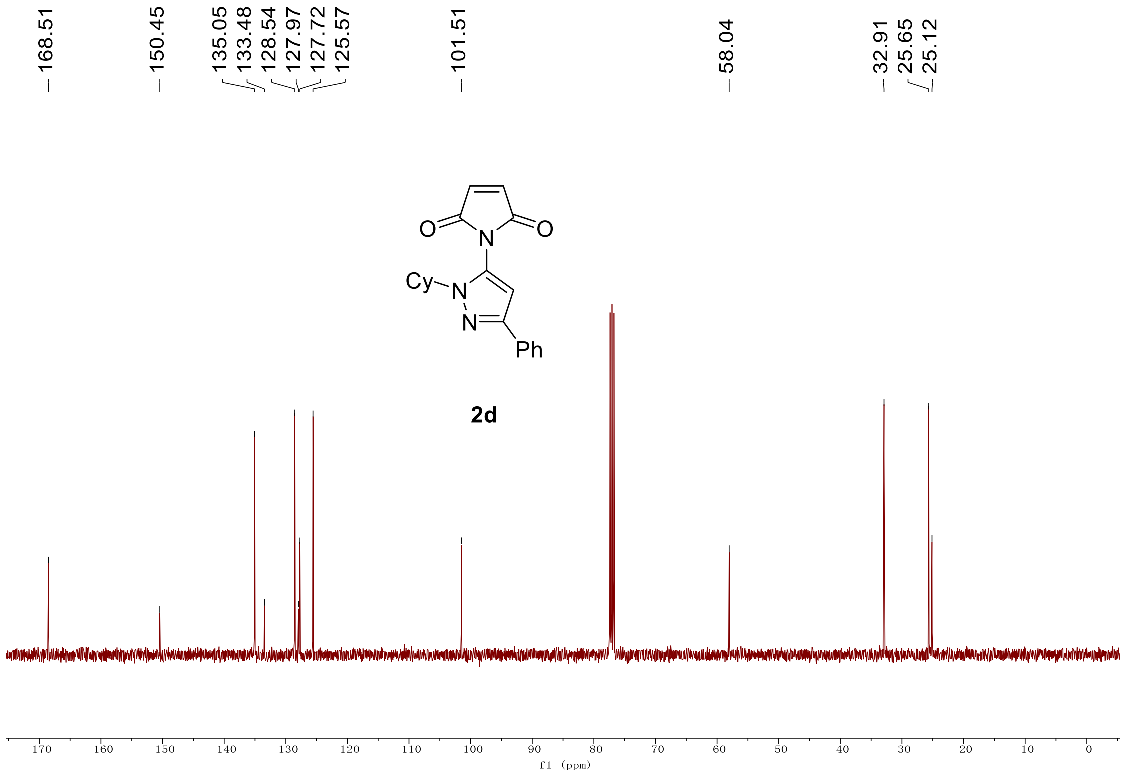

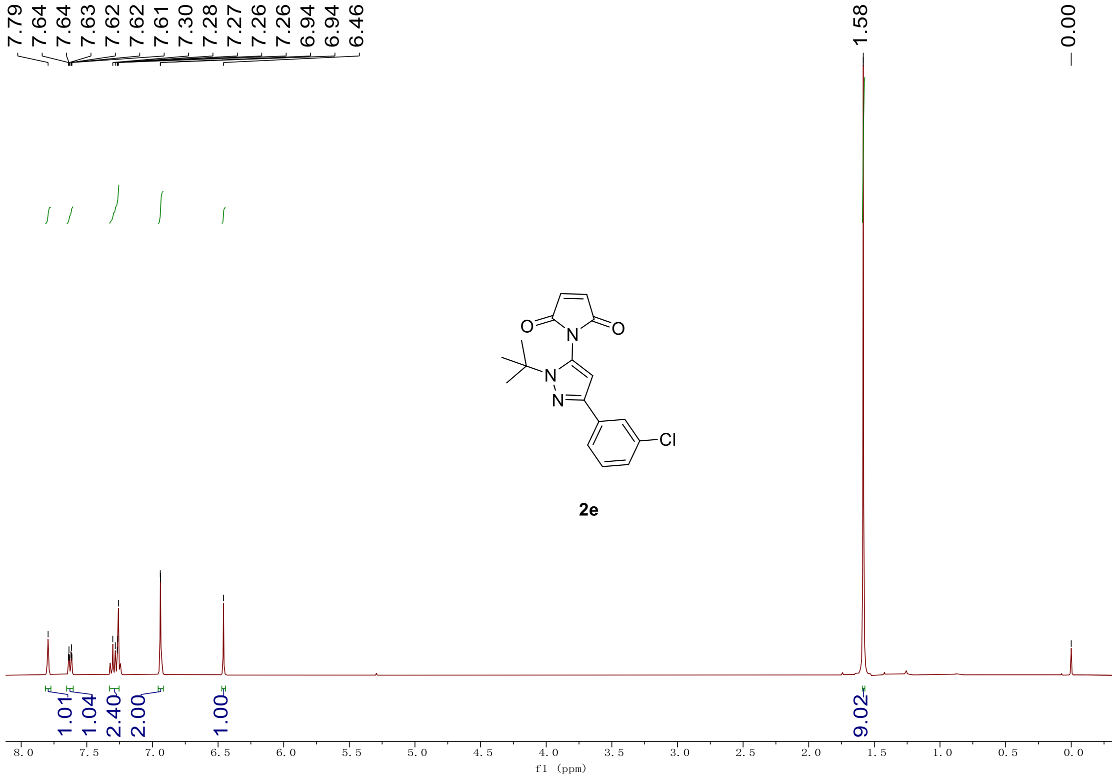

7.79
7.64
7.64
7.63
7.62
7.62
7.61
7.30
7.28
7.27
7.26
7.26
6.94
6.94
6.46
1.58
0.00
O
N
O
N
N
Cl
2e
1.01
1.04
2.40
2.00
1.00
9.02
f1 (ppm)

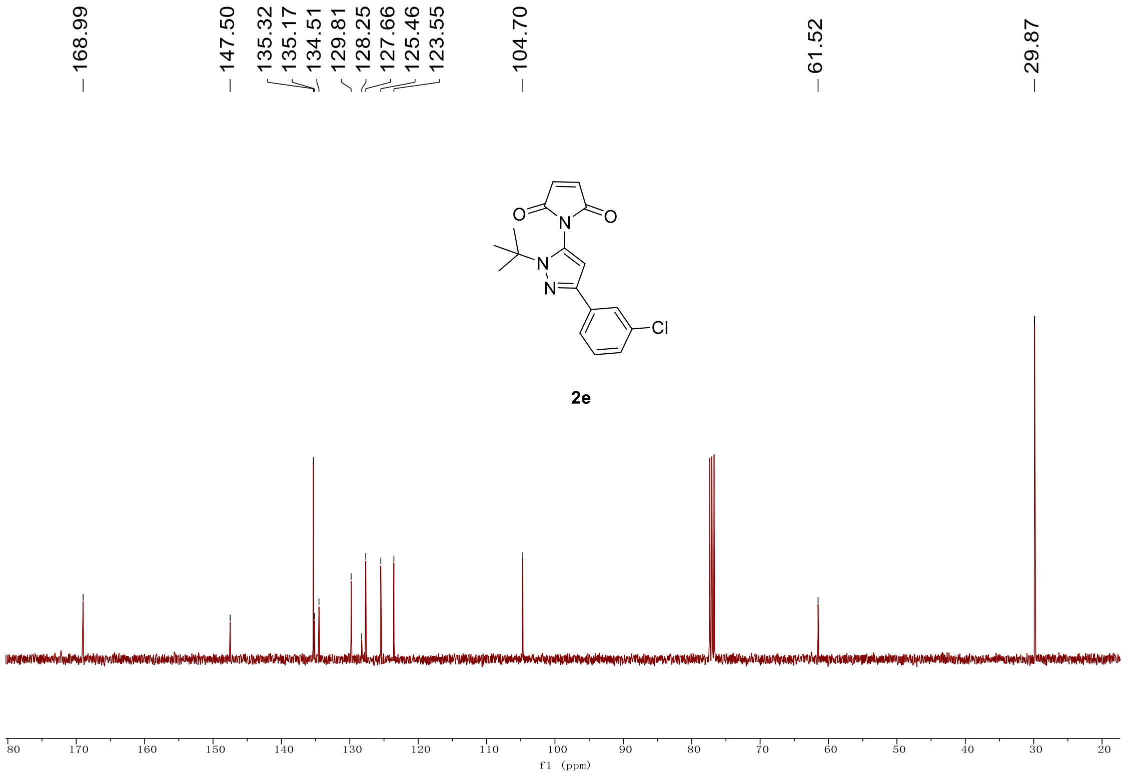

168.99
147.50
135.32
135.17
134.51
129.81
128.25
127.66
125.46
123.55
104.70
61.52
29.87
O
N
O
N
N
Cl
2e
f1 (ppm)

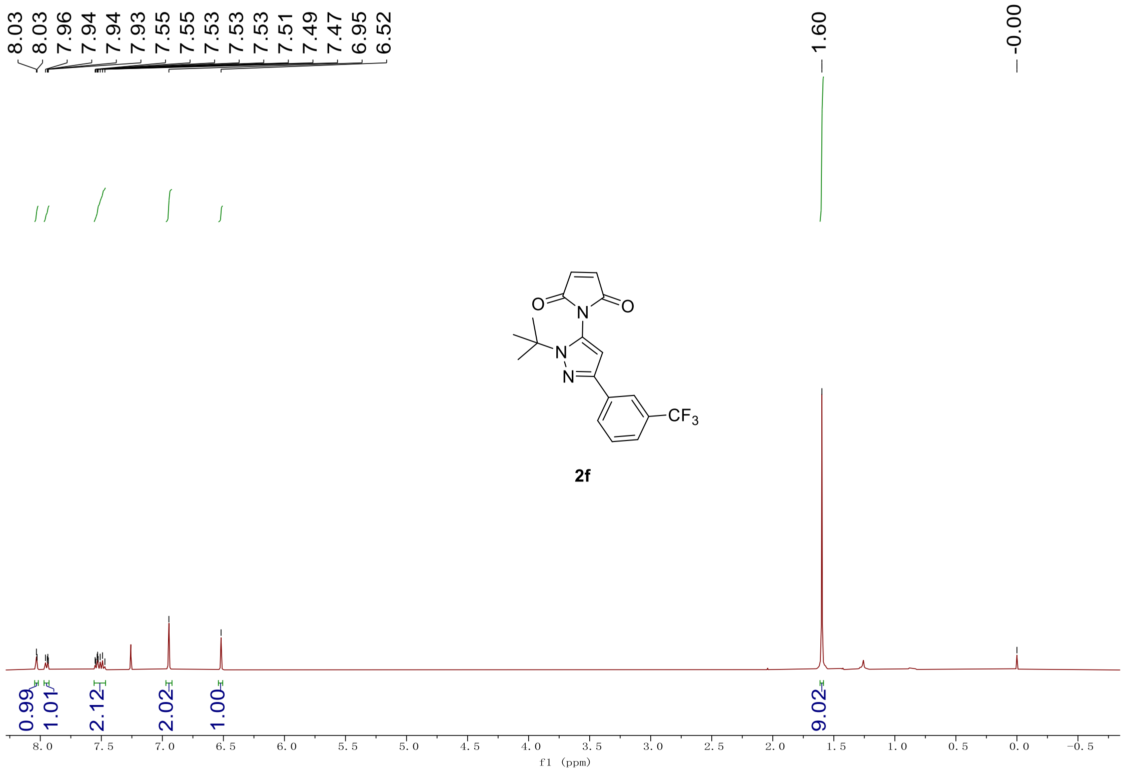
8.03
8.03
7.96
7.94
7.94
7.93
7.55
7.55
7.53
7.53
7.53
7.51
7.49
7.47
6.95
6.52
1.60
-0.00
O
N
O
N
N
CF3
0.99
1.01
2.12
2.02
1.00
9.02
f1 (ppm)

2f

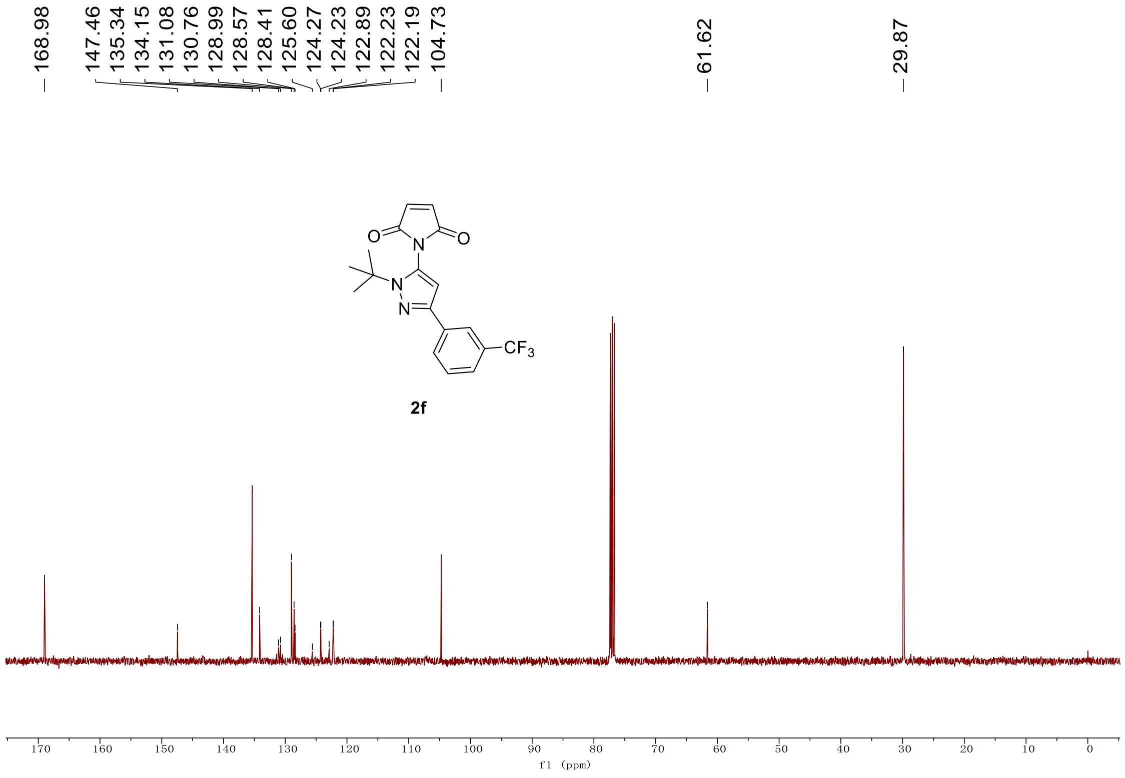
168.98
147.46
135.34
134.15
131.08
130.76
128.99
128.57
128.41
125.60
124.27
124.23
122.89
122.23
122.19
104.73
61.62
29.87
O
N
O
N
N
CF3
f1 (ppm)

2f

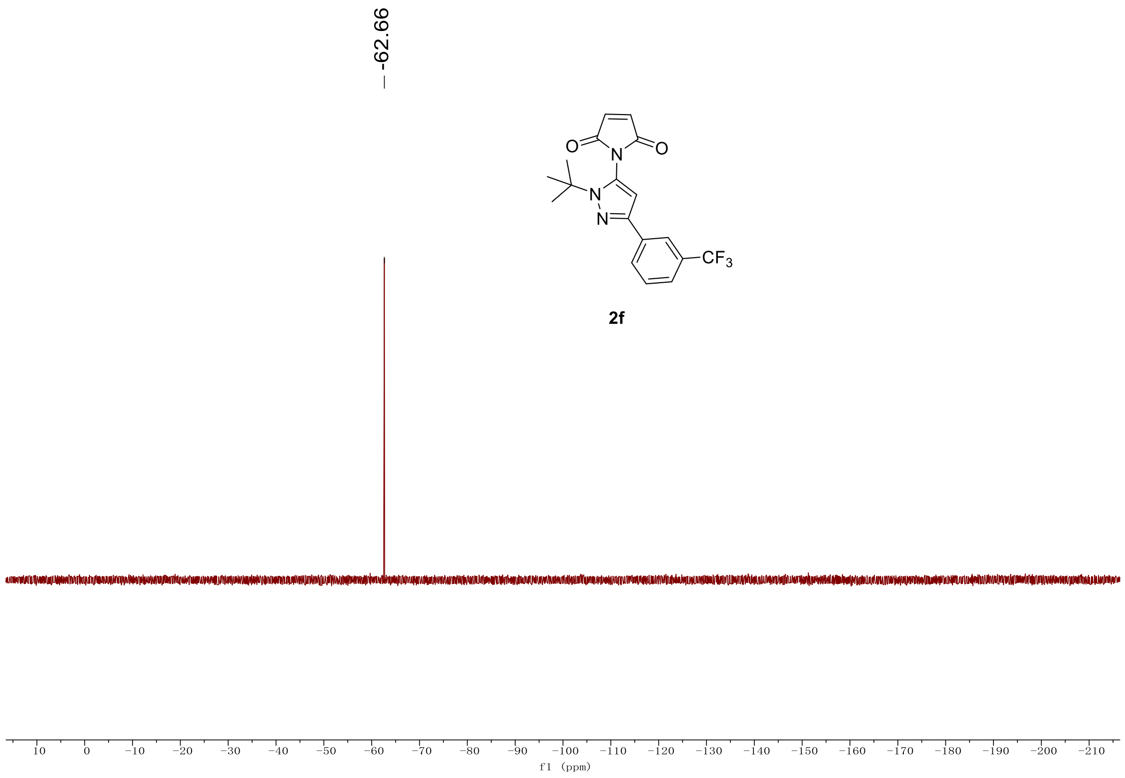
-62.66
O
N
O
N
N
CF3
2f
f1 (ppm)

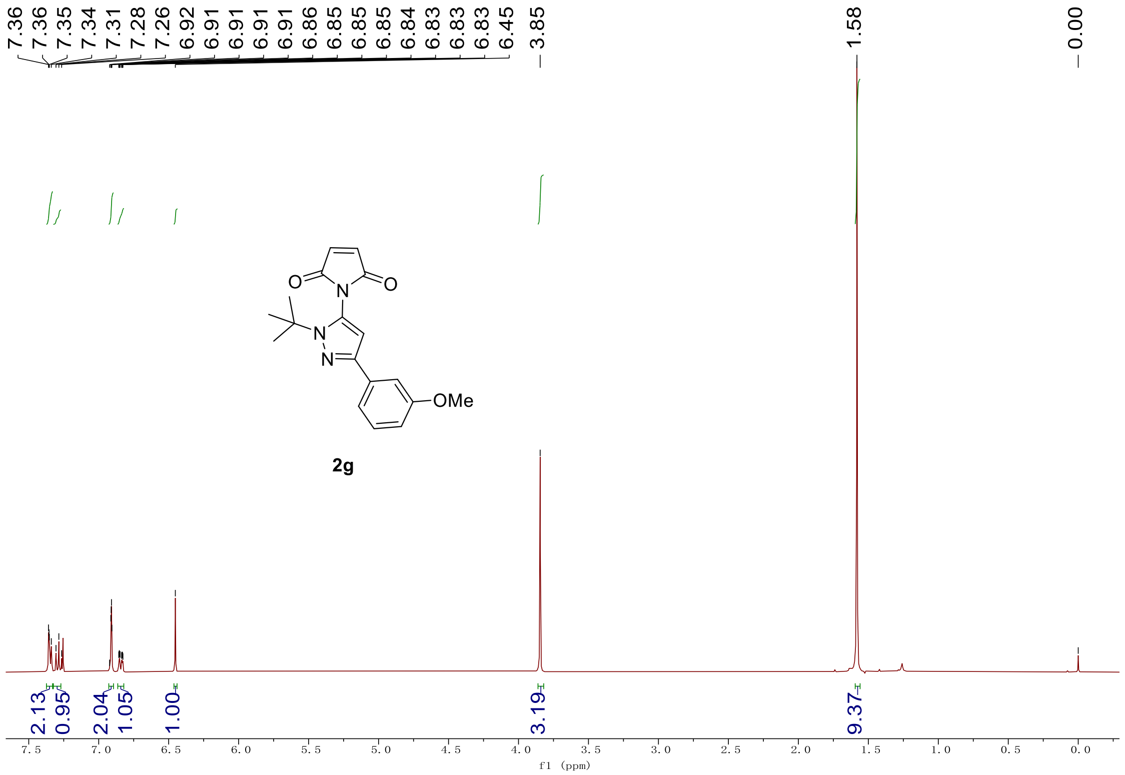
7.36
7.36
7.35
7.34
7.31
7.28
7.26
6.92
6.91
6.91
6.91
6.91
6.86
6.85
6.85
6.84
6.83
6.83
6.83
6.45
3.85
1.58
0.00
O
N
O
N
N
OMe
2g
2.13
0.95
2.04
1.05
1.00
3.19
9.37
f1 (ppm)

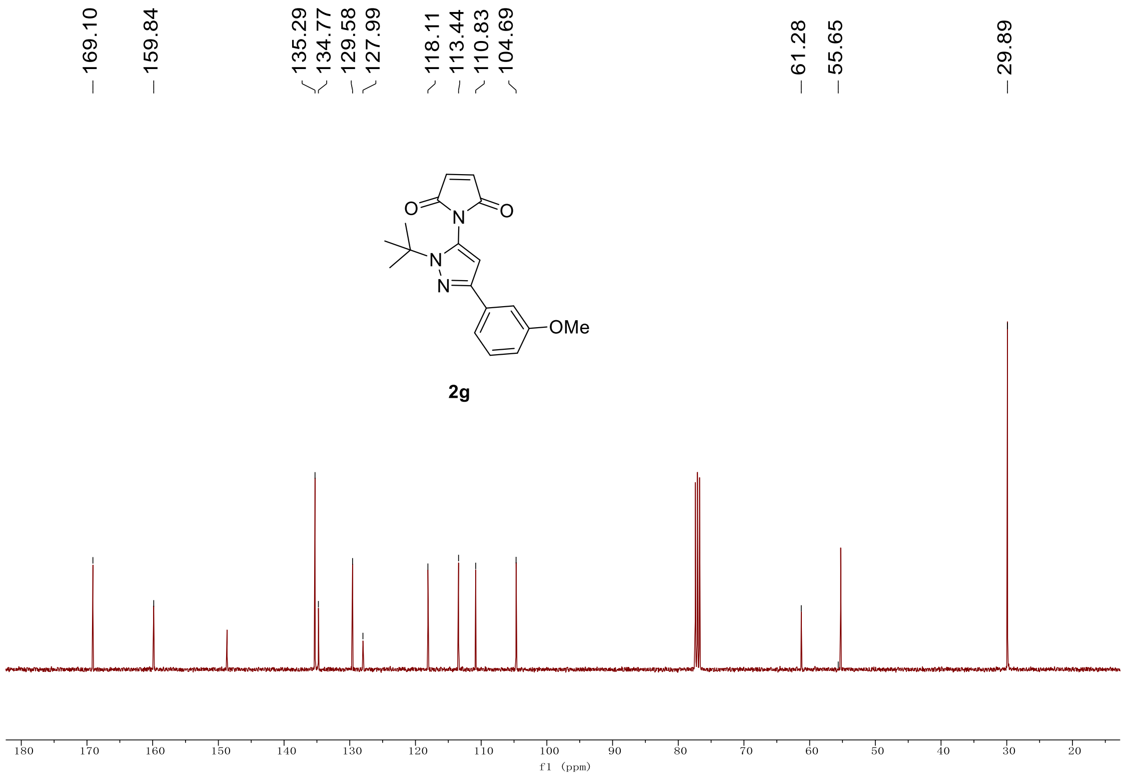
169.10
159.84
135.29
134.77
129.58
127.99
118.11
113.44
110.83
104.69
61.28
55.65
29.89
OMe
2g
f1 (ppm)

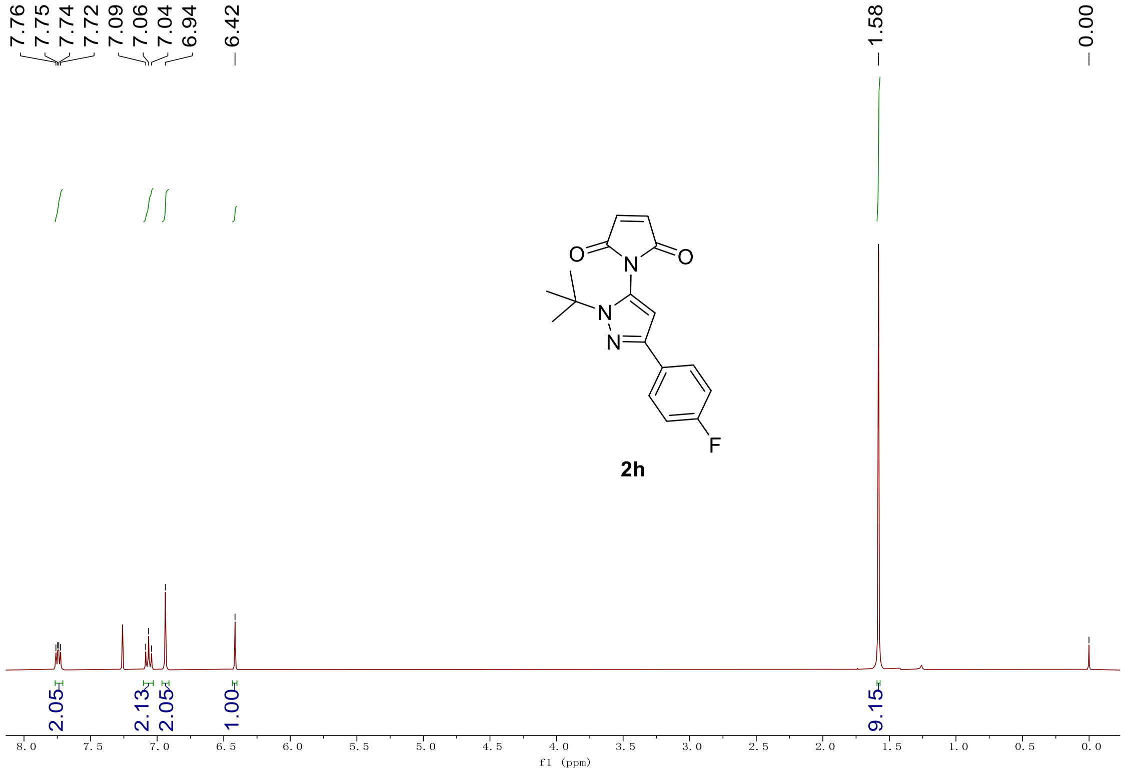
7.76
7.75
7.74
7.72
7.09
7.06
7.04
6.94
6.42
1.58
0.00
F
2h
2.05
2.13
2.05
1.00
9.15
f1 (ppm)

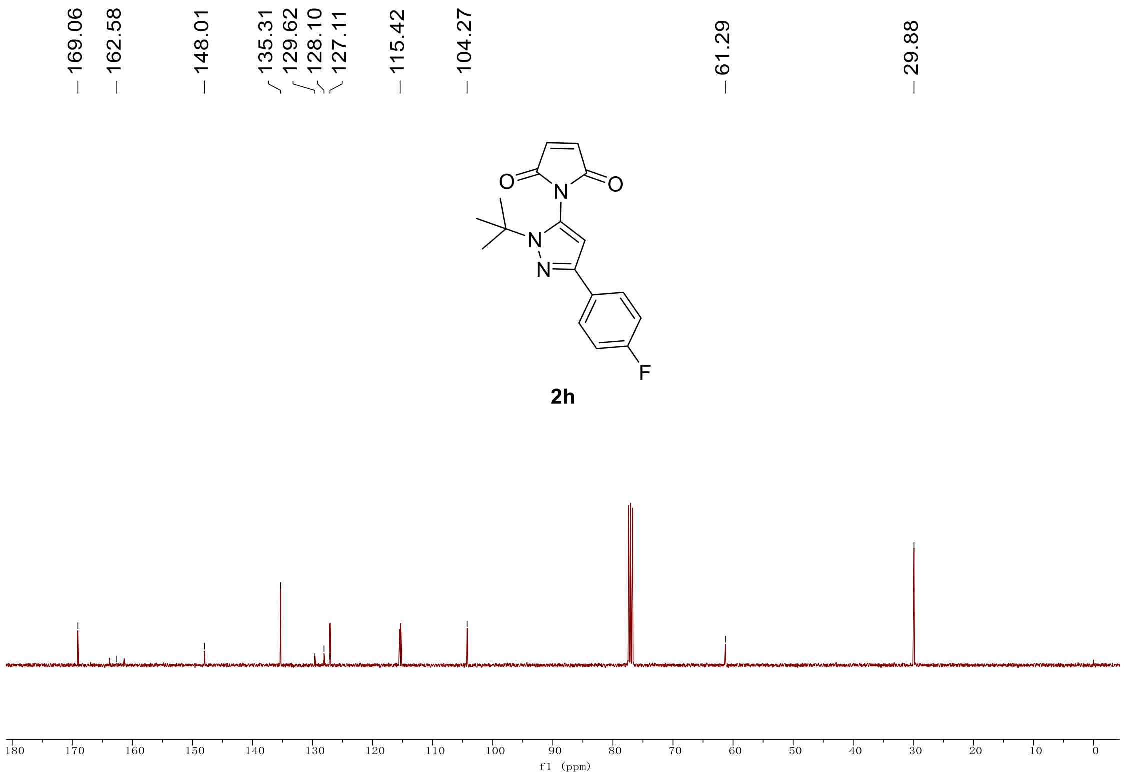
169.06
162.58
148.01
135.31
129.62
128.10
127.11
115.42
104.27
61.29
29.88
O
N
O
N
N
F
2h
180 170 160 150 140 130 120 110 100 90 80 70 60 50 40 30 20 10 0
f1 (ppm)

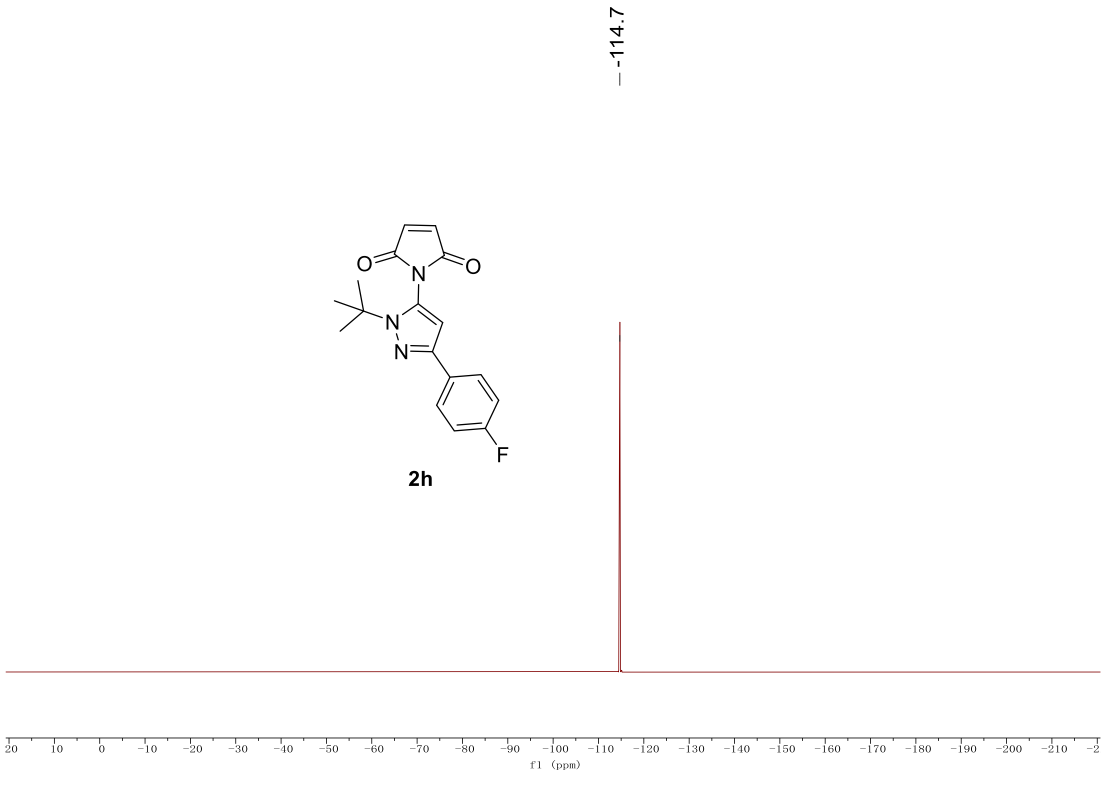
-114.7
O
N
O
N
N
F
2h
20 10 0 -10 -20 -30 -40 -50 -60 -70 -80 -90 -100 -110 -120 -130 -140 -150 -160 -170 -180 -190 -200 -210
f1 (ppm)

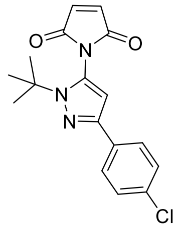

2i

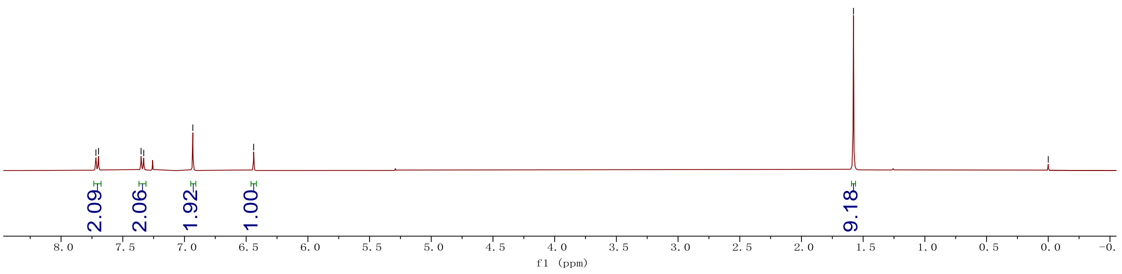
7.72
7.70
7.35
7.33
6.93
6.44
1.58
-0.00
2.09
2.06
1.92
1.00
9.18
f1 (ppm)

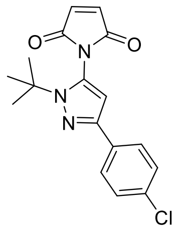

2i

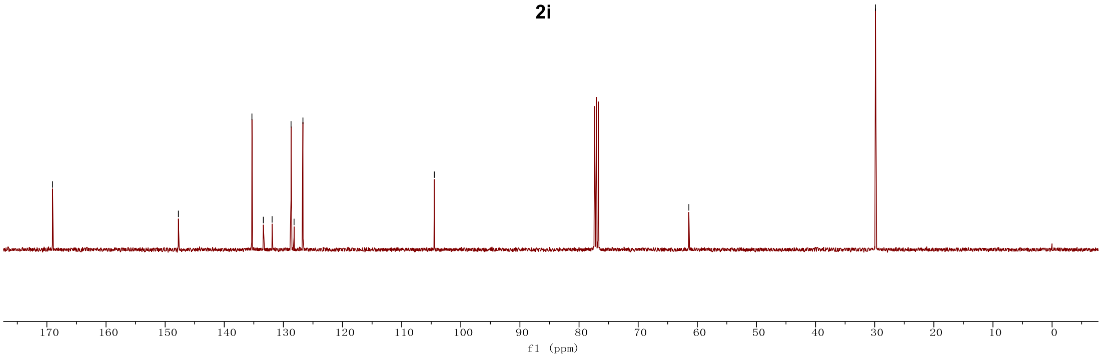
169.04
147.76
135.32
133.39
131.91
128.71
128.20
126.70
104.48
61.43
29.87
f1 (ppm)

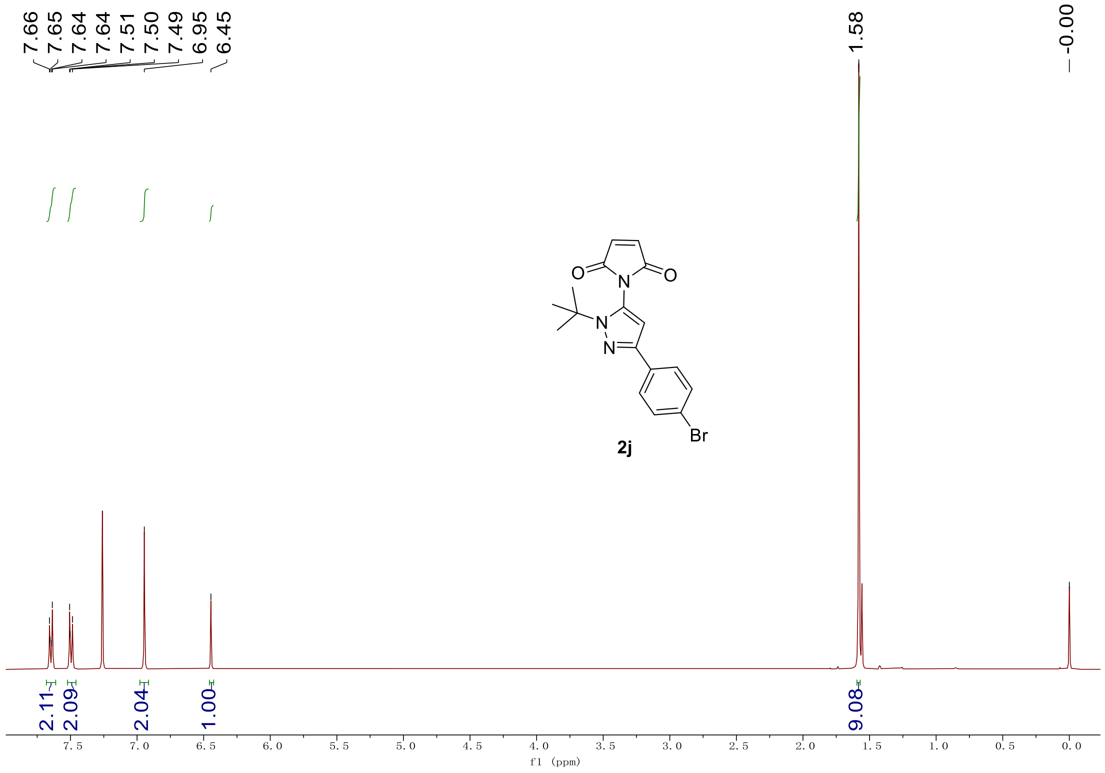
7.66
7.65
7.64
7.64
7.51
7.50
7.49
6.95
6.45
1.58
-0.00
2.11
2.09
2.04
1.00
9.08
O
N
O
N
N
Br
2j
f1 (ppm)

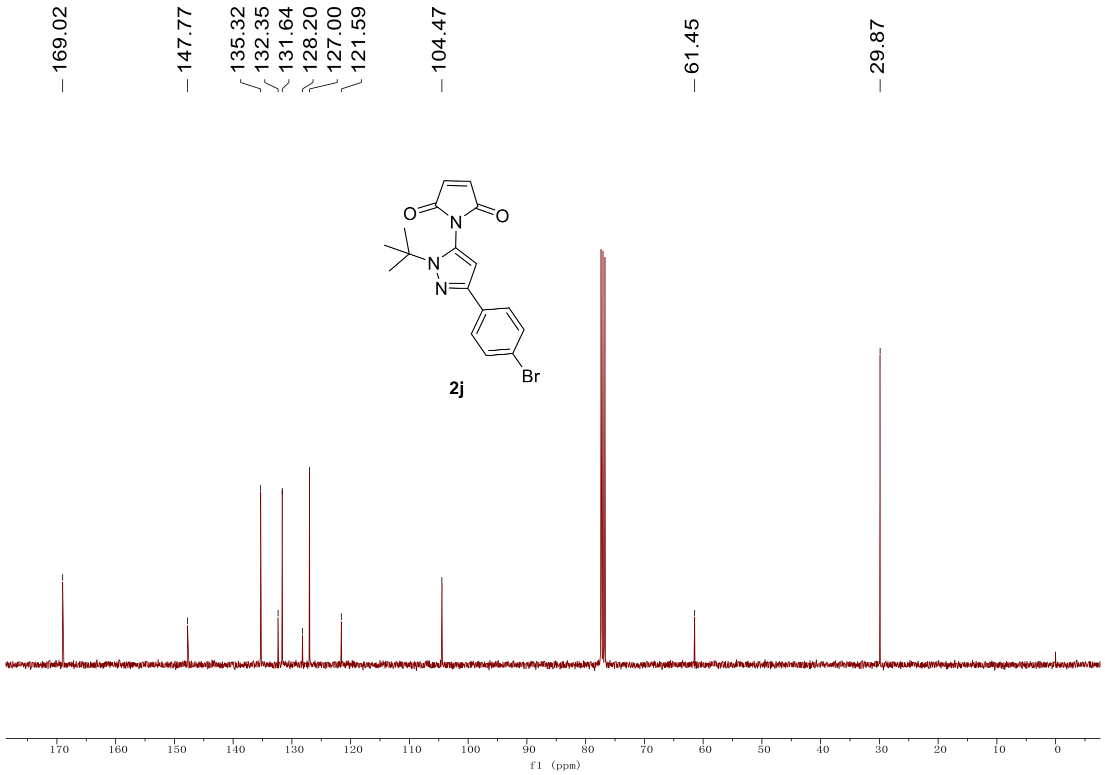
169.02
147.77
135.32
132.35
131.64
128.20
127.00
121.59
104.47
61.45
29.87
O
N
O
N
N
Br
2j
f1 (ppm)

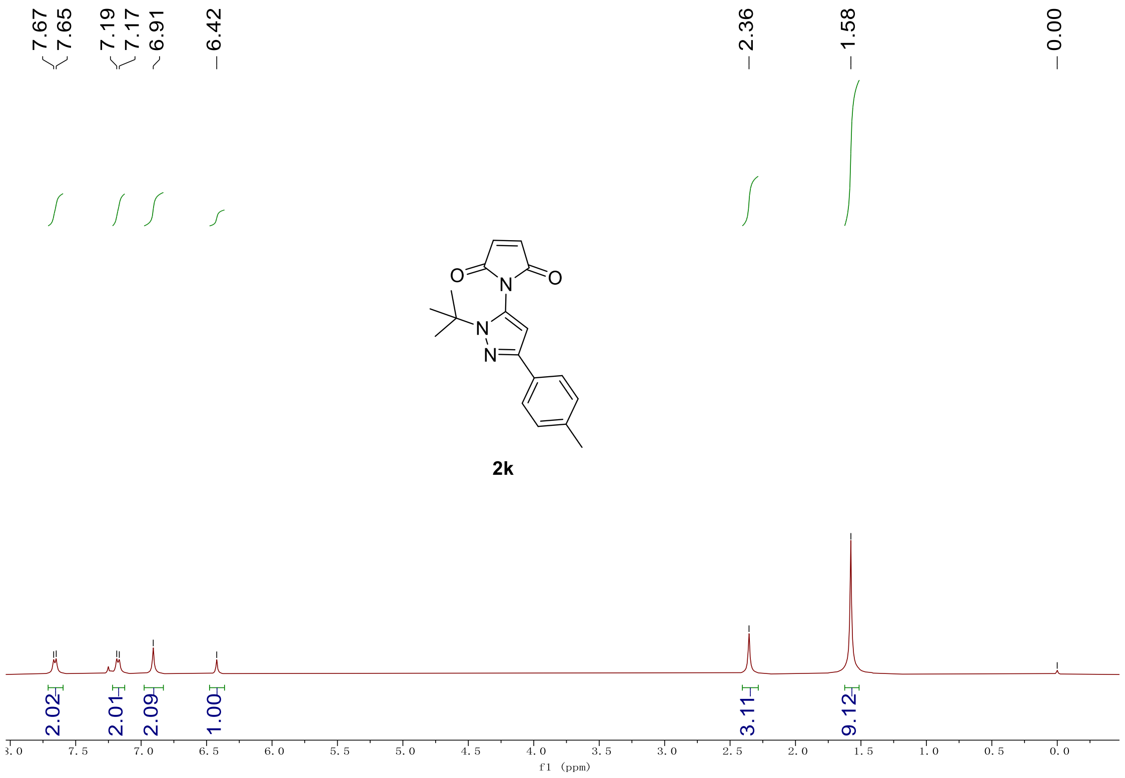
7.67
7.65
7.19
7.17
6.91
6.42
2.36
1.58
0.00
2.02
2.01
2.09
1.00
3.11
9.12
f1 (ppm)

2k

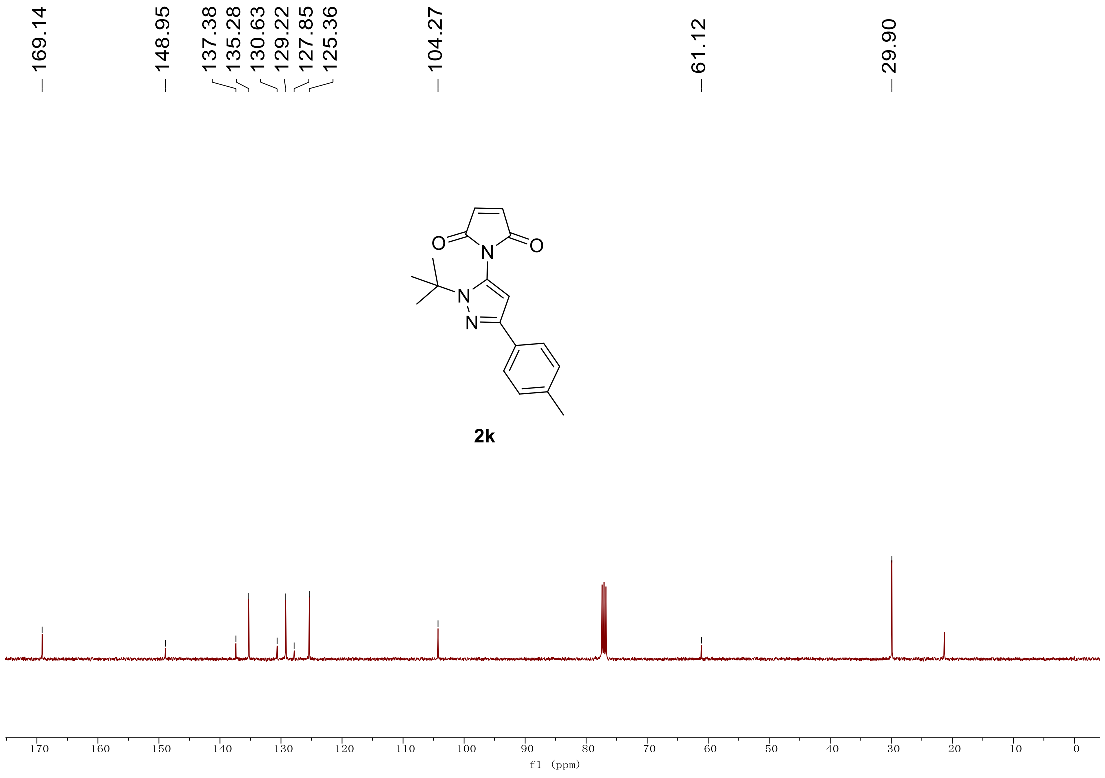
169.14
148.95
137.38
135.28
130.63
129.22
127.85
125.36
104.27
61.12
29.90
f1 (ppm)

2k

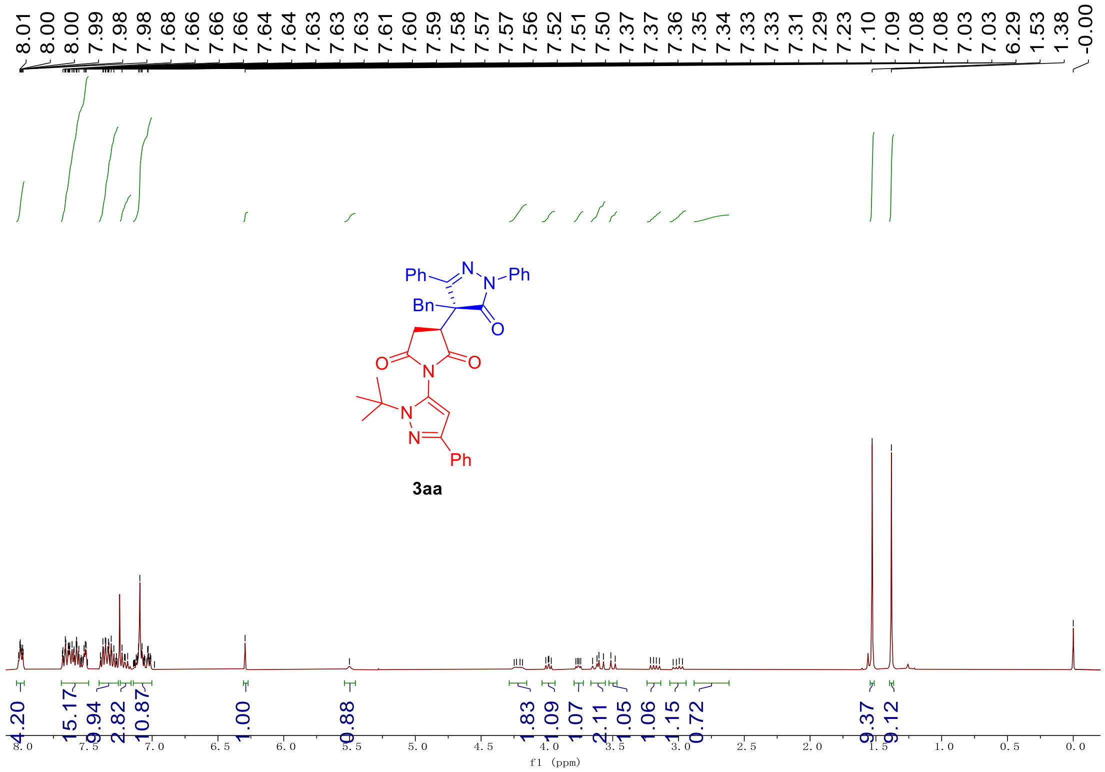
8.01 8.00 8.00 7.99 7.98 7.98 7.68 7.66 7.66 7.66 7.64 7.64 7.63 7.63 7.61 7.60 7.59 7.58 7.57 7.57 7.56 7.52 7.51 7.50 7.37 7.37 7.36 7.35 7.34 7.33 7.33 7.31 7.29 7.23 7.10 7.09 7.08 7.08 7.03 7.03 6.29 1.53 1.38 -0.00
Ph
N
N
Ph
Bn
O
O
O
N
N
N
Ph
3aa
4.20 15.17 9.94 2.82 10.87 1.00 0.88 1.83 1.09 1.07 2.11 1.05 1.06 1.15 0.72 9.37 9.12
8.0 7.5 7.0 6.5 6.0 5.5 5.0 4.5 4.0 3.5 3.0 2.5 2.0 1.5 1.0 0.5 0.0
f1 (ppm)

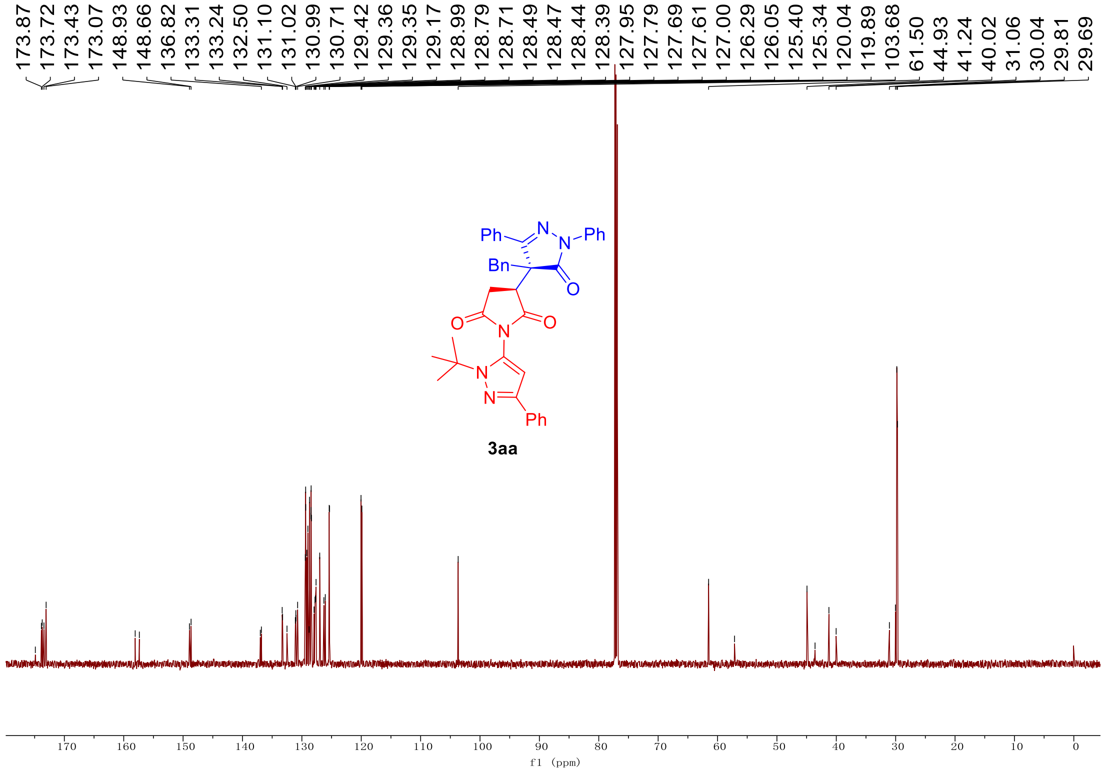
173.87 173.72 173.43 173.07 148.93 148.66 136.82 133.31 133.24 132.50 131.10 131.02 130.99 130.71 129.42 129.36 129.35 129.17 128.99 128.79 128.71 128.49 128.47 128.44 128.39 127.95 127.79 127.69 127.61 127.00 126.29 126.05 125.40 125.34 120.04 119.89 103.68 61.50 44.93 41.24 40.02 31.06 30.04 29.81 29.69
Ph
N
N
Ph
Bn
O
O
O
N
N
N
Ph
3aa
170 160 150 140 130 120 110 100 90 80 70 60 50 40 30 20 10 0
f1 (ppm)

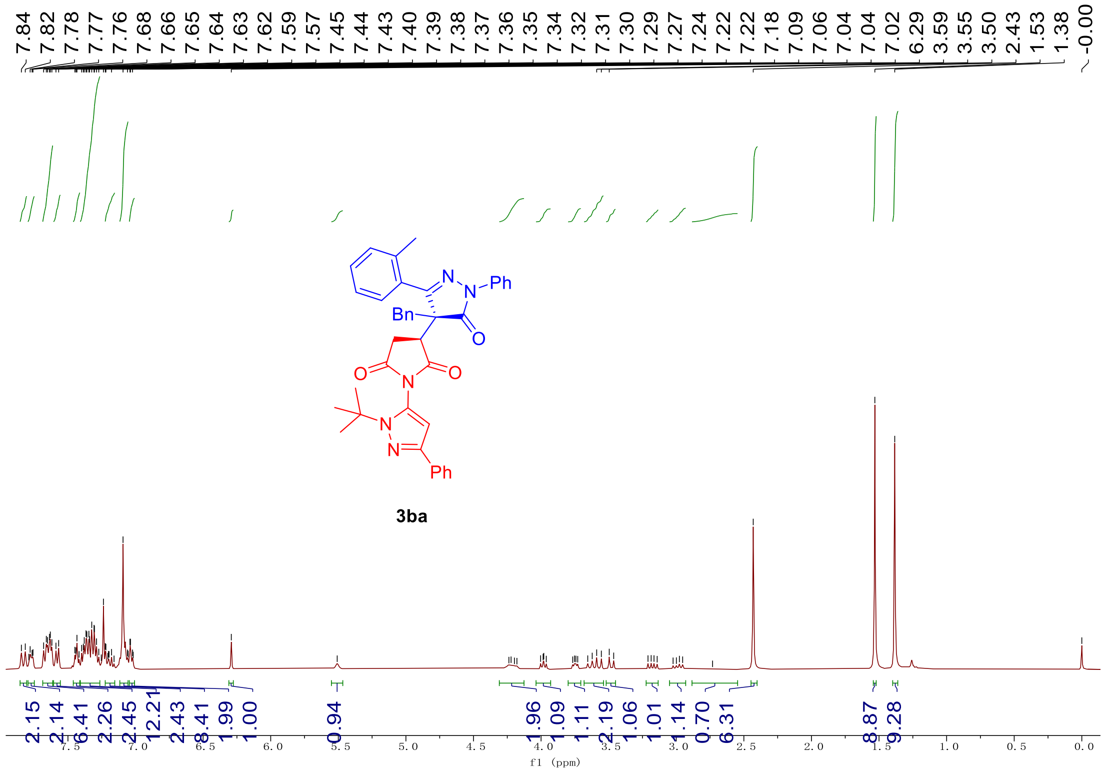

3ba

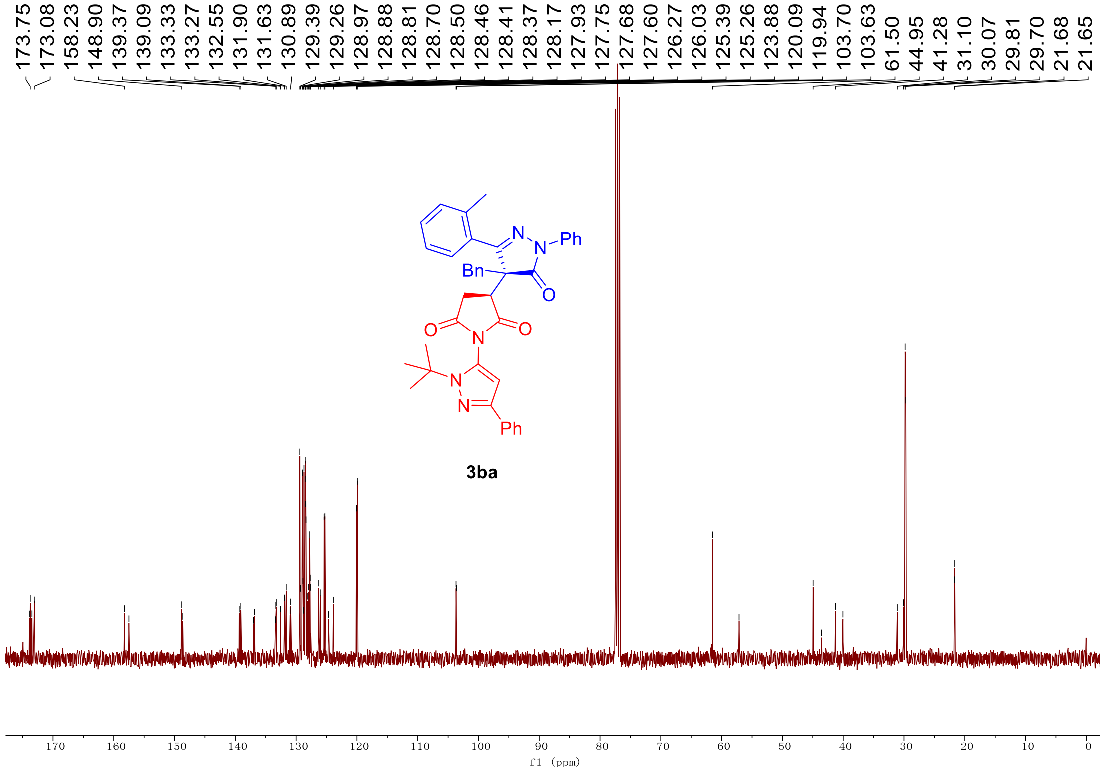

3ba

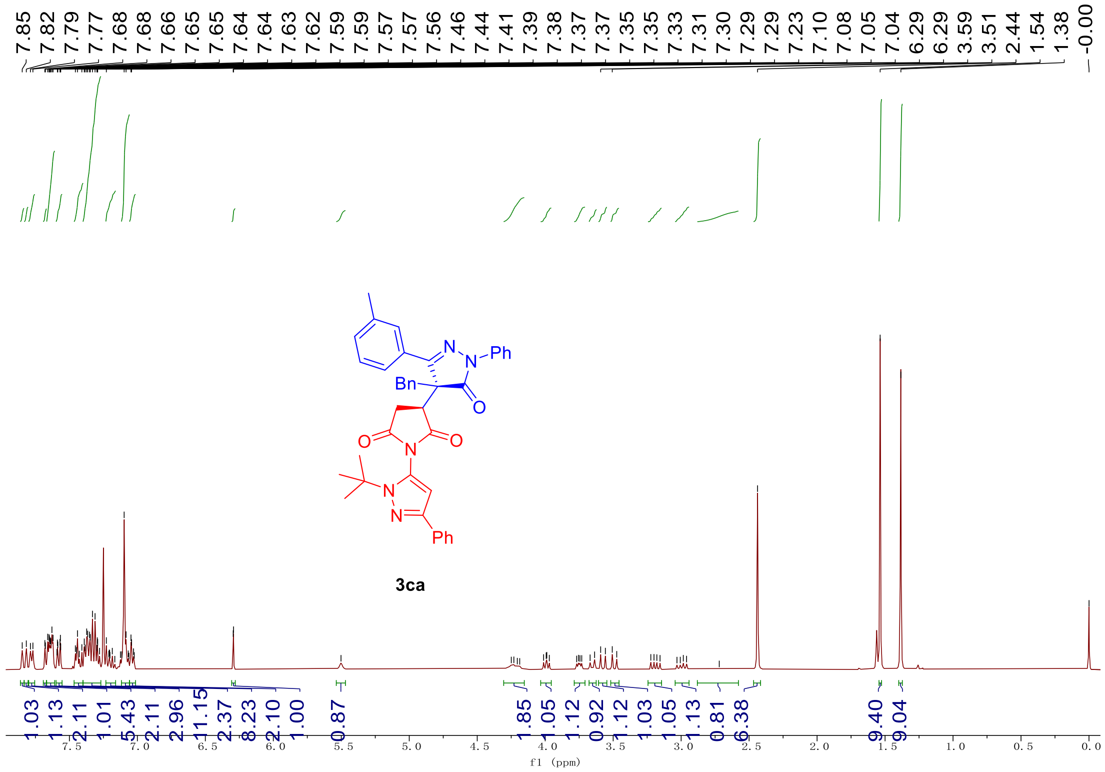
7.85
7.82
7.79
7.77
7.68
7.68
7.66
7.65
7.65
7.64
7.64
7.63
7.62
7.59
7.59
7.57
7.57
7.56
7.46
7.44
7.41
7.39
7.38
7.37
7.37
7.35
7.35
7.33
7.31
7.30
7.29
7.29
7.23
7.10
7.08
7.05
7.04
6.29
6.29
3.59
3.51
2.44
1.54
1.38
-0.00
N
N
Ph
Bn
O
O
N
O
N
N
Ph
1.03
1.13
2.11
1.01
5.43
2.11
2.96
11.15
2.37
8.23
2.10
1.00
0.87
1.85
1.05
1.12
0.92
1.12
1.03
1.05
1.13
0.81
6.38
9.40
9.04
f1 (ppm)

3ca

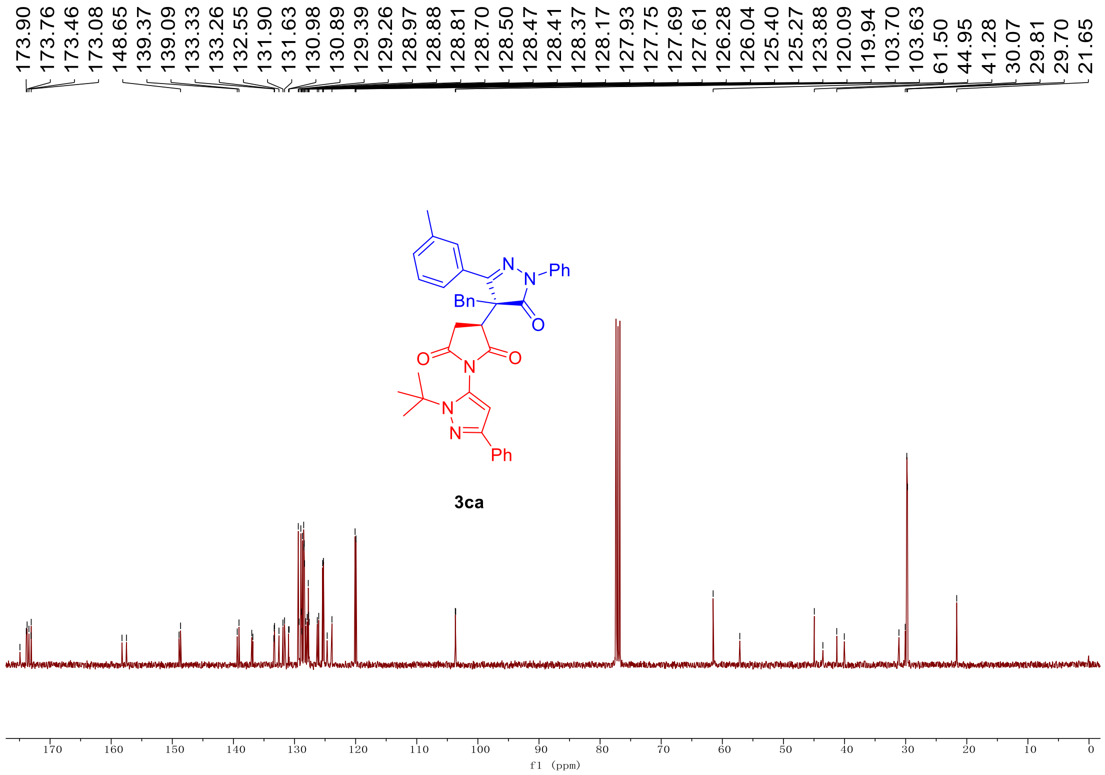
173.90
173.76
173.46
173.08
148.65
139.37
139.09
133.33
133.26
132.55
131.90
131.63
130.98
130.89
129.39
129.26
128.97
128.88
128.81
128.70
128.50
128.47
128.41
128.37
128.17
127.93
127.75
127.69
127.61
126.28
126.04
125.40
125.27
123.88
120.09
119.94
103.70
103.63
61.50
44.95
41.28
30.07
29.81
29.70
21.65
N
N
Ph
Bn
O
O
N
O
N
N
Ph
f1 (ppm)

3ca

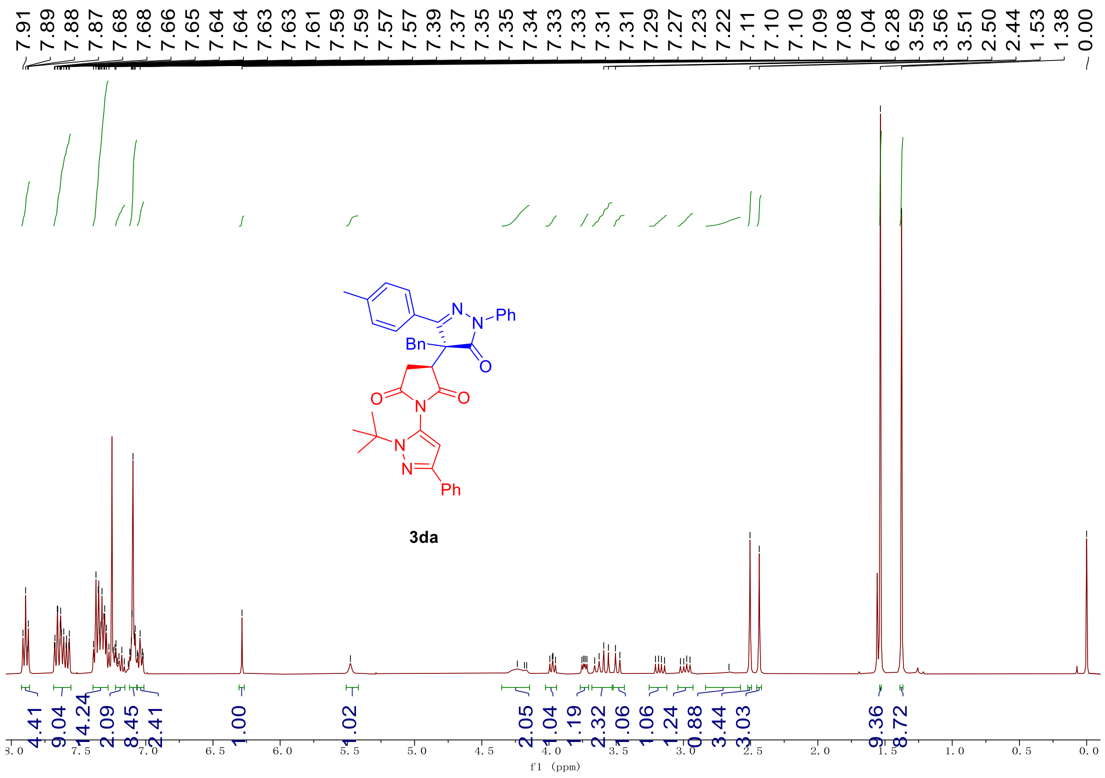
7.91 7.89 7.88 7.87 7.68 7.68 7.66 7.65 7.64 7.64 7.63 7.63 7.61 7.59 7.59 7.57 7.57 7.39 7.37 7.35 7.35 7.34 7.33 7.33 7.31 7.31 7.29 7.27 7.23 7.22 7.11 7.10 7.10 7.09 7.08 7.04 6.28 3.59 3.56 3.51 2.50 2.44 1.53 1.38 0.00
Bn
N
N
Ph
O
O
O
N
N
N
Ph
3da
4.41 9.04 14.24 2.09 8.45 2.41 1.00 1.02 2.05 1.04 1.19 2.32 1.06 1.06 1.24 0.88 3.44 3.03 9.36 8.72
f1 (ppm)

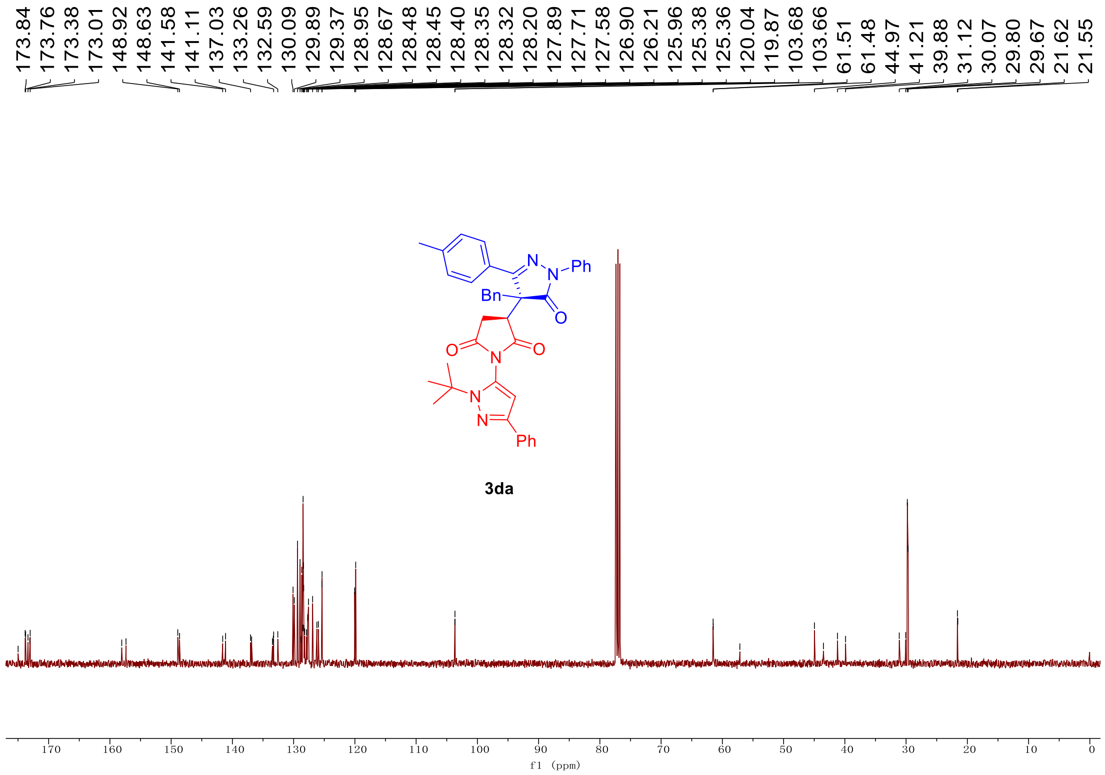
173.84 173.76 173.38 173.01 148.92 148.63 141.58 141.11 137.03 133.26 132.59 130.09 129.89 129.37 128.95 128.67 128.48 128.45 128.40 128.35 128.32 128.20 127.89 127.71 127.58 126.90 126.21 125.96 125.38 125.36 120.04 119.87 103.68 103.66 61.51 61.48 44.97 41.21 39.88 31.12 30.07 29.80 29.67 21.62 21.55
Bn
N
N
Ph
O
O
O
N
N
N
Ph
3da
f1 (ppm)

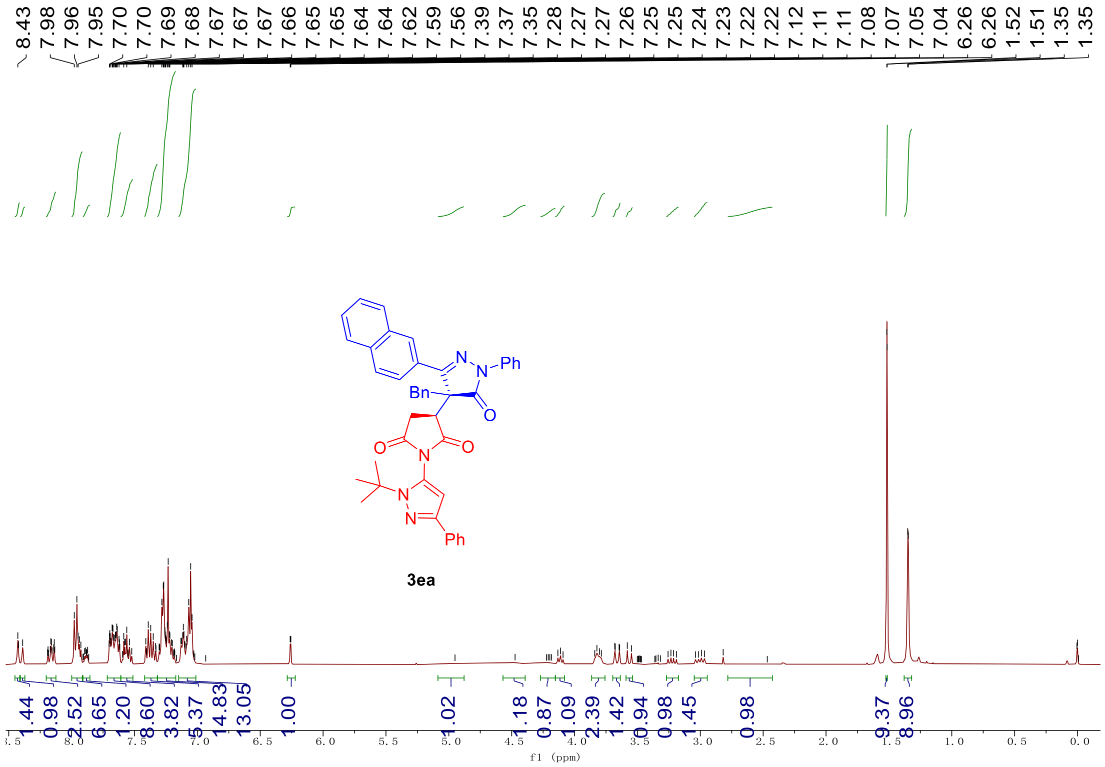

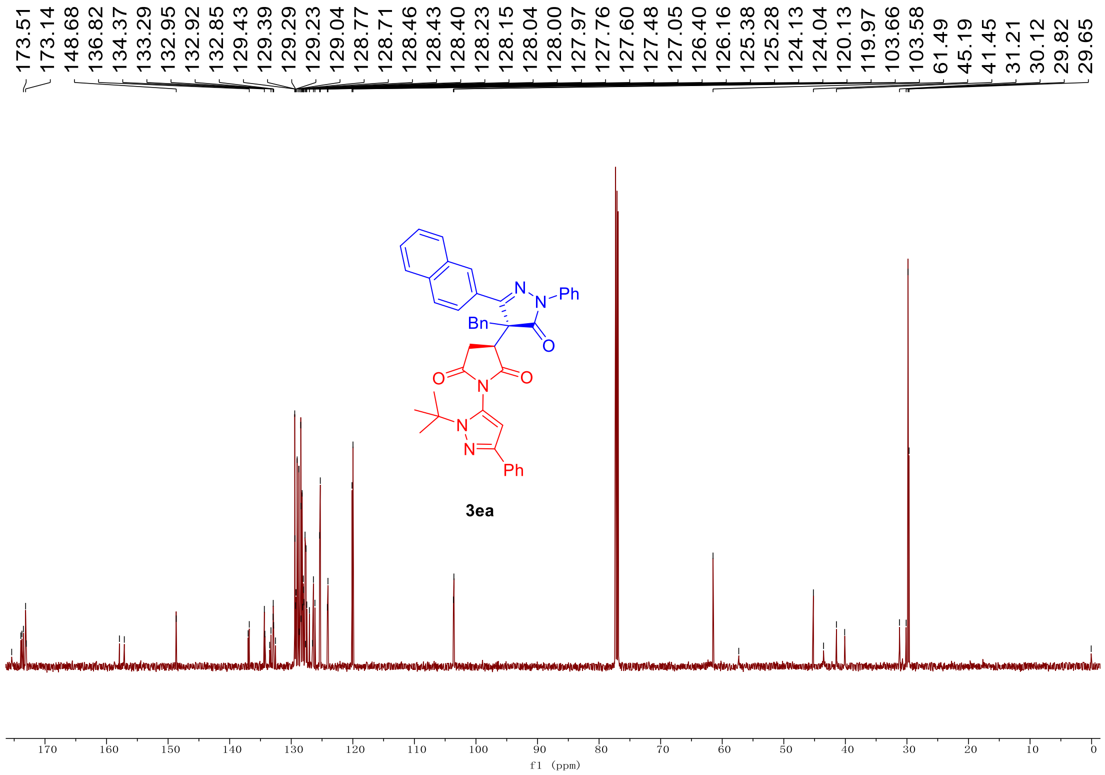

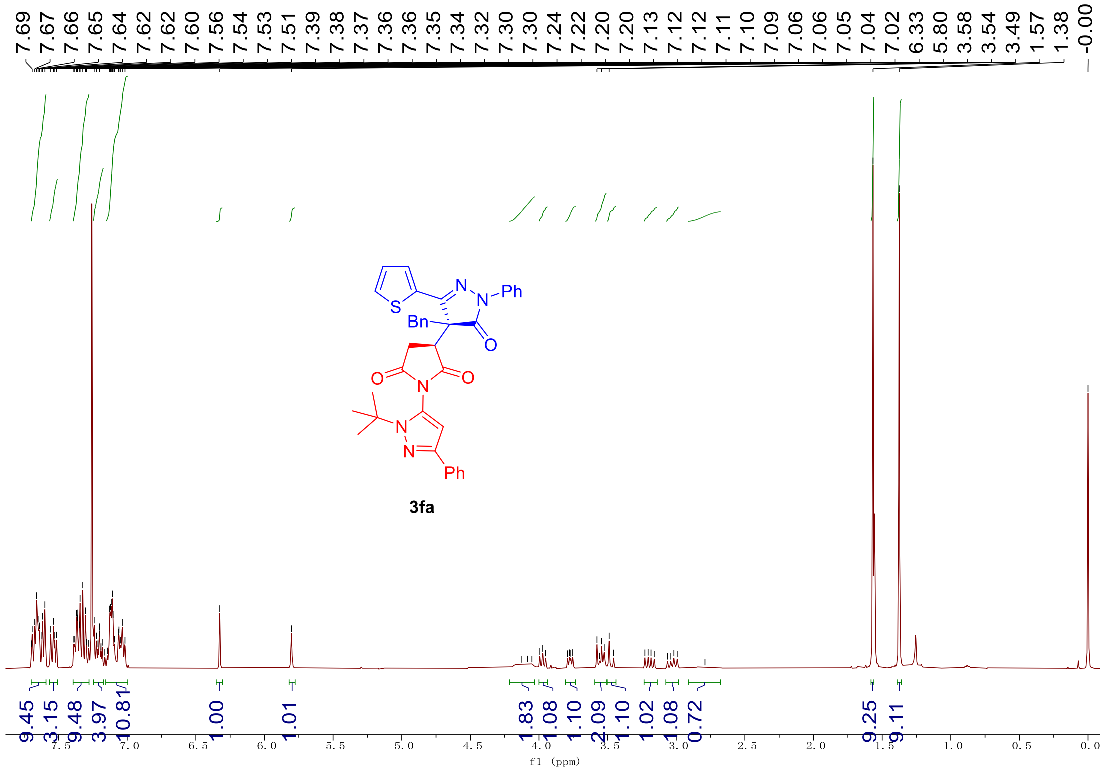
7.69
7.67
7.66
7.65
7.64
7.62
7.60
7.56
7.54
7.53
7.51
7.39
7.38
7.37
7.36
7.36
7.35
7.34
7.32
7.30
7.30
7.24
7.22
7.20
7.20
7.13
7.12
7.12
7.11
7.10
7.09
7.06
7.06
7.05
7.04
7.02
6.33
5.80
3.58
3.54
3.49
1.57
1.38
-0.00
S
N
N
Ph
Bn
O
O
O
N
N
N
Ph
3fa
9.45
3.15
9.48
3.97
10.81
1.00
1.01
1.83
1.08
1.10
2.09
1.10
1.02
1.08
0.72
9.25
9.11
f1 (ppm)

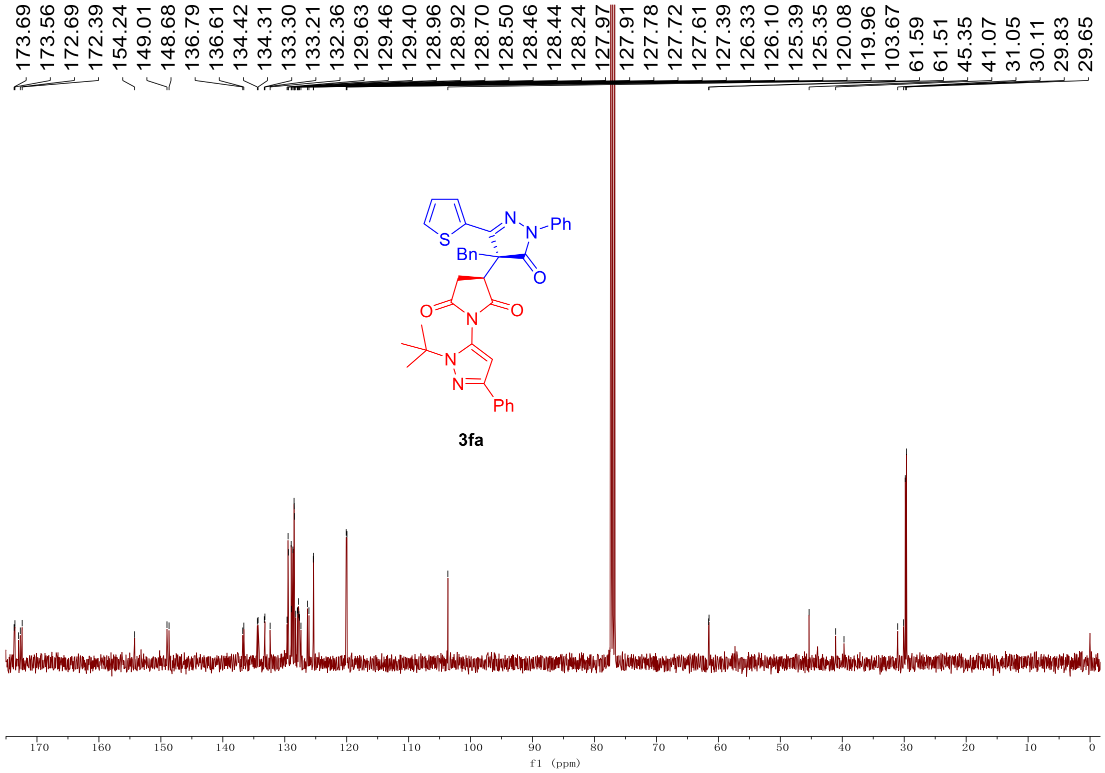
173.69
173.56
172.69
172.39
154.24
149.01
148.68
136.79
136.61
134.42
134.31
133.30
133.21
132.36
129.63
129.46
129.40
128.96
128.92
128.70
128.50
128.46
128.44
128.24
127.97
127.91
127.78
127.72
127.61
127.39
126.33
126.10
125.39
125.35
120.08
119.96
103.67
61.59
61.51
45.35
41.07
31.05
30.11
29.83
29.65
S
N
N
Ph
Bn
O
O
O
N
N
N
Ph
3fa
f1 (ppm)

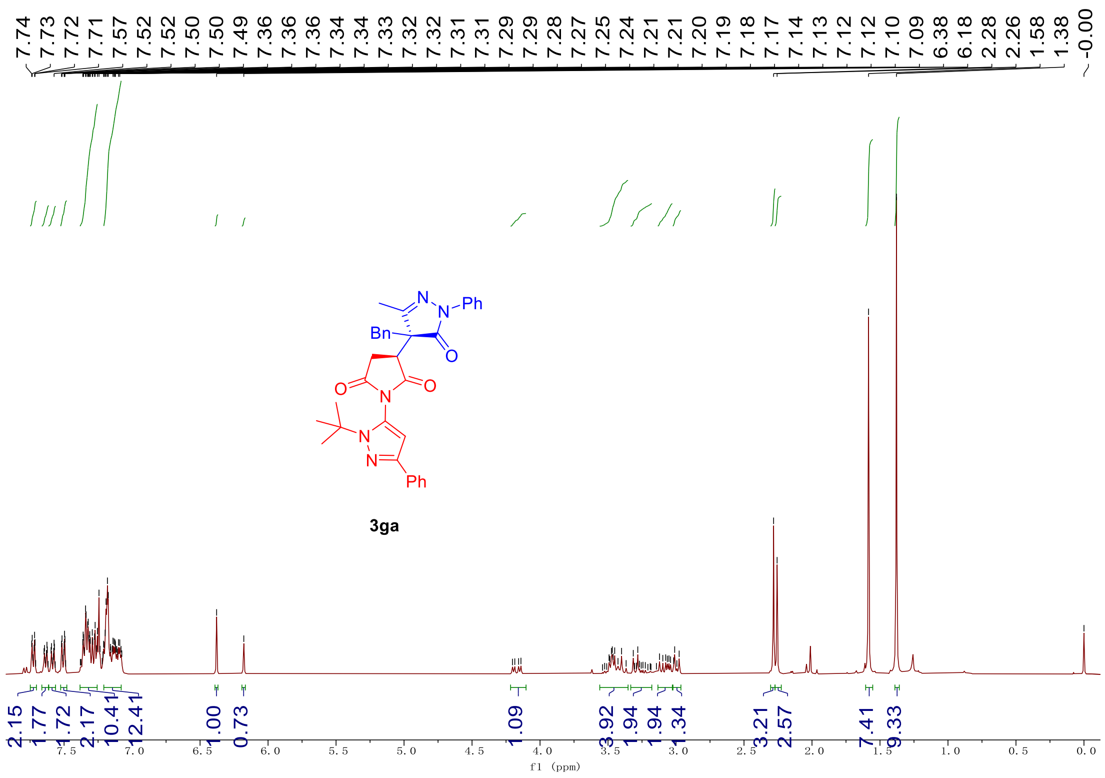

3ga

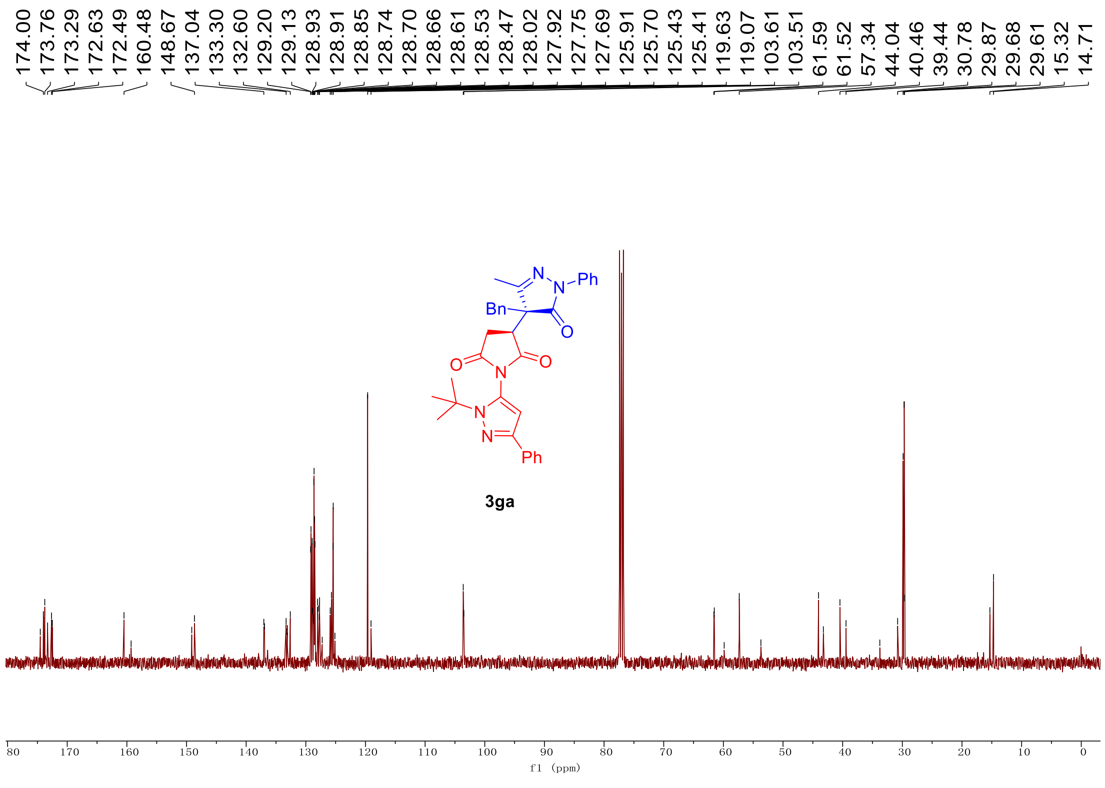

3ga

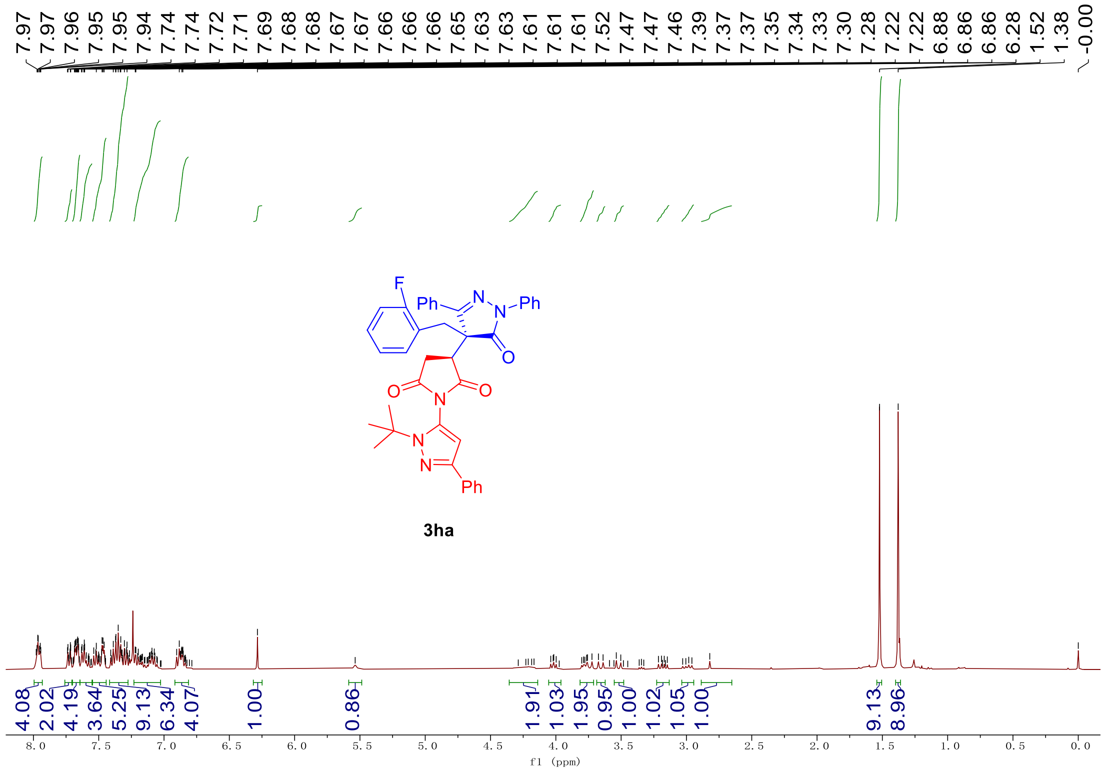

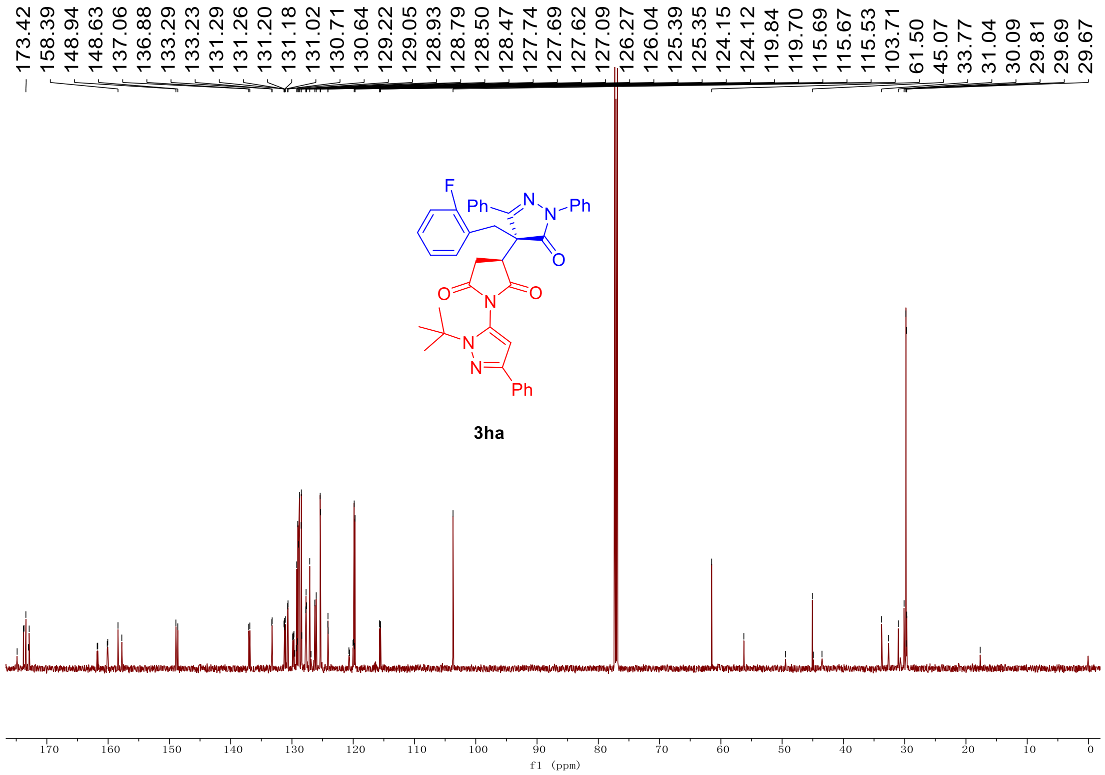

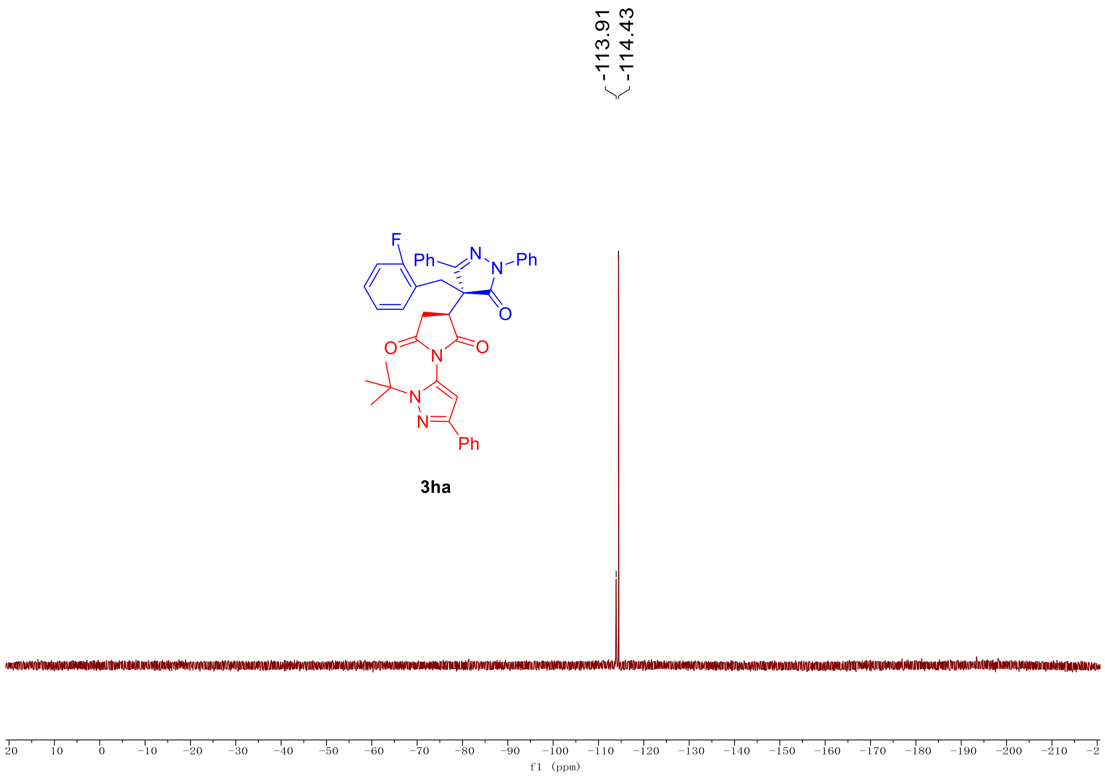

-113.91
-114.43
F
Ph
N
N
Ph
O
O
N
O
N
N
Ph
3ha
20 10 0 −10 −20 −30 −40 −50 −60 −70 −80 −90 −100 −110 −120 −130 −140 −150 −160 −170 −180 −190 −200 −210 −2
f1 (ppm)

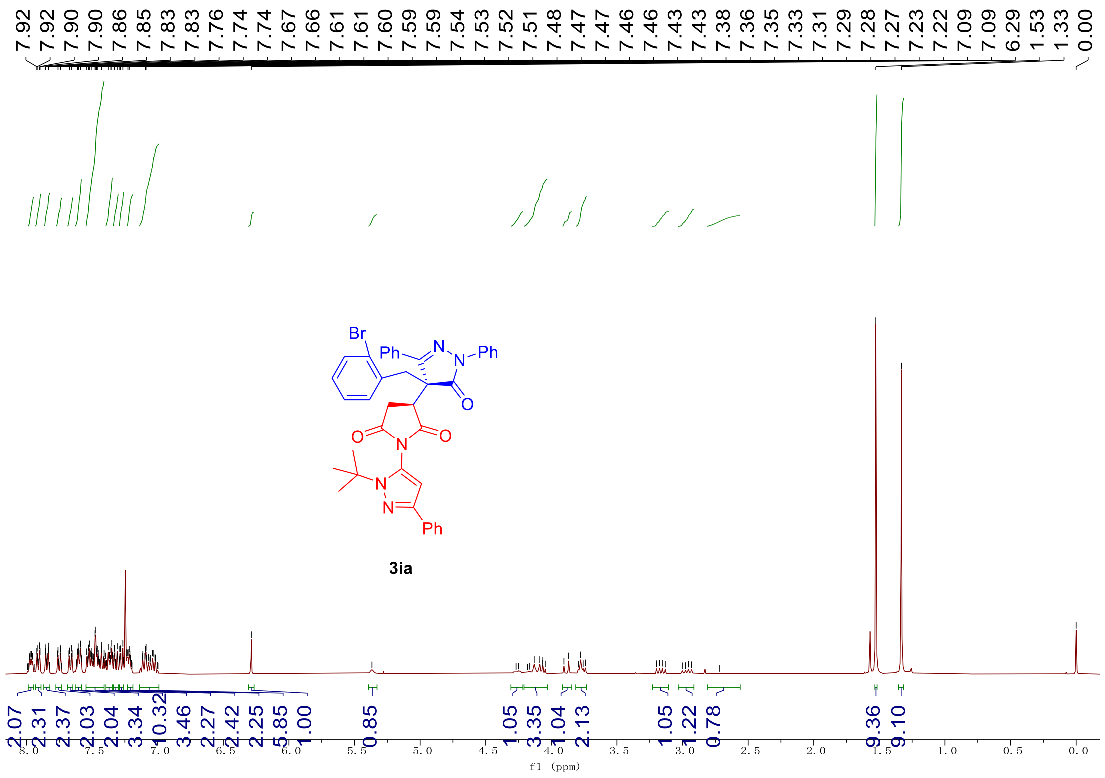

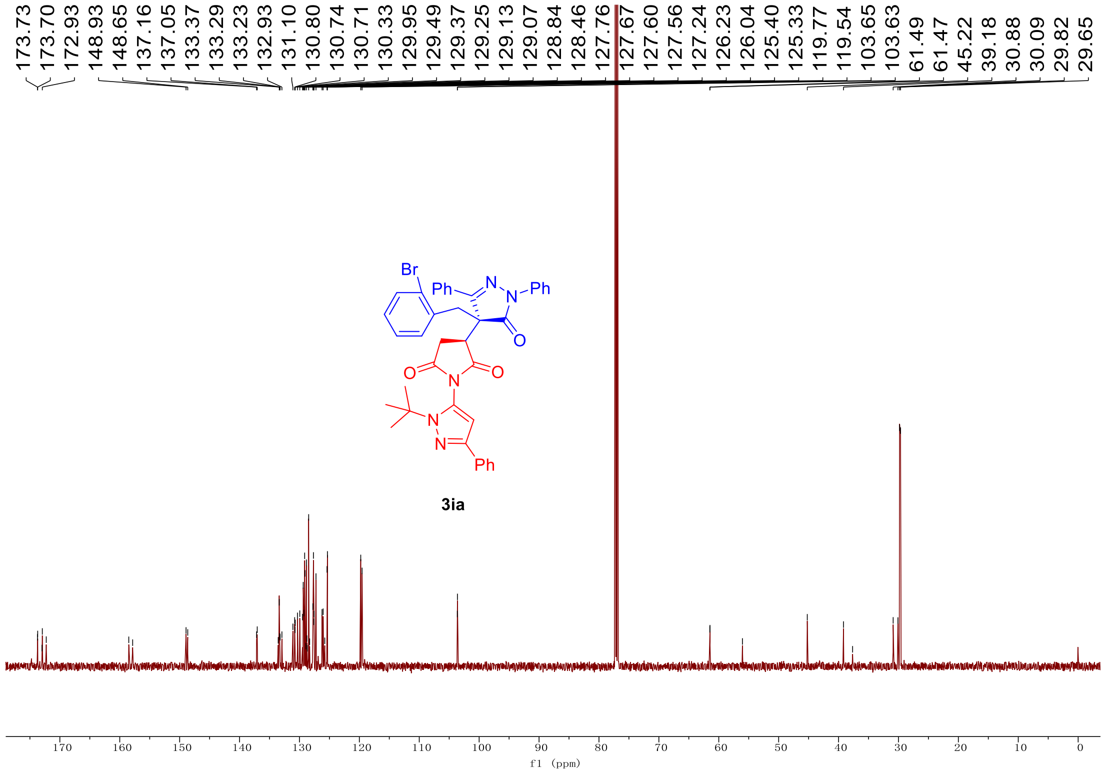

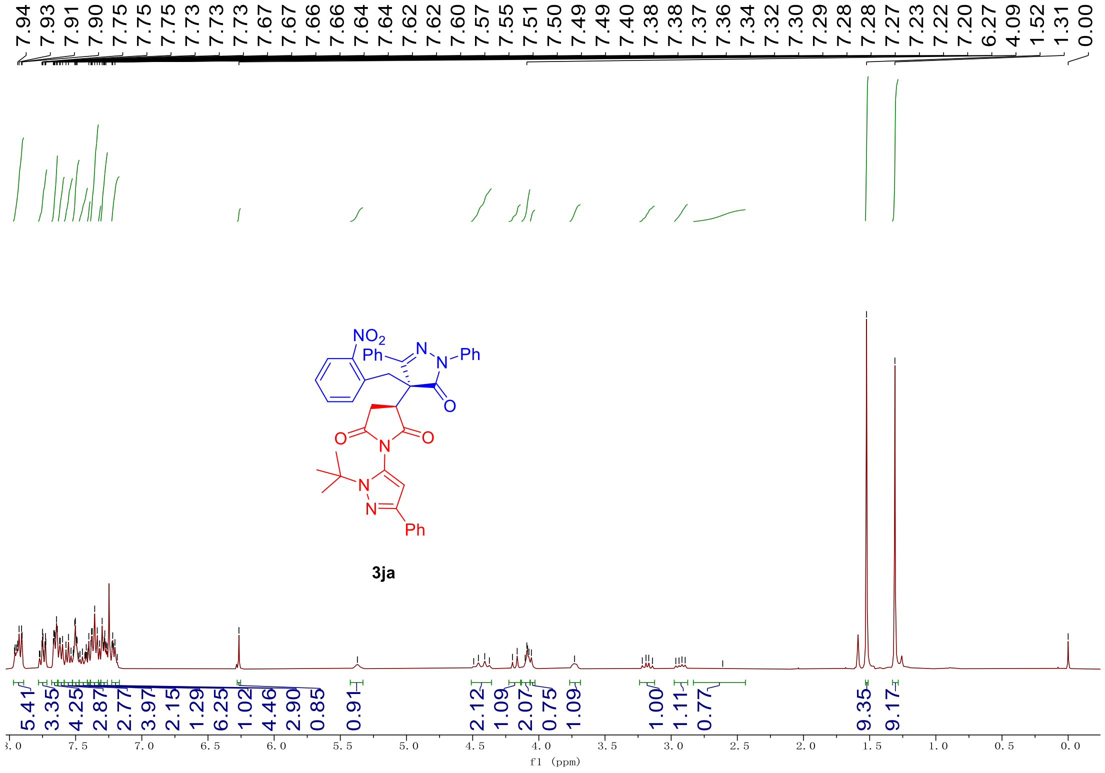
7.94 7.93 7.91 7.90 7.75 7.75 7.73 7.73 7.73 7.67 7.67 7.66 7.66 7.64 7.64 7.62 7.62 7.60 7.57 7.55 7.51 7.50 7.49 7.49 7.40 7.38 7.38 7.37 7.36 7.34 7.32 7.30 7.29 7.28 7.28 7.27 7.23 7.22 7.20 6.27 4.09 1.52 1.31 0.00
NO2
Ph
N
N
Ph
O
O
N
O
N
N
Ph
3ja
5.41 3.35 4.25 2.87 2.77 3.97 2.15 1.29 6.25 1.02 4.46 2.90 0.85 0.91 2.12 1.09 2.07 0.75 1.09 1.00 1.11 0.77 9.35 9.17
f1 (ppm)

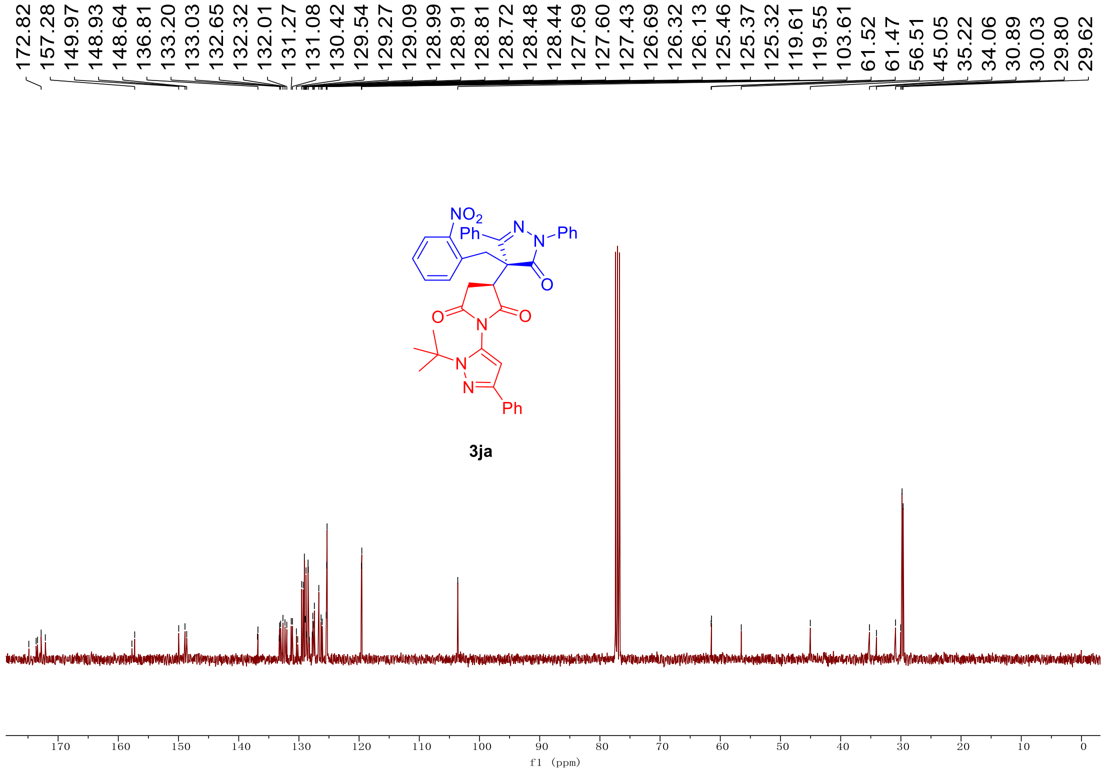
172.82 157.28 149.97 148.93 148.64 136.81 133.20 133.03 132.65 132.32 132.01 131.27 131.08 130.42 129.54 129.27 129.09 128.99 128.91 128.81 128.72 128.48 128.44 127.69 127.60 127.43 126.69 126.32 126.13 125.46 125.37 125.32 119.61 119.55 103.61 61.52 61.47 56.51 45.05 35.22 34.06 30.89 30.03 29.80 29.62
NO2
Ph
N
N
Ph
O
O
N
O
N
N
Ph
3ja
f1 (ppm)

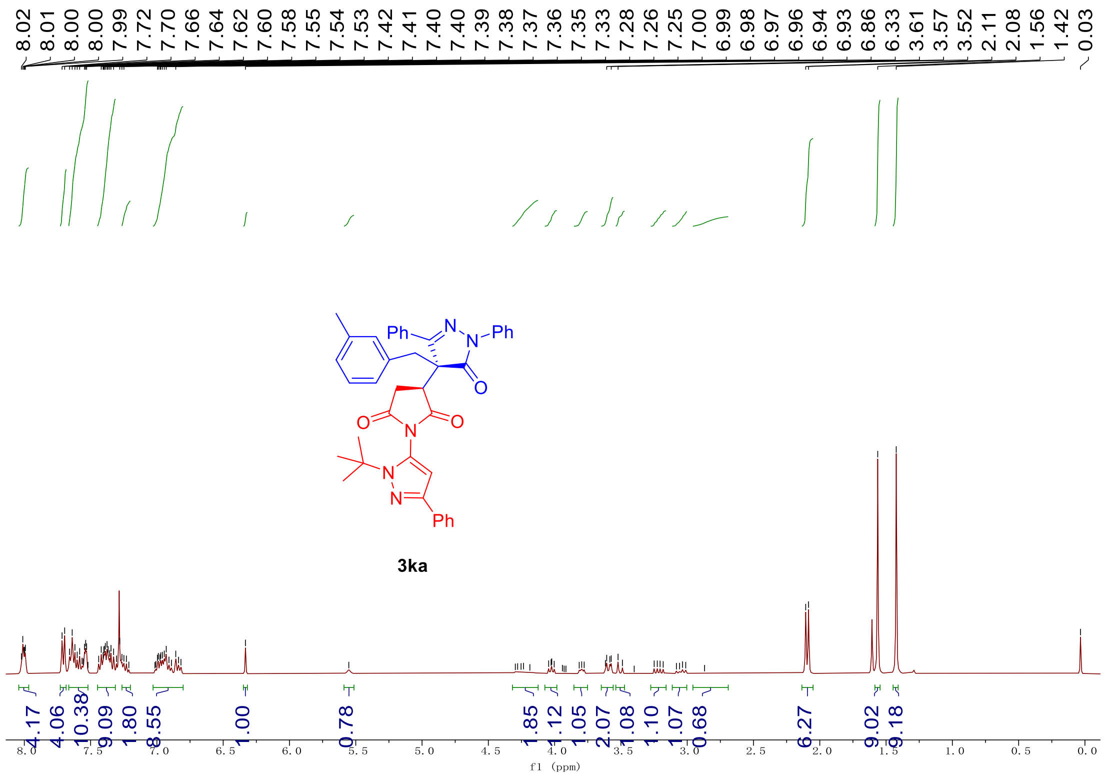

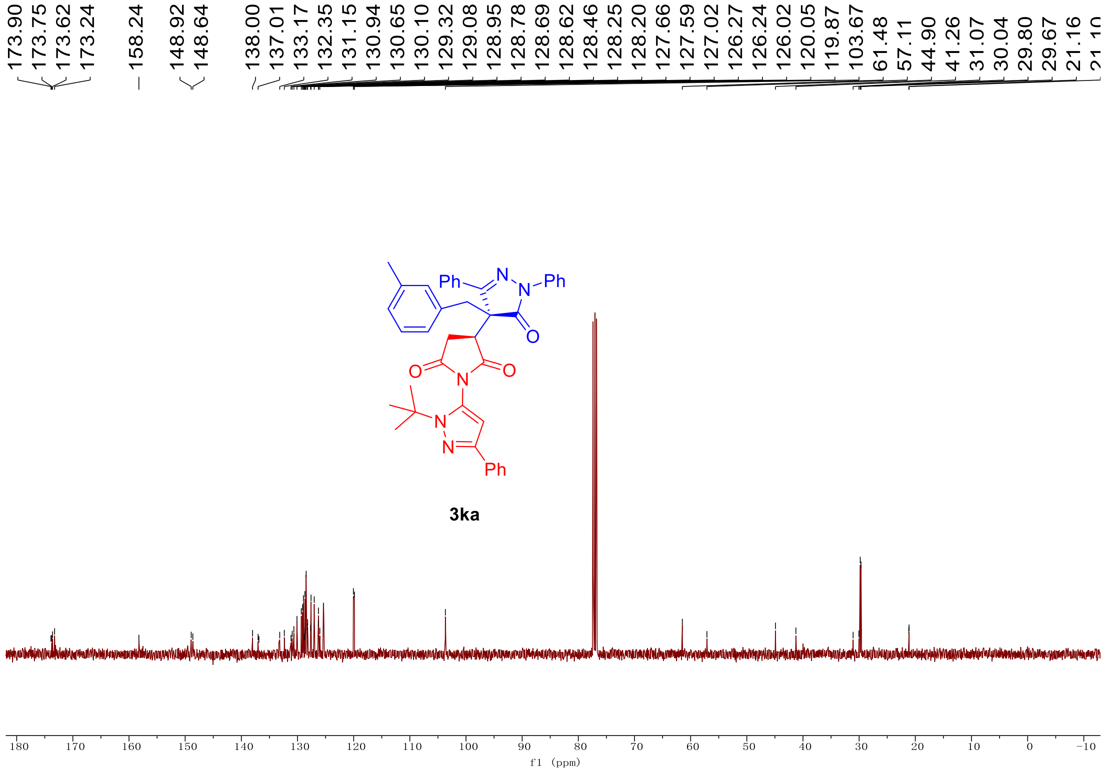

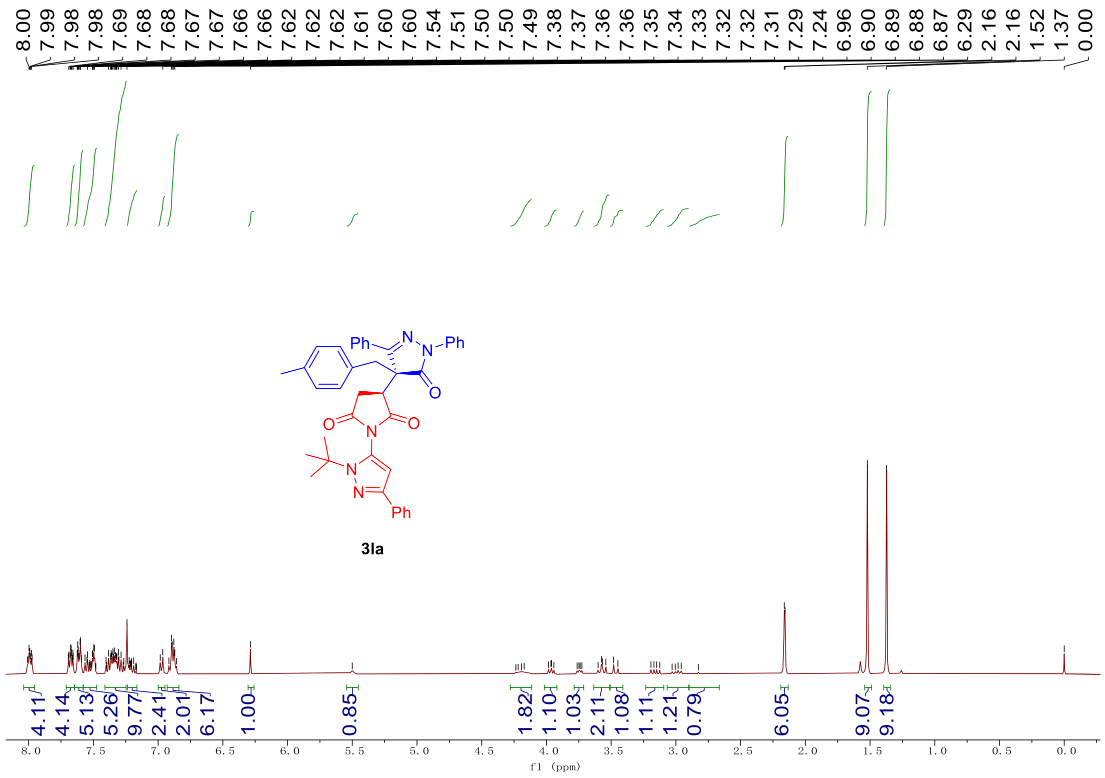
8.00 7.99 7.98 7.98 7.69 7.68 7.68 7.67 7.67 7.66 7.66 7.62 7.62 7.62 7.61 7.60 7.60 7.54 7.51 7.50 7.50 7.49 7.38 7.37 7.36 7.36 7.35 7.34 7.33 7.32 7.32 7.31 7.29 7.24 6.96 6.90 6.89 6.88 6.87 6.29 2.16 2.16 1.52 1.37 0.00
Ph
N
N
Ph
O
O
O
N
N
N
Ph
3la
4.11 4.14 5.13 5.26 9.77 2.41 2.01 6.17 1.00 0.85 1.82 1.10 1.03 2.11 1.08 1.11 1.21 0.79 6.05 9.07 9.18
8.0 7.5 7.0 6.5 6.0 5.5 5.0 4.5 4.0 3.5 3.0 2.5 2.0 1.5 1.0 0.5 0.0
f1 (ppm)

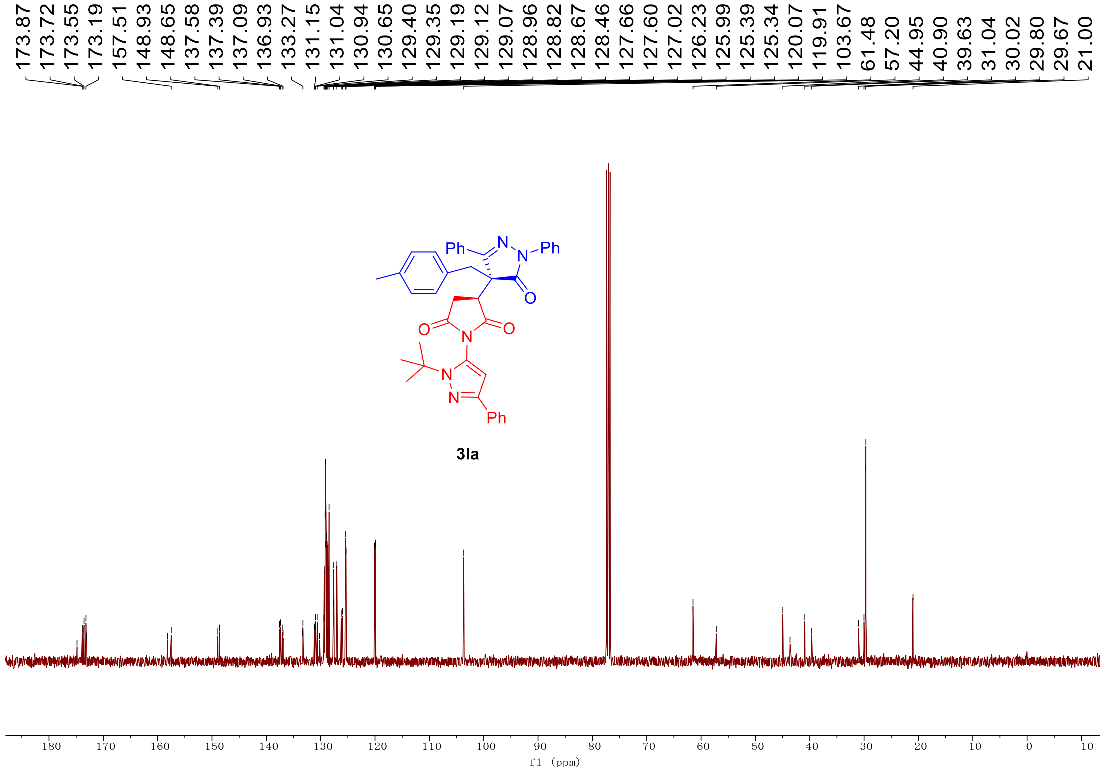
173.87 173.72 173.55 173.19 157.51 148.93 148.65 137.58 137.39 137.09 136.93 133.27 131.15 131.04 130.94 130.65 129.40 129.35 129.19 129.12 129.07 128.96 128.82 128.67 128.46 127.66 127.60 127.02 126.23 125.99 125.39 125.34 120.07 119.91 103.67 61.48 57.20 44.95 40.90 39.63 31.04 30.02 29.80 29.67 21.00
Ph
N
N
Ph
O
O
O
N
N
N
Ph
3la
180 170 160 150 140 130 120 110 100 90 80 70 60 50 40 30 20 10 0 -10
f1 (ppm)

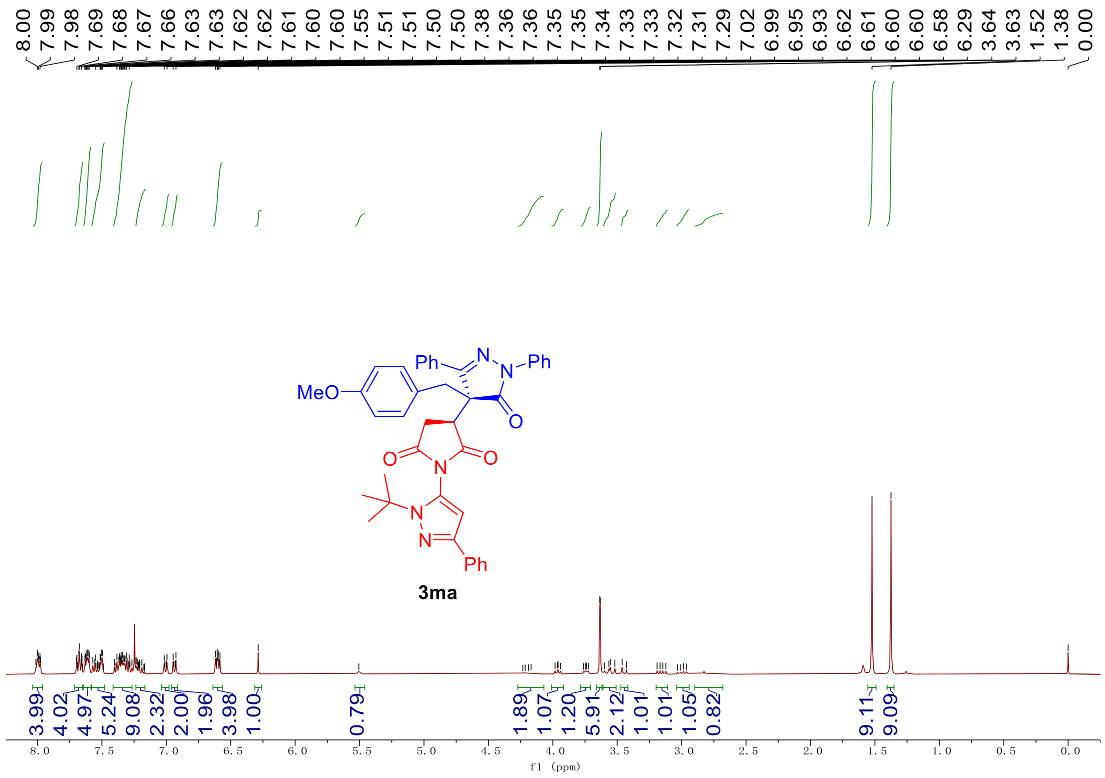
8.00 7.99 7.98 7.69 7.68 7.67 7.66 7.63 7.63 7.62 7.62 7.61 7.60 7.60 7.55 7.51 7.51 7.50 7.50 7.38 7.36 7.36 7.35 7.35 7.34 7.33 7.33 7.32 7.31 7.29 7.02 6.99 6.95 6.93 6.62 6.61 6.60 6.60 6.58 6.29 3.64 3.63 1.52 1.38 0.00
Ph
N
N
Ph
MeO
O
O
N
O
N
N
Ph
3ma
3.99 4.02 4.97 5.24 9.08 2.32 2.00 1.96 3.98 1.00 0.79 1.89 1.07 1.20 5.91 2.12 1.01 1.01 1.05 0.82 9.11 9.09
f1 (ppm)

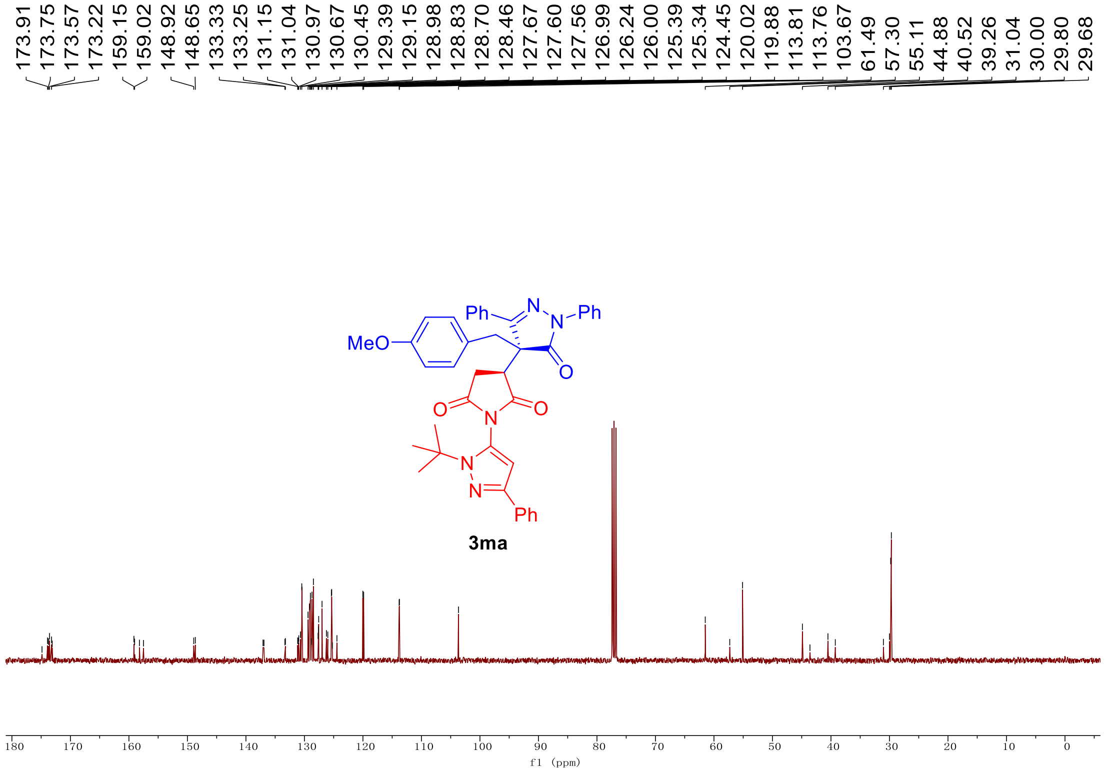
173.91 173.75 173.57 173.22 159.15 159.02 148.92 148.65 133.33 133.25 131.15 131.04 130.97 130.67 130.45 129.39 129.15 128.98 128.83 128.70 128.46 127.67 127.60 127.56 126.99 126.24 126.00 125.39 125.34 124.45 120.02 119.88 113.81 113.76 103.67 61.49 57.30 55.11 44.88 40.52 39.26 31.04 30.00 29.80 29.68
Ph
N
N
Ph
MeO
O
O
N
O
N
N
Ph
3ma
f1 (ppm)

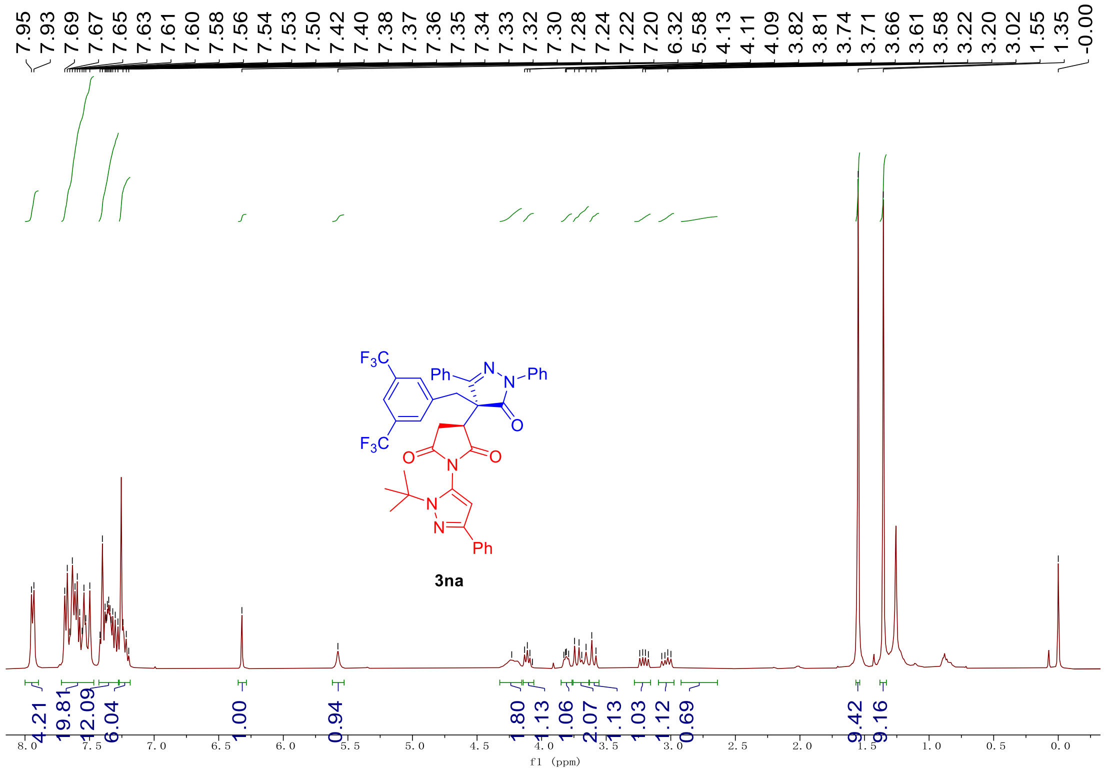
7.95 7.93 7.69 7.67 7.65 7.63 7.61 7.60 7.58 7.56 7.54 7.53 7.50 7.42 7.40 7.38 7.37 7.36 7.35 7.34 7.33 7.32 7.30 7.28 7.24 7.22 7.20 6.32 5.58 4.13 4.11 4.09 3.82 3.81 3.74 3.71 3.66 3.61 3.58 3.22 3.20 3.02 1.55 1.35 -0.00
4.21 19.81 12.09 6.04 1.00 0.94 1.80 1.13 1.06 2.07 1.13 1.03 1.12 0.69 9.42 9.16
3na
f1 (ppm)

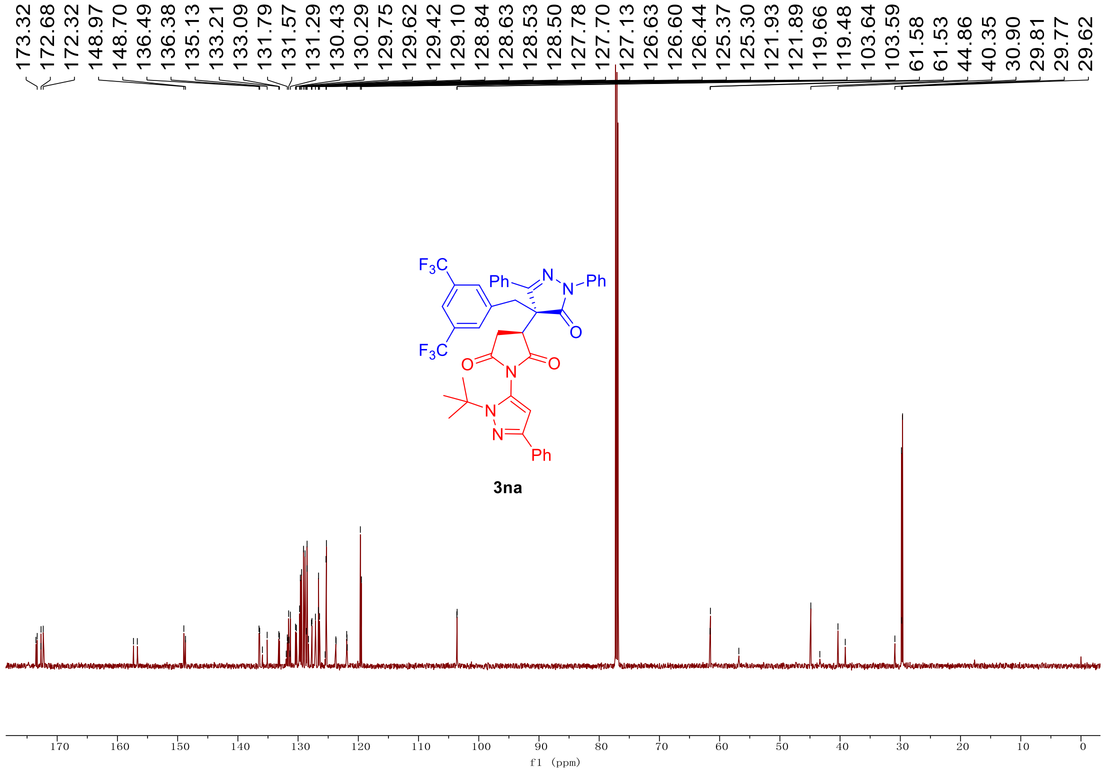
173.32 172.68 172.32 148.97 148.70 136.49 136.38 135.13 133.21 133.09 131.79 131.57 131.29 130.43 130.29 129.75 129.62 129.42 129.10 128.84 128.63 128.53 128.50 127.78 127.70 127.13 126.63 126.60 126.44 125.37 125.30 121.93 121.89 119.66 119.48 103.64 103.59 61.58 61.53 44.86 40.35 30.90 29.81 29.77 29.62
3na
f1 (ppm)

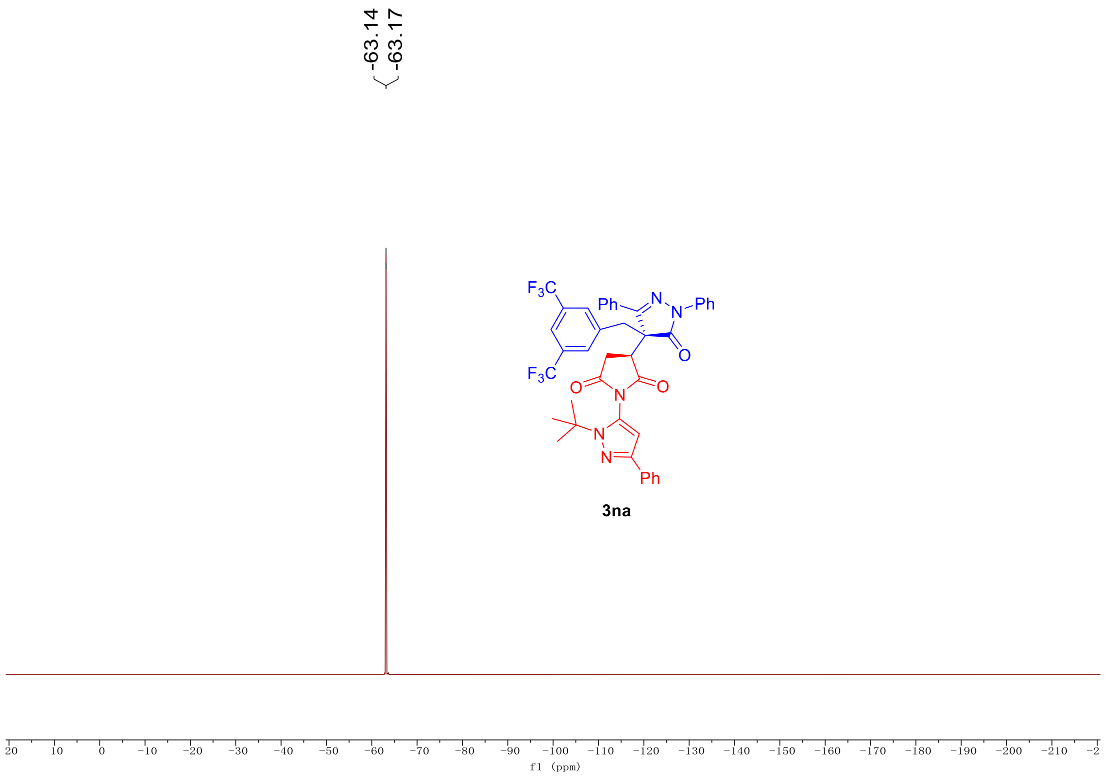
-63.14
-63.17
F3C
Ph
N
N
Ph
O
O
O
N
N
N
Ph
3na
f1 (ppm)

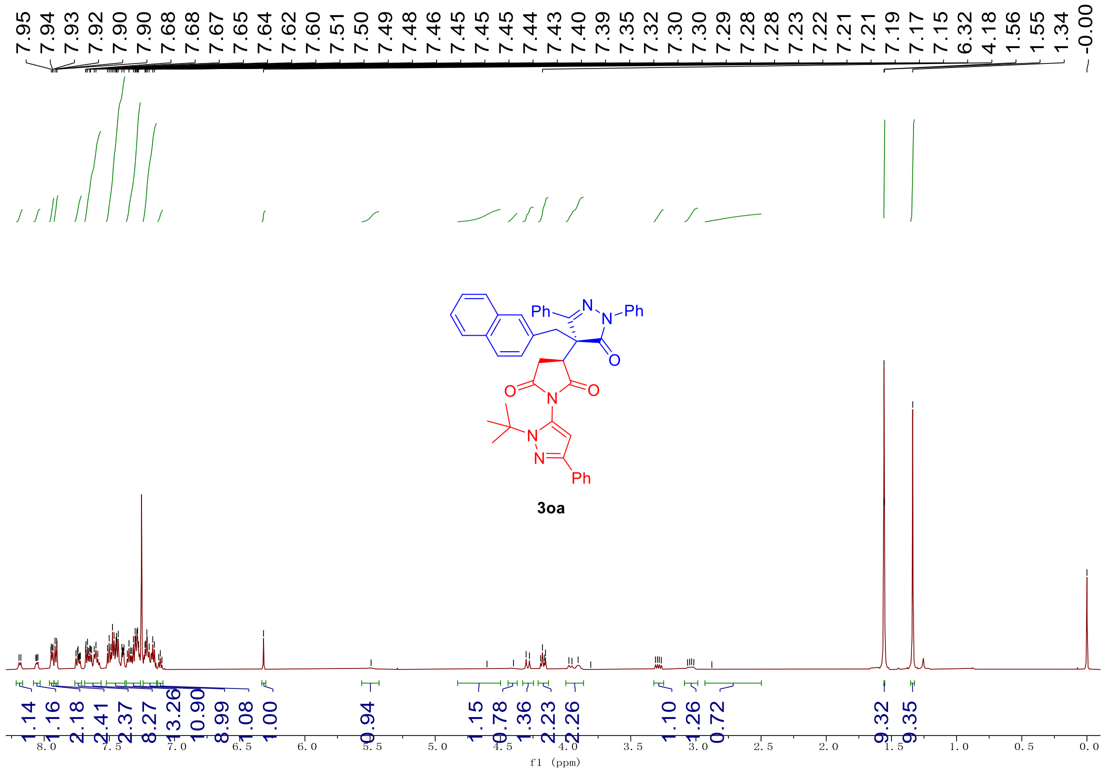
7.95
7.94
7.93
7.92
7.90
7.90
7.68
7.68
7.67
7.65
7.64
7.62
7.60
7.51
7.50
7.49
7.48
7.46
7.45
7.45
7.45
7.44
7.43
7.40
7.39
7.35
7.32
7.30
7.30
7.29
7.28
7.28
7.23
7.22
7.21
7.21
7.19
7.17
7.15
6.32
4.18
1.56
1.55
1.34
-0.00
Ph
N
N
Ph
O
O
O
N
N
N
Ph
3oa
1.14
1.16
2.18
2.41
2.37
8.27
13.26
10.90
8.99
1.08
1.00
0.94
1.15
0.78
1.36
2.23
2.26
1.10
1.26
0.72
9.32
9.35
f1 (ppm)

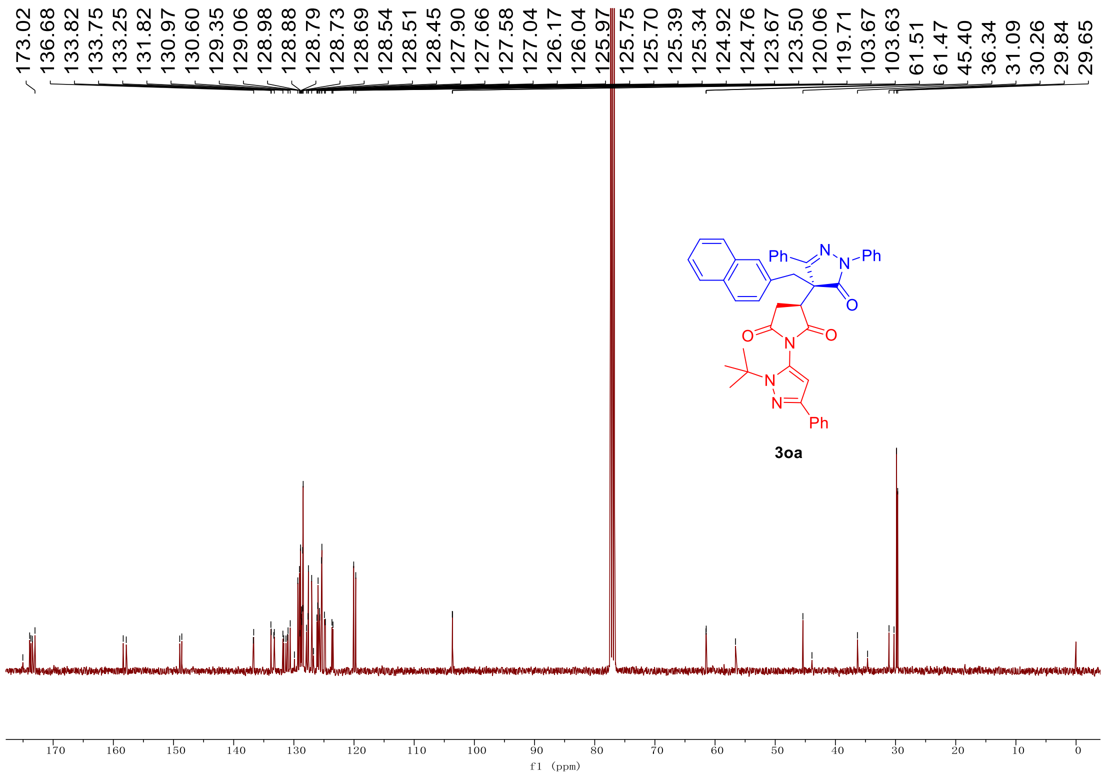
173.02
136.68
133.82
133.75
133.25
131.82
130.97
130.60
129.35
129.06
128.98
128.88
128.79
128.73
128.69
128.54
128.51
128.45
127.90
127.66
127.58
127.04
126.17
126.04
125.97
125.75
125.70
125.39
125.34
124.92
124.76
123.67
123.50
120.06
119.71
103.67
103.63
61.51
61.47
45.40
36.34
31.09
30.26
29.84
29.65
Ph
N
N
Ph
O
O
N
O
N
N
Ph
3oa
f1 (ppm)

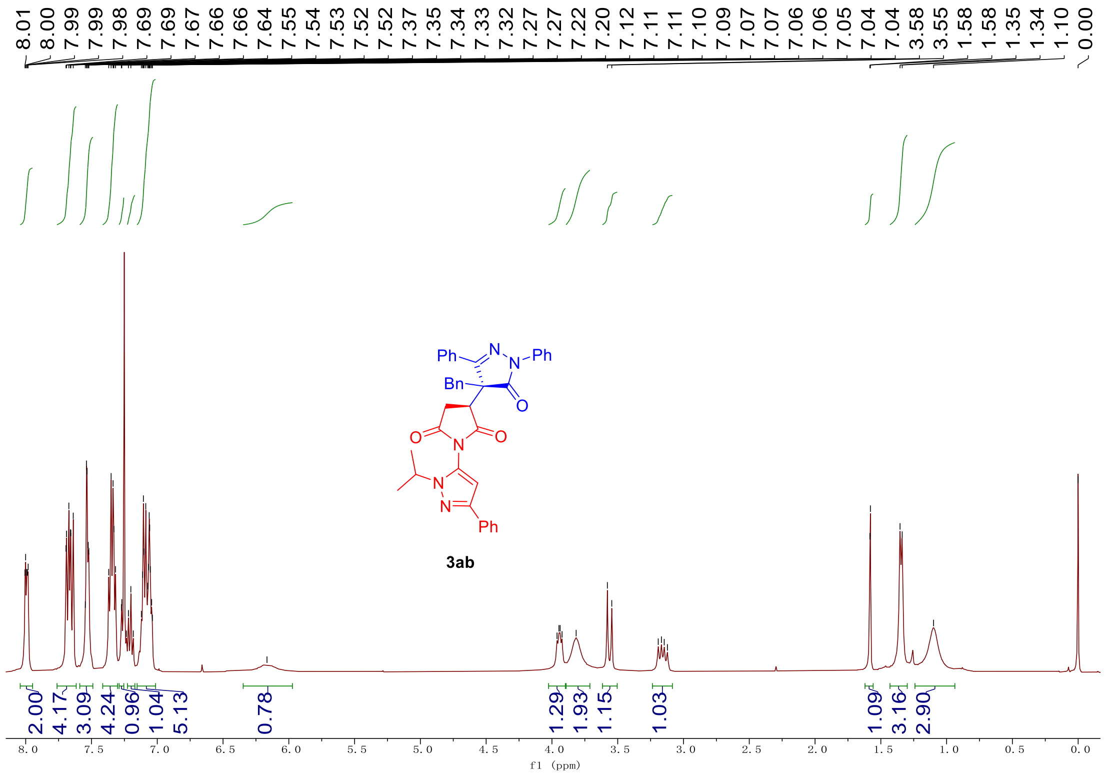
8.01
8.00
7.99
7.99
7.98
7.69
7.69
7.67
7.66
7.66
7.64
7.55
7.54
7.53
7.52
7.52
7.37
7.35
7.34
7.33
7.32
7.27
7.27
7.22
7.20
7.12
7.11
7.11
7.10
7.09
7.07
7.07
7.06
7.06
7.05
7.04
7.04
3.58
3.55
1.58
1.35
1.34
1.10
0.00
Ph
N
N
Ph
Bn
O
O
N
O
N
N
Ph
3ab
2.00
4.17
3.09
4.24
0.96
1.04
5.13
0.78
1.29
1.93
1.15
1.03
1.09
3.16
2.90
f1 (ppm)

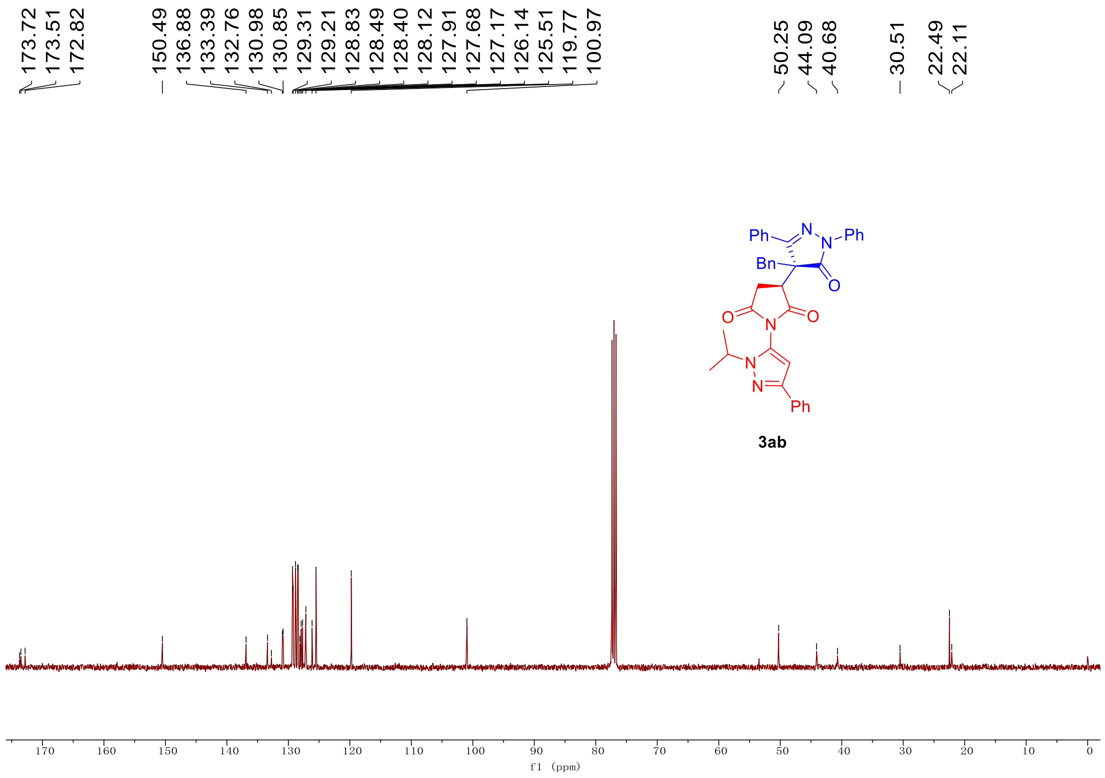
173.72
173.51
172.82
150.49
136.88
133.39
132.76
130.98
130.85
129.31
129.21
128.83
128.49
128.40
128.12
127.91
127.68
127.17
126.14
125.51
119.77
100.97
50.25
44.09
40.68
30.51
22.49
22.11
Ph
N
N
Ph
Bn
O
O
O
N
N
N
Ph
f1 (ppm)

3ab

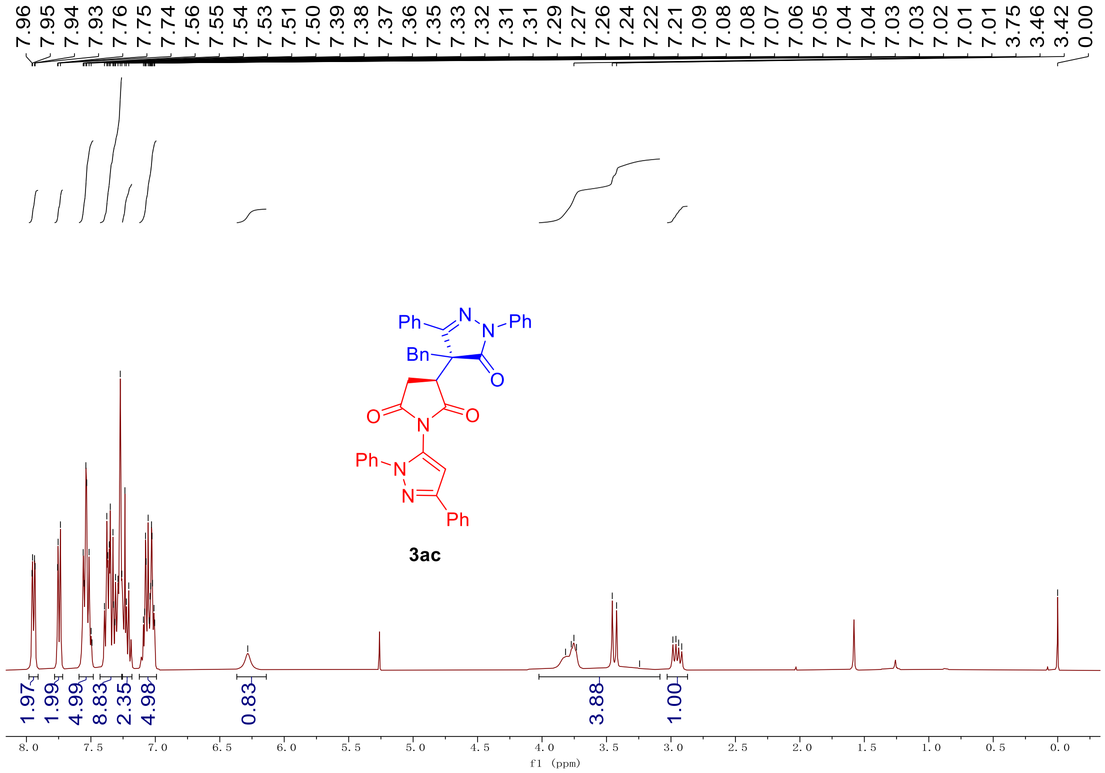
7.96
7.95
7.94
7.93
7.76
7.75
7.74
7.56
7.55
7.54
7.53
7.51
7.50
7.39
7.38
7.37
7.36
7.35
7.33
7.32
7.31
7.31
7.29
7.27
7.26
7.24
7.22
7.21
7.09
7.08
7.08
7.07
7.06
7.05
7.04
7.04
7.03
7.03
7.02
7.01
7.01
3.75
3.46
3.42
0.00
Ph
N
N
Ph
Bn
O
O
O
N
Ph
N
N
Ph
1.97
1.99
4.99
8.83
2.35
4.98
0.83
3.88
1.00
f1 (ppm)

3ac

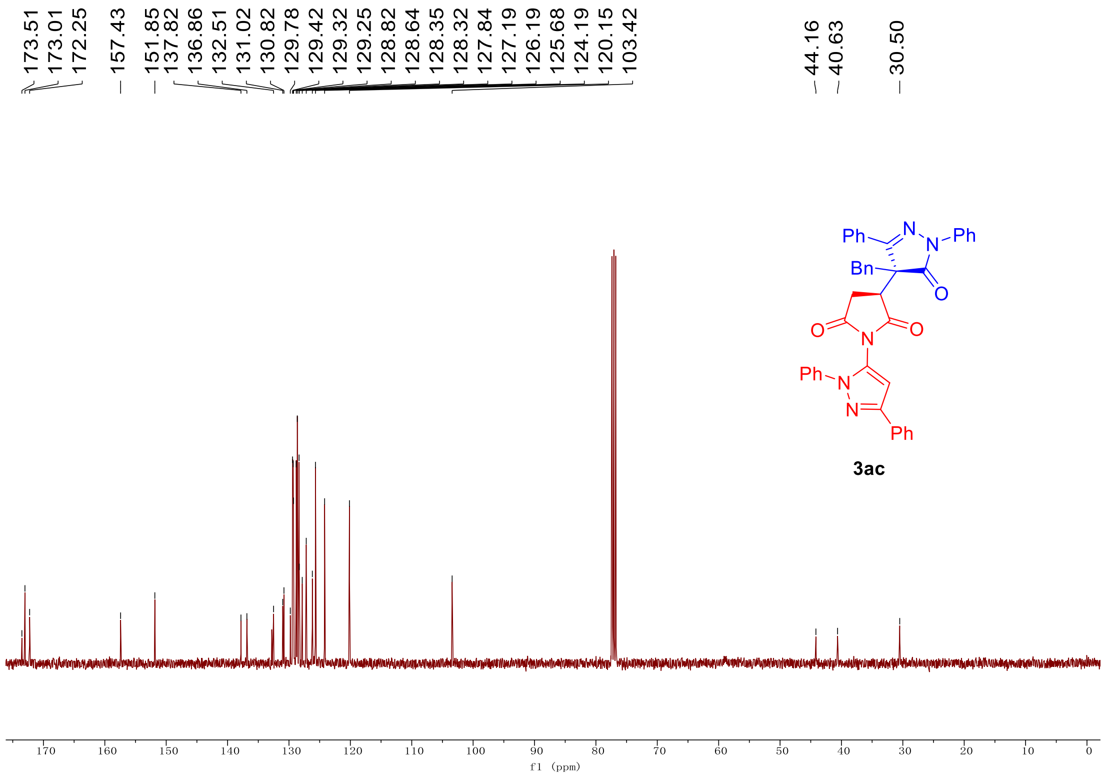
173.51 173.01 172.25 157.43 151.85 137.82 136.86 132.51 131.02 130.82 129.78 129.42 129.32 129.25 128.82 128.64 128.35 128.32 127.84 127.19 126.19 125.68 124.19 120.15 103.42 44.16 40.63 30.50
Ph N N Ph Bn O O N O Ph N N Ph
170 160 150 140 130 120 110 100 90 80 70 60 50 40 30 20 10 0
f1 (ppm)

3ac

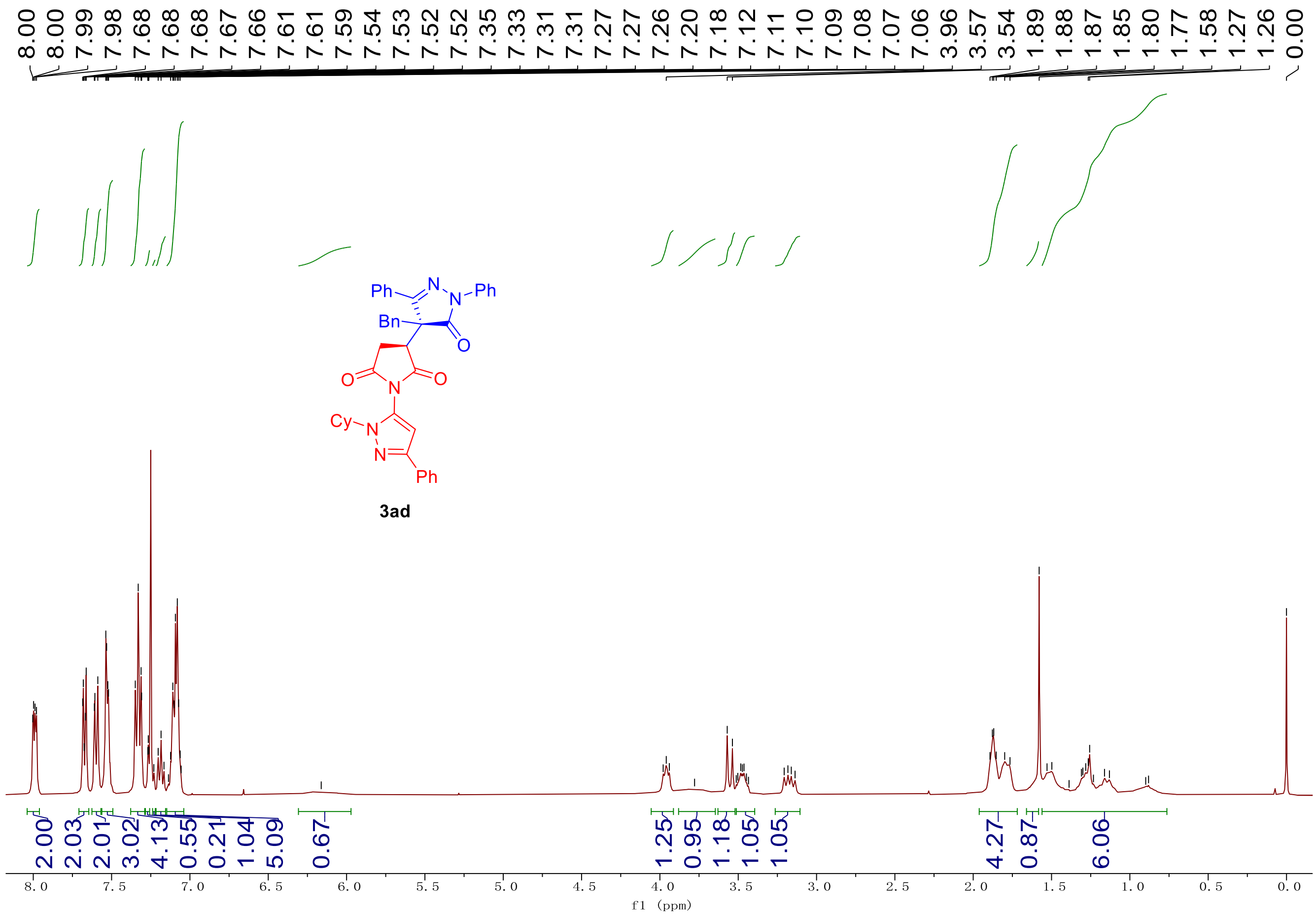
8.00 8.00 7.99 7.98 7.68 7.68 7.68 7.67 7.66 7.61 7.61 7.59 7.54 7.53 7.52 7.52 7.35 7.33 7.31 7.31 7.27 7.27 7.26 7.20 7.18 7.12 7.11 7.10 7.09 7.08 7.07 7.06 3.96 3.57 3.54 1.89 1.88 1.87 1.85 1.80 1.77 1.58 1.27 1.26 0.00
Ph N N Ph Bn O O N O Cy N N Ph
2.00 2.03 2.01 3.02 4.13 0.55 0.21 1.04 5.09 0.67 1.25 0.95 1.18 1.05 1.05 4.27 0.87 6.06
8.0 7.5 7.0 6.5 6.0 5.5 5.0 4.5 4.0 3.5 3.0 2.5 2.0 1.5 1.0 0.5 0.0
f1 (ppm)

3ad

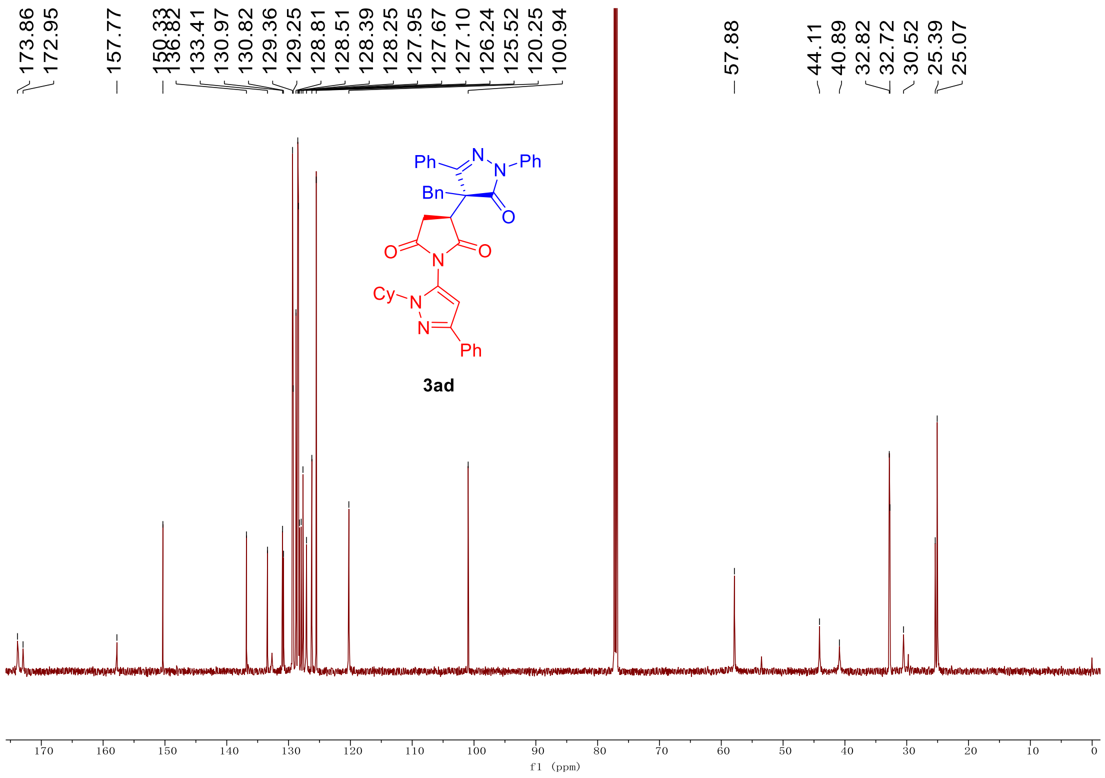
173.86
172.95
157.77
150.33
136.82
133.41
130.97
130.82
129.36
129.25
128.81
128.51
128.39
128.25
127.95
127.67
127.10
126.24
125.52
120.25
100.94
57.88
44.11
40.89
32.82
32.72
30.52
25.39
25.07
3ad
f1 (ppm)

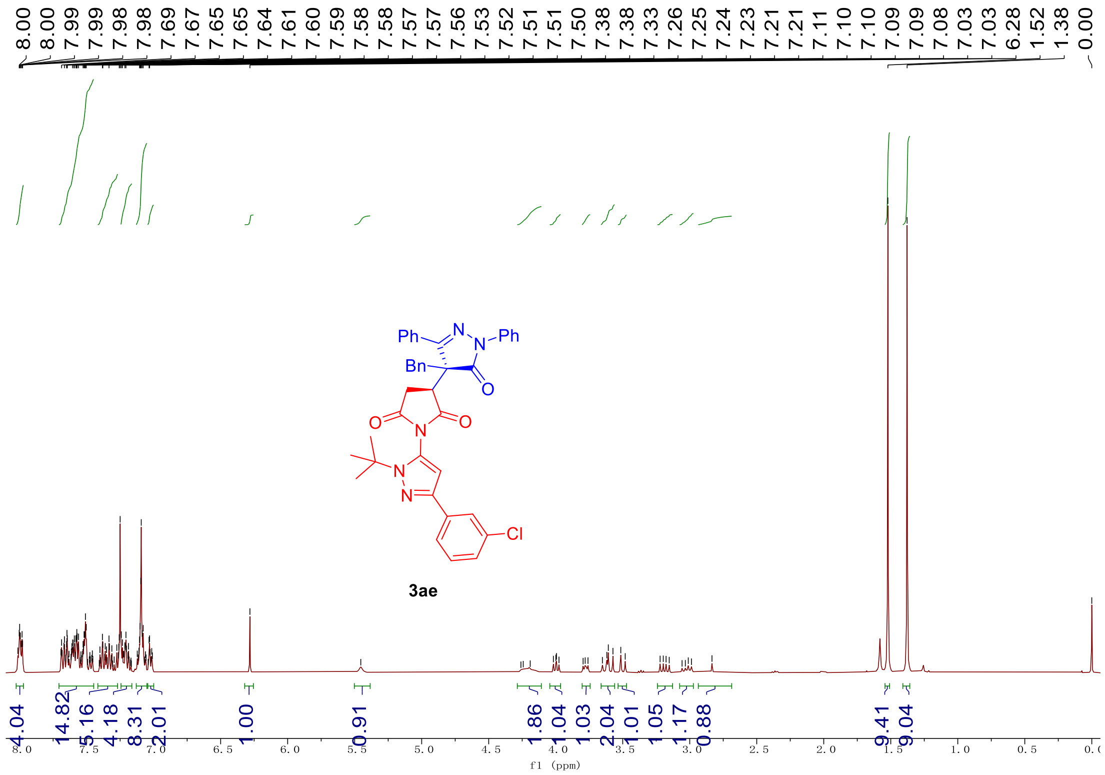
8.00
8.00
7.99
7.99
7.98
7.98
7.69
7.67
7.65
7.65
7.64
7.61
7.60
7.59
7.58
7.58
7.57
7.57
7.56
7.53
7.52
7.51
7.51
7.50
7.38
7.38
7.33
7.26
7.25
7.24
7.23
7.21
7.21
7.11
7.10
7.10
7.09
7.09
7.08
7.03
7.03
6.28
1.52
1.38
0.00
3ae
4.04
14.82
5.16
4.18
8.31
2.01
1.00
0.91
1.86
1.04
1.03
2.04
1.01
1.05
1.17
0.88
9.41
9.04
f1 (ppm)

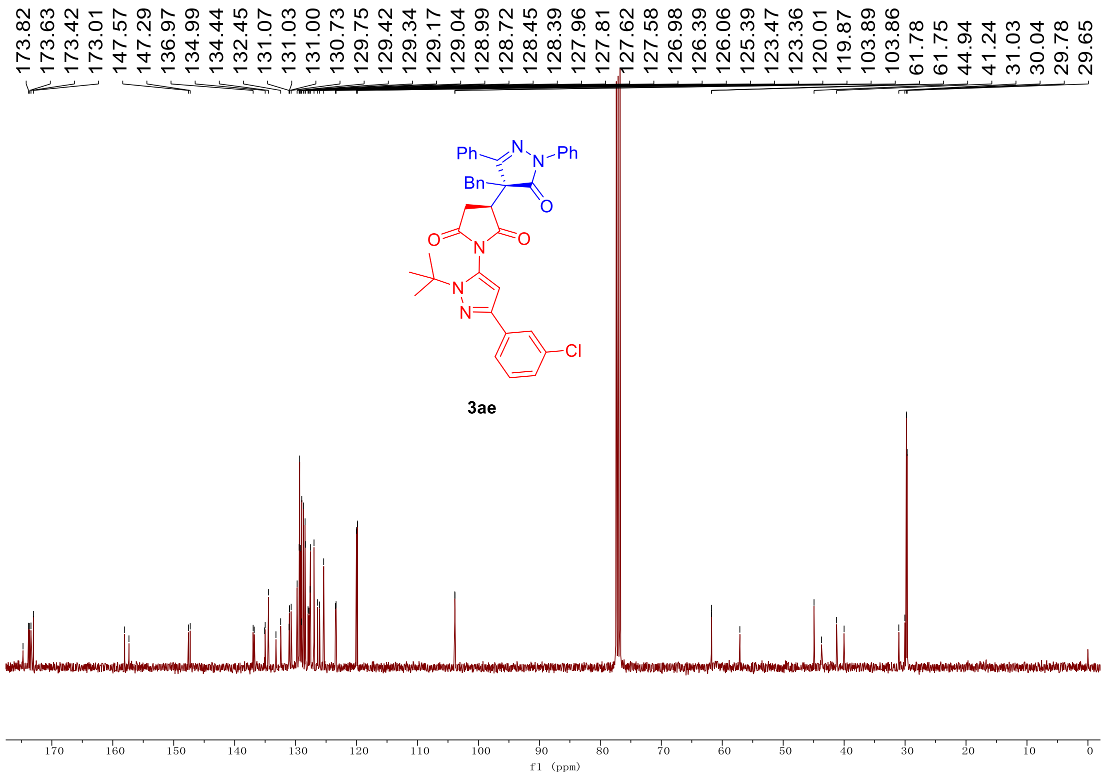

173.82
173.63
173.42
173.01
147.57
147.29
136.97
134.99
134.44
132.45
131.07
131.03
131.00
130.73
129.75
129.42
129.34
129.17
129.04
128.99
128.72
128.45
128.39
127.96
127.81
127.62
127.58
126.98
126.39
126.06
125.39
123.47
123.36
120.01
119.87
103.89
103.86
61.78
61.75
44.94
41.24
31.03
30.04
29.78
29.65
Ph
N
N
Ph
Bn
O
O
O
N
N
N
Cl
3ae
170
160
150
140
130
120
110
100
90
80
70
60
50
40
30
20
10
0
f1 (ppm)

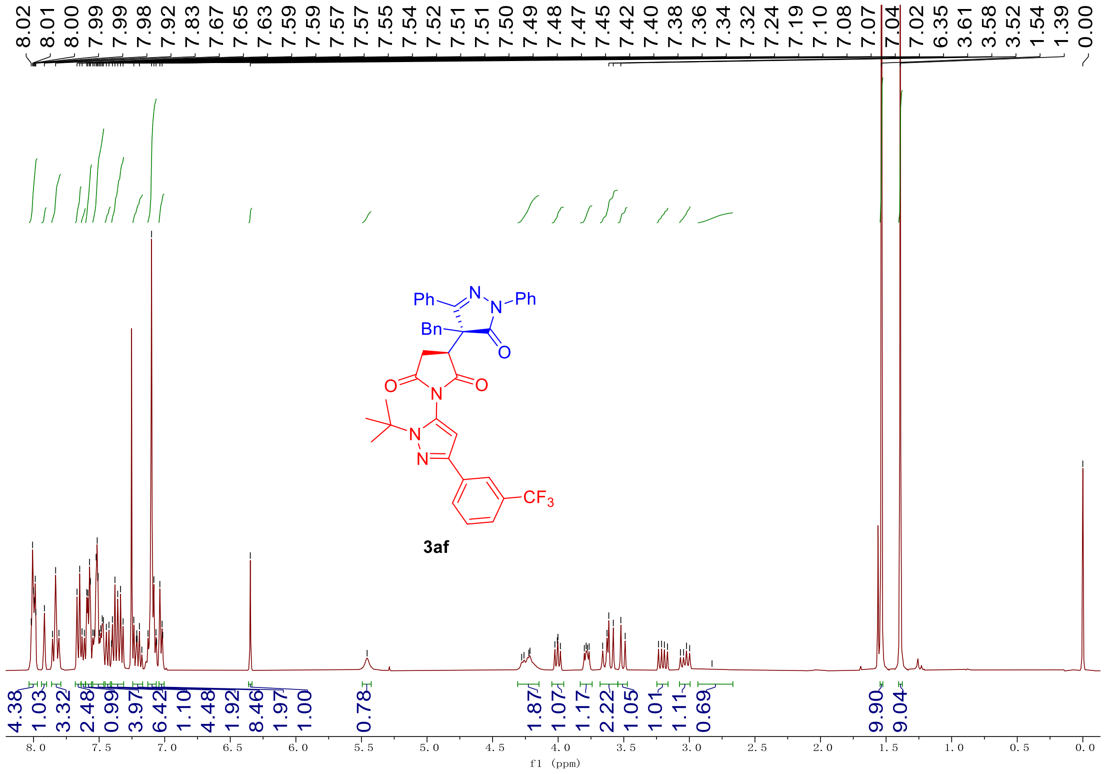

8.02
8.01
8.00
7.99
7.99
7.98
7.92
7.83
7.67
7.65
7.63
7.59
7.59
7.57
7.55
7.54
7.52
7.51
7.51
7.50
7.49
7.48
7.47
7.45
7.42
7.40
7.38
7.36
7.34
7.32
7.24
7.19
7.10
7.08
7.07
7.04
7.02
6.35
3.61
3.58
3.52
1.54
1.39
0.00
Ph
N
N
Ph
Bn
O
O
O
N
N
N
CF_3
3af
4.38
1.03
3.32
2.48
0.99
3.97
6.42
1.10
4.48
1.92
8.46
1.97
1.00
0.78
1.87
1.07
1.17
2.22
1.05
1.01
1.11
0.69
9.90
9.04
8.0
7.5
7.0
6.5
6.0
5.5
5.0
4.5
4.0
3.5
3.0
2.5
2.0
1.5
1.0
0.5
0.0
f1 (ppm)

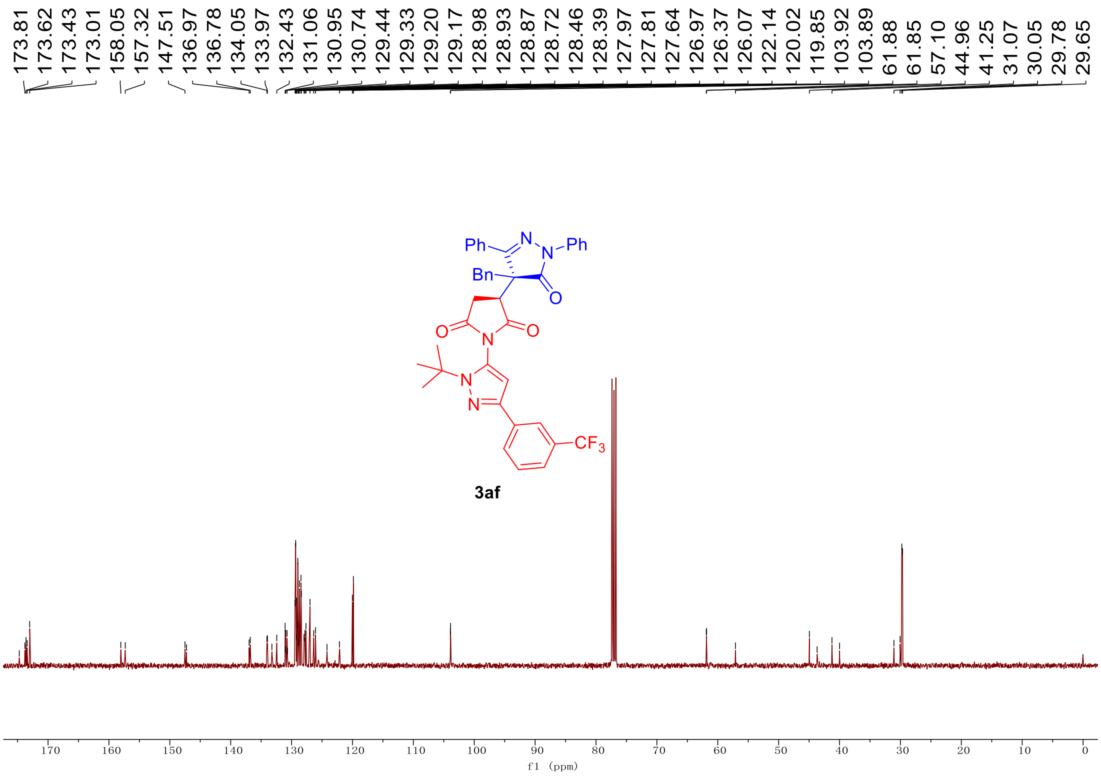
173.81
173.62
173.43
173.01
158.05
157.32
147.51
136.97
136.78
134.05
133.97
132.43
131.06
130.95
130.74
129.44
129.33
129.20
129.17
128.98
128.93
128.87
128.72
128.46
128.39
127.97
127.81
127.64
126.97
126.37
126.07
122.14
120.02
119.85
103.92
103.89
61.88
61.85
57.10
44.96
41.25
31.07
30.05
29.78
29.65
Ph
N
N
Ph
Bn
O
O
N
O
N
N
CF3
3af
f1 (ppm)

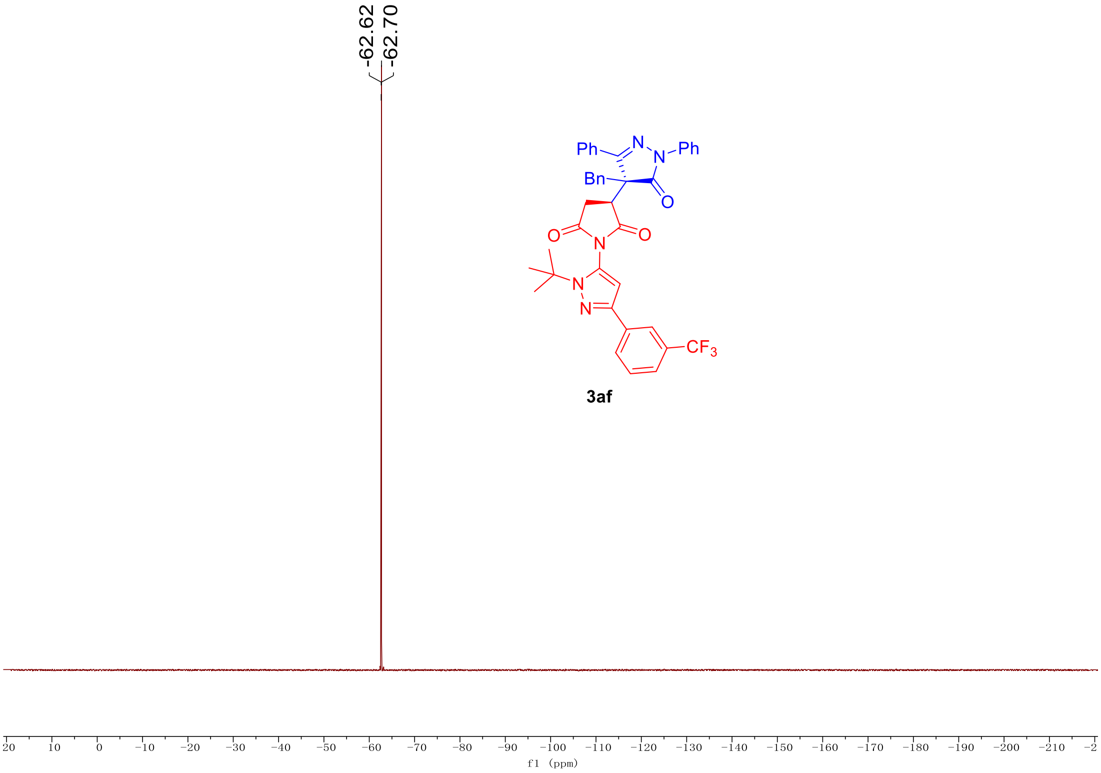
-62.62
-62.70
Ph
N
N
Ph
Bn
O
O
N
O
N
N
CF3
3af
f1 (ppm)

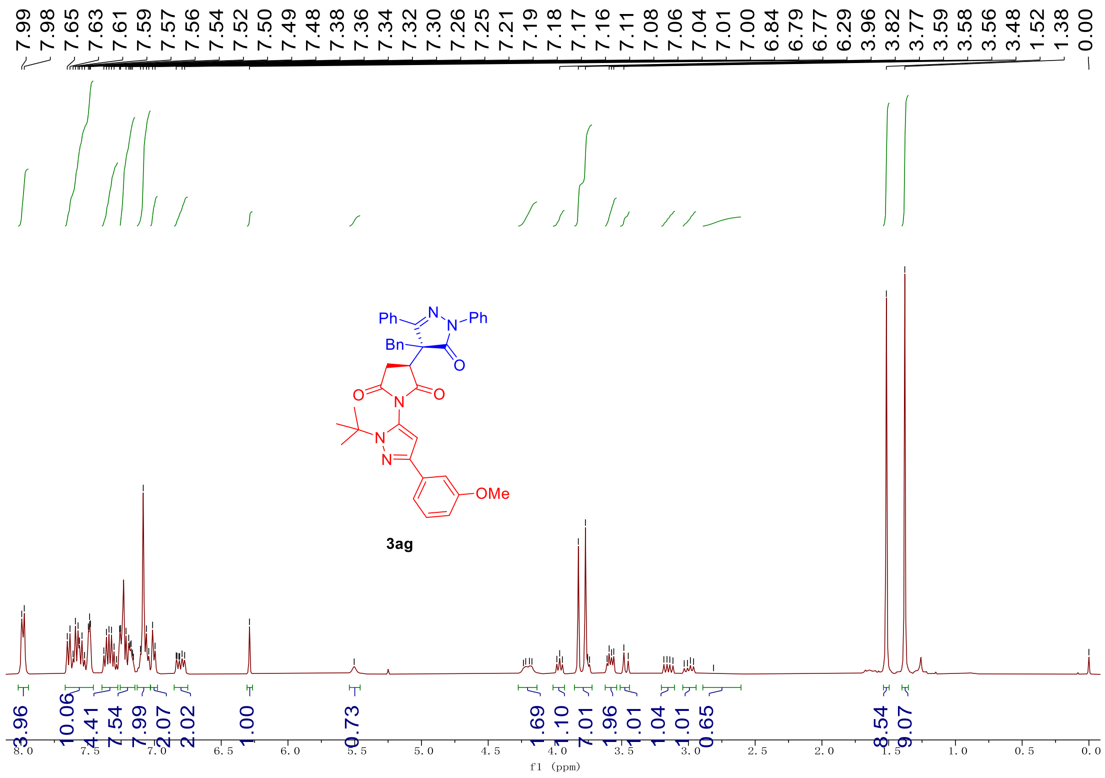
7.99 7.98 7.65 7.63 7.61 7.59 7.57 7.56 7.54 7.52 7.50 7.49 7.48 7.38 7.36 7.34 7.32 7.30 7.26 7.25 7.21 7.19 7.18 7.17 7.16 7.11 7.08 7.06 7.04 7.01 7.00 6.84 6.79 6.77 6.29 3.96 3.82 3.77 3.59 3.58 3.56 3.48 1.52 1.38 -0.00
Ph
N
N
Ph
Bn
O
O
O
N
N
N
OMe
3ag
3.96 10.06 4.41 7.54 7.99 2.07 2.02 1.00 0.73 1.69 1.10 7.01 1.96 1.01 1.04 1.01 0.65 8.54 9.07
f1 (ppm)

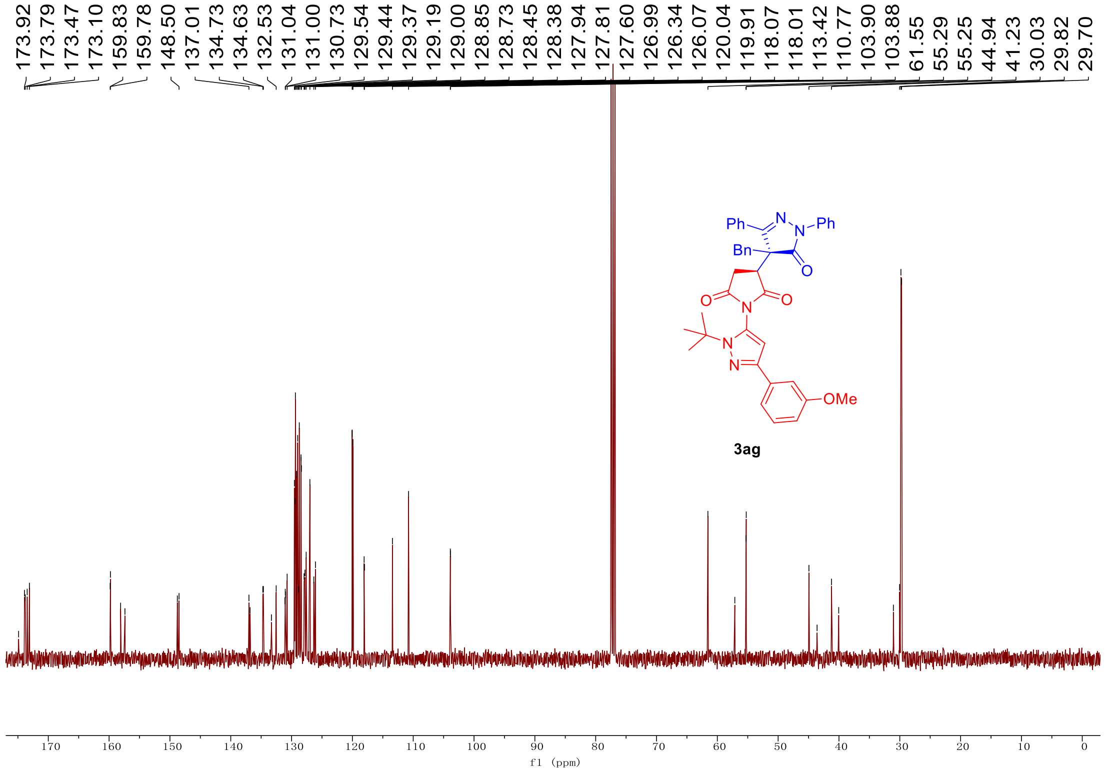
173.92 173.79 173.47 173.10 159.83 159.78 148.50 137.01 134.73 134.63 132.53 131.04 131.00 130.73 129.54 129.44 129.37 129.19 129.00 128.85 128.73 128.45 128.38 127.94 127.81 127.60 126.99 126.34 126.07 120.04 119.91 118.07 118.01 113.42 110.77 103.90 103.88 61.55 55.29 55.25 44.94 41.23 30.03 29.82 29.70
Ph
N
N
Ph
Bn
O
O
O
N
N
N
OMe
3ag
f1 (ppm)

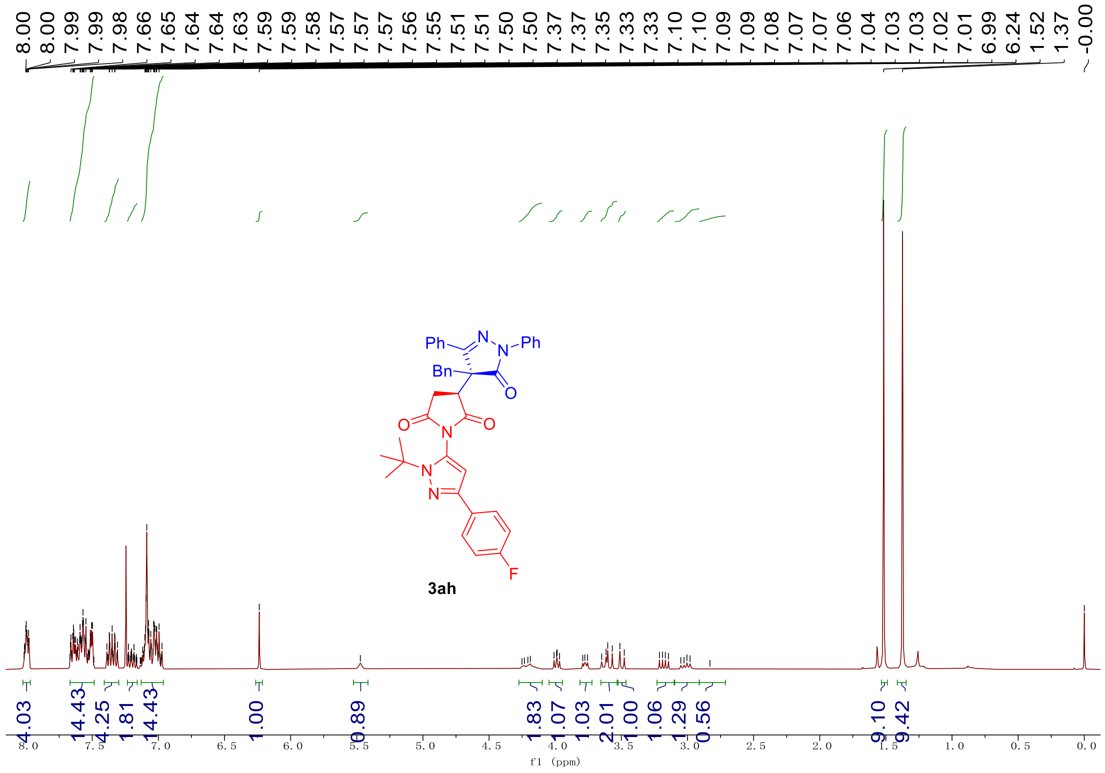
3ah
f1 (ppm)

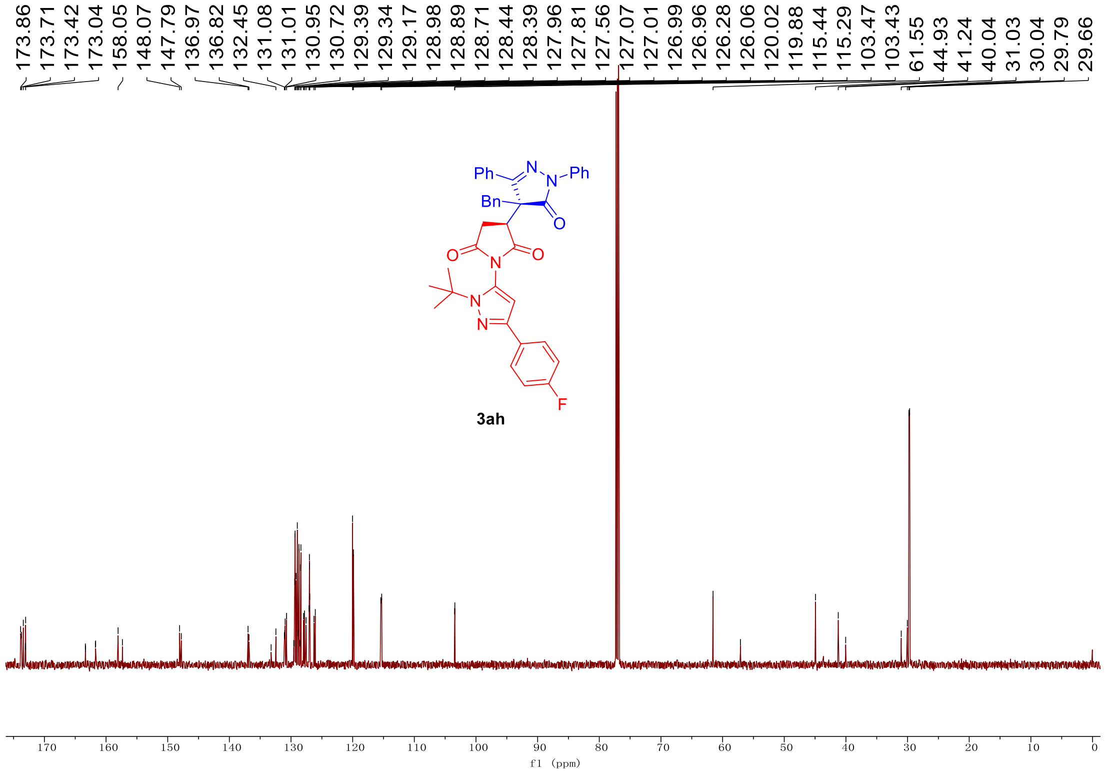
3ah
f1 (ppm)

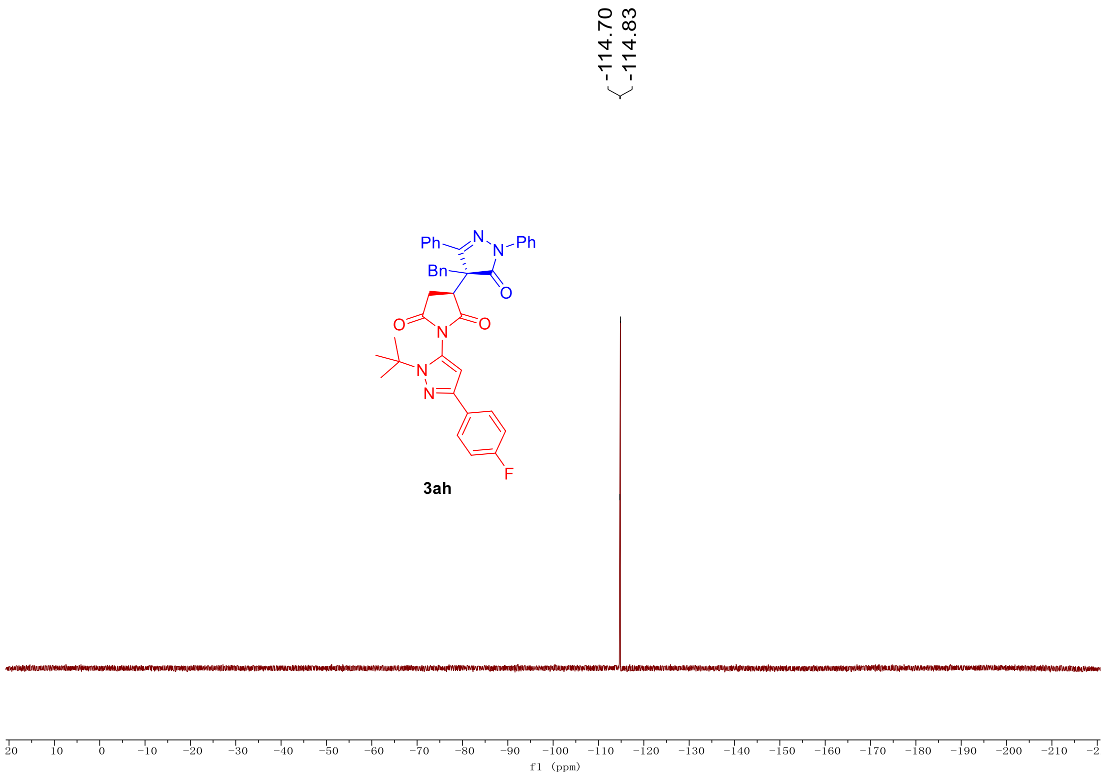
-114.70
-114.83
Ph
N
N
Ph
Bn
O
O
O
N
N
N
F
f1 (ppm)

3ah

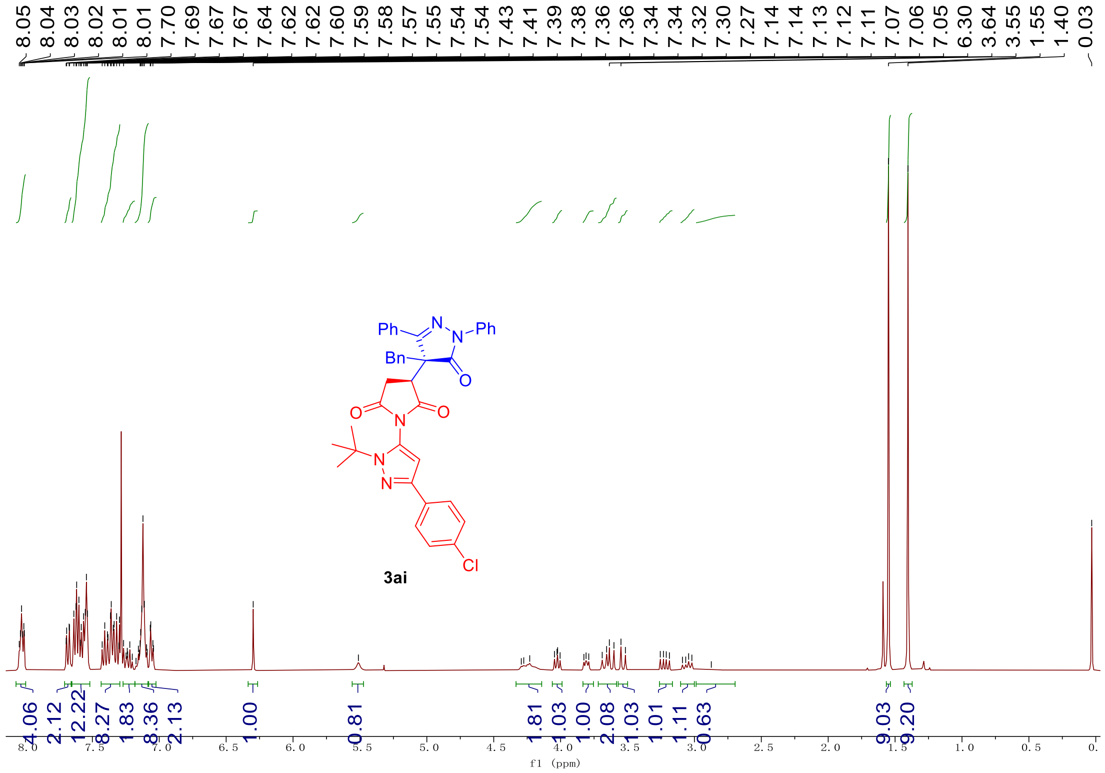
8.05 8.04 8.03 8.02 8.01 8.01 7.70 7.69 7.67 7.67 7.64 7.62 7.62 7.60 7.59 7.58 7.57 7.55 7.54 7.54 7.43 7.41 7.39 7.38 7.36 7.36 7.34 7.34 7.32 7.30 7.27 7.14 7.14 7.13 7.12 7.11 7.07 7.06 7.05 6.30 3.64 3.55 1.55 1.40 0.03
Ph
N
N
Ph
Bn
O
O
O
N
N
N
Cl
4.06 2.12 12.22 8.27 1.83 8.36 2.13 1.00 0.81 1.81 1.03 1.00 2.08 1.03 1.01 1.11 0.63 9.03 9.20
f1 (ppm)

3ai

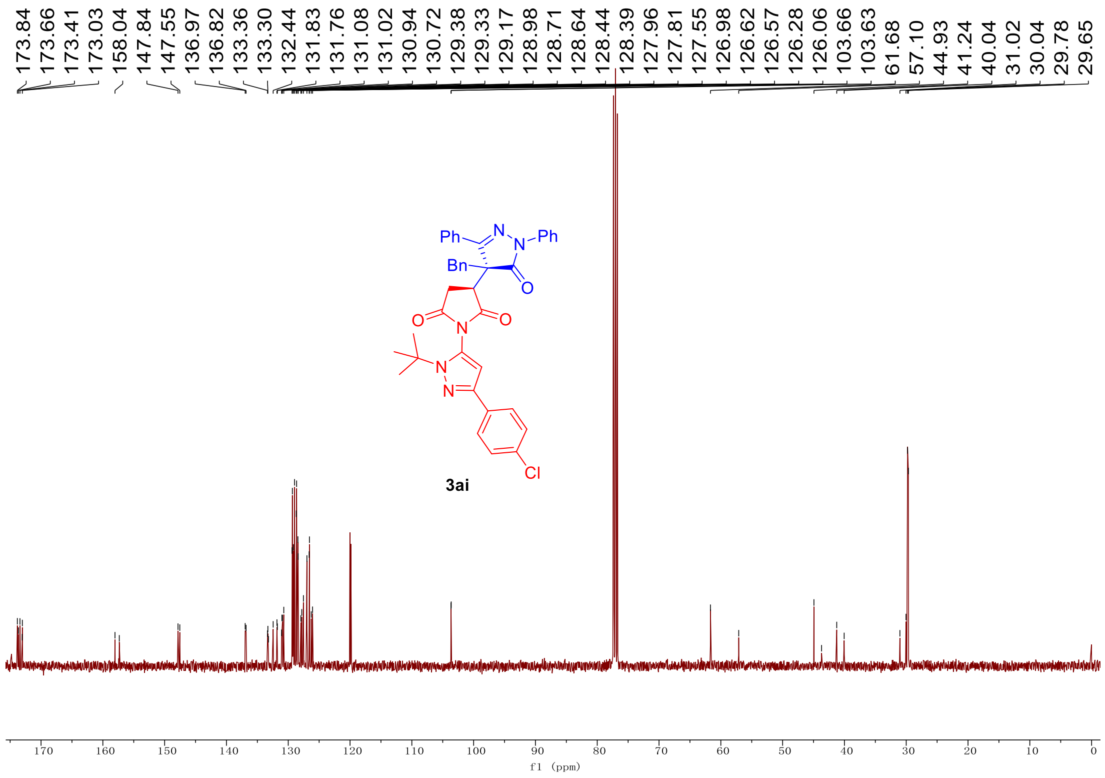
3ai

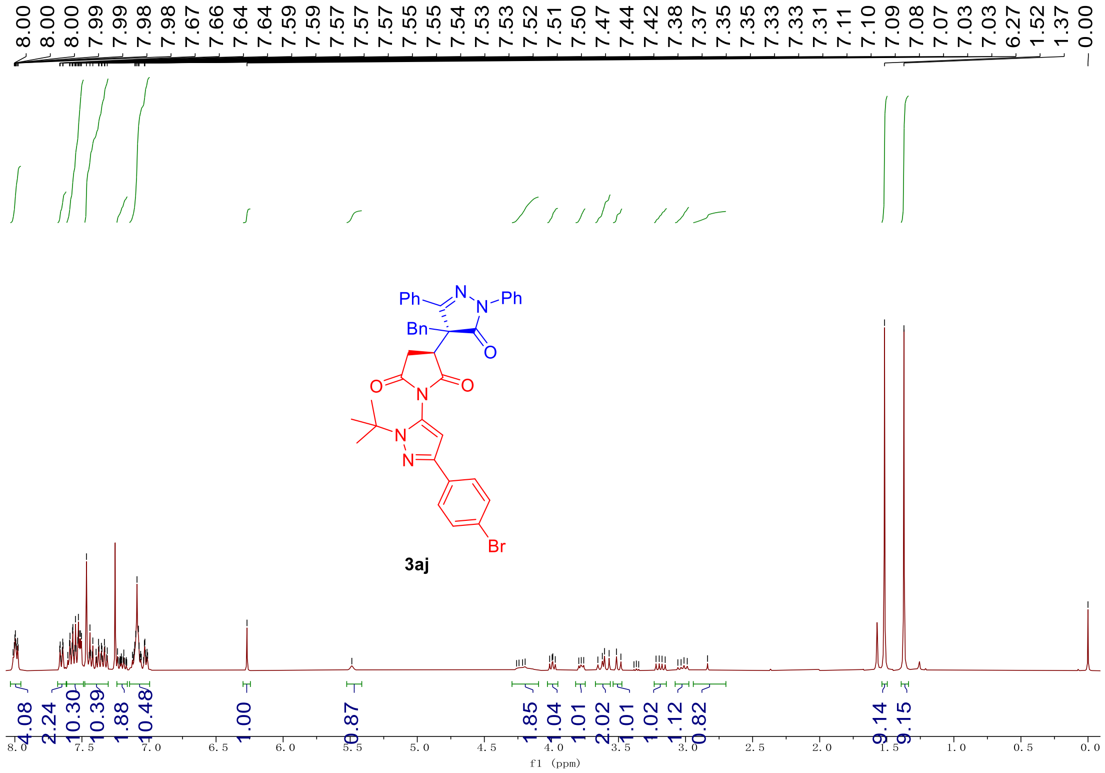
3aj

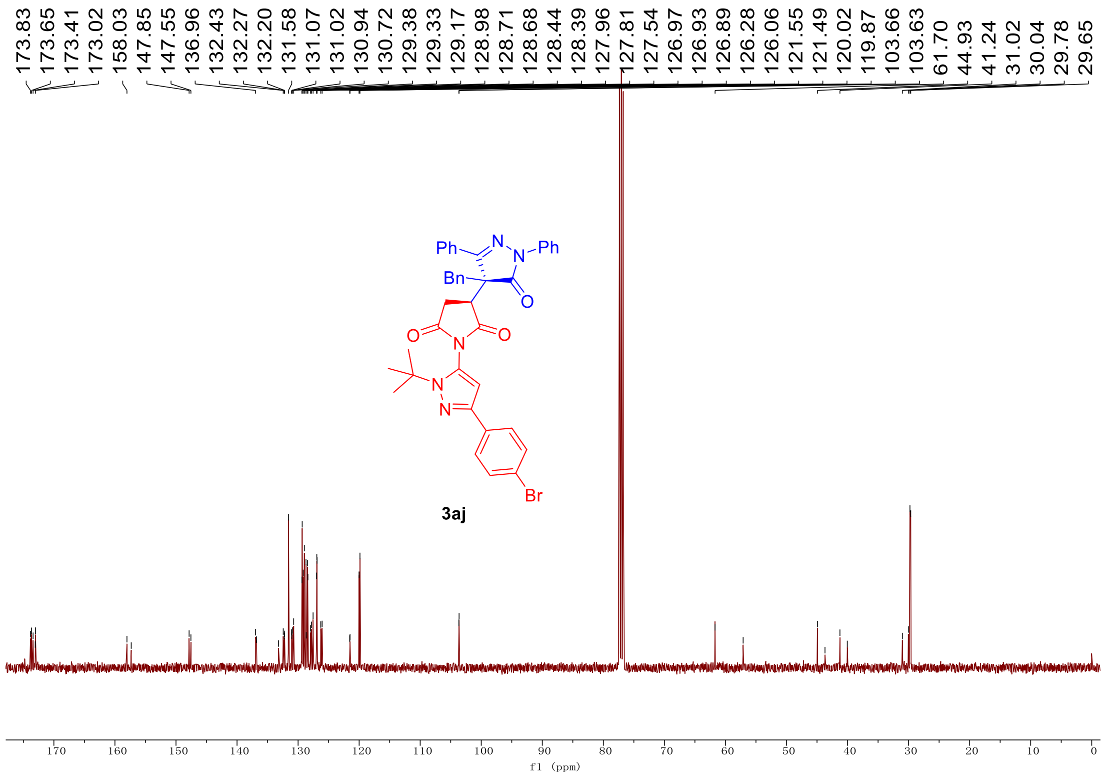
173.83
173.65
173.41
173.02
158.03
147.85
147.55
136.96
132.43
132.27
132.20
131.58
131.07
131.02
130.94
130.72
129.38
129.33
129.17
128.98
128.71
128.68
128.44
128.39
127.96
127.81
127.54
126.97
126.93
126.89
126.28
126.06
121.55
121.49
120.02
119.87
103.66
103.63
61.70
44.93
41.24
31.02
30.04
29.78
29.65
Ph
N
N
Ph
Bn
O
O
N
O
N
N
Br
3aj
f1 (ppm)

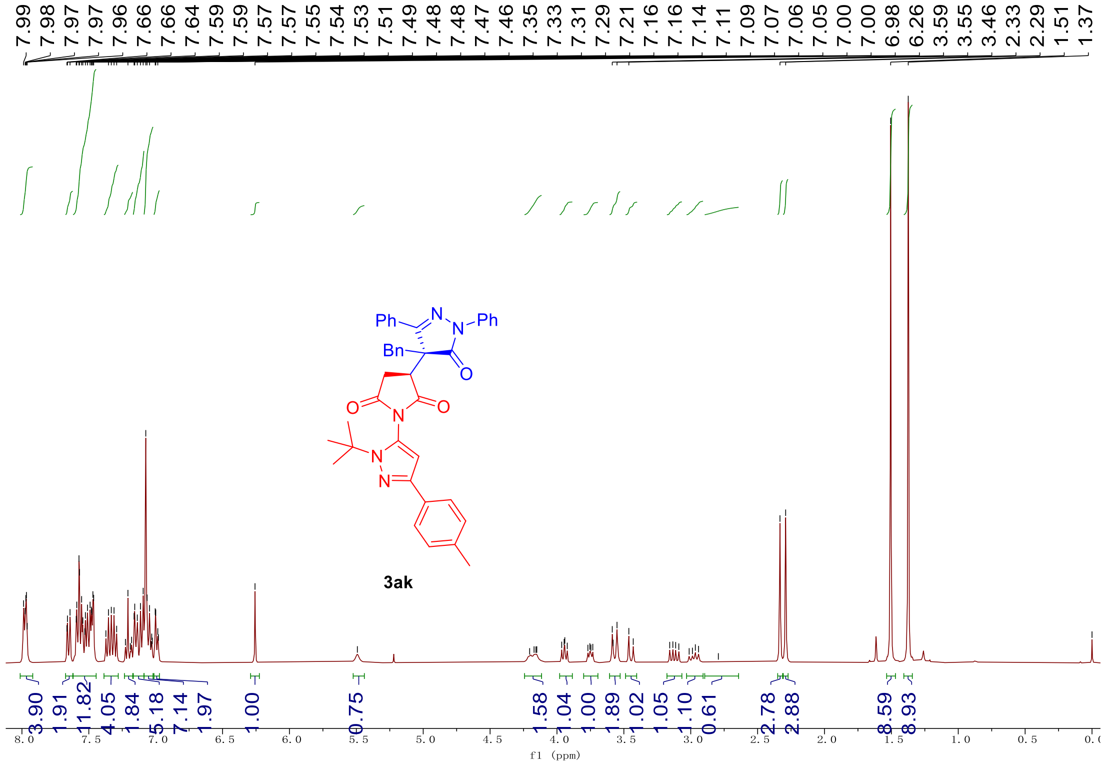
7.99
7.98
7.97
7.97
7.96
7.66
7.66
7.64
7.59
7.59
7.57
7.57
7.55
7.54
7.53
7.51
7.49
7.48
7.48
7.47
7.46
7.35
7.33
7.31
7.29
7.21
7.16
7.16
7.14
7.11
7.09
7.07
7.06
7.05
7.00
7.00
6.98
6.26
3.59
3.55
3.46
2.33
2.29
1.51
1.37
Ph
N
N
Ph
Bn
O
O
N
O
N
N
3ak
3.90
1.91
11.82
4.05
1.84
5.18
7.14
1.97
1.00
0.75
1.58
1.04
1.00
1.89
1.02
1.05
1.10
0.61
2.78
2.88
8.59
8.93
f1 (ppm)

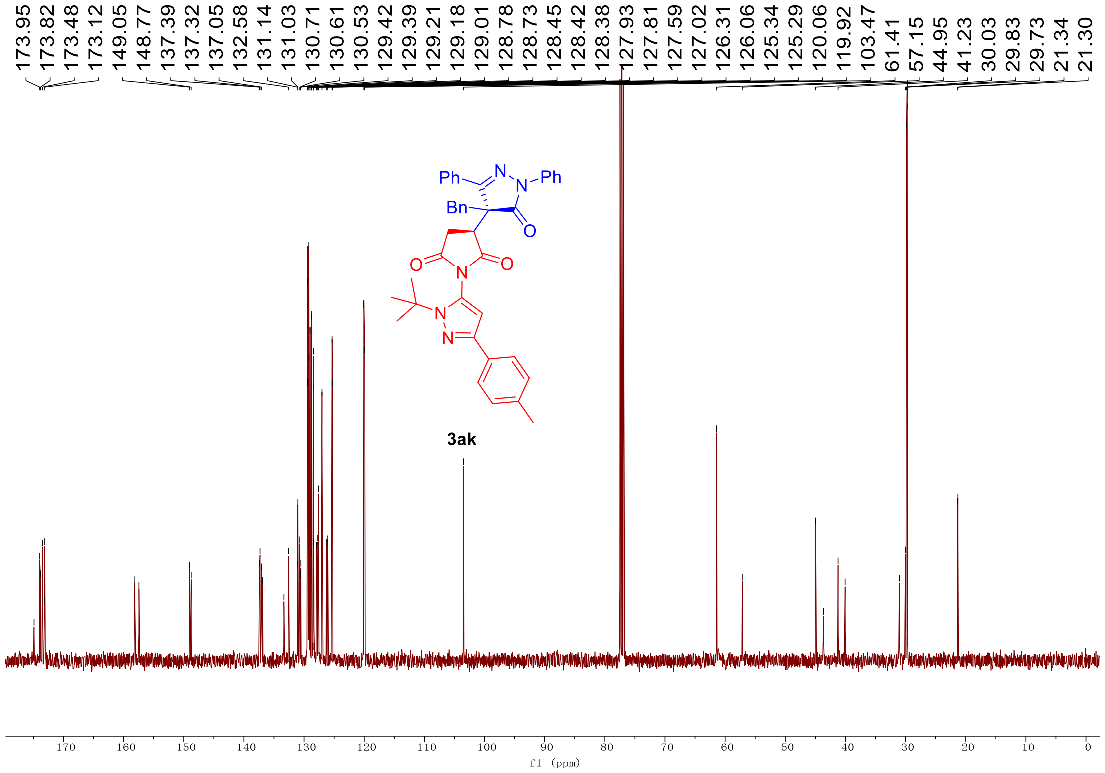
173.95
173.82
173.48
173.12
149.05
148.77
137.39
137.32
137.05
132.58
131.14
131.03
130.71
130.61
130.53
129.42
129.39
129.21
129.18
129.01
128.78
128.73
128.45
128.42
128.38
127.93
127.81
127.59
127.02
126.31
126.06
125.34
125.29
120.06
119.92
103.47
61.41
57.15
44.95
41.23
30.03
29.83
29.73
21.34
21.30
Ph
N
N
Ph
Bn
O
O
O
N
N
N
3ak
f1 (ppm)

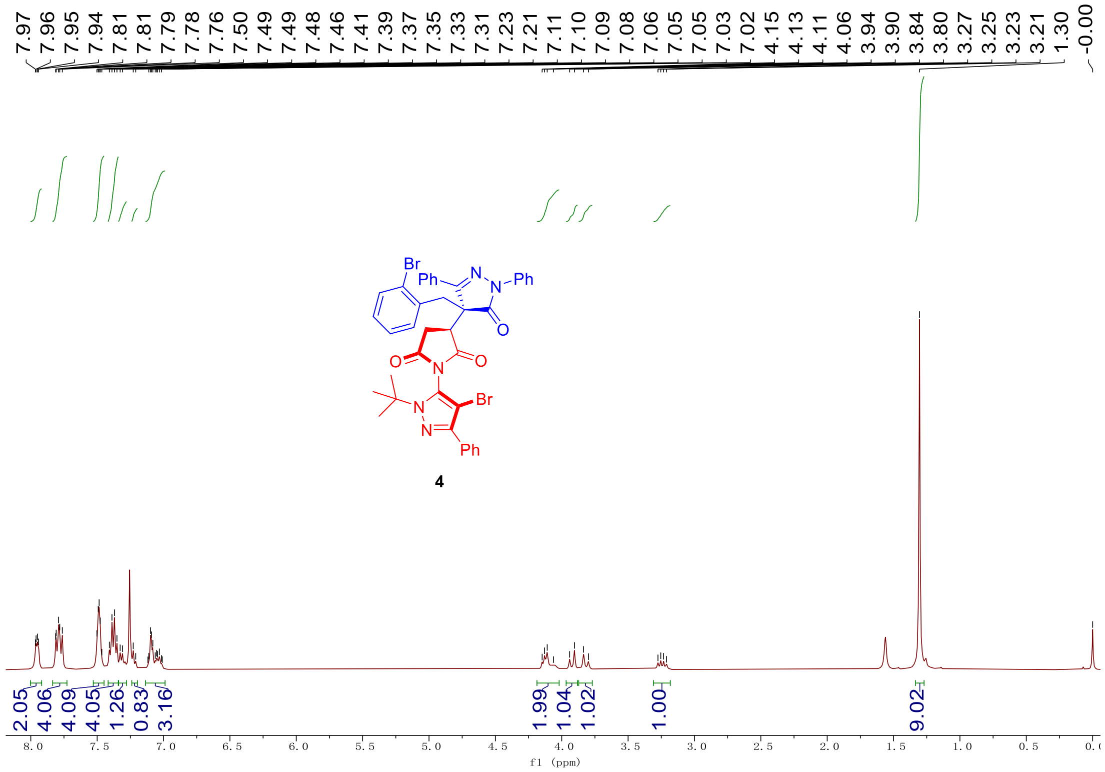
7.97
7.96
7.95
7.94
7.81
7.81
7.79
7.78
7.76
7.50
7.49
7.49
7.48
7.46
7.41
7.39
7.37
7.35
7.33
7.31
7.23
7.21
7.11
7.10
7.09
7.08
7.06
7.05
7.05
7.03
7.02
4.15
4.13
4.11
4.06
3.94
3.90
3.84
3.80
3.27
3.25
3.23
3.21
1.30
-0.00
Br
Ph
N
N
Ph
O
O
O
N
N
N
Br
Ph
4
2.05
4.06
4.09
4.05
1.26
0.83
3.16
1.99
1.04
1.02
1.00
9.02
f1 (ppm)

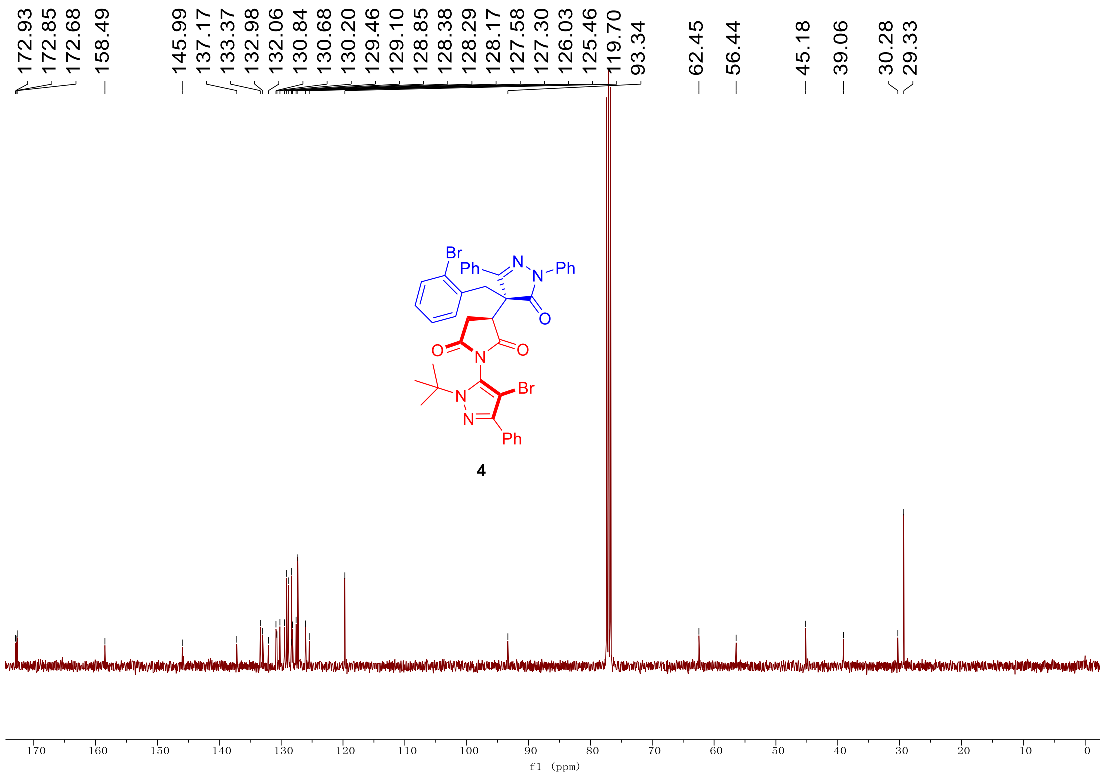
172.93
172.85
172.68
158.49
145.99
137.17
133.37
132.98
132.06
130.84
130.68
130.20
129.46
129.10
128.85
128.38
128.29
128.17
127.58
127.30
126.03
125.46
119.70
93.34
62.45
56.44
45.18
39.06
30.28
29.33
Br
Ph
N
N
Ph
O
O
N
O
N
Br
N
Ph
4
f1 (ppm)

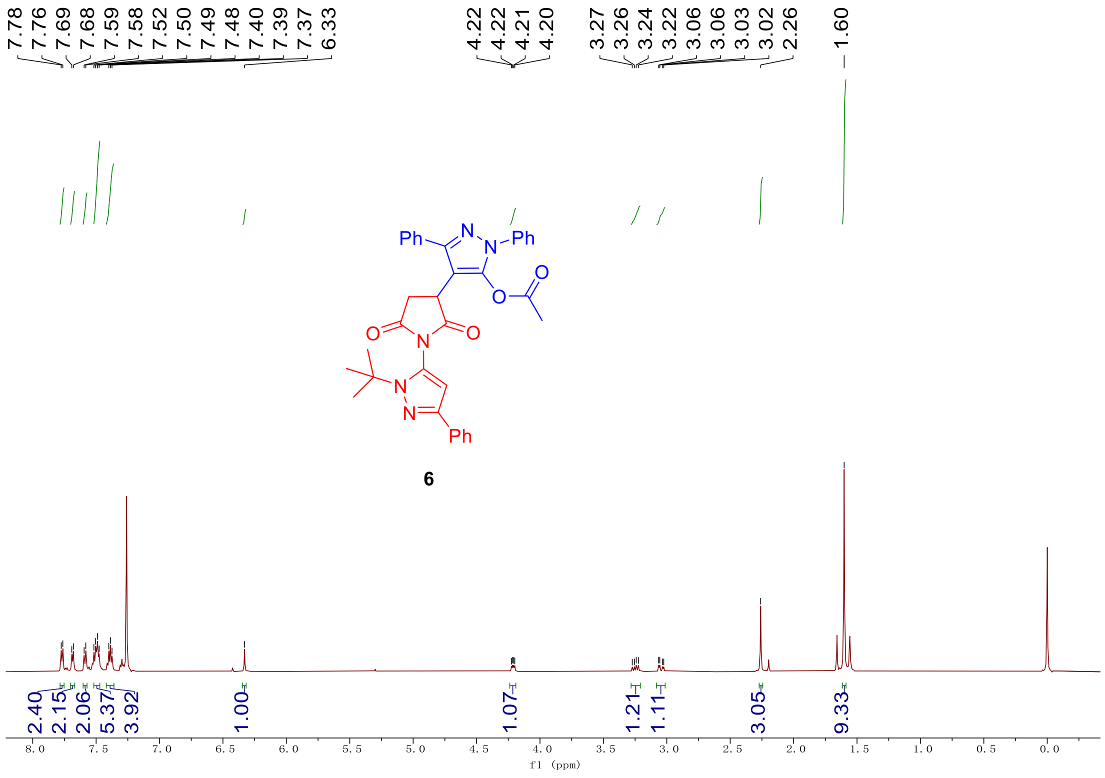
7.78
7.76
7.69
7.68
7.59
7.58
7.52
7.50
7.49
7.48
7.40
7.39
7.37
6.33
4.22
4.22
4.21
4.20
3.27
3.26
3.24
3.22
3.06
3.06
3.03
3.02
2.26
1.60
Ph
N
N
Ph
O
O
O
O
N
O
N
N
Ph
6
2.40
2.15
2.06
5.37
3.92
1.00
1.07
1.21
1.11
3.05
9.33
f1 (ppm)

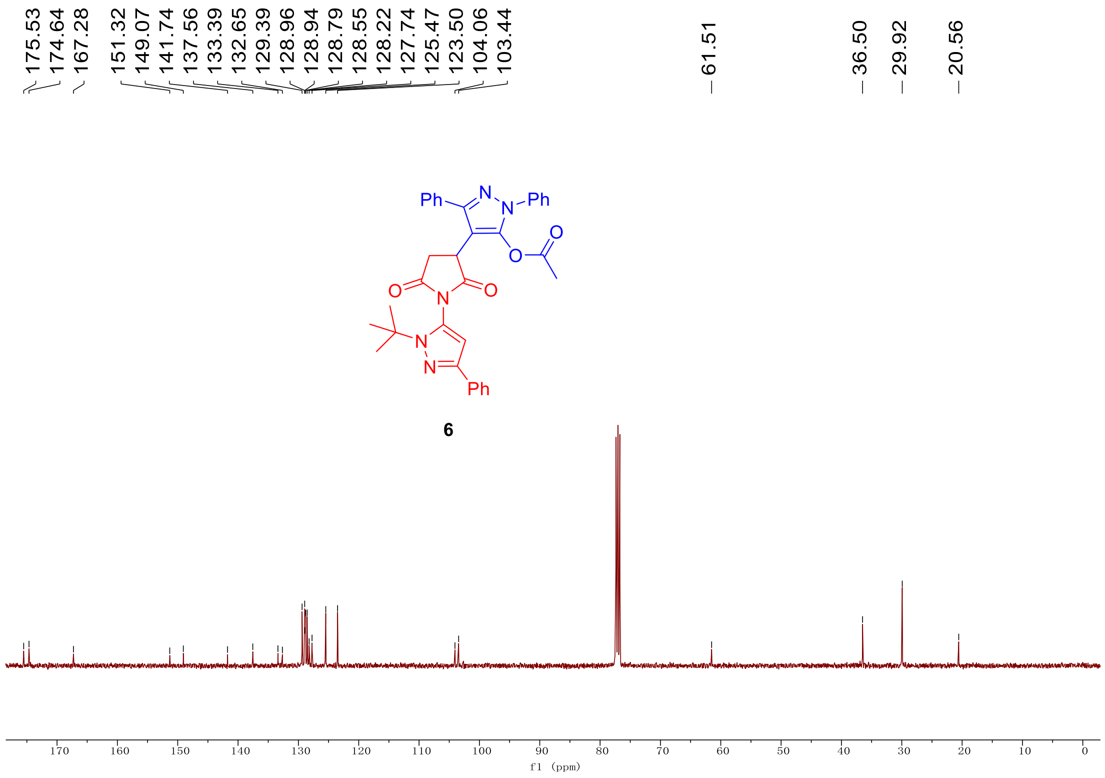
175.53
174.64
167.28
151.32
149.07
141.74
137.56
133.39
132.65
129.39
128.96
128.94
128.79
128.55
128.22
127.74
125.47
123.50
104.06
103.44
61.51
36.50
29.92
20.56
Ph
N
N
Ph
O
O
O
N
O
N
N
Ph
6
170 160 150 140 130 120 110 100 90 80 70 60 50 40 30 20 10 0
f1 (ppm)
